# Uncover the strength of polyhedral dicarbaboranes as the next generation of superbases in organocatalysis

**DOI:** 10.1038/s41467-026-77070-6

**Published:** 2026-08-26

**Authors:** Jan Vrána, Zuzana Kapustová, Martin Kamlar, Peter Šramel, Petr Vosáhlo, Zdeňka Růžičková, Josef Holub, Jan Veselý, Aleš Růžička

**Affiliations:** 1 Department of General and Inorganic Chemistry, Faculty of Chemical Technology, University of Pardubice, Pardubice, Czech Republic; 2 Department of Organic Chemistry, Faculty of Science, Charles University, Prague 2, Czech Republic; 3 Institute of Inorganic Chemistry, Czech Academy of Sciences, Husinec-Řež, Czech Republic

**Keywords:** Organocatalysis, Chemical bonding, Organometallic chemistry

## Abstract

Although boron compounds appear in many chemical transformations, it is hard to imagine, these usually Lewis acidic, species would act as Lewis bases or even base-catalysts. Here we show that the adduct *o-***2**, formed by the reaction of *closo*−1,2-dicarbadecaborane(10) with two equivalents of an *N*-heterocyclic carbene, exhibits superbasic properties with proton affinity surpassing that of the parent carbene as well as commercial superbases. It reacts selectively with C-acids, solvents and water, with its reactivity strongly influenced by substrate pK_A_. *o-***2** facilitates various Lewis base-catalyzed transformations—including aldol condensation, the Thorpe reaction, Michael addition, Robinson annulation and β-nitrostyrene polymerization—with exceptional efficiency and selectivity. Moreover, *o-***2** effectively catalyzes highly diastereoselective conjugate additions of 4-substituted pyrazolones to electron-poor olefins and spirocyclizations of pyrazolones with dibenzylideneacetones. A notable tolerance of catalyst to significant amounts of water and base-sensitive functional groups has also been observed.

## Abstract (web summary)

The authors synthesize a compound derived from a carborane bearing N-heterocyclic carbene, which exhibits superbasic properties with proton affinity surpassing that of the parent carbene as well as commercial superbases. The base facilitates various Lewis base-catalyzed transformations, including aldol condensation, the Thorpe reaction, Michael addition and Robinson annulation, with high efficiency and selectivity.

## Introduction

Modern chemistry of boranes—compounds containing electron-deficient boron, hydrogen, and other elements—has expanded across diverse fields, from hydroboration agents^1–3^, Lewis acid promoters^4–8^, Frustrated Lewis Pairs^9–11^, to organocatalysts^12–15^ as well as medicinal^16,17^ and material applications^18–23^. Clusters composed of multiple boron atoms—polyhedral boranes and, later, heteroboranes—have fascinated many chemists by their structural diversity and broad applications. Since their discovery more than a century ago, these have traditionally been considered electron-deficient and thus electrophilic, likely due to the presence of numerous electron-deficient boron atoms within their frameworks. In fact, the formation of boron clusters—especially when a heteroatom is present in the polyhedron—results in some of the most thermodynamically stable molecular compounds known to date, made possible by extensive electron density delocalization^24^. A contrasting interpretation of electron deficiency has emerged for dianionic *closo*-boranes and certain anionic or neutral *closo*-heteroboranes, which possess an ideal electron count and are now understood as three-dimensional aromatic species.

To date, most reactivity studies of polyhedral (carba)boranes have focused on skeletal transformations, metallation, nucleophilic substitution at C–H positions, and electrophilic or photochemical reactions involving halogenating and other agents. Although the vast majority of catalytic applications of polyhedral heteroboranes is associated with their metal complexes—classified into three categories, where^24^: *i)* the metal is part of the cage, *ii)* the metal is exoskeletal, but the substituted cage is part of the ligand system, or *iii)* the heteroborane acts as a weakly coordinating anion—some examples of direct catalytic activity do exist. One such example is the thermal oligomerization of isophthalonitrile to form triazines^25^. Carborane-supported, highly electrophilic silylium compounds have been reported as long-lived catalysts for hydrodefluorination of trifluoromethyl and nonafluorobutyl groups using readily available silanes under mild conditions^26^. Iodinated carboranes have demonstrated superior activity in halide abstraction reactions—comparable to superacidic agents—and some have shown organocatalytic activity in nitro-Michael reactions when used at 20 mol. % loading^27^ attributed to halogen bonding. The single-site, neutral Lewis superacidic character is further exemplified by C-substituted tris(*ortho*-carboranyl)borane^28^, which is capable of activating C-F bonds via a catalytic process. A similar phenomenon is observed in metal-free methane activation under mild conditions by a borenium cation bearing an electron-withdrawing cage C-substituent^29^. The electronic versatility of heteroborane cages—described as a dichotomy^30^—is further illustrated by electron-rich B9-connected carboranylphosphines^30,31^. A sulfonium complex has also been proposed to facilitate halogen transfer to arenes with the assistance of AgSbF_6_^32^.

In addition to reported molecular clusters used in acidic catalysis, efforts to heterogenize polyhedral boranes include the partial pyrolysis of decaborane(14) and the use of the resulting material, which contains Lewis-acid centers, for catalytic activation of silanes in the hydrogenation of various small molecules^33,34^.

Redox-active cluster compounds represent a rapidly developing area for application in organic synthesis^35^. For instance, Peters developed a diaryl-*o*-carborane radical anion appended with a Brønsted acid, acting as a proton-coupled electron transfer reagent capable of reducing azobenzenes, nitroarenes and fumarates^36^.

Another behavior in series of relatively unexplored boron nucleophiles is to react with various electrophiles. For example, magnesium diboranate complex with 4-dimethylaminopyridine is shown to act as an unambiguous nucleophile through its reactions with iodomethane, benzophenone and *N*,*N*‘-di-isopropyl carbodiimide^37^. The tetraaryl μ-hydridodiborane(4) anion revealed increased nucleophilicity of B–B bond proven by smooth reactivity with several substrates^38^. Similarly, the nucleophilic character of *closo*-hexaborate cluster anion was uncovered also in polyhedral borane series. The anion can engage in a nucleophilic substitution reaction with a wide variety of organic and main-group electrophiles^39^.

To the best of our knowledge, the only Lewis-base polyhedral (hetero)borane catalyst is bismethylthio-*C*-substituted *meta*-carborane capable of promoting halogenation of activated arenes under mild conditions using *N*-halosuccinimides^32^.

**Fig. 1:**
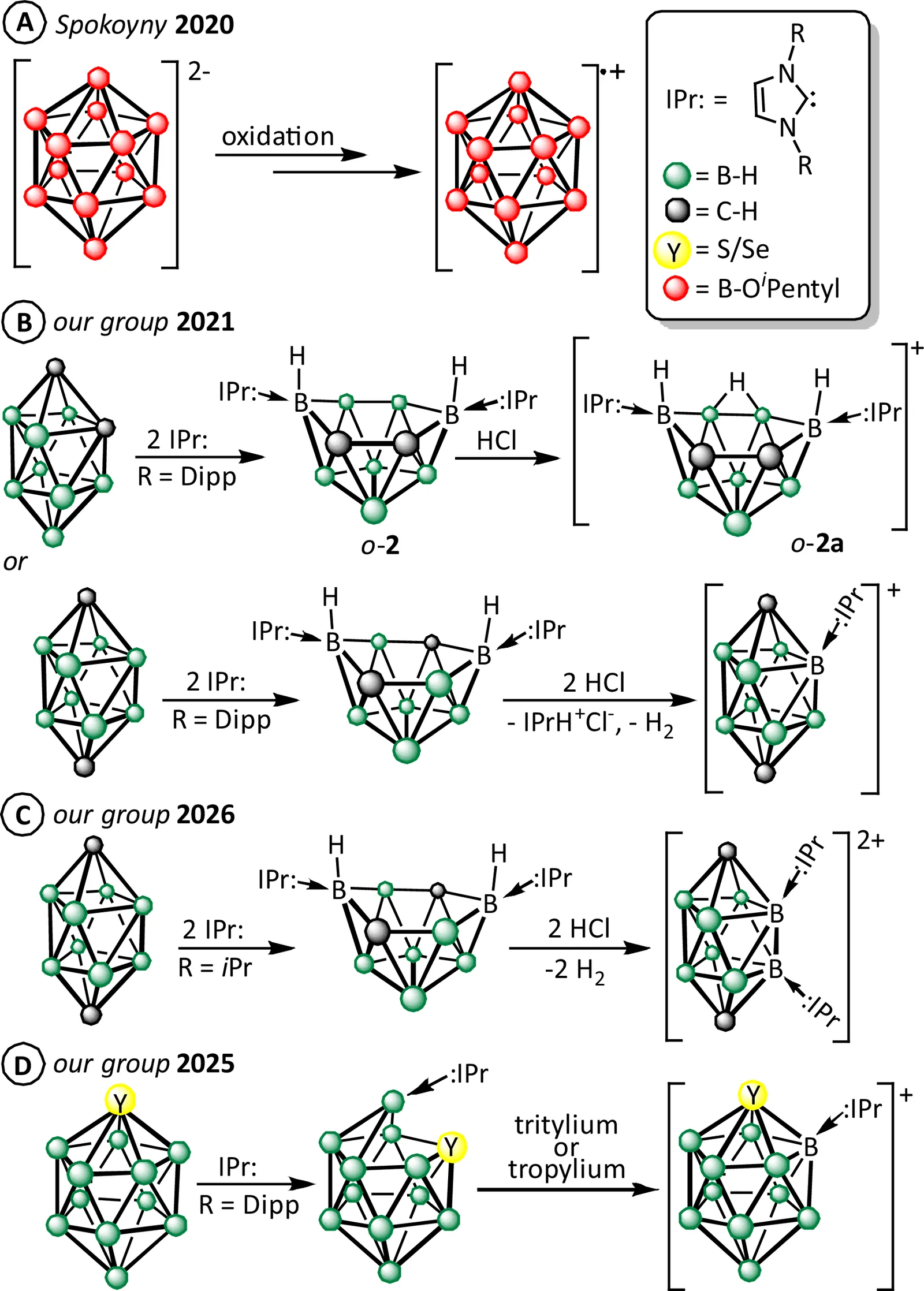
Pathways to cationic polyhedral heteroboranes. Examples of peralcoxylated 12-vertex radical cation (**A**), 10-vertex dicarbaborane cations (**B**), 10-vertex dicarbaborane dications (**C**) and 12-vertex thia/selenaborane cations (**D**).

Generally, it is rare to find cluster compounds occupying positively charged regions within molecular structures. With few exceptions—where the positive charge resides on the organic or inorganic shell surrounding the inner (hetero)boron cluster^40^ —no examples of such cationic clusters were known before 2020, when Spokoyny’s seminal report on peralkoxidated radical cation [B_12_(OR)_12_]^·+^ was disclosed (Fig. 1A)^41^. Inspired by Spokoyny’s strategy, we applied a similar approach by introducing adjacent electron density to ten-vertex dicarbaboranes or twelve-vertex chalcogenaboranes via *N*-heterocyclic-carbene coordination, followed by oxidation. The first thermally robust cationic ten-vertex dicarbaboranes (Fig. 1B)^42^ were synthesized through protonation of their bis-carbene adducts using HCl. The outcome of the reaction depends not only on the positions of the carbon atoms in original cluster, where the reaction of 1,2-dicarba isomer reacts to the nido-type cluster and the 1,10-dicarba isomer to the *closo* cation (Fig. 1B), but also on steric demand of the carbene used. Bigger carbene, decorated with bulky Dipp groups, reacts with the 1,10-dicarba isomer after protonolysis to monocationic cluster, while the presence of smaller isopropyl-substituted carbene enabled the formation of dicationic species (Fig. 1C)^43^. In parallel, cationic twelve-vertex chalcogenaboranes (Fig. 1D)^44^ were obtained via protonolysis with tropylium or tritylium tetrafluoroborate, followed by *closo*-cluster formation.

**Fig. 2:**
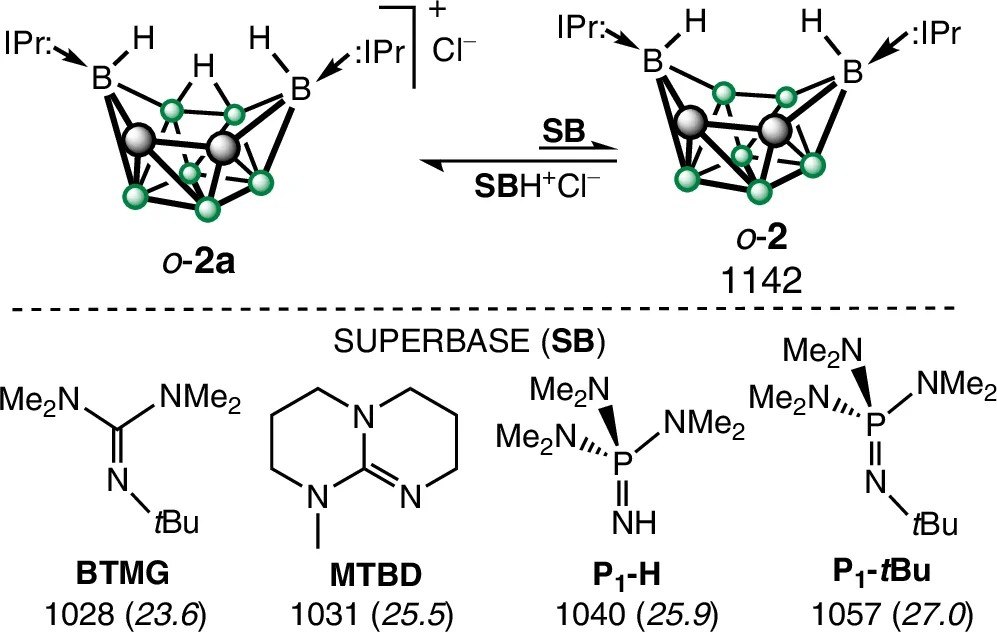
Reactivity of o-2a towards selected superbases (SB). Appropriate proton affinities (kJ/mol at M06-2X(D3)/6–311 + G(d,p) level) and pK_A_ values in DMSO (in italics in parentheses) are given below the abbreviation of each compound.

The isolation of stable and easily accessible cationic ten-vertex dicarbaboranes with a *nido* structure (Fig. 1B – *o*-**2a**) inspired us to explore potential reactivity arising from the proximity of hydrogen atoms, where the terminal hydrogen atom could act as a hydride, while the bridging hydrogen could serve as a proton source. The primary working hypothesis in this paper was to use *o*-**2a** in catalytically driven organic transformations exploiting its structural behavior, which is virtually not that far from the arrangement of zwitterionic species after a hydrogenation of frustrated Lewis pairs. In this paper, we show in contrast to our hypotheses *o*-**2a** is nearly inactive in acid-promoted reactions and hydrogenations, but its precursor exhibit unexceptional basic character and is able to promote several organic chemistry transformations.

## Results and discussion

Several reactions of high synthetic interest—leading to precursors of biologically active compounds or functional materials—were evaluated. These included: guanylation reaction between anilines and carbodiimides, ring-opening polymerization of lactides, catalytic spirocyclization yielding spiro-heterocyclic compounds with a benzofuranone motif, and a formal [4 + 2] cycloaddition affording cyclohexanone derivatives. Unfortunately, the use of *o*-**2a** (Fig. 1B), as an initiator or catalyst in these transformations generally resulted in either no conversion or negligible formation of the desired products. Details of these unsuccessful attempts are provided in SI. *o*-**2a** showed only partial success as a promoter of hydroboration reactions. For instance, the reaction of neat phenylacetylene with 1.2 molar equivalents of HBpin and 3 mol. % of *o*−**2a** at room temperature yielded the corresponding olefin in 26% yield after 18 h (see SI for details). Raising the temperature to 60 °C improved the yield to 66% under identical conditions. Solvent effects were detrimental: only traces of olefin were observed in toluene, and a 12% yield was obtained in THF. Catalyst loading also influenced the outcome, with 62% yield at 1 mol. % and 70% yield at 15 mol. % of *o*−**2a**. However, these results remain inferior to the best-performing systems in the literature. Applying the same protocol to styrene hydroboration led to less than 5% conversion at room temperature.

### Basicity of *o*-**2**

In parallel with the primary catalytic-activity evaluation of *o*-**2a**, computational investigations were also conducted on its neutral precursor, *o*−**2**. The most striking result of the computational studies was the exceptionally high proton affinity of *o*-**2**, calculated to be 1142 kJ/mol at the M06-2X(D3)/6–311 + G(d,p) level. This value exceeds that of its parent carbene, IPr:, by 66 kJ/mol, placing *o*−**2** among the strongest known basic organocatalysts. Experimental validation of this behavior was primarily carried out through reactivity studies of *o*-**2a**. This cationic carborane showed no reactivity with strong mineral acids or commonly used organic bases such as Et_3_N, DMAP or DBU. Complete deprotonation to regenerate neutral *o*-**2** occurred only in the presence of strong bases such as LDA or *t*BuOK. To assess the superbasic nature of *o*-**2** further, we performed a deprotonation test using the hydrochloride salt of 1,8-bis(dimethylamino)naphthalene (Proton Sponge), a benchmark compound at the threshold of superbase classification. *o*-**2** was able to deprotonate the substrate essentially quantitatively, yielding free proton sponge and providing direct experimental evidence of its superbasic character. In addition, similar competitive reactions of protonated form of *o*-**2,** *o*-**2a** with strongest—by 6—9 orders of magnitude stronger than Proton Sponge—commercially available superbasic guanidines and phosphazenes (**SB**, Fig. 2) were carried out to explore these properties. Both guanidines and the phosphazene **P**_**1**_**-H** deprotonated *o*-**2a** partially forming a similar dynamic equilibrium between the neutral and ionized form shifted significantly towards the parent *o***-2a**. Interestingly, the *o*-**2**/*o*-**2a** ratio was nearly identical (~15/85). Thus, we assume that the protonation/deprotonation of *o*-**2** is driven kinetically and the pK_A_ cannot fully evaluate the basicity of this carborane. The phosphazene **P**_**1**_**-*t*Bu** revealed no deprotonation of *o*-**2a**, most probably due to the steric reasons. To screen the reversibility of the reactions, we tested also the reversed reactions (**SB**H^+^ and *o*-**2**) in acetonitrile as the most suitable solvent previously used to describe the *o***-2**/weak acid equilibria. The NMR spectra (CD_3_CN, 500 MHz) were recorded immediately over the temperature range of −20 to 60 °C, and no effect of temperature was observed. Inspecting all the spectra, the same result was obtained, the **SB**H^+^ has been only partially deprotonated (~15%), confirming there is no thermodynamic equilibrium.

Therefore, we calculated the protonation affinities, which revealed, that *o*-**2** is overpasses all the **SB**s by 100 kJ/mol (Fig. 2), being thus one of the strongest neutral bases. The most striking difference between *o*-**2** and the tested superbases is the behavior towards water. Guanidines and phosphazenes are hygroscopic and the interaction with water implies higher loading in catalytic reactions or the water has to be removed, *e. g*. by molecular sieves. On the contrary, *o*-**2** fully tolerates water. When *o*-**2** is dissolved in water (combined with acetonitrile for increase solubility), compound *o*-**2w** is isolated, from which, after solvent vacuo evaporation at room temperature pure *o*-**2** is recovered. To the best of our knowledge, this phenomenon has not been described for any base.

**Fig. 3:**
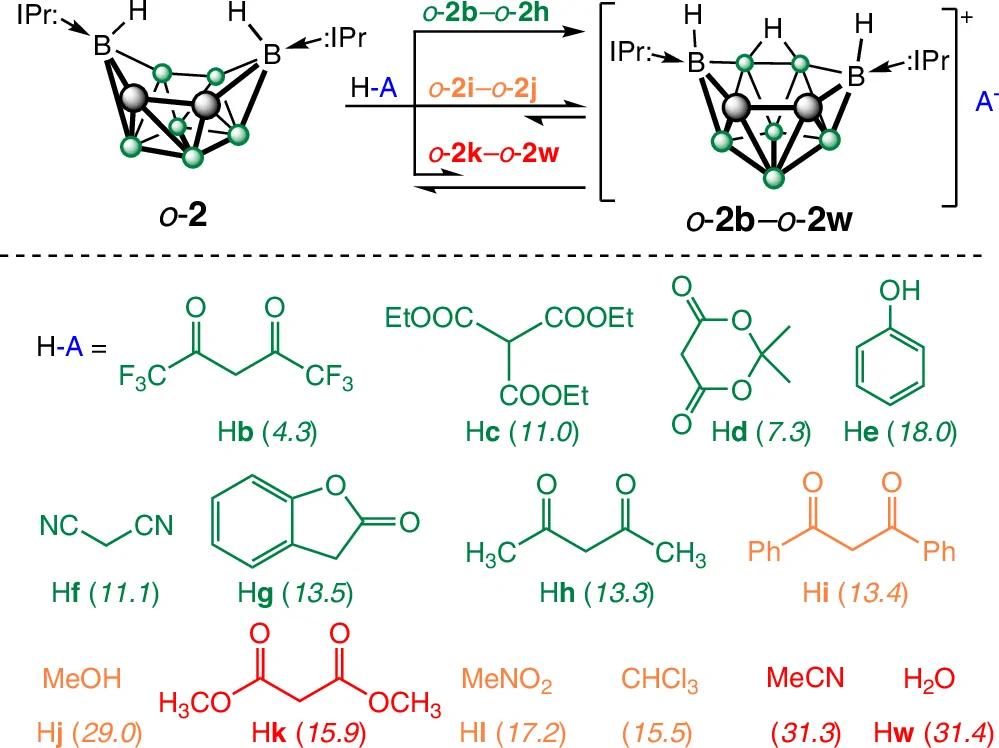
Reactivity of o-2 towards C-acids and protic solvents. Appropriate pK_A_ values in DMSO are given in italics in parentheses.

The superbasic properties of *o*-**2** prompted us to investigate its reactivity with a range of acids, solvents and water. As schematically illustrated in Fig. 3, four distinct types of reactions have been identified. Depending on the nature and acidity of the substrate, an equilibrium is established between the strong base and its conjugate. The equilibrium has been monitored by analyzing the reaction mixtures using both ^1^H (CD_3_CN, 500 MHz) and ^11^B NMR spectroscopy.

In the first group of reactions, involving relatively strong acids, the chemical equilibria are shifted entirely toward the products. Specifically, hydrogen chloride (or hydrochloric acid – H**a**), hexafluoroacetylacetone (H**b**), triethyl methanetricarboxylate (H**c**) and Meldrum’s acid (H**d**) react smoothly via protonation of *o*-**2**, yielding the corresponding anions and the cation *o*-**2**H^+^. The reaction of the less acidic phenol (H**e**) also proceeded well, although the isolated product was an adduct of *o*-**2e** with two phenol molecules. The reaction of *o*-**2** with malononitrile (H**f**) is even more complex: one molar equivalent of H**f** reacts more slowly, and the equilibrium can be shifted by using less polar solvents (e.g. hexanes, benzene, diethyl ether), from which the product *o*-**2f** precipitates. This compound appears to be highly reactive because it cleaves otherwise inert solvents such as dichloromethane or acetonitrile. When an excess of malononitrile is used, its condensation with malononitrilate (Thorpe reaction) leads to the formation of *o*-**2f’**. A similar reaction with 2-coumaranone (H**g**) consistently proceeds with a 1:2 stoichiometry regardless of the initial ratio, yielding a Claisen-condensation product of the two heterocycles (see Supplementary page S34).

In the second group of substrates, equilibria are established between *o*-**2** and less acidic compounds, such as acetylacetone and diphenyl-1,3-propanedione (H**h** and H**i**), and their ionized forms, with an 85:15 ratio (see Supplementary Figs. S42, S49). These equilibria are only marginally affected by temperature variations in the range of −40–60 °C. However, the products tend to precipitate as colorless powders in less polar solvents, similar to *o*-**2f**. The addition of two molar equivalents of the organic substrate is sufficient to maximize product yield, as confirmed by both ^1^H (CD_3_CN, 500 MHz) and ^11^B NMR spectra (Supplementary Figs. S47, S48). This transformation is also visually evident: The parent *o*-**2** is yellow, whereas all products are colorless.

The reactivity of carborane *o*-**2** with third-group substrates—methanol, dimethyl malonate and nitromethane—resembles that observed with diketones. However, the equilibrium is strongly biased toward the starting compounds. For example, in the ^1^H NMR spectra of reactions involving *o*-**2** with one, three or ten equivalents of methanol, no protonation was detected. Interestingly, dissolving *o*-**2** in neat methanol led to immediate discoloration, indicating full conversion to *o*-**2j**. A parallel reaction with deuterated methanol has confirmed the incorporation of deuterium at the B–B bridging position, as corroborated by the disappearance of the corresponding signal in the ^1^H NMR spectrum (see below). Notably, this compound can only be prepared in solution, because any attempt to remove the solvent shifts the equilibrium back to the starting compounds—even at room temperature. Dimethyl malonate and nitromethane protonate *o*-**2** more efficiently, because increasing concentrations of the corresponding products have been identified in reactions with one, three or ten equivalents of the organic substrate (Supplementary Figs. S60–S67). Full conversion to the desired compounds *o*-**2k** and *o*-**2l** was achieved only when *o*-**2** was treated with neat reagent. Surprisingly, the reaction of *o*-**2** with water—typically a dead end for most of carboranes with other than 12 vertexes—proceeds to an equilibrium similar to that seen with methanol, yielding protonated *o*-**2** and most likely the OH^⁻^ counterion (Supplementary Figs. S68–S71. Since the resulting anions are only weakly coordinated to the cationic moieties (specifically the CH=CH units of the carbenes), they exhibit high reactivity. For instance, anions derived from weaker acids slowly deprotonate dichloromethane.

Unfortunately, within all three substrate groups, no clear correlation could be established between the acidity of the substrate (pK_A_ value) and the extent of the respective acid–base equilibrium, even though the K_A_ values of the substrates (excluding HCl) differ by more than eight orders of magnitude.

It is worth mentioning that all the reactions are readily monitored by ^1^H (CD_3_CN, 500 MHz)^13^,C and ^11^B NMR spectroscopy. In particular, the resonances of the bridging hydrogen atoms in B–H–B moieties appeared in a narrow range −4.18–(−4.25) ppm, serving as clear indicators of cation formation. A more complex, yet feasible, approach involved tracking broad signals in the ^11^B NMR spectra, with integral intensity ratios of 2:2:1:2:1 corresponding to both neutral and cationic species. Only minor differences in chemical shifts were observed for the B1, B3, B6, and B9 atoms. The ^1^H and ^13^C spectra of the anions did not show any major differences from those of the neutral precursors, except for the signals of the deprotonated C–H groups, which—as reported in the literature^42^—were shifted downfield.

The molecular structures of *o*-**2b,** *o*-**2c,** *o*−**2f’,** *o*-**2g**, and *o*-**2i** have been unambiguously confirmed by single-crystal X-ray diffraction analysis. The cationic moieties in these compounds exhibit structural parameters identical to those previously reported for *o*-**2a**. Among the anionic components, two organic anions—(acac-F_6_)^−^ and (PhCOCHCOPh)^−^—are known in their free forms. However, the [C(COOEt)_3_]^−^ anion, derived from *o*-**2c**, has not yet been reported as uncoordinated to a metal center. The negative charge is delocalized across the planar C(COO)_3_ core, as evidenced by the narrow ranges of C–C (1.419(2)–1.467(2) Å) and C=O (1.226(2)–1.227(2) Å) bond lengths. This anion interacts weakly through delocalized bonds and oxygen atoms with the hydrogen atoms of the carbene moieties, which are perpendicularly coordinated from both sides of the anion. The structure of the [NCC(=C(NH_2_)CHCN)CN]^−^ anion (from *o*-**2f’**) closely resembles that of a neutral malononitrile-derived condensation product, with the only exception of the shortened C67–C68 bond (Supplementary Fig. S34), measured at 1.379(3) Å (Δ = 0.123 Å), which is consistent with the formation of a multiple bond. X-ray diffraction analysis has also confirmed the structure of the [2-(CH_2_CO(C_8_H_5_O_2_))C_6_H_4_O]^−^ anion, originating from 2-coumaranone in *o*-**2i**.

Based on the collected data, it is possible to hypothesize several potential applications of *o*-**2** in organic synthesis and catalysis: i) Base-catalyzed transformations: Owing to its superbasic character, o-**2** may serve as an efficient catalyst in base-mediated reactions. This is further supported by its reversible interactions with weaker acids and its stability across various solvent systems, ii) C–C coupling reactions: Stoichiometric evaluations using the Claisen condensation and the Thorpe reaction of H**f** and H**g** have prompted further exploration of o-**2** in diverse coupling methodologies, iii) The activation of weak electrophiles: The reactivity of “naked” anions derived from weak acids that o-**2** could facilitate transformations involving otherwise unreactive electrophilic substrates, iv) Ring-opening polymerization: the observed γ-lactone ring opening in 2-coumaranone implies that o-**2** may be applicable in initiating ring-opening polymerizations, and v) Hydroelementation reactions: finally, *o*-**2** shows promise in hydroelementation processes, potentially expanding its utility in selective bond-forming reactions.

### Organocatalysis

Organocatalyzed reactions leading to the formation of new carbon–carbon (C–C) bonds are among the major objectives in synthetic organic chemistry, yet and (carba)boranes have never been employed in Lewis-base organocatalysis. Therefore, we decided to investigate the catalytic properties of the carborane derivative *o*-**2** in different types of C–C bond-forming reactions, such as conjugate addition and aldol reactions.

The first test reaction was the Knoevenagel condensation of malononitrile and benzaldehyde. The first reaction was carried out as an NMR experiment at room temperature in deuterated acetonitrile, using 5 mol. % of *o*-**2** as the catalyst. Upon mixing, the catalyst was fully protonated, and the condensation proceeded with complete conversion within two hours. It is worth noting that the water formed during the reaction had no influence on the catalyst’s performance. Next, to compare the activity with other superbases, we conducted the Knoevenagel condensation catalyzed by *o*-**2,** **MTBD** and **P**_**1**_**-H** (Table 1). The **SB** catalyzed condensations required considerably higher amount of the superbase, highlighting again the water-tolerance of the carborane *o*-**2**. Moreover, *o*-**2** facilitates selectively the condensation, while **MTBD** and **P**_**1**_**-H** revealed the byproduct of Thorpe reaction, prepared in the stoichiometric reaction of *o*-**2** with malononitrile (*o*-**2f’**).

**Table 1:** Knoevenagel condensation of malononitrile and benzaldehyde catalyzed by *o*-**2**


Entry	Catalyst	Cat. loading (mol%)	Conversion^a^ (%)	Time	A/B ratio^a^ (%)
1	*o*-**2**	0.5	60	24	60/0
2	*o*-**2**	0.5	90	48	90/0
3	*o*-**2**	1	21	2	65/0
4	o-**2**	1	99	16	99/0
5	*o*-**2**	3	79	2	79/0
6	*o*-**2**	3	97	6	97/0
7	*o*-**2**	5	99	2	99/0
8	**MTBD**	5	56	2	53/3
9	**MTBD**	5	94	24	89/5
10	**MTBD**	10	61	1	56/5
11	**MTBD**	10	65	2	59/6
12	**MTBD**	20	97	1	87/10
13	**MTBD**	20	88	2	78/10
14	**P**_**1**_**–H**	10	85	1	76/9
15	**P**_**1**_**–H**	10	91	2	79/12
16	**P**_**1**_**–H**	20	80	1	60/20
17	**P**_**1**_**–H**	20	82	2	62/20

^a^Determined by ^1^H NMR (CD_3_CN, 500 MHz).

To broaden the scope of catalyzed reactions, the classical base-induced aldol condensation was selected. The aldol condensation reaction is a fundamental organic transformation that facilitates the formation of C–C bonds^45^. This reaction can be catalyzed by various Lewis or Brønsted acids or bases. Our objective (Table 2) was to utilize *o*-**2** as a basic catalyst in the model aldol-condensation reaction of butanal to form 2-ethylhex-2-enal under mild conditions. The catalyst *o*-**2** was investigated as a non-nucleophilic base where the α-hydrogen of butanal is deprotonated to form the enolate, which subsequently undergoes a nucleophilic attack on the carbonyl group of another butanal molecule. The reaction was conducted at 21 °C under an argon atmosphere, resulting in a poor yield of 32% within 24 h using 10 mol. % of the catalyst (entries 1 and 2). Reducing the catalyst loading to 2 mol. % provided an excellent yield of 92% (entries 3 and 4) within two hours, whereas 1 mol. % was still sufficient, giving a 97% yield after 24 h. The improved performance is likely a consequence of the shift in equilibrium between the neutral and anionic forms of *o*-**2** described above. Further reduction of the catalyst loading led to poorer results, likely due to the presence of poisoned and deactivated catalyst, which did not catalyze the reaction. In these reactions, the catalyst performance was sensitive to air contamination. Interestingly, it not negatively affected by the water produced during the aldol condensation, but the addition of water counterintuitively increased the yield of 2-ethylhex-2-enal (entries 7–10). This may be attributed to the protonation of the catalyst and the formation of [*o*-**2**H]^+^ (OH)⁻, which remains catalytically active, or to the above-described equilibria involving stoichiometric addition of weak acids to *o*-**2**. Conversely, a higher amount of water protects the catalytically active species by shifting the equilibrium in the desired direction. At higher catalyst concentrations, an induction period of approximately one hour was observed. This can be attributed to the gradual formation of water, slowly shifting the equilibrium from *o*-**2** to *o*-**2**H^+^, thereby accelerating the reaction. A significant increase in activity was observed when reactions conducted at 30 °C instead of 21 °C were directly compared (Fig. 1, entries 11 and 12 vs. 3 and 4), while catalyst decomposition was observed above 60 °C.

**Table 2:** Aldol condensation of butyraldehyde catalyzed by *o*-**2**


Entry	Catalyst	Cat. loading (mol%)	Time (h)	Yield (%)^a^
1	*o*-**2**	10	1	<5
2	*o*-**2**	10	24	32
3	*o*-**2**	2	1	24
4	*o*-**2**	2	2	92
5	*o*-**2**	1	2	71
6	*o*-**2**	1	24	97
7	*o*-**2**	10^b^	2	24
8	*o*-**2**	10^b^	24	90
9	*o*-**2**	2^b^	2	32
10	*o*-**2**	2^b^	24	99
11	*o*-**2**	2^c^	1	57
12	*o*-**2**	2^c^	2	95
13	**MTBD**	1	1	<5
14	**MTBD**	1	24	76
15	**MTBD**	5	2	<5
16	**MTBD**	5	24	98
17	**P**_**1**_**-H**	1	2	<5
18	**P**_**1**_**-H**	1	24	<5
19	**P**_**1**_**-H**	5	2	25
20	**P**_**1**_**-H**	5	24	97

^a^Determined by ^1^H NMR (CD_3_CN, 500 MHz).

^b^20 mol. % of water was added with the catalyst.

^c^30 °C.

Similar trend as for Knoevenagel condensation has been found in the aldol condensation of butyraldehyde. **MTBD** and **P**_**1**_**-H** required five times higher loading to match the performance of *o***-2**.

The catalytic activity of *o*-**2** was further evaluated in the cross-aldol reaction between butyraldehyde and non-enolizable benzaldehyde. In this case, the formation of the desired aldol product was negligible. Instead, only products resulting from self- and cross-aldol condensation were detected (for details, see SI). These findings suggest that the catalyst *o*-**2** does not exert a significant influence on the selectivity of the cross-aldol reaction.

**Fig. 4:**
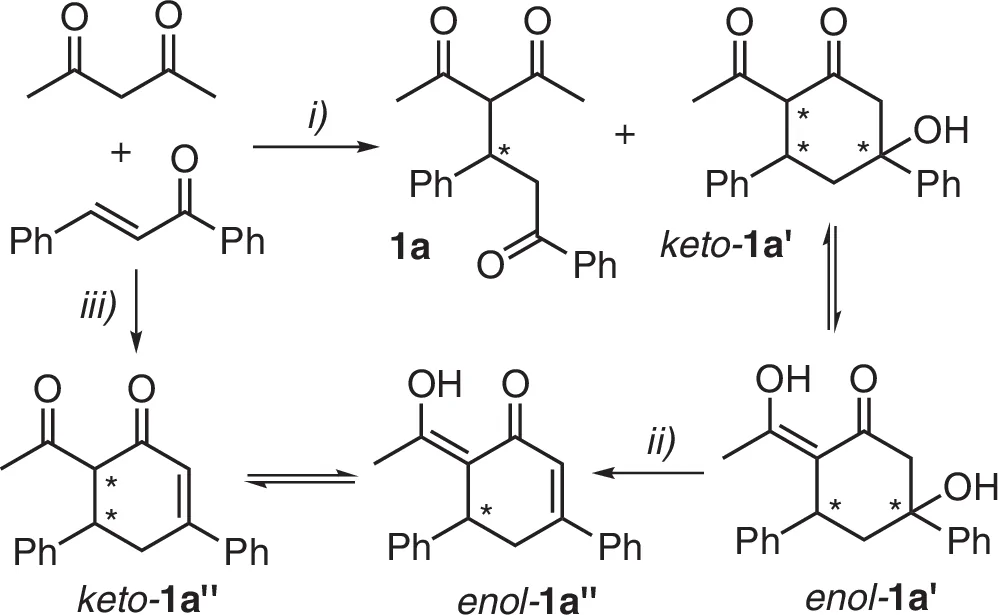
The model Michael addition of acetylacetone to *trans*-chalcone and subsequent dehydration to the Robinson annulation product *keto*-1a”. Conditions: *i)* 1–4 mol. % *o-*2; *ii)* 80 °C, two days, or I_2_ (1.1 eq), RT, two days; *iii)* 1–4 mol. %, 80 °C, two days.

Among the many developed methods, the Michael-type reaction has been extensively investigated over the past decades and has proven to be a highly significant tool for constructing valuable and complex organic compounds^46–48^. Aminocatalysis and Lewis-base catalysis, commonly represented by Cinchona alkaloids, also play an important role in this area^49–51^. As a model reaction for optimization, we selected the Michael conjugate addition of acetylacetone to *trans*-chalcone (Fig. 4). We primarily tested the influence of catalyst *o*-**2** loading, the reaction time, temperature and solvent. Interestingly, it was discovered that, in addition to the desired Michael adduct **1a’**, a cyclic intermediate product **1a”** of the Robinson annulation is also formed. The final product ratio strongly depends on the parameters mentioned above. The best results in terms of *trans*-chalcone conversion—exceeding 80%—were achieved with 3 mol. % of *o*-**2** and a reaction time of over two hours (an initiation period of one hour is needed at RT). Selectivity toward the Michael product is higher at shorter reaction times, while Robinson products dominate after extended reaction periods. More detailed information is summarized in Supplementary Tables S10–12.

Based on the obtained optimization data and our practical experience with model reactions, we concluded that a subsequent reaction scope should be carried out. Specifically, reactions of *trans*-chalcone or β-nitrostyrene with various donors were performed using 3 mol. % of *o*-**2** in CD_3_CN at room temperature, with ^1^H-NMR progress monitoring after 1 h, 1 day and 7 days. More detailed information is summarized in Supplementary Tables S14, S15.

**Fig. 5:**
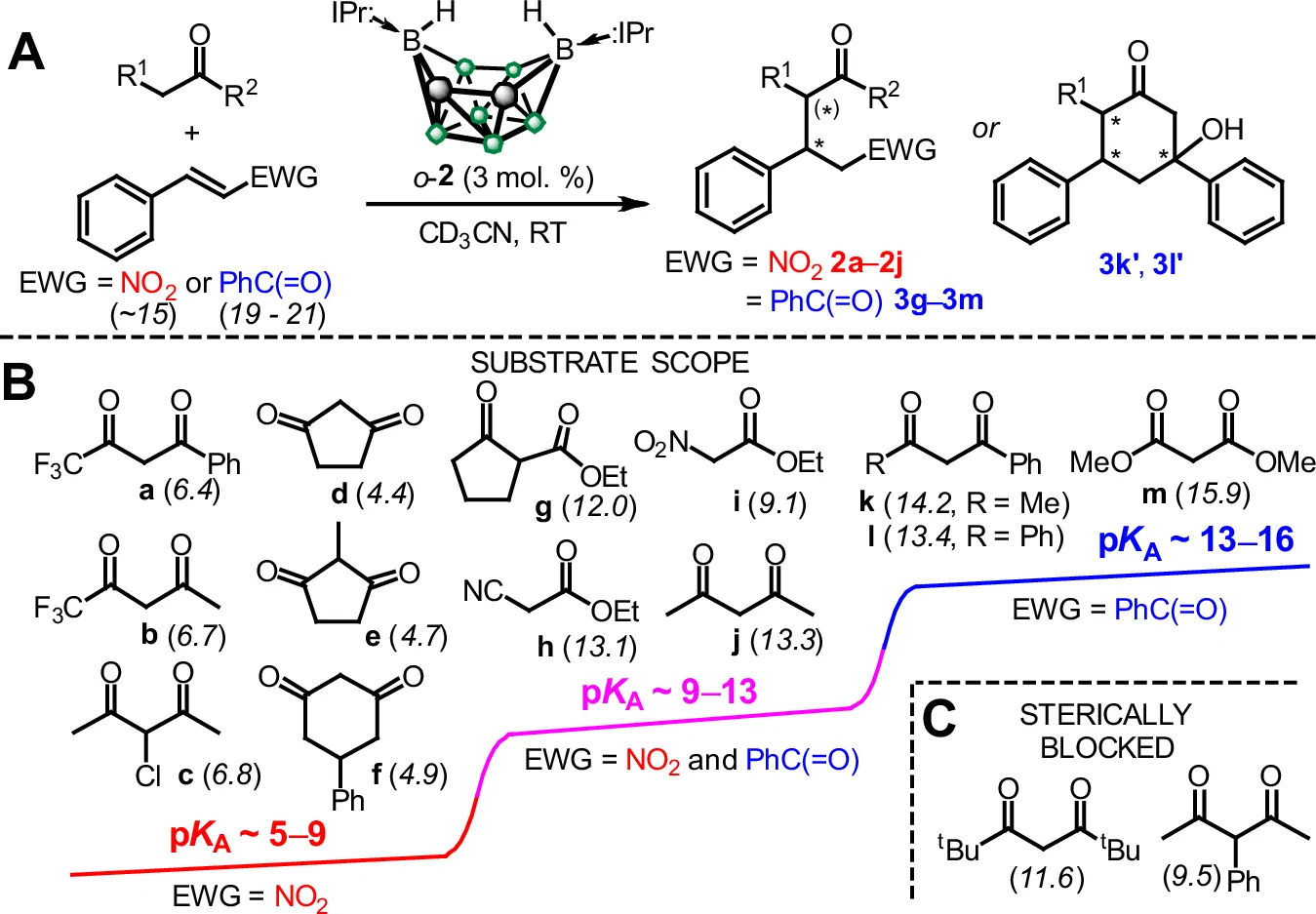
Acidity-dependent reactivity of Michael-donor substrates catalyzed by *o*-2. Michael addition reactions of β*-*nitrostyrene and *trans*-chalcone (**A**). The correlation between the reaction outcome (**B**) is illustrated using the pK_A_ values of the acids a–m in DMSO. Unreactive Michael-donors are presented in (**C**).

To summarize the reaction scope (Fig. 5), the most acidic substrates **a**–**f**, with p*K*_A_ values between 5 and 9, readily react with β*-*nitrostyrene to form the desired products but remain unreactive toward *trans*-chalcone. Both transformations are feasible for substrates **g**–**j**, which possess medium acidity (pK_A_ values of 9–13), while the most basic substrates (pK_A_ ~13–16) in the series **k**–**m** react exclusively with *trans*-chalcone. When β*-*nitrostyrene is exposed to substrates **k**–**m** under identical conditions, it undergoes quantitative polymerization. The polymerization result is the same as reported in the literature, totally insoluble gummy material with no chance to perform GPC measurement in any solvent, melting point of 285 °C and absence of peak around 1630 cm^−1^ (attributable to vibration of double bond)^52^. This behavior is attributed to the presence of the most acidic proton in the system and may serve as a precedent for the polymerization of other polar monomers^53^. Despite reasonable acidity, some reagents have proven unsuitable due to steric hindrance (Fig. 5C).

Moving into a more specific area of a catalytically driven reactivity with potential applications, we selected a model reaction involving conjugate addition of pyrazolone derivatives. These compounds are known for their strong nucleophilicity, notable biological activity^54,55^ and ability to react with electron-deficient olefins to form spirocyclic compounds via a double Michael cascade—sometimes even in an enantioselective fashion^56,57^. Specifically, we investigated the catalytic performance of *o*-**2** in the 1,4-addition reaction between β-nitrostyrene derivatives and pyrazolones. Our study began with screening the reaction between 4-benzyl-5-methyl-2-phenylpyrazol-3-one (**4d**) and β-nitrostyrene **5a** in various solvents, using 20 mol. % *o-***2** catalyst loading (Table 3).

**Table 3:** The optimization of the conjugate addition between pyrazolone 4d and β-nitrostyrene 5a


Entry	Cat. loading^a^	Solvent	Temperature (°C)	Time (h)	Dr^b^	Yield of 6a (%)^c^
1	20	Toluene	25	20	7:1	65
2	20	DMSO	25	20	6:1	68
3	20	DCM	25	10	7:1	75
4	10	DCM	25	10	8:1	85
5	5	DCM	25	10	8:1	95

^a^The catalyst *o*-**2** (mol%) was treated with nitrostyrene **5a** (2 equiv.) followed by the solvent (1 mL) and pyrazolone derivative **4d** (1 equiv.) addition.

^b^Ratio determined by ^1^H (CD_3_CN, 500 MHz) NMR of crude reaction mixture.

^c^Isolated yield.

To our delight, the model reaction carried out in toluene afforded the corresponding Michael adduct **6a** after 20 h in a reasonable yield of 65%, isolated as an inseparable mixture of two diastereoisomers (*Dr*. 7:1) (Table 3, entry 1). Switching the solvent to DMSO resulted in a comparable reaction time and yield (68%), although a slight decrease in diastereoselectivity (*Dr*. 6:1) was observed. When DCM was employed as the solvent, full conversion was achieved within 10 h, and the desired product **6a** was isolated in a high yield of 75%, again as an inseparable diastereoisomeric mixture (*Dr*. 7:1) (Table 3, entry 3). Further optimization in the catalyst loading revealed that the reaction proceeded efficiently even with 5 mol. % *o*-**2** catalyst loading, reaching full conversion after 40 h. Notably, under these conditions, an excellent yield of 95% and improved diastereoselectivity (*Dr*. 8:1) of the corresponding adduct **6a** were obtained (Table 3, entry 5).

**Fig. 6:**
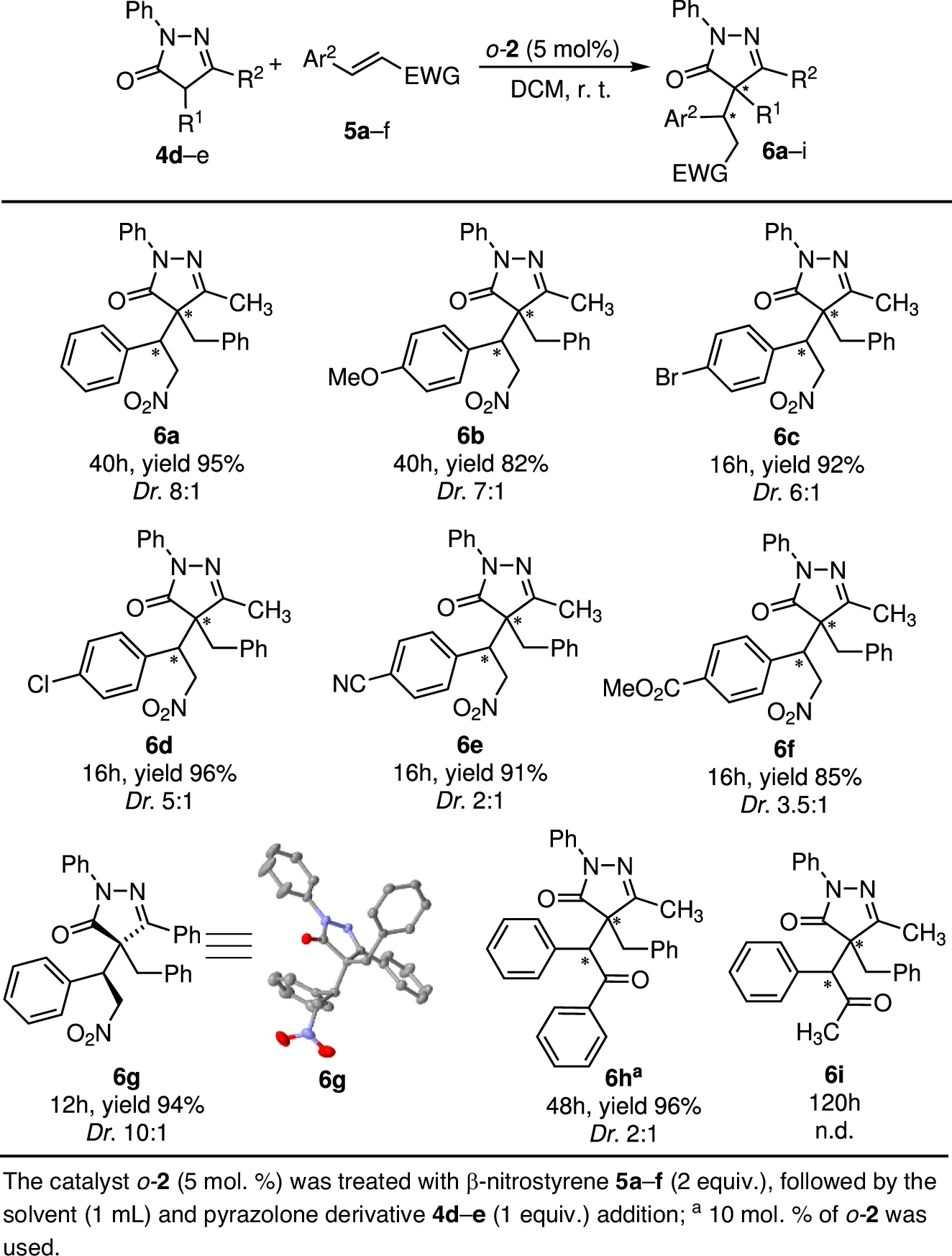
The substrate scope of the conjugate addition reaction between the pyrazolones **4d**–**e** and the olefins **5a**–**f**.

With these optimal reaction conditions in hand, we proceeded to evaluate the substrate scope of the reaction (Fig. 6). First, the substitution on the aromatic ring of β-nitrostyrene was examined with respect to diastereoselectivity and the yield of the corresponding adducts **6**. As summarized in Fig. 6, the reaction tolerated not only the model unsubstituted β-nitrostyrene **4a** but also the electron-rich *p*-methoxy-β-nitrostryrene **5b**, which afforded the Michael adduct **6b** in 82% yield after 40 h. Nearly quantitative yields were obtained with the *p*-Br- and *p*-Cl-substituted β-nitrostyrenes **5c** and **5d**, yielding as much as 92% for the bromo-substituted product **6c** and 96% for the chloro-substituted product **6d**. In a superbase-driven transformations, the obvious drawback is often an intolerance of catalyst to various functional groups of substrates. To proof a compatibility of *o*-**2** catalyst with such functional groups as nitriles or esters, the reaction of **4d** with **5e** and **5f**, respectively, were carried out at the same conditions. In the cases of *o*-**2** catalyzed reactions, only a slight decrease in activity and diastereoselectivity, when compared to other substrates, to **6e** and **6f** was observed, while the reactions driven by superbases or KO^t^Bu gave mixtures of various products. The sterically more demanding pyrazolone, 4-benzyl-2,5-diphenylpyrazol-3-one (**4e**), reacted with the model β-nitrostyrene **5a** to give the product **6g** with enhanced diastereocontrol (*Dr*. 10:1) and a high yield of 94% after 12 h. The structure and the relative configuration of **6g** were confirmed by X-ray diffraction analysis. To expand the scope further, model pyrazolone derivative **4d** was reacted with *trans*-chalcone as a Michael acceptor. Unfortunately, the reaction carried out with 5 mol. % of the catalyst *o-***2** was sluggish, and full conversion required 10 mol. % of *o-***2**. Under these conditions, the corresponding product **6h** was obtained after 48 h as an inseparable mixture of two diastereoisomers (*Dr*. 2:1), but in an almost quantitative yield of 96%, which corroborates our observations from the model Michael addition.

In spite of the low diastereocontrol observed in this reaction, we recognized that a dibenzylideneacetone derivative—as an extended chalcone moiety—could act as a double Michael acceptor in a diastereoselective conjugate cascade reaction with unsubstituted pyrazolones under carborane *o-***2** catalysis, leading to the formation of spiropyrazolones.

The strategy of employing double conjugate addition to divinyl ketones under chiral primary amine catalysis has been previously applied to a range of heterocycles, including oxindols^58,59^, pyrazolones^57^, benzofuranones^60,61^, as well as benzothiophenones and rhodanines^56^. However, these protocols typically required relatively high catalyst loading (20 mol. %) and acidic additives to generate the key reactive intermediate, a chiral iminium.

To validate this concept, we investigated the cascade reaction of 2-phenyl-5-methyl-pyrazol-3-one (**4a**) with unsubstituted dibenzylideneacetone **7a** in DCM as a solvent using 10 mol. % of the catalyst *o-***2** at room temperature. To our delight, full conversion was achieved after 5 h, and the corresponding spirocycle **8a** was isolated in an acceptable yield of 50% as a single diastereoisomer. Solvent screening revealed high tolerance of the reaction toward non-polar, polar aprotic, chlorinated and etheric solvents, consistently providing excellent diastereoselectivity (*Dr*. >20:1) in all cases (Table 4, entries 2–6). Further optimization of catalyst loading demonstrated that *o-***2** retained excellent catalytic activity even at 1 mol. % loading in DCM at 45 °C (Table 4, entry 9). Under these conditions, full conversion was reached after 6 h, and the corresponding spiropyrazolone **8a** was isolated in a high yield of 88% as a single diastereoisomer.

**Table 4:** The optimization of the reaction condition between pyrazolone **4a** and dibenzylideneacetone **7a**


Entry	Cat. loading (mol. %)	Solvent	Temperature (°C)	Time (h)	Drᵃ (%)	Yield of 8a (%)ᵇ
1	10	DCM	25	5	>20:1	50
2	10	MeCN	25	16	>20:1	48
3	10	THF	25	16	>20:1	43
4	10	CHCl₃	25	8	>20:1	65
5	10	PhMe	25	16	>20:1	48
6	10	DMSO	25	10	>20:1	75
7	5	CHCl₃	25	12	>20:1	40
8	5	DCM	25	12	>20:1	41
9	1	DCM	45	6	>20:1	88
10	1	DMSO	45	3	>20:1	25

The cat. *o*-**2** (mol. %) was treated with **7a** (1.5 equiv.) followed by the solvent (1 mL) and **4a** (1 equiv.).

^a^Determined by the ^1^H (CD_3_CN, 500 MHz) NMR of the crude reaction mixture.

^b^Isolated yield.

**Fig. 7:**
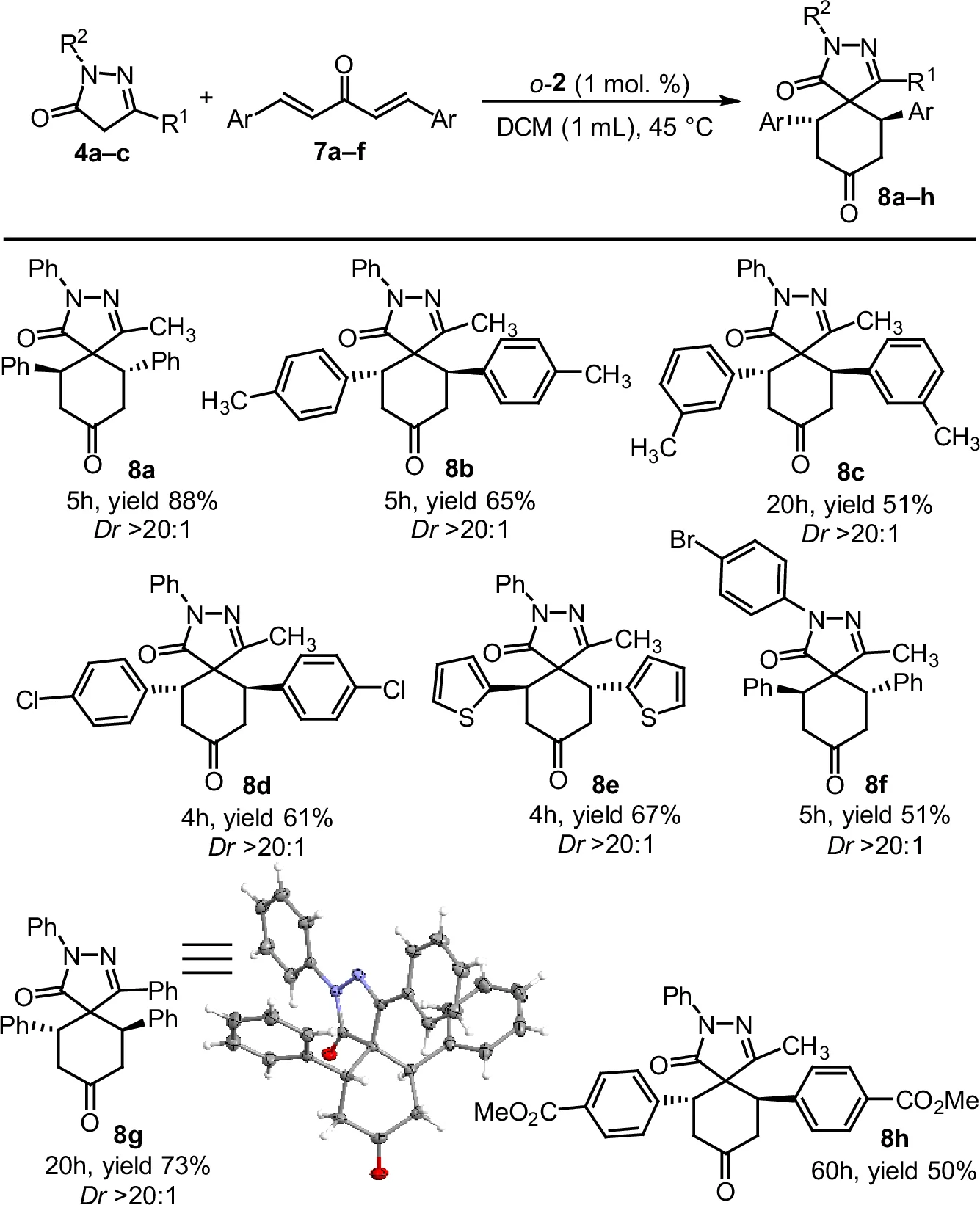
The substrate scope of the spirocyclization between the pyrazolones **4a**–**c** and the dibenzylideneacetones **7a**–**f**.

Next, the scope of the spirocyclization reaction was explored using various substituted dibenzylideneacetone derivatives (Fig. 7). Initially, derivatives bearing methyl substituents in the *para-* and *meta*-positions of the aromatic ring were tested with model pyrazolone **4a** in a cascade reaction. Both corresponding spirocycles **8b** and **8c** were isolated as single diastereoisomers in yields of 65% for the *para*-methyl-substituted product **8b** and 51% for the *meta*-methyl-substituted product **8c**. Furthermore, dibenzylideneacetone **7d**, bearing a slightly electron-withdrawing chlorine substituent in the *para*-position, was successfully employed, affording spiropyrazolone **8d** as a single diastereoisomer in a good yield of 61%. The heteroaryl derivative 1,5-di(thiophen-2-yl)penta-1,4-dien-3-one (**7e**) also underwent spirocyclization, yielding spiropyrazolone derivative **8e** in 67% yield. Additionally, the sterically more demanding, fully aromatic substituted 2-phenyl-5-phenyl-pyrazol-3-one (**4b**) was tested with model dibenzylideneacetone **7a**, achieving full conversion after 20 h and affording the corresponding product **8g** in a high yield of 73%. The structure of the compound and the relative configuration of both phenyl rings on the cyclohexanone moiety were confirmed by single-crystal X-ray diffraction, revealing the product as a *trans*-isomer. Similarly as for conjugate addition, the tolerance of *o-***2** catalytic system towards sensitive functional groups is demonstrated on spirocyclization leading to **8h**, where no unwanted byproducts were identified.

To conclude, mentioned findings highlight the unique reactivity profile of the carborane-based Lewis base *o-***2**. While its cationic counterpart *o-***2a** exhibits minimal activity, *o-***2** engages in diverse reactions with C-acids, solvents and water. Depending on the pK_A_ of the substrate, the reaction equilibrium shifts either toward the protonated form for stronger acids or remains with the reactants for more basic species. This behavior, along with its calculated proton affinity, supports the classification of *o-***2** as a superbase with low nucleophilicity. Under the applied conditions, certain substrates undergo co-condensation via C–C-bond formation. When used catalytically, *o-***2** demonstrates exceptional activity in Lewis-base-mediated transformations such as aldol condensation, Thorpe reaction, Michael addition, followed by the Robinson annulation and related processes—universal reactivity that is rare not only among carboranes but also for most compounds reported in the literature. The catalytic performance correlates with the acidity (pK_A_) of the substrates, yielding distinct conversions and products. Although, similar reactions can be performed also by moisture-stable conjugates of superbases with carboxylic acids, the reactivation of the superbase by epoxides, prolonged warming and water intolerance is reported^62^. Moreover, conjugate-addition reactions between 4-substituted pyrazolone derivatives and electron-deficient olefins (e.g. β-nitrostyrene and *trans*-chalcone derivatives) catalyzed by *o-***2** furnished the products **6a**–**h** with excellent yields (82–96%) and moderate to high diastereoselectivity (*Dr*. 2:1 to 10:1), featuring two newly formed quaternary and tertiary carbon centers. Furthermore, *o-***2** proved to be an efficient catalytic platform for highly diastereoselective spirocyclization between unsubstituted pyrazolones and dibenzylideneacetones with only 1 mol. % of catalyst loading, delivering the spiropyrazolones **8a**–**h** in good to high yields (51–88%) as single diastereoisomers. The described processes are not only selective; they also demonstrate tolerance for superbase-sensitive functional groups like nitriles and esters. These results demonstrate that umpolung of polyhedral (car)boranes via increasing the electron density at the boron atoms and even inside the cluster—such as through carbene adduct formation—and their application in Lewis-base catalysis or anionic polymerization may offer a powerful and versatile strategy for future developments in synthetic chemistry.

## Methods

### Synthesis

The synthesis of *o*-**2b**–*o*-**2w** was carried out under an argon atmosphere using standard Schlenk tube technique. Solvents were dried using a Pure Solv–Innovative Technology equipment under argon gas atmosphere. Starting compound 6,9-IPr_*2*_-5,10-C_2_B_8_H_10_ (*o*-**2**) was prepared according to the published procedure^42^ from toluene solution of *closo*-*o*-C_2_B_8_H_10_ (1.030 g, 8.54 mmol) (10 mL), which was added dropwise to a stirred solution of IPr (7.302 g, 18.8 mmol) in toluene (100 mL) at room temperature. The yellow reaction mixture was stirred for one hour and then the volatiles were removed in vacuo. The crude product was washed with hexane (2 × 30 mL) to remove the excess of IPr. The yellowish powder of *o***-2** was dried in vacuo. Yield 7.286 mg, 95%. The spectral data agreed with the previously published^42^. Starting pyrazolones **4a**–**4b** are commercially available. Detailed description of synthesis and catalysis (including generalized Procedures A-I) is given in the Supplementary Information file.

### NMR spectroscopy

^1^H (400.1, 500.1 and 600.1 MHz)^11^,B (160.4 MHz) and ^13^C (101.6, 125.8 and 150.9 MHz) NMR spectra were recorded on Bruker Avance 500 and 600 MHz spectrometers or Bruker Ultrashield 400 MHz, using 5 mm tuneable broad-band probes. Appropriate chemical shifts in ^1^H and ^13^C NMR spectra were related to the residual signals of the solvents (CDCl_3_: *δ*(^1^H) = 7.24 ppm and *δ*(^13^C) = 77.23 ppm; acetone-d_6_: *δ*(^1^H) = 2.05 ppm and *δ*(^13^C) = 29.92 ppm; CD_2_Cl_2_: *δ*(^1^H) = 5.33 ppm and *δ*(^13^C) = 54.24 ppm; THF-d_8_: *δ*(^1^H) = 1.73 and 3.58 ppm and *δ*(^13^C) = 25.37 and 67.57 ppm)^11^.B chemical shifts were related to external standard BF_3_•OEt_2_ (*δ*(^11^B) = 0.0 ppm).

### sc-XRD

Full-sets of diffraction data for *o*-**2b,** *o*-**2c,** *o*-**2f’,** *o*-**2g,** *o*-**2i,** *enol*-**1a”,** **6g** and **8g** were collected at 150(2)K with a Bruker D8-Venture diffractometer equipped with Mo (Mo/K_α_ radiation; λ = 0.71073 Å) microfocus X-ray (IµS) source, Photon CMOS detector and Oxford Cryosystems cooling device was used for data collection. The frames were integrated with the Bruker SAINT software package using a narrow-frame algorithm. Data were corrected for absorption effects using the Multi-Scan method (SADABS). Obtained data were treated and structures solved and refined by XT-version 2014/5 and SHELXL-2014/7 software implemented in APEX3 v2016.5-0 or APEX4 v2021.10-0 (Bruker AXS) system^63^.

Hydrogen atoms were mostly localized on a difference Fourier map, however, to ensure uniformity of treatment of crystal, all ‘organic’ hydrogens were recalculated into idealized positions (riding model) and assigned temperature factors H_iso_(H) = 1.2 U_eq_ (pivot atom) or of 1.5U_eq_ (methyl). Hydrogen atoms in methyl, methine, methylene C–H moieties and aromatic rings were placed with C–H distances of 0.96, 0.97, 0.98, 1.00 and 0.93 Å, while hydrogens in N–H, *O–*H or B–H groups were placed according to the maxima on the Fourier maps and refined freely. Some of the B–H moieties from the carborane units were placed with distances of 1.1 and 1.25 Å for terminal and bridging hydrogen atoms, respectively.

## Supplementary information

Supplementary Information

Transparent Peer Review file

## Data Availability

The crystallographic data for structural analysis have been deposited with the Cambridge Crystallographic Data Centre, CCDC nos. 2404722-2404729. Copies of this information may be obtained free of charge from The Director, CCDC, 12 Union Road, Cambridge CB2 1EY, UK (fax: +44-1223-336033; e-mail: deposit@ccdc.cam.ac.uk or www: http://www.ccdc.cam.ac.uk). The NMR data have been deposited at Figshare.com and are available at https://doi.org/10.6084/m9.figshare.30163174.
