# Supplementary material for: Uncover the strength of polyhedral dicarbaboranes as the next generation of superbases in organocatalysis
Source: Nat Commun. 2026 Aug 26;17:10198. doi: 10.1038/s41467-026-77070-6 (PMC13612482; doi:10.1038/s41467-026-77070-6)
Supplement: Supplementary file 1 [file 41467_2026_77070_MOESM1_ESM.pdf]

Suppplementary information for

Uncover the Strength of Polyhedral Dicarbaboranes as The Next Generation of Superbases in Organocatalysis

Jan Vrána,[1], Zuzana Kapustová,[1] Martin Kamlar,[2] Peter Šramel,[1] Petr Vosáhlo,[1] Zdeňka Růžičková,[1] Josef Holub,[3] Jan Veselý,[2] Aleš Růžička[1*]*

[1]Department of General and Inorganic Chemistry, Faculty of Chemical Technology, University of Pardubice, Studentská 573, 532 10 Pardubice, Czech Republic

[2]Department of Organic Chemistry, Faculty of Science, Charles University, Hlavova 8, 128 00 Prague 2, Czech Republic

[3]Institute of Inorganic Chemistry, Czech Academy of Sciences, Řež 1001, 250 68 Husinec-Řež, Czech Republic

*Jan Vrána – jan.vrana@upce.cz, *Jan Veselý – jan.vesely@natur.cuni.cz, *Aleš Růžička – ales.ruzicka@upce.cz

Table of contents

Supplementary methods........ S3

Synthesis and characterisation of starting material........ S4

Synthesis and characterisation of *o*-**2b**–*o*-**2w**........ S6

General procedure of testing the basicity of *o*-**2**........ S60

Aldol condensation catalysed by carborane *o*-**2**........ S60

Michael reaction catalysed by carborane *o*-**2**........ S66

Catalytic conjugated addition reaction of pyrazolones with nitrostyrene derivatives........ S76

Catalytic spirocyclization reaction........ S91

References........ S106

Supplementary methods

NMR spectroscopy

^{1}H, ^{11}B and ^{13}C NMR spectra were recorded on Bruker Avance 500 and 600 MHz spectrometers or Bruker Ultrashield 400 MHz, using 5 mm tuneable broad-band probe. Appropriate chemical shifts in ^{1}H (400.1, 500.1 and 600.1 MHz) and ^{13}C NMR (101.6, 125.8 and 150.9 MHz) spectra were related to the residual signals of the solvents ($CDCl_3$: $\delta(^{1}H) = 7.24$ ppm and $\delta(^{13}C) = 77.23$ ppm; acetone-d_6: $\delta(^{1}H) = 2.05$ ppm and $\delta(^{13}C) = 29.92$ ppm; CD_2Cl_2: $\delta(^{1}H) = 5.33$ ppm and $\delta(^{13}C) = 54.24$ ppm; THF-d_8: $\delta(^{1}H) = 1.73$ and 3.58 ppm and $\delta(^{13}C) = 25.37$ and 67.57 ppm). ^{11}B (160.4 MHz) chemical shifts were related to external standard BF_3•OEt_2 ($\delta(^{11}B) = 0.0$ ppm). Numbering of the carborane is as follows:

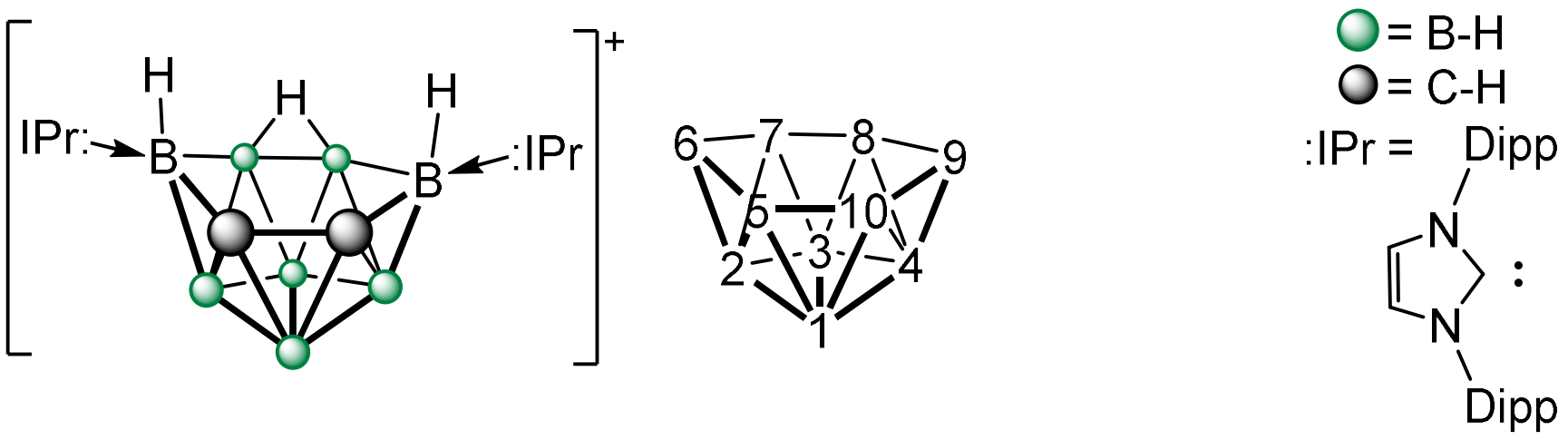

Supplementary Figure S1. Numbering of the cation $[6,9\text{-}IPr_2\text{-}5,10\text{-}C_2B_8H_{11}]^+$.

sc-XRD

Full-sets of diffraction data for *o*-**2b**, *o*-**2c**, *o*-**2f'**, *o*-**2g**, *o*-**2i**, *enol*-**1a''**, **6g** and **8g** were collected at 150(2)K with a Bruker D8-Venture diffractometer equipped with Mo (Mo/K_α radiation; $\lambda = 0.71073$ Å) microfocus X-ray (IμS) source, Photon CMOS detector and Oxford Cryosystems cooling device was used for data collection.

The frames were integrated with the Bruker SAINT software package using a narrow-frame algorithm. Data were corrected for absorption effects using the Multi-Scan method (SADABS). Obtained data were treated, structures solved and refined by XT-version 2014/5 and SHELXL-2014/7 software implemented in APEX3 v2016.5-0 or APEX4 v2021.10-0 (Bruker AXS) system.[S1]

Hydrogen atoms were mostly localized on a difference Fourier map, however, to ensure uniformity of treatment of crystal, all 'organic' hydrogens were recalculated into idealized positions (riding model) and assigned temperature factors $H_{iso}(H) = 1.2\ U_{eq}$ (pivot atom) or of $1.5U_{eq}$ (methyl). Hydrogen atoms in methyl, methine, methylene C-H moieties and aromatic rings were placed with C-H distances of 0.96, 0.97, 0.98, 1.00 and 0.93 Å, while hydrogens in N-H, *O*-H or B-H groups were placed according to the maxima on the Fourier maps and refined freely. Some of the B-H moieties from the carborane units were placed with distances of 1.1 and 1.25 Å for terminal and bridging hydrogen atoms, respectively.

$R_{int} = \sum|F_o^2 - F_{o,mean}^2|/\sum F_o^2$, $S = [\sum(w(F_o^2 - F_c^2)^2)/(N_{diffrs} - N_{params})]^{½}$ for all data, $R(F) = \sum||F_o| - |F_c||/\sum|F_o|$ for observed data, $wR(F^2) = [\sum(w(F_o^2 - F_c^2)^2)/(\sum w(F_o^2)^2)]^{½}$ for all data.

Crystallographic data for structural analyses have been deposited with the Cambridge Crystallographic Data Centre, CCDC nos. 2404722 (*enol*-**1a''**), 2404723 (**6g**), 2404724 (*o*-**2b**), 2404725 (*o*-**2f'**), 2404726 (*o*-**2c**), 2404727 (*o*-**2i**), 2404728 (*o*-**2g**) and 2404729 (**8g**). Copies of this information may be obtained free of charge from The Director, CCDC, 12 Union Road, Cambridge CB2 1EY, UK (fax: +44-1223-336033; e-mail: deposit@ccdc.cam.ac.uk or www: http://www.ccdc.cam.ac.uk).

In some of the structures have disordered anions or the carborane cage. This has been solved as a static disorder.

Synthesis

The synthesis of *o*-**2b**–*o*-**2w** was carried out under an argon atmosphere using standard Schlenk tube technique. Solvents were dried using a Pure Solv–Innovative Technology equipment under argon gas atmosphere. Starting compound $6,9\text{-}IPr_2\text{-}5,10\text{-}C_2B_8H_{10}$ (*o*-**2**) was prepared according to the published procedure,[42] where the solution of *closo*-*o*-$C_2B_8H_{10}$ (1.030 g, 8.54 mmol) in toluene (10 mL) was added dropwise to a stirred solution of IPr (7.302 g, 18.8 mmol) in toluene (100 mL) at room temperature. The yellow reaction mixture was stirred for one hour and then the volatiles were removed in vacuo. The crude product was washed with hexane (2x 30 mL) to remove the excess IPr. The slightly yellow powder of *o*-**2** was dried in vacuo. Yield 7.286 g, 95 %. The spectral data agreed with the previously published. Starting pyrazolones **4a**–**4b** are commercially available.

Synthesis and characterization of starting material:

Nitrostyrenes preparation

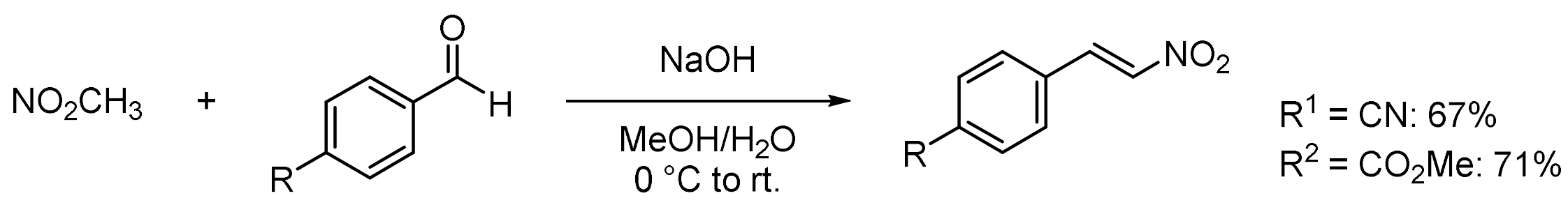

4-(2-Nitrovinyl)benzonitrile (5e)

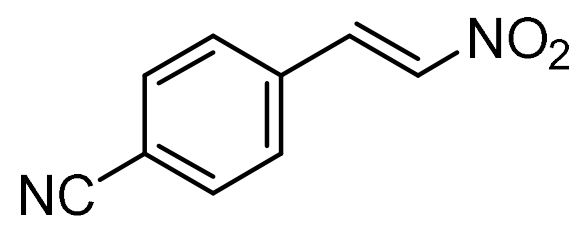

Compound prepared according to the published procedure,[S2] a solution of NaOH (210 mg, 5.25 mmol, 1.05 equiv.) in ice-cold water (10 mL) was dropwise added to a solution of aldehyde (656 mg, 5 mmol, 1 equiv.) and nitromethane (0.27 mL, 5 mmol, 1 equiv.) in MeOH (10 mL). The reaction was kept bellow 10 °C during the dripping and full conversion was observed by TLC monitoring after stirring at the same temperature for one hour. Reaction mixture was then transferred to separatory funnel and was slowly dripped into a solution of HCl (25 mL, 6M solution). Formed precipitate of the product was collected by filtration and washed with cold water, EtOH and dried in vacuo yielding pale yellow solid (580 mg, 67%).

^{1}H NMR (25 °C, 400 MHz, $CDCl_3$) δ = 7.99 (d, J(^{1}H-^{1}H) = 13.8 Hz, 1H), 7.76 (d, J(^{1}H-^{1}H) = 8.5 Hz, 2H), 7.66 (d, J(^{1}H-^{1}H) = 8.4 Hz, 2H), 7.61 (d, J(^{1}H-^{1}H) = 13.7 Hz, 1H) ppm; **^{13}C NMR** (25 °C, 101 MHz, $CDCl_3$) δ = 139.6, 136.7, 134.5, 133.2, 129.5, 117.9, 115.4 ppm.

Methyl-4-(2-nitrovinyl)benzoate (5f)

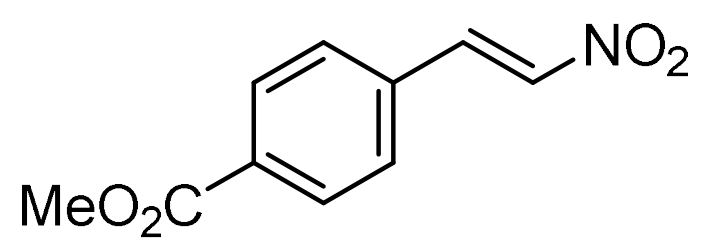

Compound prepared according to the published procedure,[S3] to a solution of aldehyde (821 mg, 5 mmol, 1 equiv.) and nitromethane (0.67 mL, 12.5 mmol, 2.5 equiv.) in MeOH (10 mL), an aqueous solution of NaOH (500 mg in 1.3 mL of water, 2.5 equiv.) was added dropwise at 0 °C. Full conversion was observed by TLC monitoring after stirring at the same temperature for one hour. Crushed ice was then added and reaction mixture was then poured onto a solution of HCl (10.3 mL, 5M solution) at 0 °C. The formed precipitate was collected by filtration, washed with cold water and purified by crystallization yielding the corresponding product as pale-yellow solid (740 mg, 71%). Spectral data correspond to the published data.

^{1}H NMR (25 °C, 400 MHz, $CDCl_3$) δ = 8.15 – 8.08 (m, 2H), 8.01 (d, J(^{1}H-^{1}H) = 13.8 Hz, 1H), 7.65 – 7.60 (m, 3H), 3.95 (s, 3H) ppm; **^{13}C NMR** (25 °C, 101 MHz, $CDCl_3$) δ = 166.1, 138.8, 137.7, 134.3, 133.2, 130.6, 129.1, 52.6 ppm.

Pyrazolones preparation

Pyrazolones **4c**–**4e** were prepared according to the modified published procedure (**Procedure A**).[S4] The corresponding oxoester derivative (10 mmol 1 equiv.) and phenylhydrazine derivative (10 mmol, 1 equiv.) were dissolved in ethanol (15 mL) and reaction mixture was heated to reflux overnight. Corresponding reaction mixture was then evaporated and solid residue were purified either by column chromatography (SiO_2, eluent: Hex/EtOAc) or crystallization from diethyl ether.

2-(4-bromophenyl)-5-methyl-2,4-dihydr*o*-3*H*-pyrazol-3-one (4c)

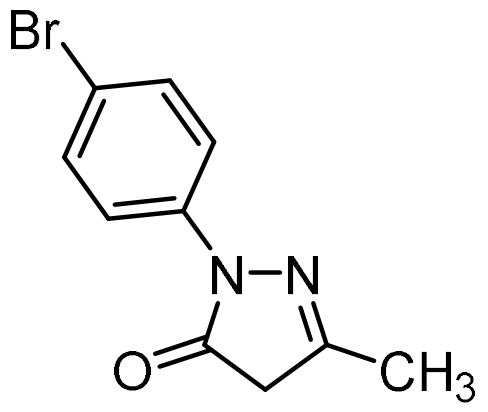

Compound was prepared by **Procedure A**. Compound **4c** was isolated upon crystallization from diethyl ether as off-white solid in 65% yield (1.65 g). **^{1}H NMR** (25 °C, $CDCl_3$, 600 MHz): δ = 7.77 (d, *J*(^{1}H-^{1}H) = 9.0 Hz, 2H), 7.47 (d, *J*(^{1}H-^{1}H) = 9.0 Hz, 2H), 3.40 (s, 2H), 2.17 (s, 3H) ppm. **^{13}C NMR** (25 °C, $CDCl_3$, 150.9 MHz): δ = 170.6, 156.7, 137.3, 131.9 (2C), 120.2 (2C), 117.8, 43.2, 17.1 ppm; LRMS (ESI) m/z: calcd for $C_{10}H_9BrN_2ONa$ $[M+Na]^+$ 275.0; found 275.1.

4-Benzyl-3-methyl-1-phenyl-2,4-dihydr*o*-3*H*-pyrazol-3-one (4d)

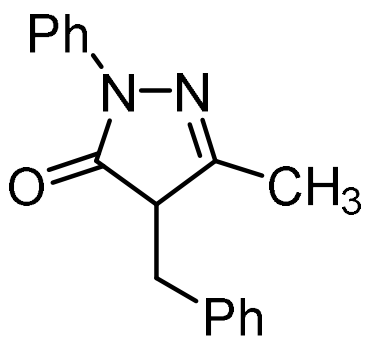

Compound was prepared by **Procedure A**. Compound **4d** was isolated by FLC (SiO_2, eluent: Hex/EtOAc = 6/1) as pale-yellow solid in 51% yield (1.35 g). **^{1}H NMR** (25 °C, DMS*O*-d_6, 600 MHz): δ = 7.78–7.71 (m, 2H), 7.50–7.38 (m, 2H), 7.33–7.13 (m, 3H), 3.62 (s, 2H), 2.06 (s, 3H) ppm. **^{13}C NMR** (25 °C, DMS*O*-d_6, 150.9 MHz): δ = 147.7, 141.0, 138.2, 128.8 (2C), 128.2 (2C), 128.0 (2C), 125.7, 124.5, 119.2 (3C), 27.4, 11.8 ppm; HRMS (ESI) m/z: calcd for $C_{17}H_{17}N_2O$ $[M+H]^+$ 265.1335; found 265.1337.

4-Benzyl-1,3-diphenyl-2,4-dihydr*o*-3*H*-pyrazol-3-one (4e)

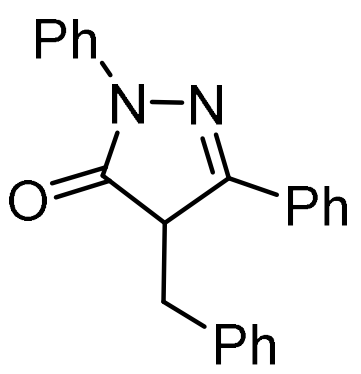

Compound was prepared by **Procedure A**. Compound **4e** was isolated by FLC (SiO_2, eluent: Hex/EtOAc = 7/1) as off-white solid in 68% yield (2.22 g). Off-white solid. Yield 68%. **^{1}H NMR** (25 °C, DMS*O*-d_6, 600 MHz): δ = 7.86 (d, *J*(^{1}H-^{1}H) = 8.0 Hz, 2H), 7.58 (d, *J*(^{1}H-^{1}H) = 7.5 Hz, 2H), 7.49 (t, *J*(^{1}H-^{1}H) = 7.9 Hz, 2H), 7.39–7.33 (m, 3H), 7.29–7.25 (m, 3H), 7.21–7.13 (m, 3H), 3.94 (s, 2H) ppm. **^{13}C NMR** (25 °C, DMS*O*-d_6, 150.9 MHz): δ = 149.1, 140.9, 138.6, 128.9 (2C), 128.5 (3C), 128.3 (2C), 127.8 (2C), 127.1 (3C), 125.8, 125.5 (2C), 120.9 (2C), 27.7 (2C) ppm; LRMS (ESI) m/z: calcd for $C_{22}H_{18}N_2ONa$ $[M+Na]^+$ 349.1; found 349.1.

Dienones preparation

The dienones **7a**–**7e** were prepared according to the modified published procedure (**Procedure B**).[S5] The corresponding aldehyde (18.8 mmol, 2 equiv.) and acetone (0.7 mL, 9.4 mmol, 1 equiv.) were dissolved in ethanol (20 mL) and a solution of KOH (527 mg, 9.4 mmol, 1 equiv.) in H_2O (10 mL) was added dropwise. The resulting mixture was allowed to stir overnight at room temperature and then diluted with H_2O (50 mL). The products were extracted with DCM (2 × 50 mL) and the combined organic phase was dried over $MgSO_4$ and concentrated in vacuo. The crude product was purified using column chromatography (SiO_2, eluent: Hex/EtOAc).

(1*E*,4*E*)-1,5-diphenylpenta-1,4-dien-3-one[S7] (7a)

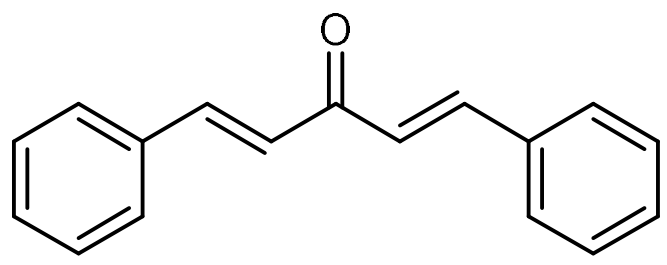

Compound was prepared by **Procedure B**. Compound **7a** was isolated by FLC (SiO_2, eluent: Hex/EtOAc = 6/1) as yellow solid in 60% yield (1.32 g). **^{1}H NMR** (25 °C, $CDCl_3$, 400 MHz): δ = 7.75 (d, $J(^1H-^1H)$ = 16.0 Hz, 2H), 7.68–7.58 (m, 4H), 7.49–7.38 (m, 6H), 7.09 (d, $J(^1H-^1H)$ = 16.0 Hz, 2H) ppm. **^{13}C NMR** (25 °C, $CDCl_3$, 100.61 MHz): δ 189.1, 143.5 (2C), 134.9 (2C), 130.6 (2C), 129.1 (4C), 128.5 (4C), 125.6 (2C) ppm. LRMS (ESI) m/z: calcd for $C_{17}H_{14}ONa$ $[M+Na]^+$ 257.1; found 257.1.

(1*E*,4*E*)-1,5-di-*p*-tolylpenta-1,4-dien-3-one[S5] (7b)

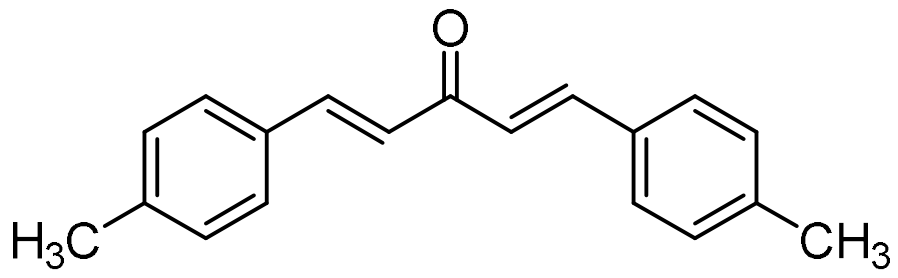

Compound was prepared by **Procedure B**. Compound **7b** was isolated after FLC (SiO_2, eluent: Hex/EtOAc = 6/1) as yellow solid in 81% yield (2.00 g).**^{1}H NMR** (25 °C, $CDCl_3$, 400 MHz): δ = 7.72 (d, $J(^1H-^1H)$ = 15.9 Hz, 2H), 7.59–7.48 (d, $J(^1H-^1H)$ = 8.0 Hz, 4H), 7.22 (d, $J(^1H-^1H)$ = 7.9 Hz, 4H), 7.04 (d, $J(^1H-^1H)$ = 15.9 Hz, 2H), 2.39 (s, 6H) ppm. **^{13}C NMR** (25 °C, $CDCl_3$, 100.61 MHz): δ = 189.2, 143.3 (2C), 141.1 (2C), 132.3 (2C), 129.8 (4C), 128.5 (4C), 124.8, 124.7, 21.7 (6C) ppm. LRMS (ESI) m/z: calcd for $C_{19}H_{18}ONa$ $[M+Na]^+$ 285.2; found 285.2.

(1*E*,4*E*)-1,5-di-*m*-tolylpenta-1,4-dien-3-one[S5] (7c)

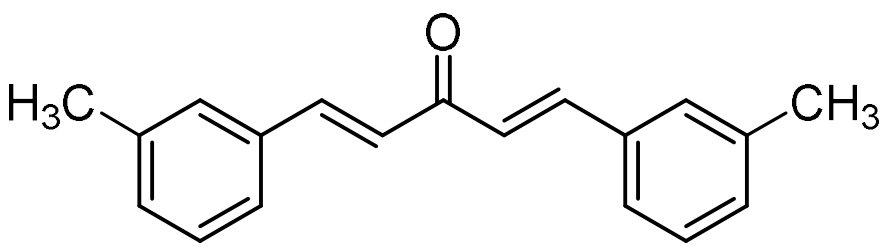

Compound was prepared by **Procedure B**. Compound **7c** was isolated after FLC (SiO_2, eluent: Hex/EtOAc = 8/1) as yellow solid in 75% yield (1.85 g).**^{1}H NMR** (25 °C, $CDCl_3$, 400 MHz): δ = 7.72 (d, $J(^1H-^1H)$ = 15.9 Hz, 2H), 7.59–7.48 (d, $J(^1H-^1H)$ = 8.0 Hz, 4H), 7.22 (d, $J(^1H-^1H)$ = 7.9 Hz, 4H), 7.04 (d, $J(^1H-^1H)$ = 15.9 Hz, 2H), 2.39 (s, 6H) ppm. **^{13}C NMR** (25 °C, $CDCl_3$, 100.61 MHz): δ = 189.2, 143.3 (2C), 141.1 (2C), 132.3 (2C), 129.8 (4C), 128.5 (4C), 124.8, 124.7, 21.7 (6C) ppm. LRMS (ESI) m/z: calcd for $C_{19}H_{18}ONa$ $[M+Na]^+$ 285.2; found 285.3.

(1*E*,4*E*)-1,5-bis(4-chlorophenyl)penta-1,4-dien-3-one[S5] (7d)

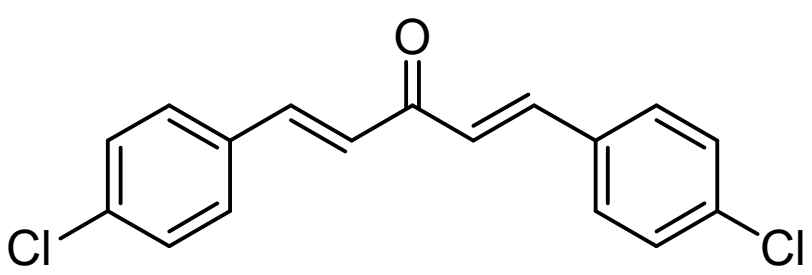

Compound was prepared by **Procedure B**. Compound **7d** was isolated after FLC (SiO_2, eluent: Hex/EtOAc = 8/1) as pale-yellow solid in 68% yield (1.94 g). **^{1}H NMR** (25 °C, $CDCl_3$, 400 MHz): δ = 7.68 (d, $J(^1H-^1H)$ = 15.9 Hz, 2H), 7.54 (d, $J(^1H-^1H)$ = 8.6 Hz, 4H), 7.38 (d, $J(^1H-^1H)$ = 8.5 Hz, 4H), 7.02 (d, $J(^1H-^1H)$ = 15.9 Hz, 2H) ppm. **^{13}C NMR** (25 °C, $CDCl_3$, 100.61 MHz): δ = 188.5, 142.2 (2C), 136.6 (2C), 133.3 (2C), 129.7 (4C), 129.4 (4C), 125.8 (2C) ppm. LRMS (ESI) m/z: calcd for $C_{17}H_{12}ClONa$ $[M+Na]^+$ 325.0; found 325.0.

(1*E*,4*E*)-1,5-di(thien-2-yl)penta-1,4-dien-3-one[S5] (7e)

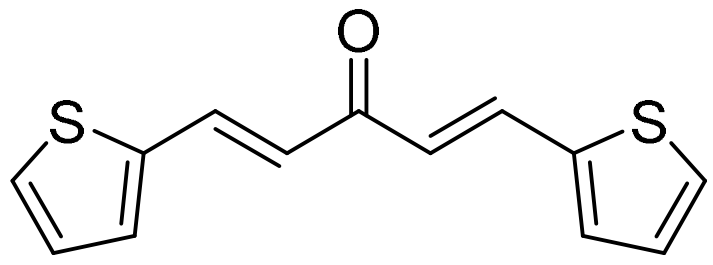

Compound was prepared by **Procedure B**. Compound **7e** was isolated after FLC (SiO_2, eluent: Hex/EtOAc = 6/1) as pale-yellow solid in 34% yield (780 mg). **^{1}H NMR** (25 °C, $CDCl_3$, 400 MHz): δ = 7.84 (d, J(^{1}H-^{1}H) = 15.5 Hz, 2H), 7.41 (dd, J(^{1}H-^{1}H) = 5.1 Hz, J'(^{1}H-^{1}H) = 1.0 Hz, 2H), 7.33 (d, J(^{1}H-^{1}H) = 3.9 Hz, 2H), 7.08 (dd, J(^{1}H-^{1}H) = 5.0 Hz, J'(^{1}H-^{1}H) = 3.6 Hz, 2H), 6.81 (d, J(^{1}H-^{1}H) = 15.5 Hz, 2H) ppm. **^{13}C NMR** (25 °C, $CDCl_3$, 100.61 MHz): δ = 187.9, 140.4, 135.8 (2C), 132.0 (2C), 129.0 (2C), 128.5 (2C), 124.5 (2C) ppm. LRMS (ESI) m/z: calcd for $C_{13}H_{10}OS_2Na$ $[M+Na]^+$ 269.0; found 269.0.

Dimethyl 4,4'-(3-oxopenta-1,4-diene-1,5-diyl)dibenzoate (7f)

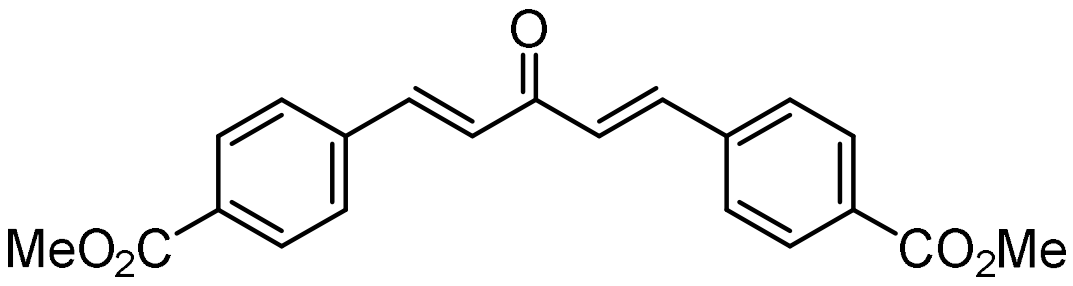

Compound was prepared by **Procedure B**. Compound **7f** , prepared according to the standard procedure in 5 mmol scale, was isolated after FLC (SiO_2, eluent: Hex/EtOAc = 6/1) as off-white solid material in 26% yield (455 mg). Spectral data correspond to the published data.[S6]

^{1}H NMR (25 °C, 400 MHz, $CDCl_3$) δ 8.08 (d, J(^{1}H-^{1}H) = 8.4 Hz, 4H), 7.76 (d, J(^{1}H-^{1}H) = 15.9 Hz, 2H), 7.68 (d, J(^{1}H-^{1}H) = 8.2 Hz, 4H), 7.15 (d, J(^{1}H-^{1}H) = 15.9 Hz, 2H), 3.94 (s, 6H) ppm; **^{13}C NMR** (25 °C, 101 MHz, $CDCl_3$)) δ = 188.4, 166.6 (2C), 142.4 (2C), 139.0 (2C), 131.8 (2C), 130.3 (4C), 128.4 (4C), 127.3 (2C), 52.5 (2C) ppm.

Attempted catalytic studies of *o*-2a

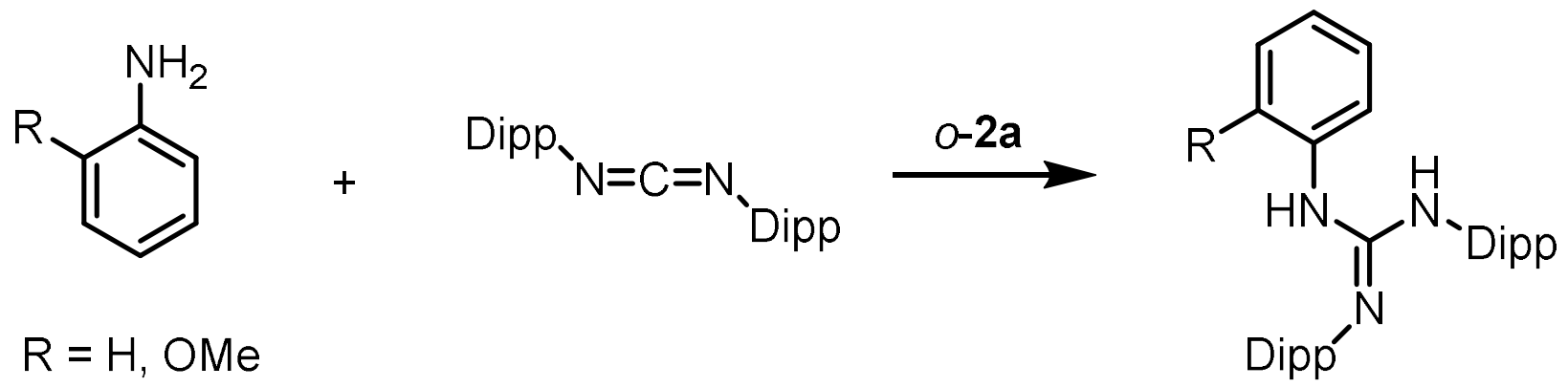

Entry	Cat. loading (mol%)	R	Solvent	Temperature (°C)	Time (d)	Conversion[a] (%)
**1**	1	OMe	CH_3CN	80	1	2
**2**	1	H	CH_3CN	80	1	2
**3**	1	H	Tol	100	3	3
**4**	10	H	Tol	100	1	5
**5**	10	H	Tol	100	3	12

[a] determined by ^{1}H NMR

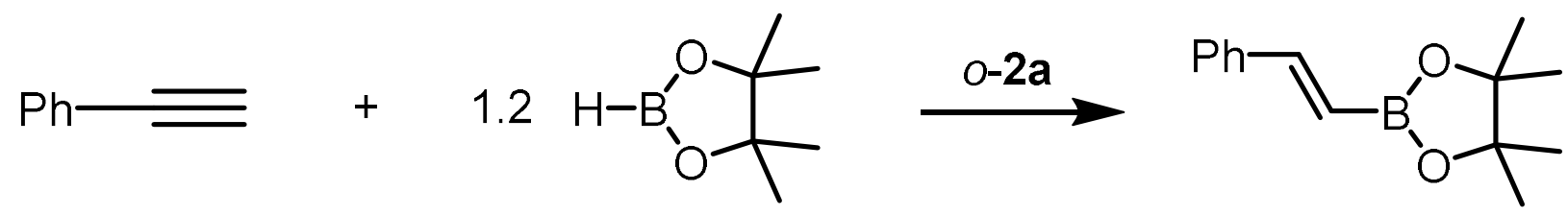

Entry	Cat. loading (mol%)	Solvent	Temperature (°C)	Time (h)	Conversion[a] (%)
**1**	1	neat	60	18	62
**2**	3	neat	RT	18	26
**3**	3	neat	60	18	66
**4**	3	Tol	60	18	0
**5**	3	THF	60	18	12
**6**	15	neat	60	18	70

[a] determined by GC-MS analysis

No conversion were observed in hydrosilylations of benzophenone.

Synthesis and characterisation of *o*-**2b**–*o*-**2w**

Synthesis of [6,9-IPr$_2$-5,10-$C_2B_8H_{11}$]$^+$[$F_3CC(O)CHC(O)CF_3$]$^-$ (*o*-**2b**)

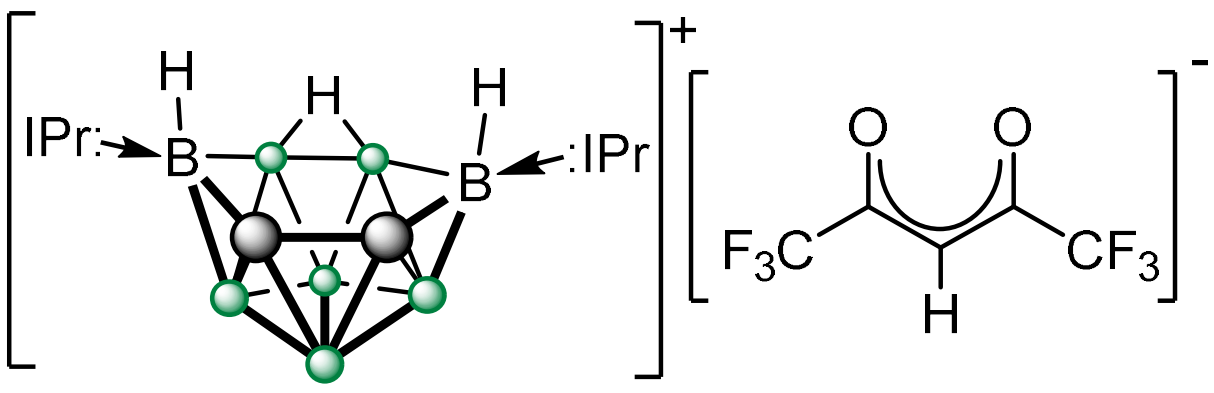

1,1,1,5,5,5-hexafluor*o*-pentane-2,4-dione (67.7 µL, 0.48 mmol) was added to a stirred solution of *o*-**2** (358 mg, 0.40 mmol) in toluene (15 mL) at room temperature. The resulting suspension was stirred for additional 15 minutes and filtrated. The solid was washed with toluene (10 mL) and the resulting colourless powder of *o*-**2b** was dried *in vacuo*. The crystals suitable for X-ray diffraction analysis were grown from a saturated solution of *o*-**2b** in acetonitrile at 4 °C. Yield 363 mg, 83%. **Dp** 215 °C. **^{1}H NMR** (25 °C, CD_2Cl_2, 500 MHz): δ = −4.24 (s broad, 1H, B*H*B), −0.85 (s, 1H, B3), −0.21 (s, 2H, B6,9), 0.58 (s, 2H, BC*H*), 1.06 (d, ^3J(^{1}H-^{1}H) = 6.8 Hz, 14H, CH(C*H*$_3$)$_2$ + B7,8), 1.10 (d, ^3J(^{1}H-^{1}H) = 6.8 Hz, 12H, CH(C*H*$_3$)$_2$), 1.13 (d, ^3J(^{1}H-^{1}H) = 6.8 Hz, 12H, CH(C*H*$_3$)$_2$), 1.17 (d, ^3J(^{1}H-^{1}H) = 6.8 Hz, 12H, CH(C*H*$_3$)$_2$), 1.52 (s, 1H, B1), 1.70 (s, 2H, B2,4), 2.18 (sept, ^3J(^{1}H-^{1}H) = 6.8 Hz, 4H, C*H*(CH$_3$)$_2$), 2.40 (sept, ^3J(^{1}H-^{1}H) = 6.8 Hz, 4H, C*H*(CH$_3$)$_2$), 5.37 (s, 1H, C(O)C*H*), 7.31 (d, ^3J(^{1}H-^{1}H) = 7.7 Hz, 4H, *m*-C$_6$*H*$_3$), 7.32 (d, ^3J(^{1}H-^{1}H) = 7.7 Hz, 4H, *m*-C$_6$*H*$_3$), 7.43 (s, 4H, NC*H*), 7.56 (t, ^3J(^{1}H-^{1}H) = 7.8 Hz, 4H, *p*-C$_6$*H*$_3$) ppm. ^{13}C{^{1}H} NMR (25 °C, CD_2Cl_2, 125.76 Hz): δ = 22.6 (s, CH(*C*H$_3$)$_2$), 23.4 (s, CH(*C*H$_3$)$_2$), 25.6 (s, CH(*C*H$_3$)$_2$), 25.8 (s, CH(*C*H$_3$)$_2$), 26.9 (s broad, B*C*H), 29.80 (s, *C*H(CH$_3$)$_2$), 29.81 (s, *C*H(CH$_3$)$_2$), 83.2 (s, C(O)*C*H), 119.5 (q, ^1J(^{19}F-^{13}C) = 292 Hz, *C*F$_3$), 125.1 (s, *m*-*C*$_6$H$_3$), 125.8 (s, N*C*H), 126.0 (s, N*C*H), 132.3 (s, *p*-*C*$_6$H$_3$), 133.4 (s, *ipso*-*C*$_6$H$_3$), 146.2 (s, *o*-*C*$_6$H$_3$), 146.8 (s, *o*-*C*$_6$H$_3$), 160.3 (s broad, B*C*N), 172.7 (q, ^2J(^{19}F-^{13}C) = 29 Hz, *C*O) ppm. **^{11}B NMR** (25 °C, CD_3CN, 160.42 MHz): δ = −54.9 (d, ^1J(^{11}B-^{1}H) = 146 Hz, 1B, B3), −31.7 (d, ^1J(^{11}B-^{1}H) = 117 Hz, 2B, B6,9), −22.9 (d, ^1J(^{11}B-^{1}H) = 124 Hz, 1B, B1), −7.6 (s broad, 2B, B2,4), −4.9 (s broad, 2B, B7,8) ppm.

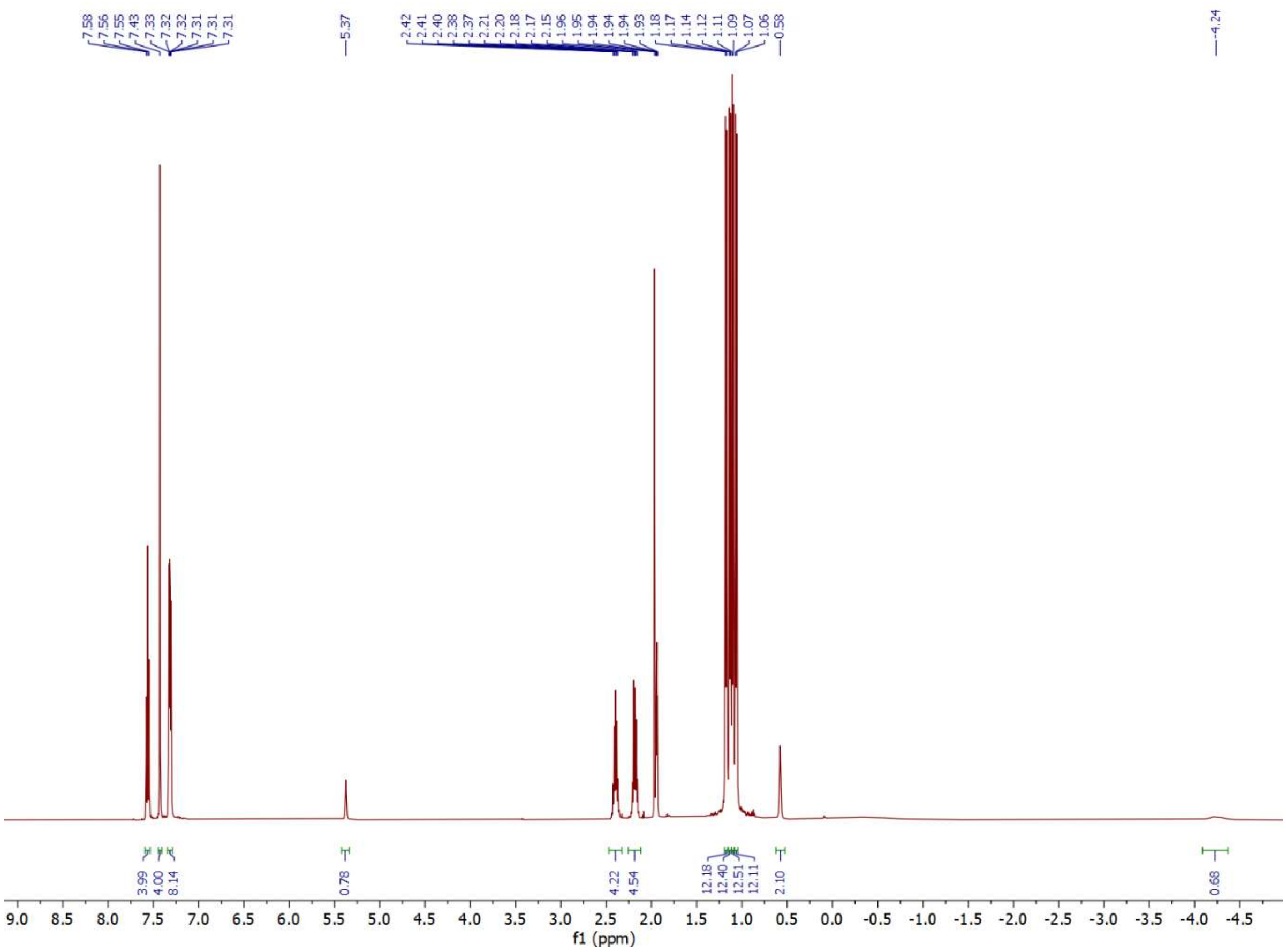

Supplementary Figure S2. The ^{1}H (500.1 MHz) NMR spectrum of *o*-2b in CD_3CN.

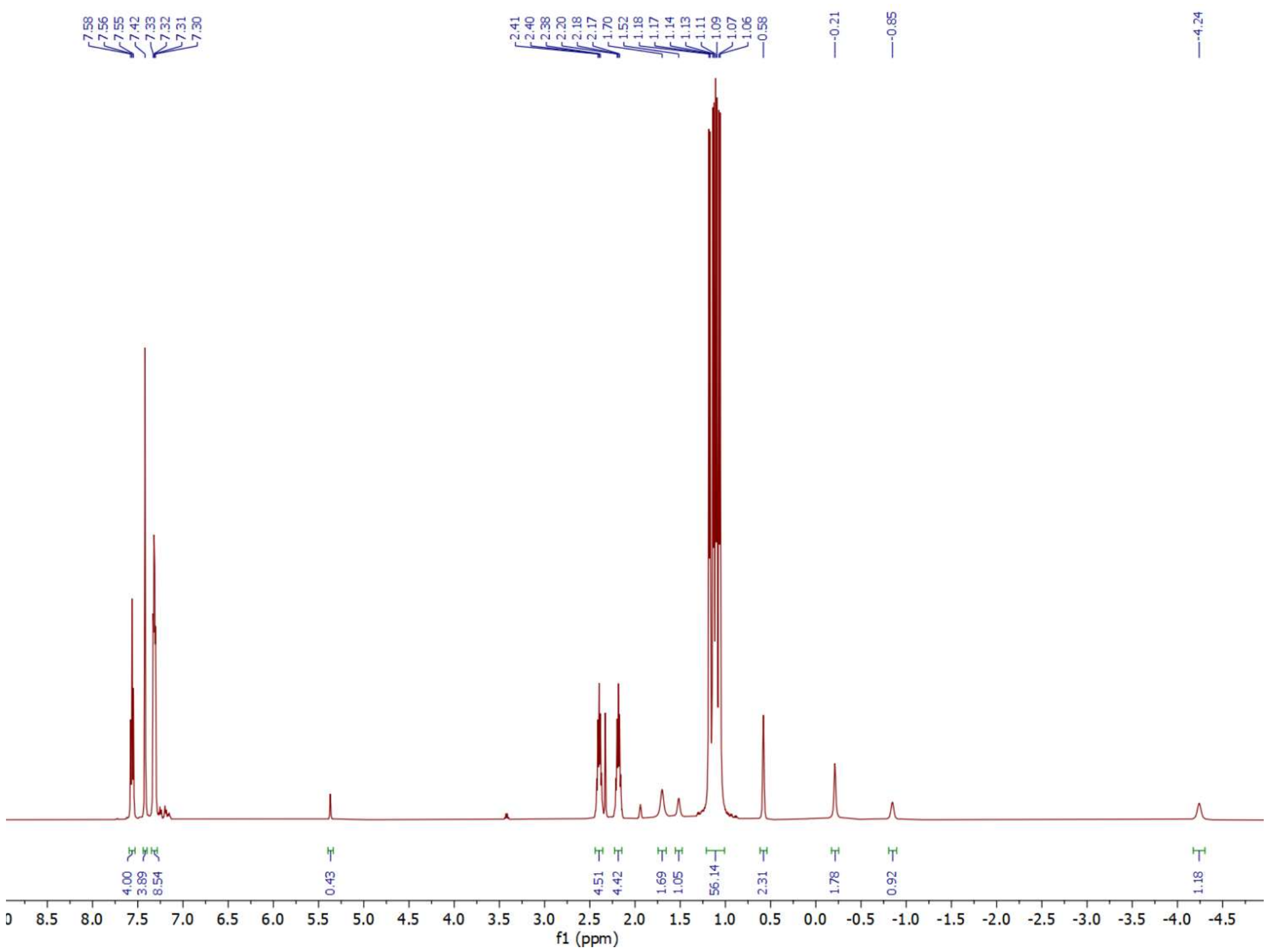

Supplementary Figure S3. The $^{1}H\{^{11}B\}$ (500.1 MHz) NMR spectrum of *o*-2b in CD_3CN.

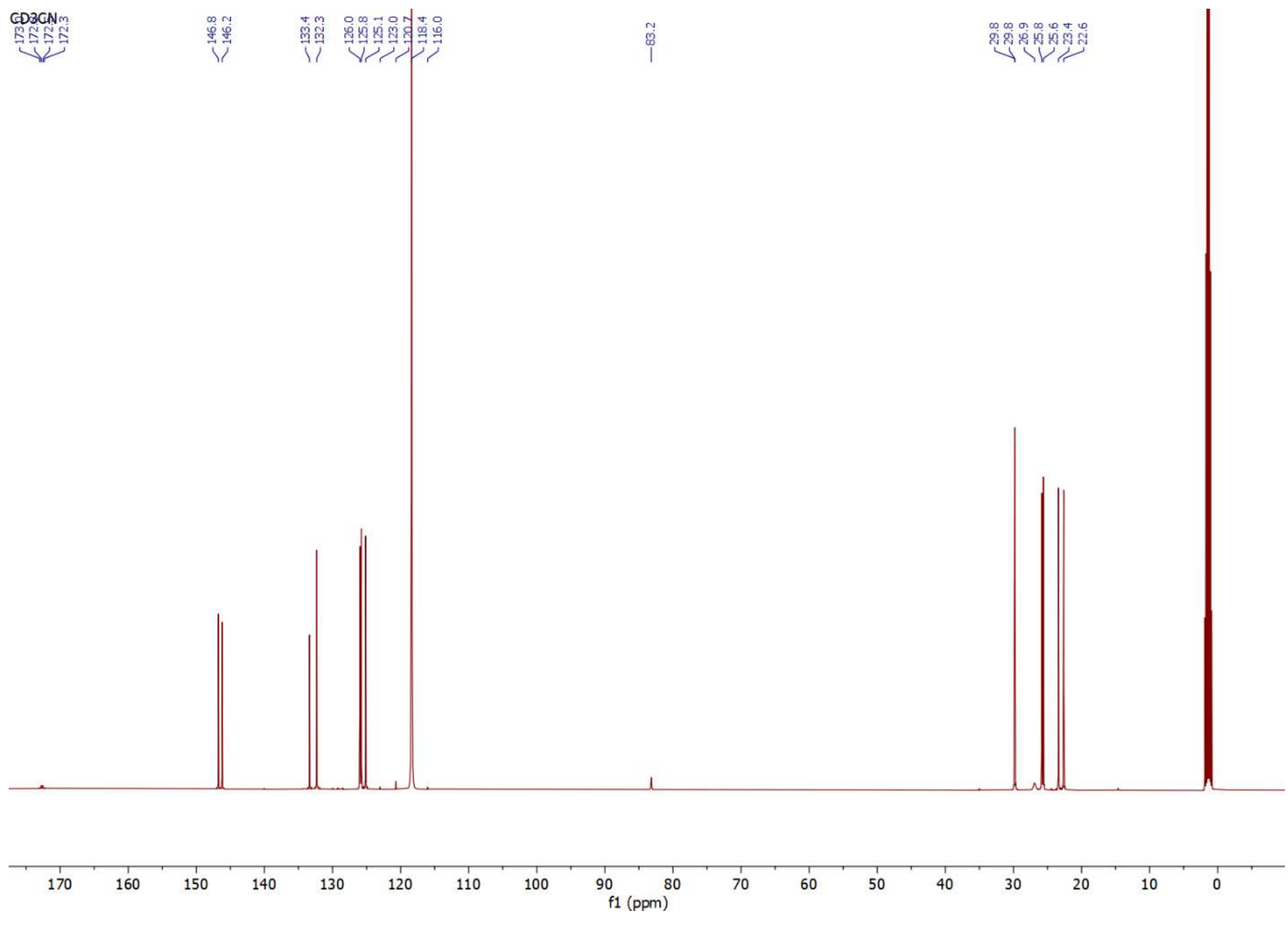

Supplementary Figure S4. The $^{13}C\{^{1}H\}$ (125.8 MHz) NMR spectrum of *o*-2b in CD_3CN.

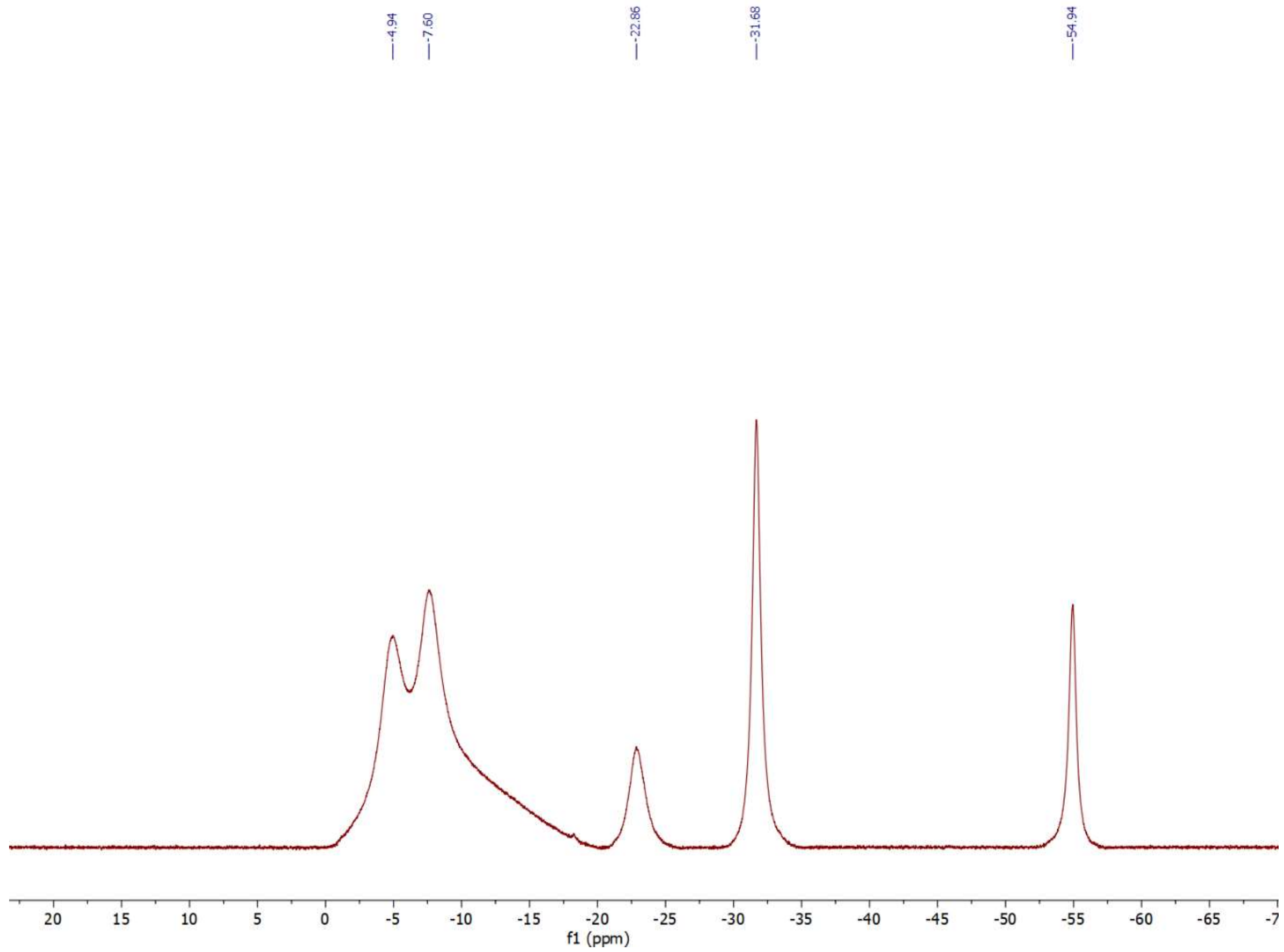

Supplementary Figure S5. The $^{11}B\{^1H\}$ (160.4) NMR spectrum of *o*-2b in CD_3CN.

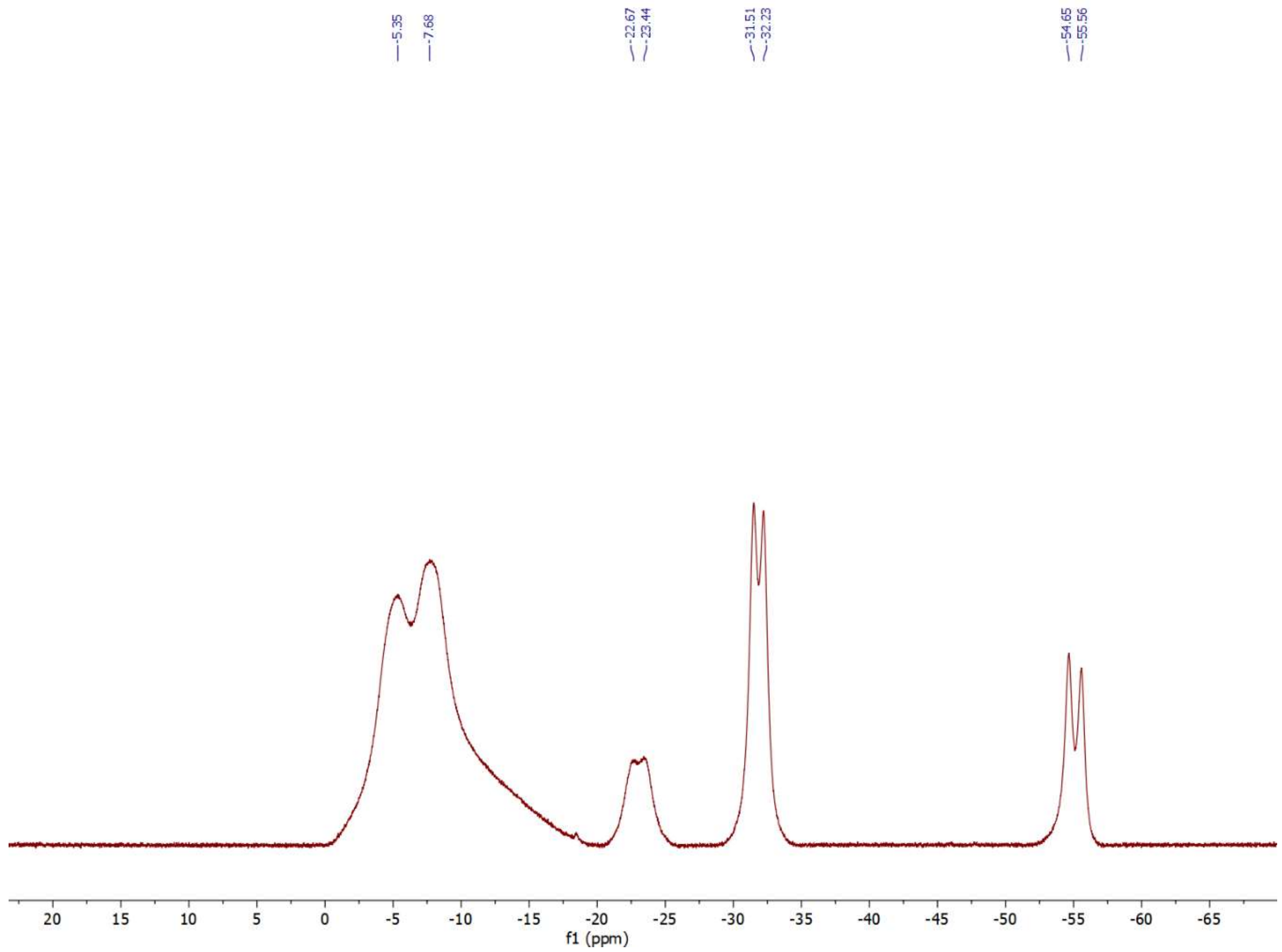

Supplementary Figure S6. The ^{11}B (160.4) NMR spectrum of *o*-2b in CD_3CN.

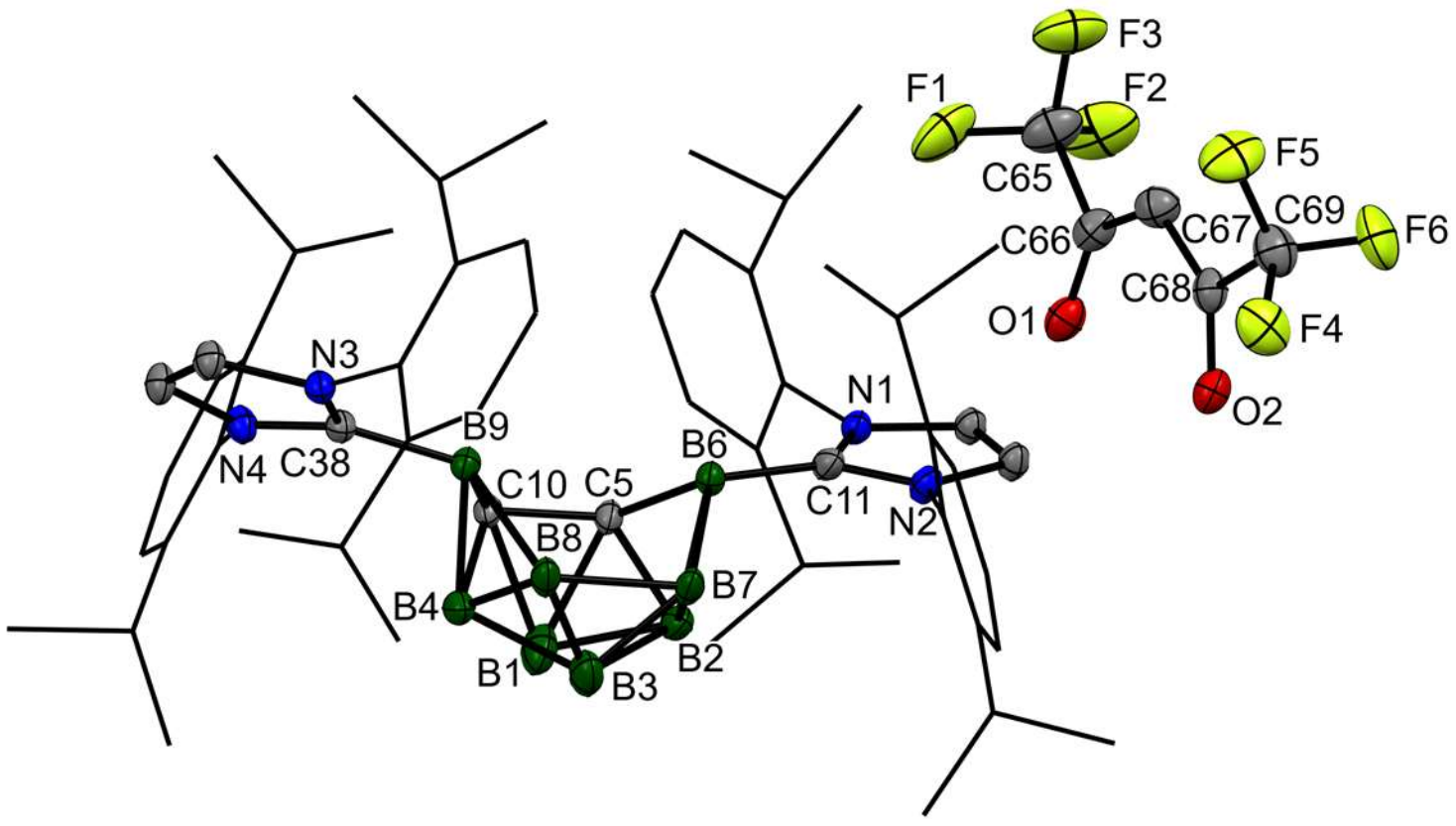

Supplementary Figure S7. The molecular structure of *o*-2b. ORTEP view, 30% probability level; solvent molecule (acetonitrile) has been omitted for clarity. Selected interatomic distances [Å]: B9-C10 1.645(9), C5-C10 1.547(4), C5-B6 1.651(9), B6-B7 1.973(9), B7-B8 1.846(5), B8-B9 1.965(9), C66-O1 1.226(3), C66-C67 1.395(4), C67-C68 1.409(4), C68-O2 1.230(3).

Supplementary Table S1. Experimental details of *o*-2b.

Experimental details

Crystal data	
Chemical formula	$C_{56}H_{84}B_8N_4{\cdot}C_5HF_6O_2{\cdot}C_2H_3N$
M_r	1147.86
Crystal system, space group	Triclinic, *P*-1
Temperature (K)	150
a, *b*, *c* (Å)	11.7007(5), 15.1728(5), 19.5017(8)
α, β, γ (°)	103.395(1), 97.169(2), 92.135(1)
V (Å^3)	3333.8(2)
Z	2
Radiation type	Mo*K*α
μ (mm^{-1})	0.08
Crystal size (mm)	0.42 × 0.26 × 0.23
Data collection	
Diffractometer	Bruker D8 - Venture
Absorption correction	Multi-scan<br>*SADABS2016*/2 - Bruker AXS area detector scaling and absorption correction Reference: Krause, L., Herbst-Irmer, R., Sheldrick G.M. & Stalke D., *J. Appl. Cryst.* 2015, 48, 3–10.
T_{min}, T_{max}	0.707, 0.745
No. of measured, independent and observed [$I > 2\sigma(I)$] reflections	166290, 15226, 11824
R_{int}	0.077
$(\sin \theta/\lambda)_{max}$ (Å^{-1})	0.650
Refinement	
$R[F^2 > 2\sigma(F^2)]$, $wR(F^2)$, S	0.077, 0.225, 1.04
No. of reflections	15226
No. of parameters	949
No. of restraints	942
H-atom treatment	H atoms treated by a mixture of independent and constrained refinement
$\Delta\rho_{max}$, $\Delta\rho_{min}$ (e Å^{-3})	0.69, −0.53

Synthesis of $[6,9\text{-}IPr_2\text{-}5,10\text{-}C_2B_8H_{11}]^+[C(COOEt)_3]^-$ (*o*-2c)

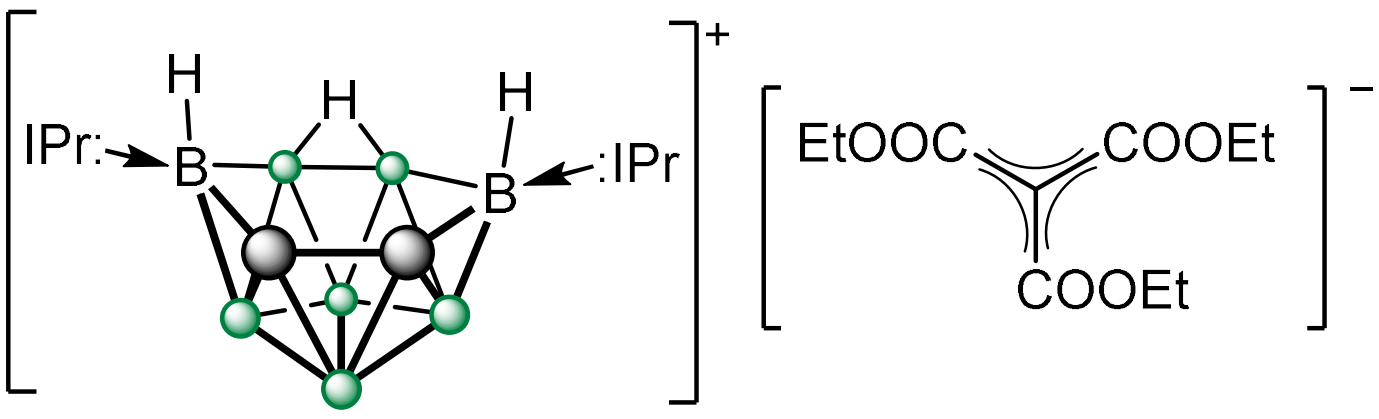

Triethyl methanetricarboxylate (30.0 µL, 0,14 mmol) was added to a stirred solution of *o*-**2** (113 mg, 0.13 mmol) in diethyl ether (5 mL) at room temperature. The resulting suspension was stirred for additional 15 minutes and filtrated. The solid was washed with diethyl ether (10 mL) and the resulting colourless powder of *o*-**2c** was dried *in vacuo*. The crystals suitable for X-ray diffraction analysis were grown from a saturated solution of *o*-**2c** in acetonitrile at 4 °C. Yield 200 mg, 86%.**Dp** 198 °C. **^{1}H NMR** (25 °C, CD_3CN, 500 MHz): δ = −4.24 (s broad, 1H, B*H*B), −0.85 (s, 1H, B3), −0.21 (s, 2H, B6,9), 0.58 (s, 2H, BC*H*), 1.07 (d, $^3J(^1H\text{-}^1H)$ = 6.8 Hz, 14H, $CH(CH_3)_2$ + B7,8), 1.10 (d, $^3J(^1H\text{-}^1H)$ = 6.8 Hz, 12H, $CH(CH_3)_2$), 1.10 (t, $^3J(^1H\text{-}^1H)$ = 7.1 Hz, 9H, CH_2CH_3), 1.13 (d, $^3J(^1H\text{-}^1H)$ = 6.8 Hz, 12H, $CH(CH_3)_2$), 1.17 (d, $^3J(^1H\text{-}^1H)$ = 6.8 Hz, 12H, $CH(CH_3)_2$), 1.51 (s, 1H, B1), 1.70 (s, 2H, B2,4), 2.18 (sept, $^3J(^1H\text{-}^1H)$ = 6.8 Hz, 4H, $CH(CH_3)_2$), 2.40 (sept, $^3J(^1H\text{-}^1H)$ = 6.8 Hz, 4H, $CH(CH_3)_2$), 3.86 (q, $^3J(^1H\text{-}^1H)$ = 7.1 Hz, 6H, CH_2CH_3), 7.31 (d, $^3J(^1H\text{-}^1H)$ = 7.7Hz, 4H, *m*-C_6H_3), 7.32 (d, $^3J(^1H\text{-}^1H)$ = 7.7 Hz, 4H, *m*-C_6H_3), 7.45 (s, 4H, NC*H*), 7.56 (t, $^3J(^1H\text{-}^1H)$ = 7.7 Hz, 4H, *p*-C_6H_3) ppm. **$^{13}C\{^1H\}$ NMR** (25 °C, CD_3CN, 125.76 Hz): δ = 15.4 (s, CH_2CH_3), 22.6 (s, $CH(CH_3)_2$), 23.4 (s, $CH(CH_3)_2$), 25.6 (s, $CH(CH_3)_2$), 25.8 (s, $CH(CH_3)_2$), 26.9 (s broad, B*C*H), 29.8 (s, $CH(CH_3)_2$), 57.7 (s, CH_2CH_3), 76.6 (s, *C*(CO)$_3$), 125.1 (s, *m*-C_6H_3), 125.8 (s, N*C*H), 126.0 (s, N*C*H), 132.3 (s, *p*-C_6H_3), 133.4 (s, *ipso*-C_6H_3), 146.2 (s, *o*-C_6H_3), 146.8 (s, *o*-C_6H_3), 160.3 (s broad, B*C*N), 170.4 (s, *C*(=O)O) ppm. **^{11}B NMR** (25 °C, CD_3CN, 160.42 MHz): δ = -55.0 (d, $^1J(^{11}B\text{-}^1H)$ = 145 Hz, 1B, B3), −31.7 (d, $^1J(^{11}B\text{-}^1H)$ = 113 Hz, 2B, B6,9), −22.9 (d, $^1J(^{11}B\text{-}^1H)$ = 148 Hz, 1B, B1), −7.6 (s broad, 2B, B2,4), −4.8 (s broad, 2B, B7,8) ppm.

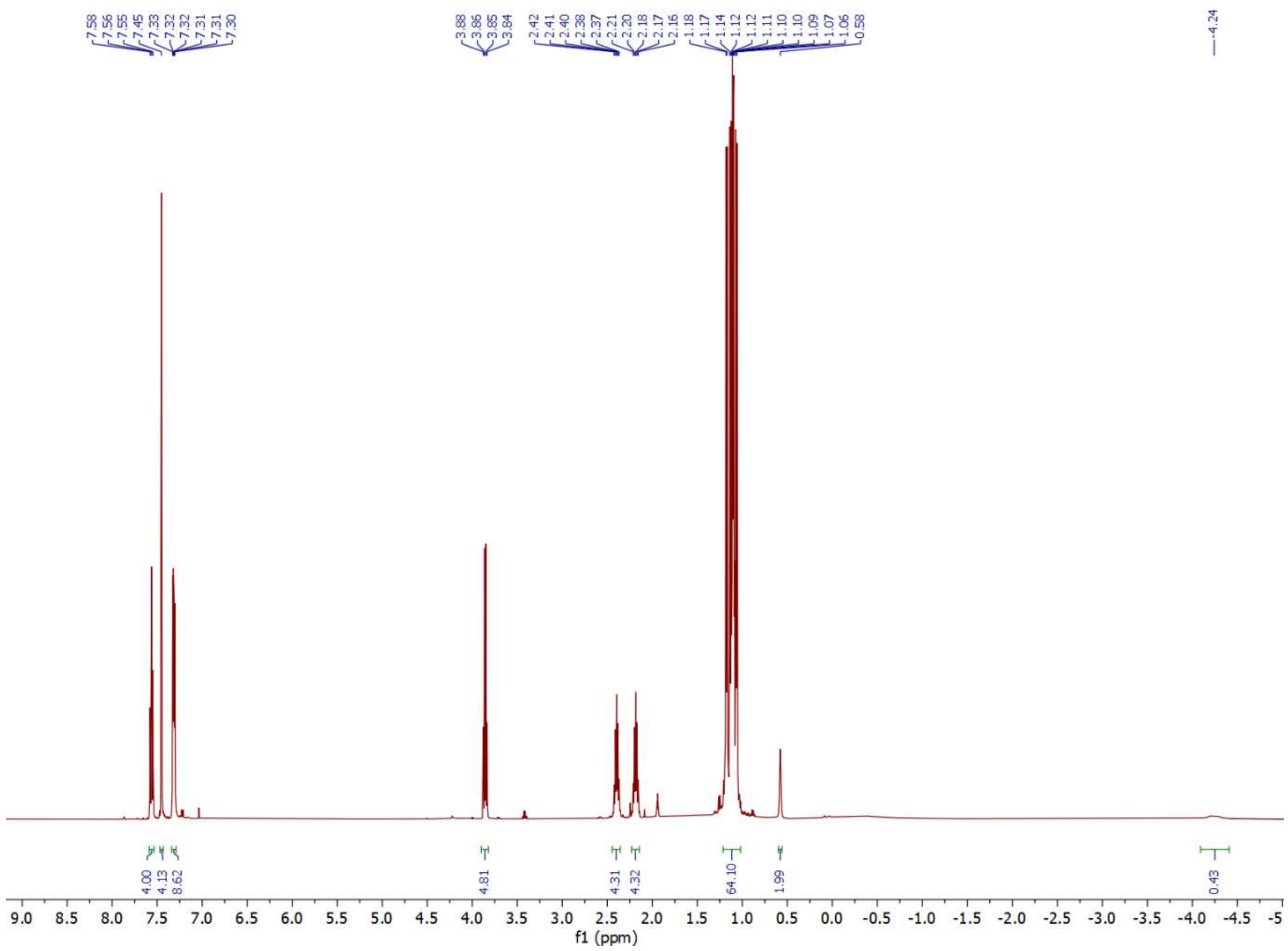

Supplementary Figure S8. The ^{1}H (500.1 MHz) NMR spectrum of *o*-2c in CD_3CN.

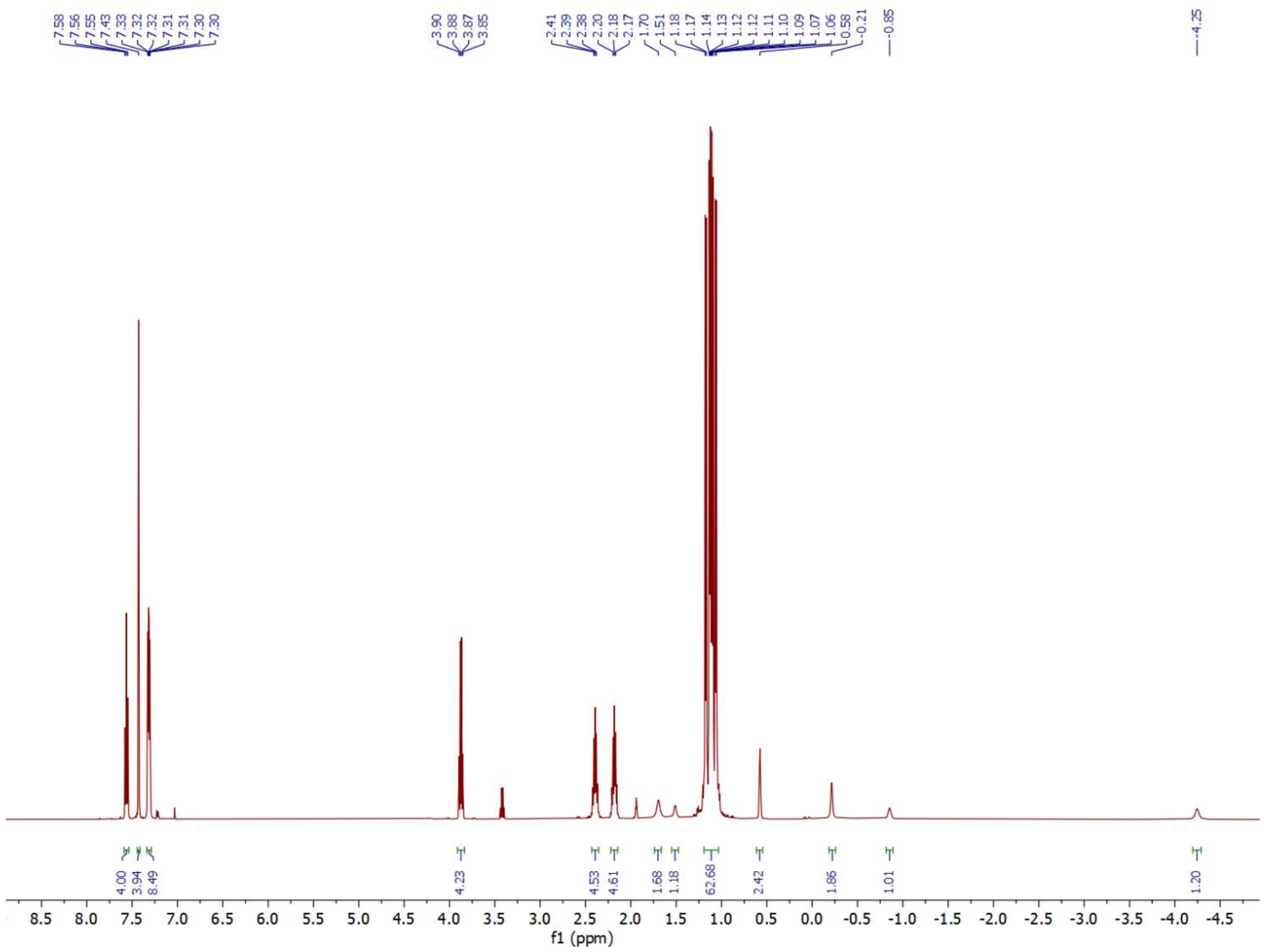

Supplementary Figure S9. The $^{1}H\{^{11}B\}$ (500.1) NMR spectrum of *o*-2c in CD_3CN.

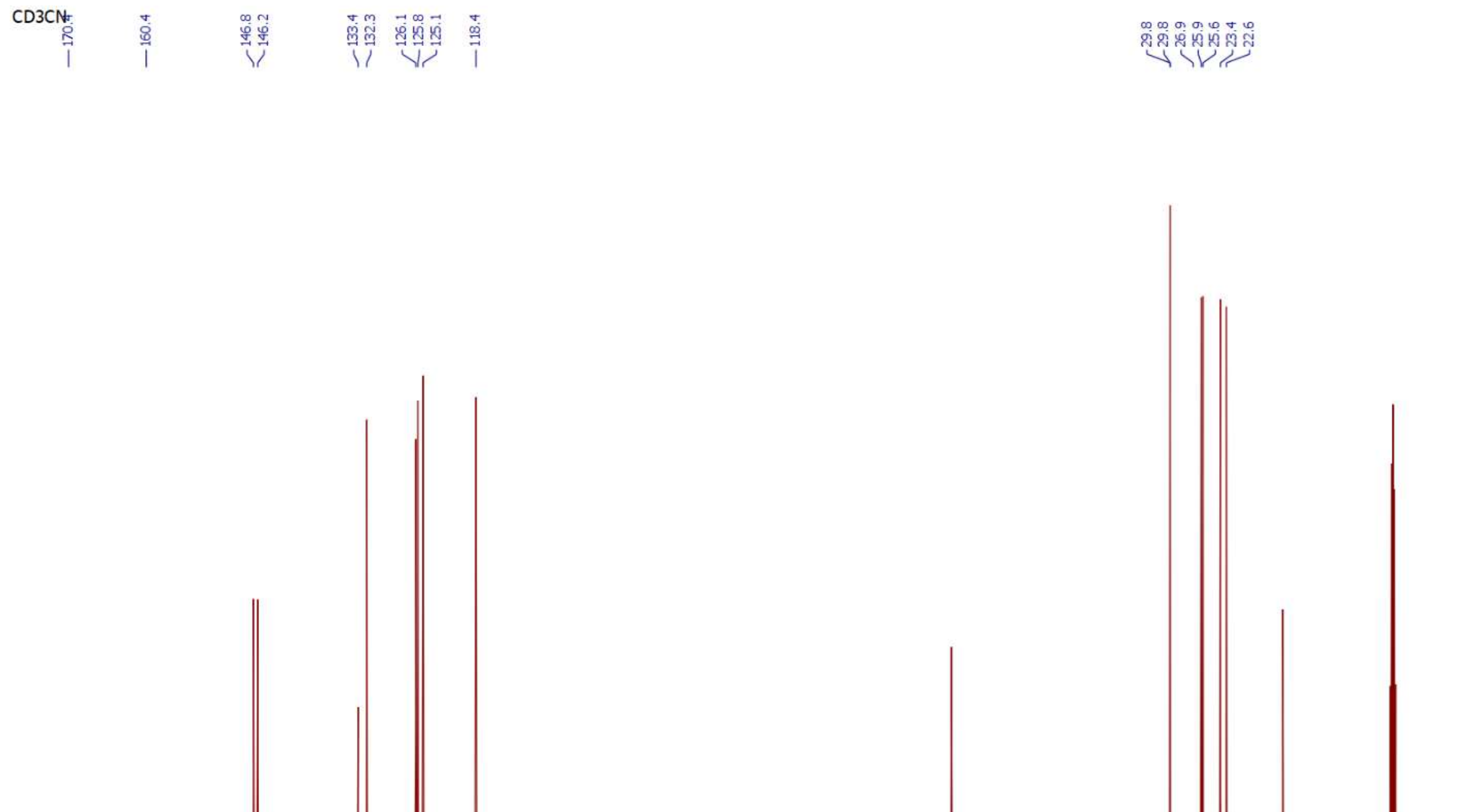

Supplementary Figure S10. The $^{13}C\{^{1}H\}$ (125.8 MHz) NMR spectrum of *o*-2c in CD_3CN.

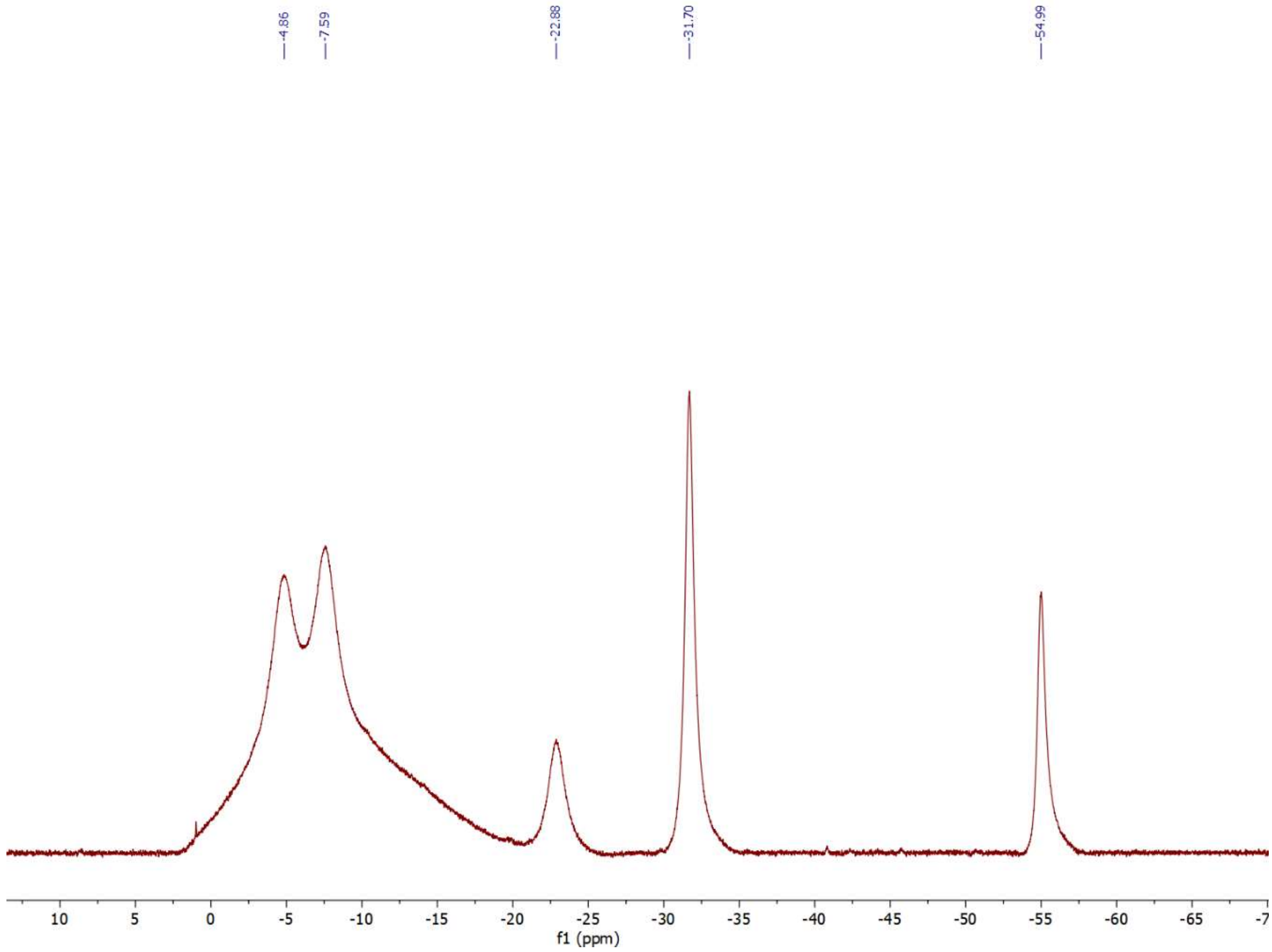

Supplementary Figure S11. The $^{11}B\{^{1}H\}$ (160.4) NMR spectrum of *o*-2c in CD_3CN.

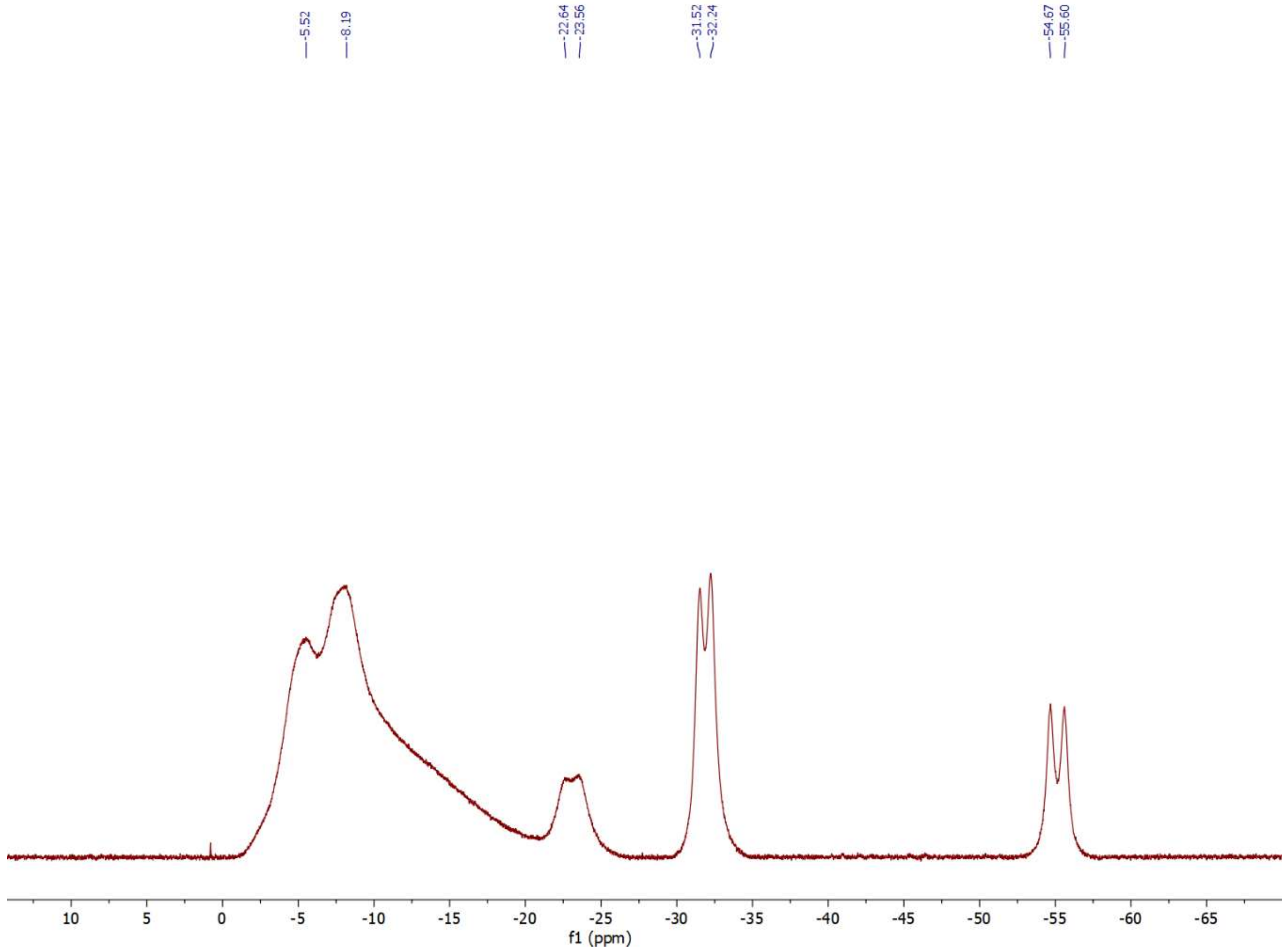

Supplementary Figure S12. The ^{11}B (160.4) NMR spectrum of *o*-2c in CD_3CN.

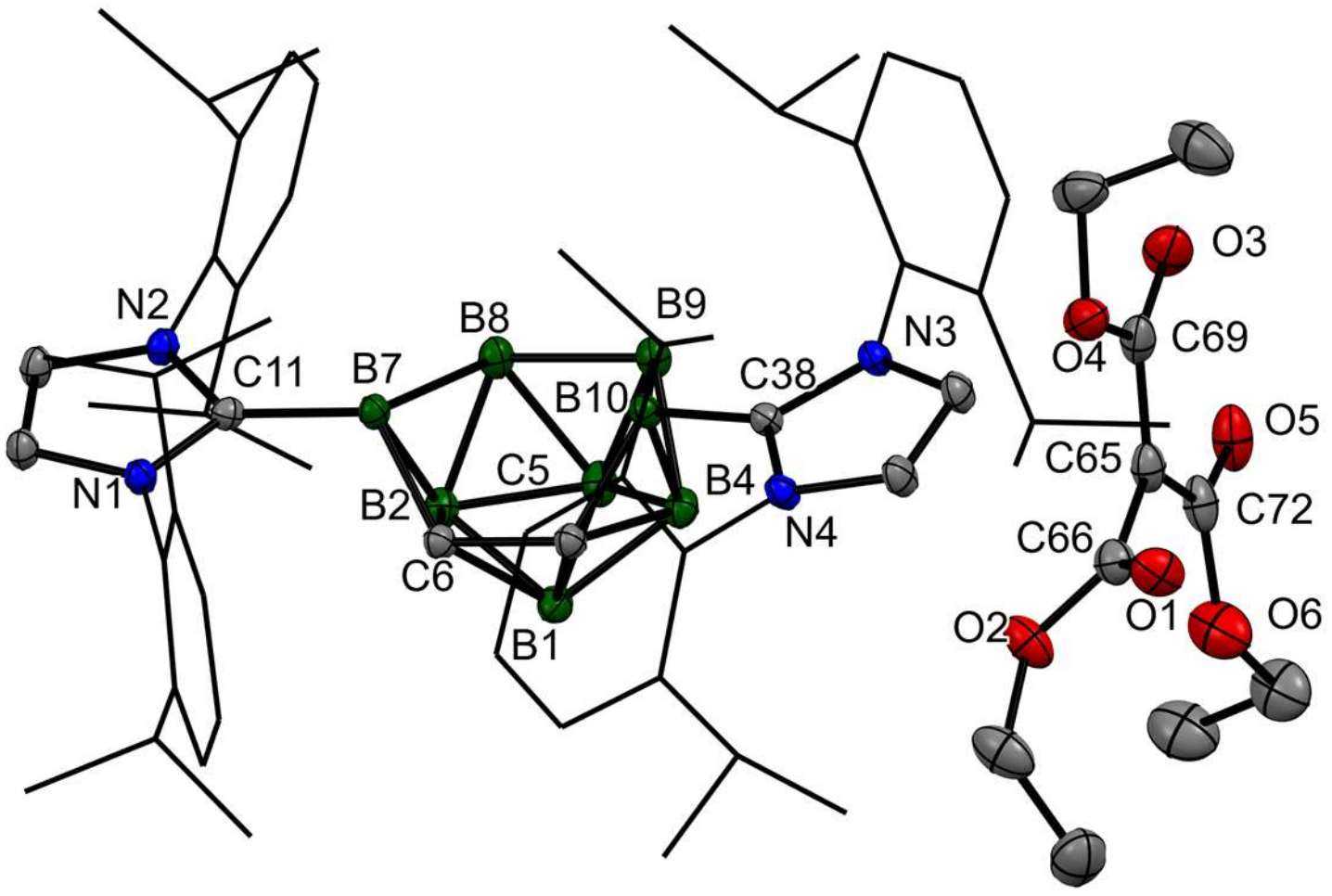

Supplementary Figure S13. The molecular structure of *o*-2c. ORTEP view, 30% probability level; solvent molecule (acetonitrile) has been omitted for clarity. Selected interatomic distances [Å]: B7-C6 1.6314(19), C5-C6 1.5292(18), C5-B10 1.6349(19), B9-B10 1.997(2), B7-B8 1.997(2), B8-B9 1.843(2), C66-O1 1.226(2), C65-C66 1.467(2), C65-C69 1.425(2), C65-C72 1.419(2), C69-O3 1.226(2), C72-O5 1.227(2).

Supplementary Table S2. Experimental details of *o*-**2c**.

Crystal data	
Chemical formula	$C_{56}H_{83}B_8N_4 \cdot C_{10}H_{15}O_6 \cdot C_2H_3N$
M_r	1171.01
Crystal system, space group	Triclinic, *P*-1
Temperature (K)	150
a, *b*, *c* (Å)	14.6688(4), 15.7415(4), 17.0295(5)
α, β, γ (°)	99.249(1), 91.017(1), 113.155(1)
V (Å^3)	3554.27 (17)
Z	2
Radiation type	Mo*K*α
μ (mm^{-1})	0.07
Crystal size (mm)	0.31 × 0.28 × 0.21
Data collection	
Diffractometer	Bruker D8 - Venture
Absorption correction	Multi-scan<br>*SADABS2016*/2 - Bruker AXS area detector scaling and absorption correction Reference: Krause, L., Herbst-Irmer, R., Sheldrick G.M. & Stalke D., *J. Appl. Cryst.* 2015, 48, 3–10.
T_{min}, T_{max}	0.700, 0.746
No. of measured, independent and observed [$I > 2\sigma(I)$] reflections	212713, 17559, 13219
R_{int}	0.073
$(\sin \theta/\lambda)_{max}$ (Å^{-1})	0.667
Refinement	
$R[F^2 > 2\sigma(F^2)]$, $wR(F^2)$, S	0.056, 0.155, 1.02
No. of reflections	17559
No. of parameters	867
No. of restraints	762
H-atom treatment	H atoms treated by a mixture of independent and constrained refinement
$\Delta\rho_{max}$, $\Delta\rho_{min}$ (e Å^{-3})	0.51, −0.33

Synthesis of $[6,9\text{-IPr}_2\text{-}5,10\text{-C}_2\text{B}_8\text{H}_{11}]^+[\text{HC}(\mu\text{-C(O)O})_2\text{C}(\text{CH}_3)_2]^-$ (*o*-2d)

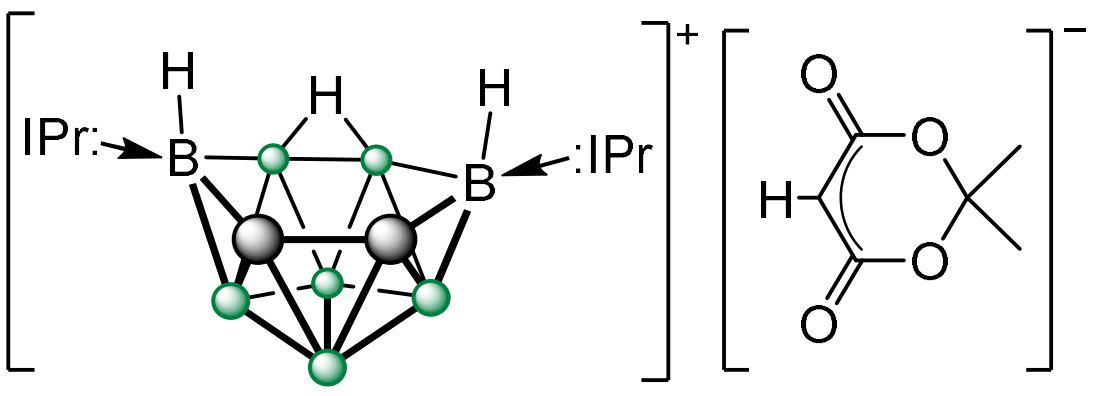

Solution of 2,2-dimethyl-1,3-dioxane-4,6-dione (34 mg, 0.24 mmol) in acetonitrile (5 mL) was added to a stirred solution of *o*-**2** (141 mg, 0.16 mmol) in acetonitrile (5 mL) at room temperature. The reaction mixture was stirred overnight, and the solvent was removed in vacuo. The solid was washed twice with diethyl ether (10 mL) and the resulting colourless powder of *o*-**2d** was dried in vacuo. Yield 113 mg, 69%, **Dp** 178 °C. **^{1}H NMR** (25 °C, CD_3CN, 500 MHz): δ = –4.24 (s broad, 1H, B*H*B), −0.84 (s, 1H, B3), −0.21 (s, 2H, B6,9), 0.58 (s, 2H, BC*H*), 1.06 (d, $^3J(^1\text{H-}^1\text{H})$ = 6.8 Hz, 14H, CH(C*H*$_3$)$_2$ + B7,8), 1.10 (d, $^3J(^1\text{H-}^1\text{H})$ = 6.8 Hz, 12H, CH(C*H*$_3$)$_2$), 1.13 (d, $^3J(^1\text{H-}^1\text{H})$ = 6.8 Hz, 12H, CH(C*H*$_3$)$_2$), 1.17 (d, $^3J(^1\text{H-}^1\text{H})$ = 6.8 Hz, 12H, CH(C*H*$_3$)$_2$), 1.51 (s, 7H, C(C*H*$_3$)$_2$ + B1), 1.70 (s, 2H, B2,4), 2.18 (sept, $^3J(^1\text{H-}^1\text{H})$ = 6.8 Hz, 4H, C*H*(CH$_3$)$_2$), 2.39 (sept, $^3J(^1\text{H-}^1\text{H})$ = 6.8 Hz, 4H, C*H*(CH$_3$)$_2$), 3.34 (m, 1H, C(O)C*H*), 7.31 (d, $^3J(^1\text{H-}^1\text{H})$ = 7.7 Hz, 4H, *m*-C$_6$*H*$_3$), 7.32 (d, $^3J(^1\text{H-}^1\text{H})$ = 7.7 Hz, 4H, *m*-C$_6$*H*$_3$), 7.43 (m, 4H, NC*H*), 7.56 (t, $^3J(^1\text{H-}^1\text{H})$ = 7.8 Hz, 4H, *p*-C$_6$*H*$_3$) ppm. **^{13}C{^{1}H} NMR** (25 °C, CD_3CN, 125.76 Hz): δ = 22.6 (s, CH(*C*H$_3$)$_2$), 23.4 (s, CH(*C*H$_3$)$_2$), 25.6 (s, CH(*C*H$_3$)$_2$), 25.8 (s, CH(*C*H$_3$)$_2$), 26.4 (s, C(*C*H$_3$)$_2$), 26.9 (s broad, B*C*H), 29.81 (s, *C*H(CH$_3$)$_2$), 29.83 (s, *C*H(CH$_3$)$_2$), 62.9 (s, C(O)*C*H), 100.9 (s, *C*(CH$_3$)$_2$), 125.2 (s, *m-C*$_6$H$_3$), 125.8 (s, N*C*H), 126.0 (s, N*C*H), 132.3 (s, *p-C*$_6$H$_3$), 133.4 (s, *ipso-C*$_6$H$_3$), 146.2 (s, *o-C*$_6$H$_3$), 146.8 (s, *o-C*$_6$H$_3$), 160.1 (s broad, B*C*N), 167.9 (s, O*C*O) ppm. **^{11}B NMR** (25 °C, CD_3CN, 160.42 MHz): δ = −55.2 (d, $^1J(^{11}\text{B-}^1\text{H})$ = 155 Hz, 1B, B3), −31.9 (d, $^1J(^{11}\text{B-}^1\text{H})$ = 114 Hz, 2B, B6,9), −22.9 (d, $^1J(^{11}\text{B-}^1\text{H})$ = 136 Hz, 2B, 1B, B1), −7.6 (s broad, 2B, B2,4), −4.9 (s broad, 2B, B7,8) ppm.

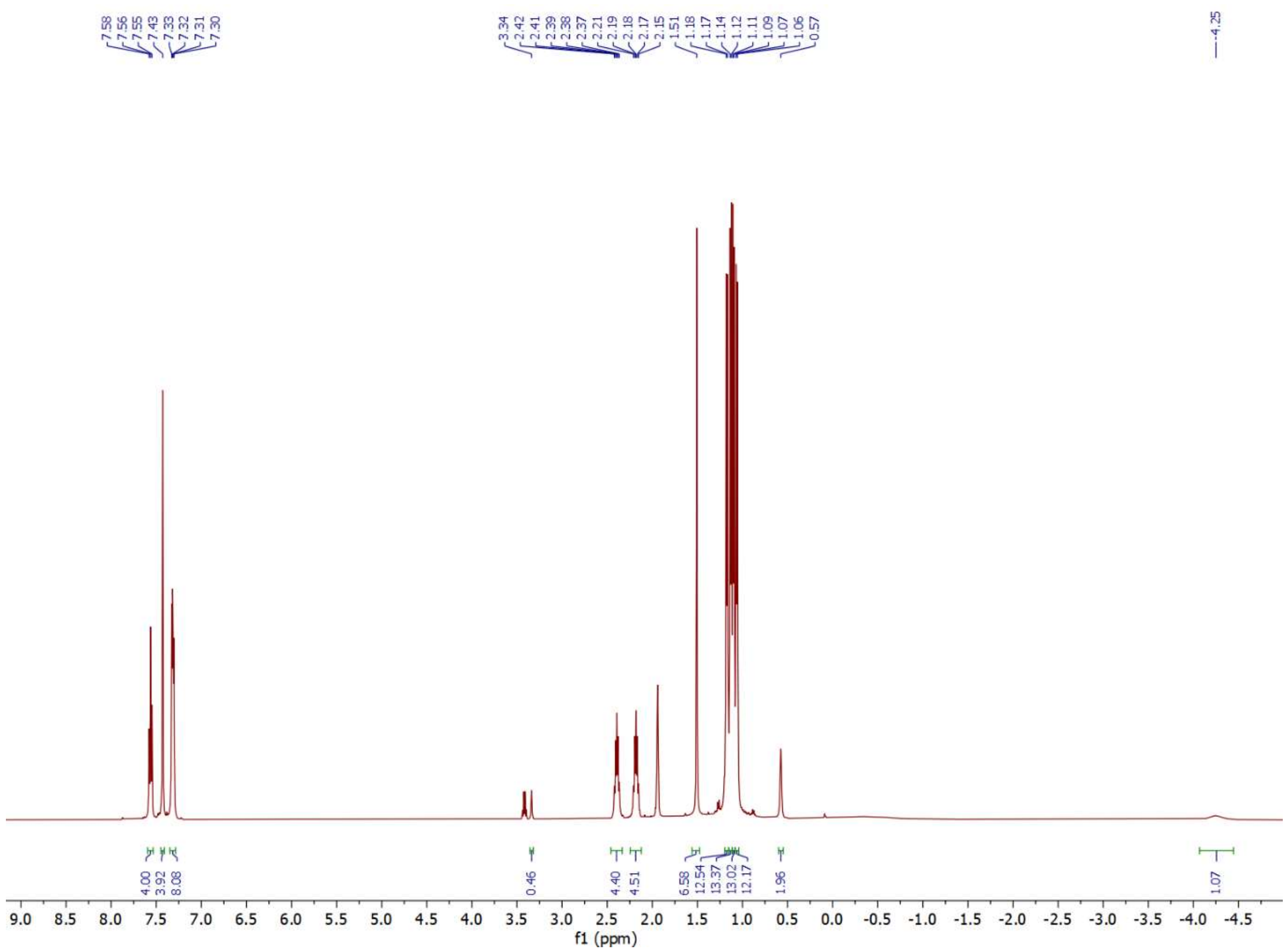

Supplementary Figure S14. The ^{1}H (500.1 MHz) NMR spectrum of *o*-2d in CD_3CN.

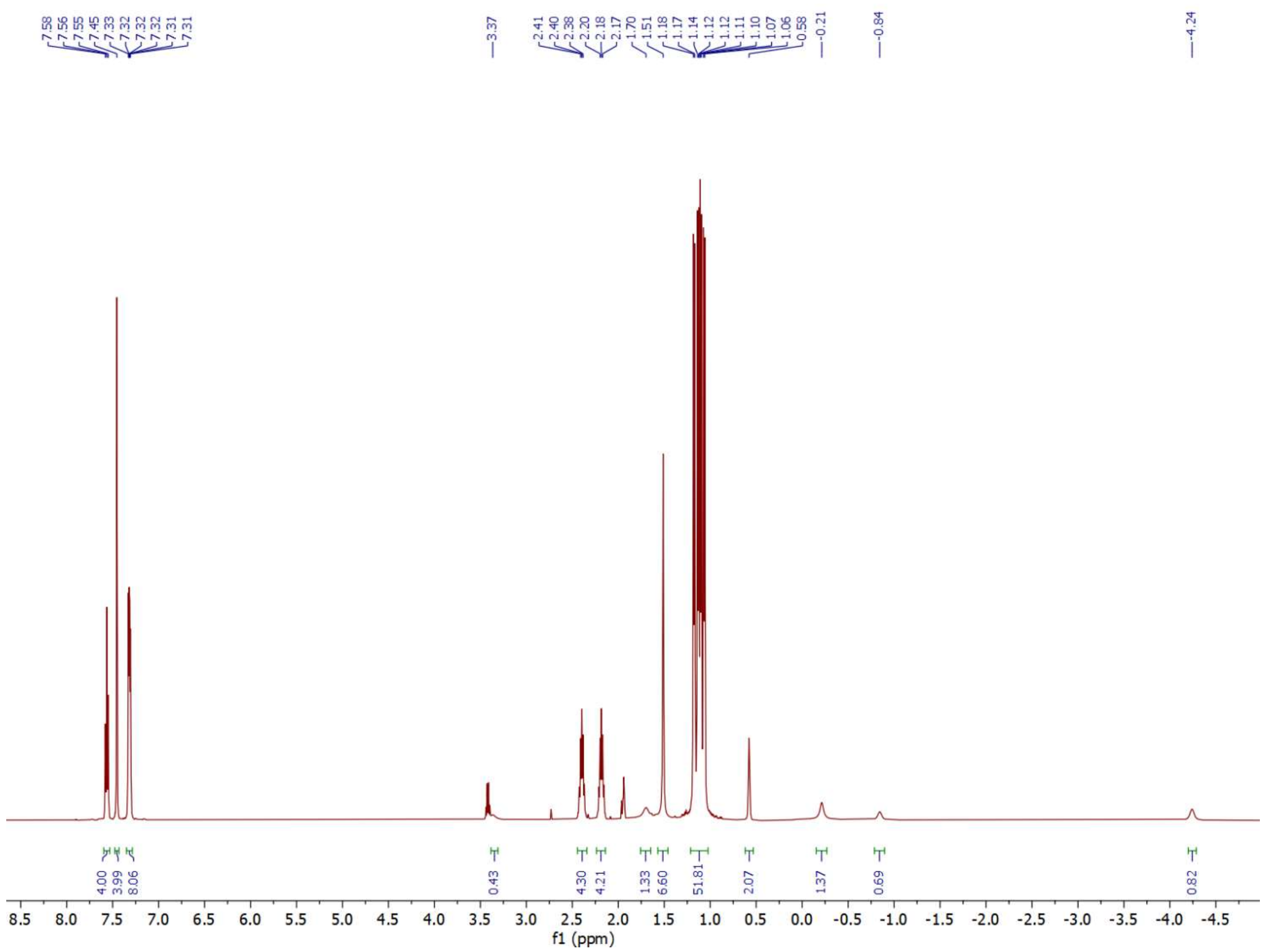

Supplementary Figure S15. The $^{1}H\{^{11}B\}$ (500.1) NMR spectrum of *o*-2d in CD_3CN.

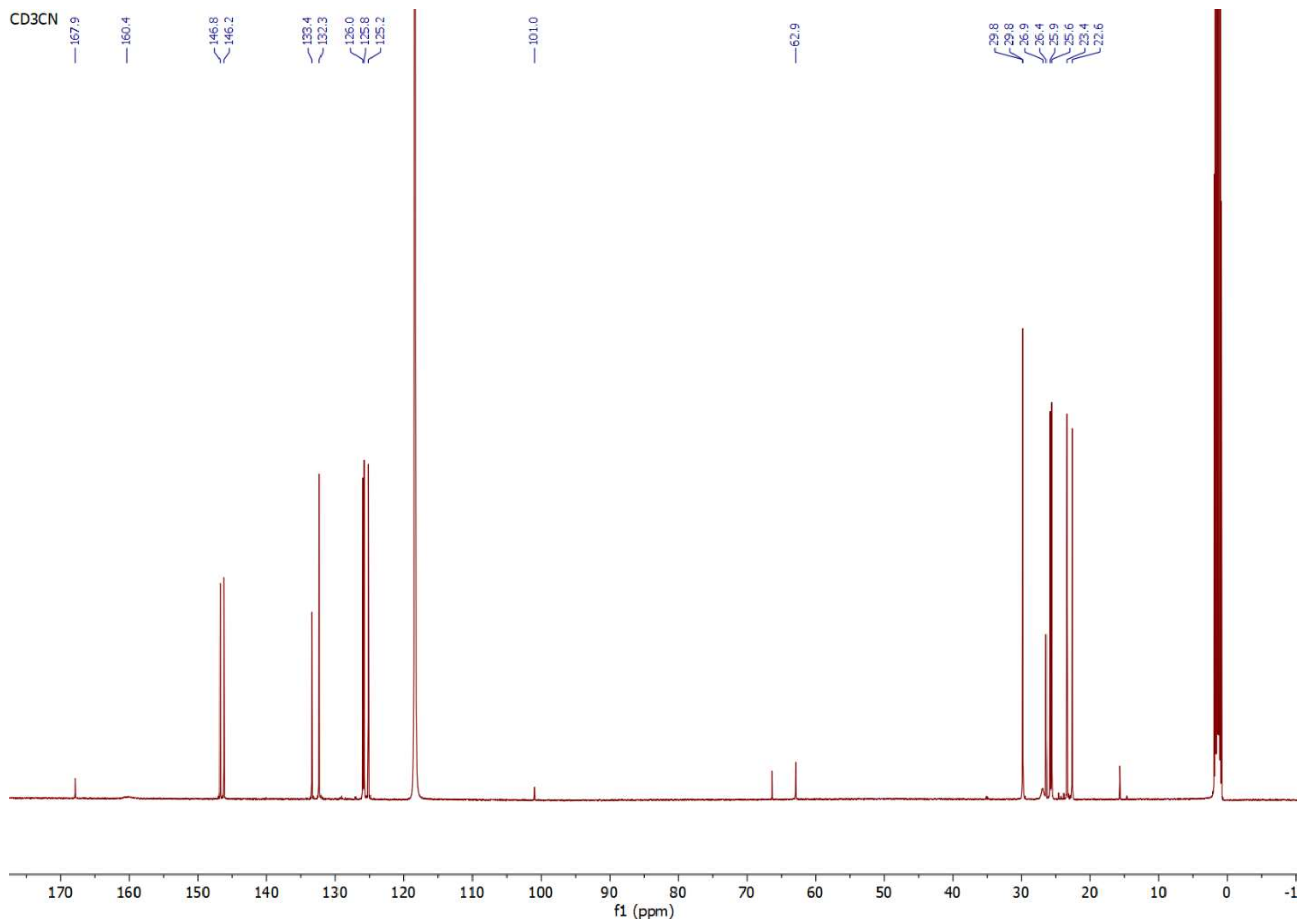

Supplementary Figure S16. The $^{13}C\{^{1}H\}$ (125.8 MHz) NMR spectrum of *o*-2d in CD_3CN.

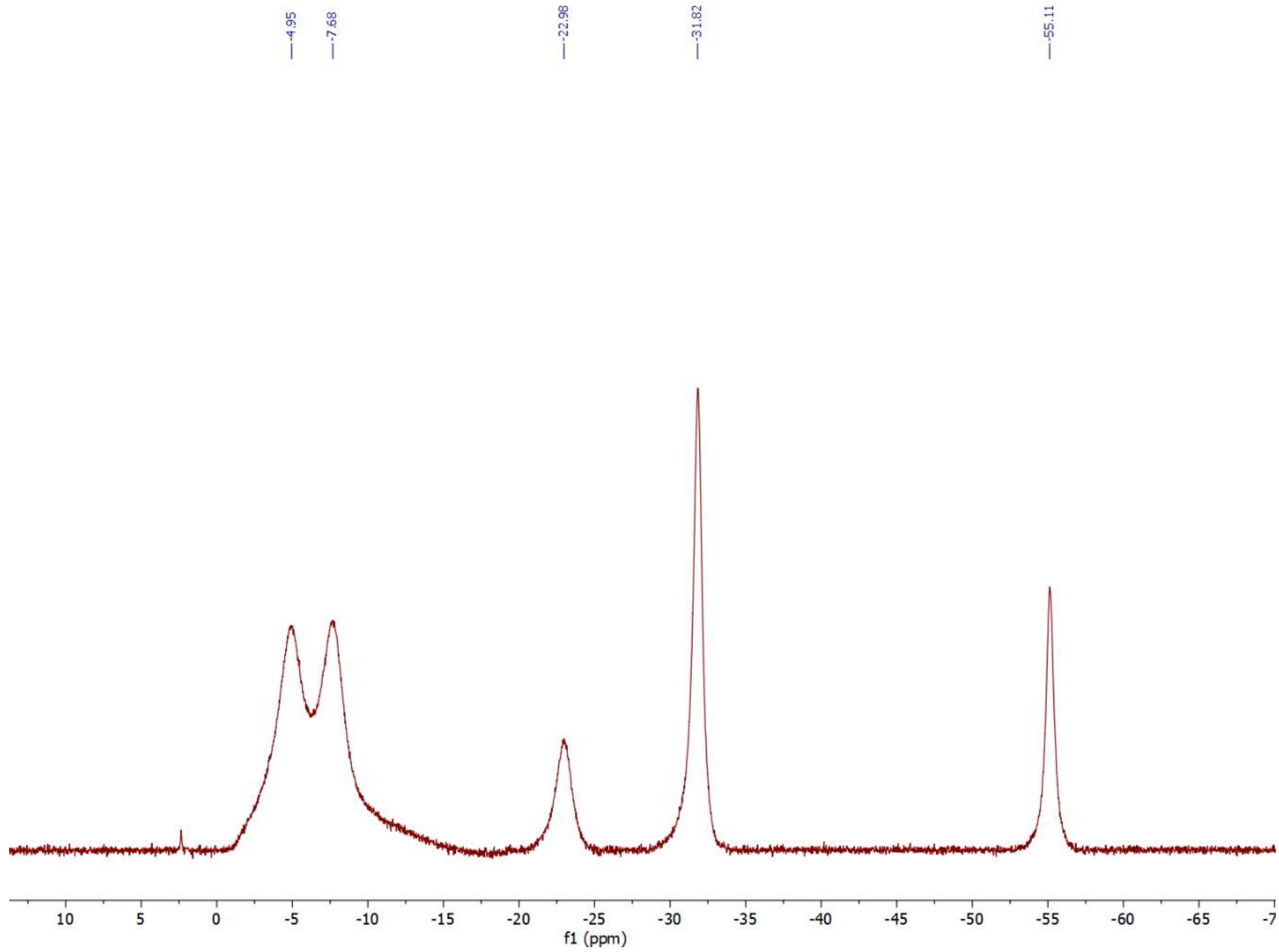

Supplementary Figure S17. The $^{11}B\{^{1}H\}$ (160.4) NMR spectrum of *o*-2d in CD_3CN.

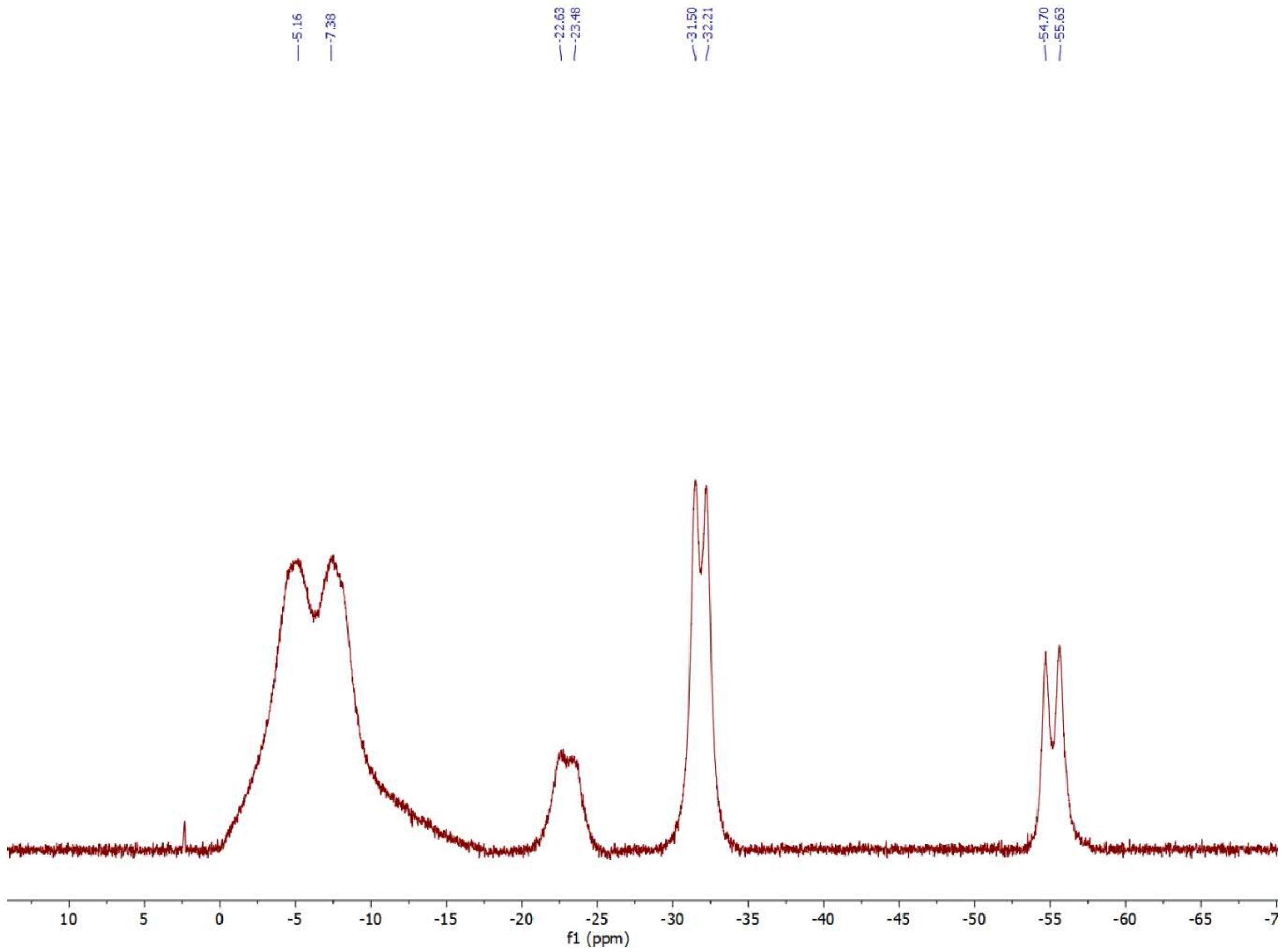

Supplementary Figure S18. The ^{11}B (160.4) NMR spectrum of *o*-2d in CD_3CN.

Synthesis of $[6,9\text{-}IPr_2\text{-}5,10\text{-}C_2B_8H_{11}]^+[PhO]^-$ .2PhOH (*o*-2e)

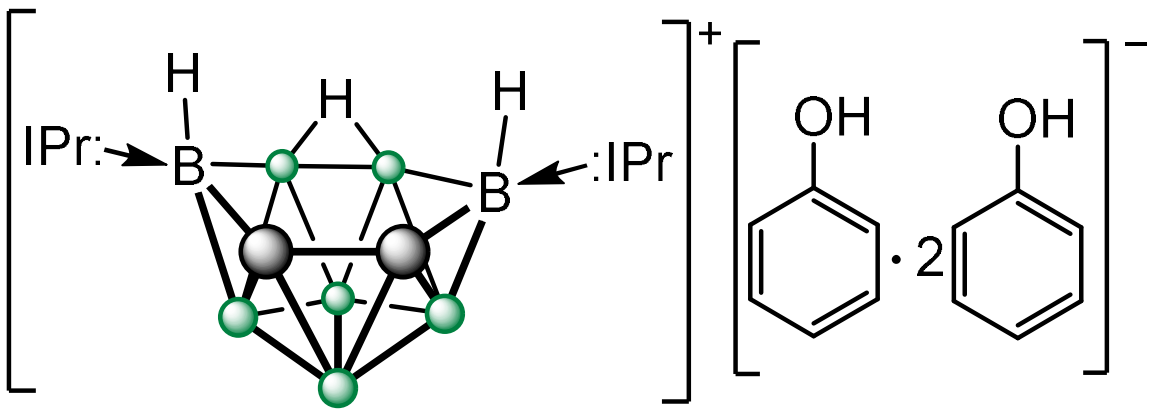

Solution of phenol (41 mg, 0.44 mmol) in acetonitrile (5 mL) was added to a stirred solution of *o*-**2** (97 mg, 0.11 mmol) in acetonitrile (5 mL) at room temperature. The reaction mixture was stirred overnight, and the solvent was removed in vacuo. The solid was washed twice with diethyl ether (15 mL) and the resulting colourless powder of *o*-**2e** was dried in vacuo. Yield 104 mg, 97%, **Dp** 178 °C. **^{1}H NMR** (25 °C, CD_3CN, 500 MHz): δ = −4.24 (s broad, 1H, B*H*B), −0.84 (s, 1H, B3), −0.21 (s, 2H, B6,9), 0.58 (s, 2H, BC*H*), 1.06 (d, $^3J(^1H\text{-}^1H)$ = 6.8 Hz, 14H, CH(CH_3)$_2$ + B7,8), 1.10 (d, $^3J(^1H\text{-}^1H)$ = 6.8 Hz, 12H, CH(CH_3)$_2$), 1.13 (d, $^3J(^1H\text{-}^1H)$ = 6.8 Hz, 12H, CH(CH_3)$_2$), 1.17 (d, $^3J(^1H\text{-}^1H)$ = 6.8 Hz, 12H, CH(CH_3)$_2$), 1.52 (s, 1H, B1), 1.70 (s, 2H, B2,4), 2.18 (sept, $^3J(^1H\text{-}^1H)$ = 6.8 Hz, 4H, C*H*(CH_3)$_2$), 2.39 (sept, $^3J(^1H\text{-}^1H)$ = 6.8 Hz, 4H, C*H*(CH_3)$_2$), 6.55 (t, $^3J(^1H\text{-}^1H)$ = 7.3 Hz, 3H, *p*-C_6H_5), 6.71 (t, $^3J(^1H\text{-}^1H)$ = 7.7 Hz, 6H, *o*-C_6H_5), 7.04 (t, $^3J(^1H\text{-}^1H)$ = 8.0 Hz, 6H, *m*-C_6H_5), 7.31 (d, $^3J(^1H\text{-}^1H)$ = 7.7 Hz, 4H, *m*-C_6H_3), 7.32 (d, $^3J(^1H\text{-}^1H)$ = 7.7 Hz, 4H, *m*-C_6H_3), 7.43 (m, 4H, NC*H*), 7.56 (t, $^3J(^1H\text{-}^1H)$ = 7.8 Hz, 4H, *p*-C_6H_3) ppm. **$^{13}C\{^1H\}$ NMR** (25 °C, CD_3CN, 125.76 Hz): δ = 22.6 (s, CH(CH_3)$_2$), 23.4 (s, CH(CH_3)$_2$), 25.6 (s, CH(CH_3)$_2$), 25.8 (s, CH(CH_3)$_2$), 26.4 (s, C(CH_3)$_2$), 26.9 (s broad, B*C*H), 29.81 (s, *C*H(CH_3)$_2$), 29.83 (s, *C*H(CH_3)$_2$), 62.9 (s, C(O)*C*H), 100.9 (s, *C*(CH_3)$_2$), 125.2 (s, *m*-C_6H_3), 125.8 (s, N*C*H), 126.0 (s, N*C*H), 132.3 (s, *p*-C_6H_3), 133.4 (s, *ipso*-C_6H_3), 146.2 (s, *o*-C_6H_3), 146.8 (s, *o*-C_6H_3), 160.1 (s broad, B*C*N), 167.9 (s, O*C*O) ppm. **^{11}B NMR** (25 °C, CD_3CN, 160.42 MHz): δ = −55.2 (d, $^1J(^{11}B\text{-}^1H)$ = 155 Hz, 1B, B3), −31.9 (d, $^1J(^{11}B\text{-}^1H)$ = 114 Hz, 2B, B6,9), −22.9 (d, $^1J(^{11}B\text{-}^1H)$ = 136 Hz, 2B, 1B, B1), −7.6 (s broad, 2B, B2,4), −4.9 (s broad, 2B, B7,8) ppm. Single crystalline material was obtained from saturated acetonitrile solution.

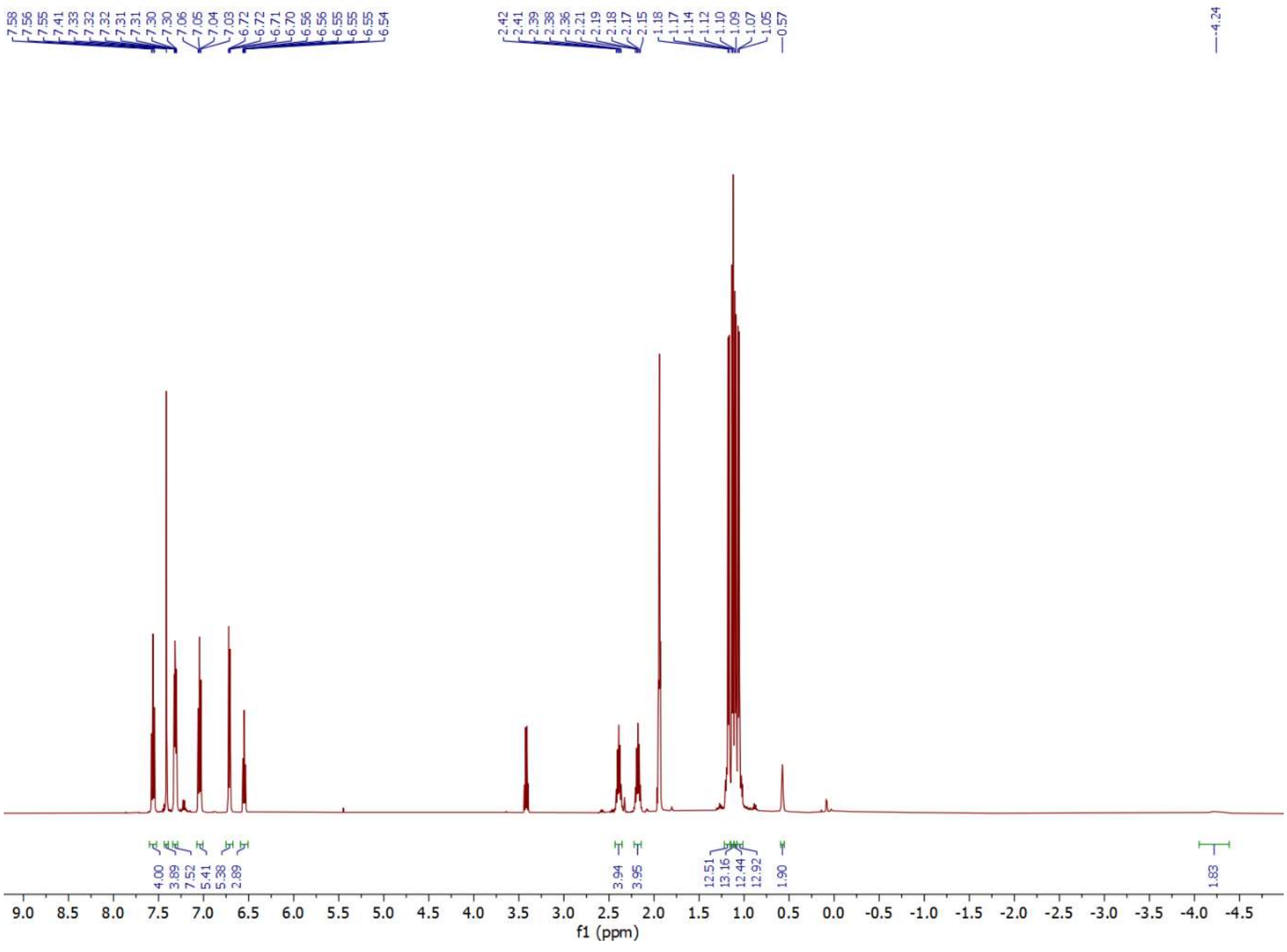

Supplementary Figure S19. The ^{1}H (500.1 MHz) NMR spectrum of *o*-2e in CD_3CN.

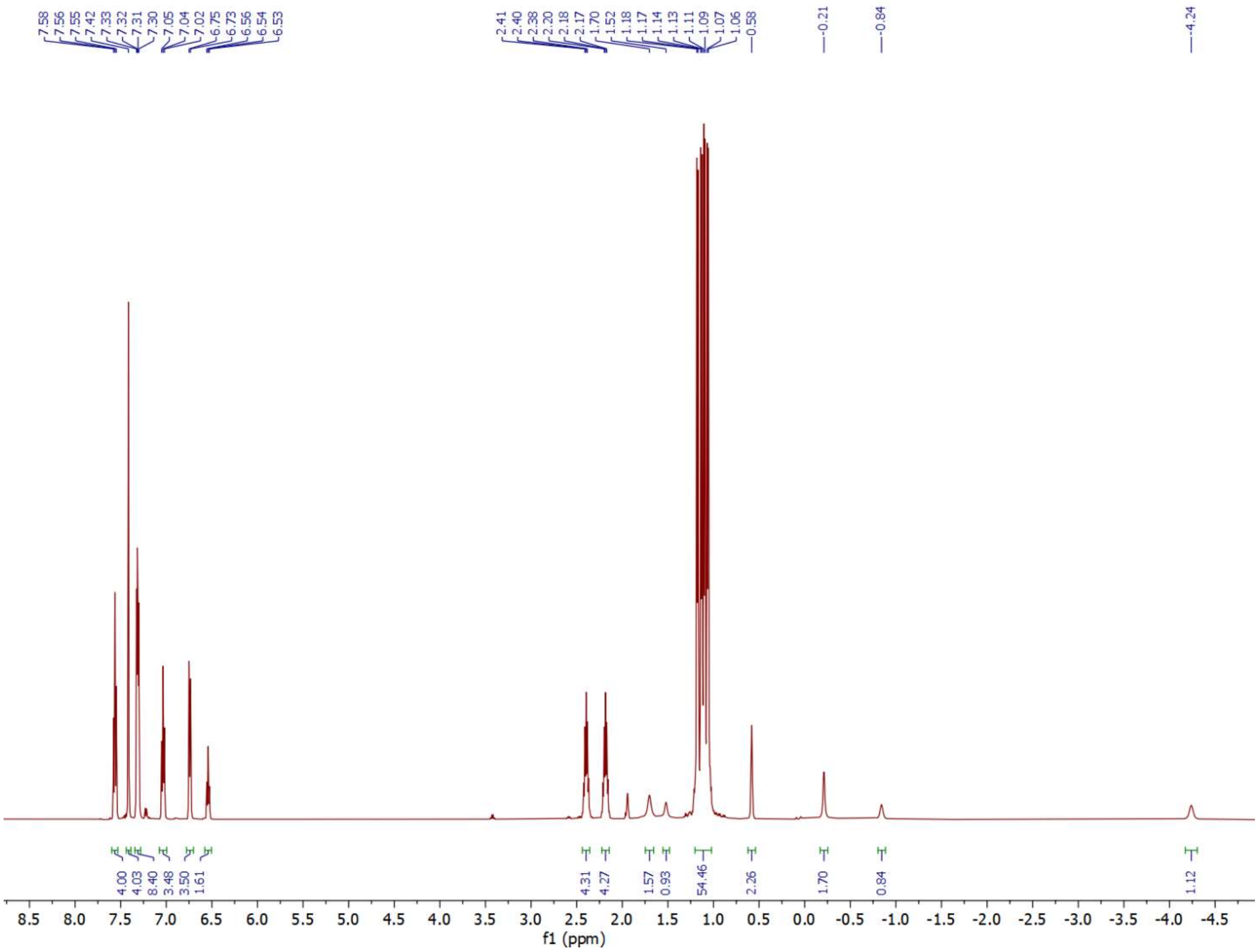

Supplementary Figure S20. The $^{1}H\{^{11}B\}$ (500.1 MHz) NMR spectrum of *o*-2e in CD_3CN.

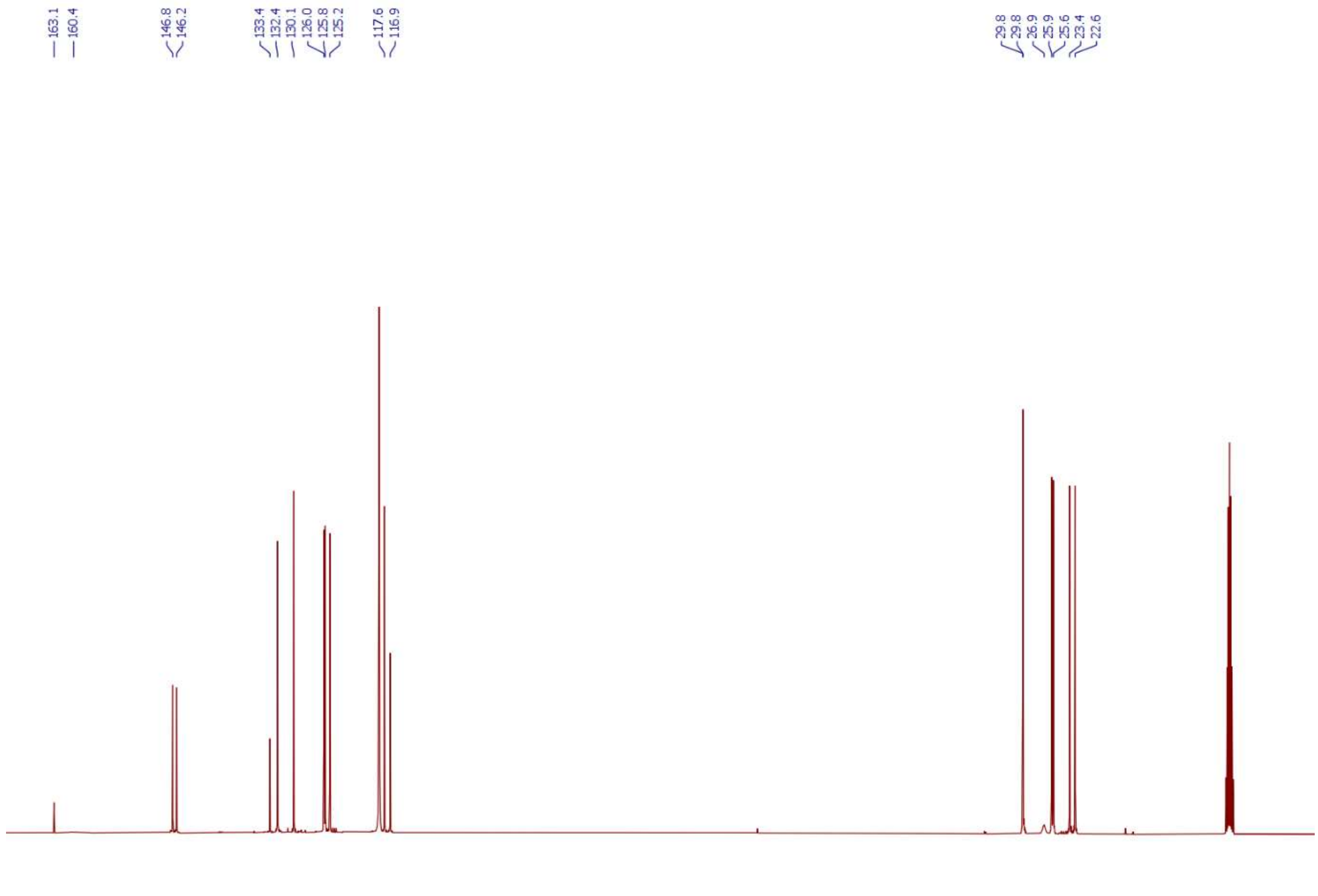

Supplementary Figure S21. The $^{13}C\{^{1}H\}$ (125.8 MHz) NMR spectrum of *o*-2e in CD_3CN.

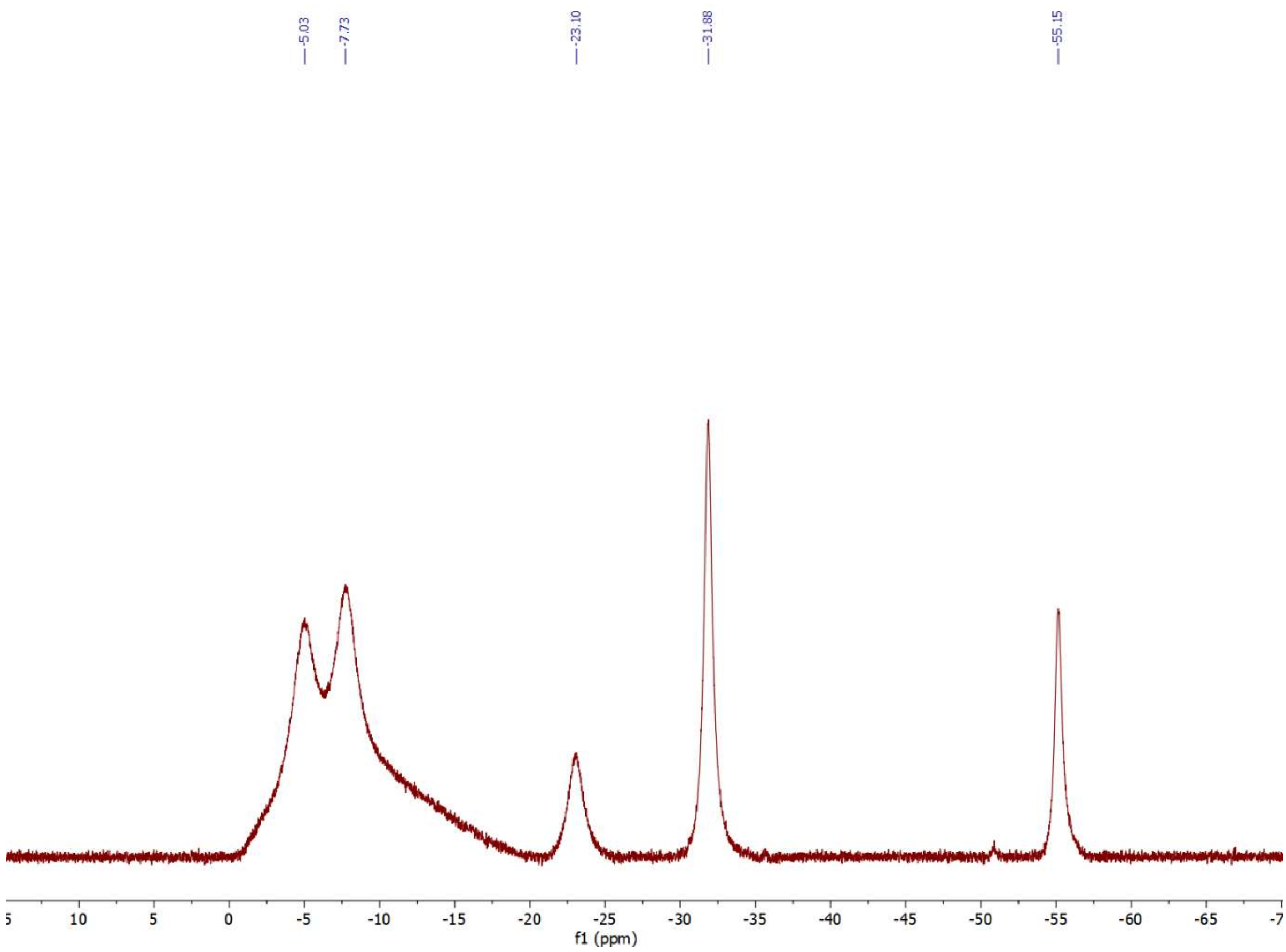

Supplementary Figure S22. The $^{11}B\{^{1}H\}$ (160.4) NMR spectrum of *o*-2e in CD_3CN.

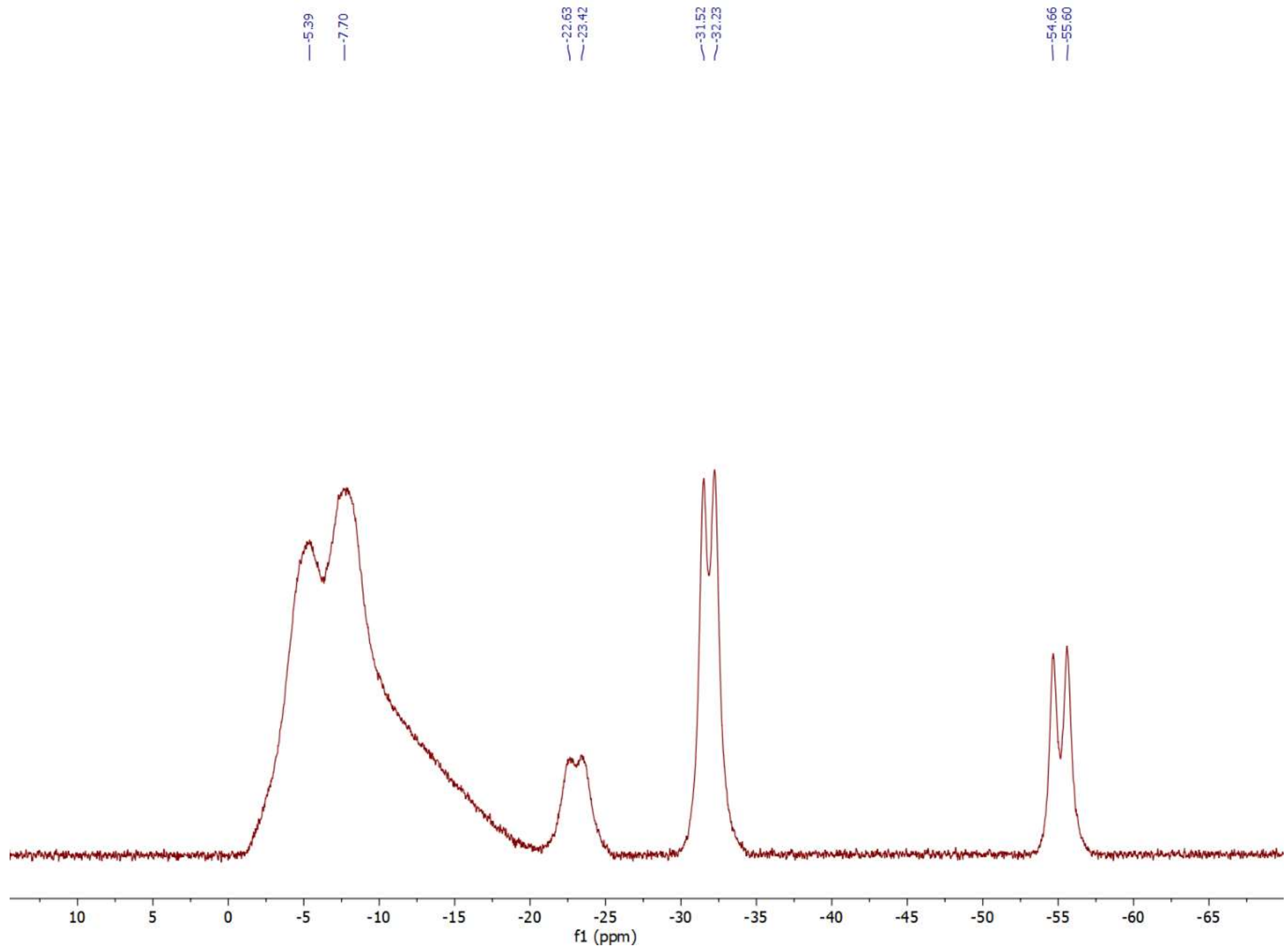

Supplementary Figure S23. The ^{11}B (160.4) NMR spectrum of *o*-2e in CD_3CN.

Synthesis of $[6,9\text{-}IPr_2\text{-}5,10\text{-}C_2B_8H_{11}]^+[NCCHCN]^-$ (*o*-2f)

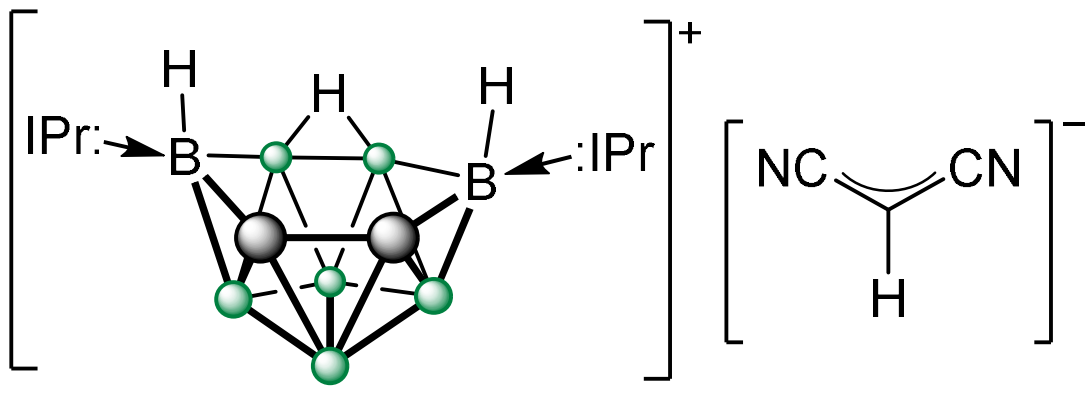

Solution of malononitrile (12 mg, 0.18 mmol) in diethyl ether (5 mL) was added to a stirred solution of *o*-**2** (150 mg, 0.17 mmol) in diethyl ether (5 mL) at room temperature. The reaction mixture was stirred for 30 minutes and filtrated. The solid was washed with diethyl ether (15 mL) and the resulting colourless powder of *o*-**2f** was dried in vacuo. Yield 150 mg, 93%. Dp 205 °C. **^{1}H NMR** (25 °C, DCM-D_2, 500 MHz): δ = −4.25 (s broad, 1H, B*H*B), −0.85 (s, 1H, B3), −0.21 (s, 2H, B6,9), 0.57 (s, 2H, BC*H*), 1.08 (d, $^3J(^1H\text{-}^1H)$ = 6.8 Hz, 14H, CH(C*H*$_3$)$_2$ + B7,8), 1.12 (d, $^3J(^1H\text{-}^1H)$ = 6.8 Hz, 12H, CH(C*H*$_3$)$_2$), 1.13 (d, $^3J(^1H\text{-}^1H)$ = 6.8 Hz, 12H, CH(C*H*$_3$)$_2$), 1.18 (d, $^3J(^1H\text{-}^1H)$ = 6.8 Hz, 12H, CH(C*H*$_3$)$_2$), 1.51 (s, 1H, B1), 1.70 (s, 2H, B2,4), 2.18 (sept, $^3J(^1H\text{-}^1H)$ = 6.9 Hz, 4H, C*H*(CH$_3$)$_2$), 2.39 (sept, $^3J(^1H\text{-}^1H)$ = 6.8 Hz, 4H, C*H*(CH$_3$)$_2$), 7.32 (d, $^3J(^1H\text{-}^1H)$ = 7.9 Hz, 8H, *m*-C$_6$*H*$_3$), 7.42 (s, 4H, NC*H*), 7.56 (t, $^3J(^1H\text{-}^1H)$ = 7.9 Hz, 4H, *p*-C$_6$*H*$_3$) ppm, signal for NCC*H* not observed. **^{13}C{^{1}H} NMR** (25 °C, DCM-D_2, 125.76 Hz): δ = -1.7 (s broad, NC*C*H), 22.6 (s, CH(*C*H$_3$)$_2$), 23.4 (s, CH(*C*H$_3$)$_2$), 25.6 (s, CH(*C*H$_3$)$_2$), 25.9 (s, CH(*C*H$_3$)$_2$), 26.9 (s broad, B*C*H), 29.8 (s, *C*H(CH$_3$)$_2$), 125.1 (s, *m*-*C*$_6$H$_3$), 125.8 (s, N*C*H), 126.0 (s, N*C*H), 131.7 (s broad, N*C*CH), 132.3 (s, *p*-*C*$_6$H$_3$), 133.4 (s, *ipso*-*C*$_6$H$_3$), 146.2 (s, *o*-*C*$_6$H$_3$), 146.7 (s, *o*-*C*$_6$H$_3$), 160.1 (s broad, B*C*N) ppm. **^{11}B NMR** (25 °C, CD_3CN, 160.42 MHz): δ = −55.1 (d, $^1J(^{11}B\text{-}^1H)$ = 145 Hz, 1B, B3), −31.8 (d, $^1J(^{11}B\text{-}^1H)$ = 112 Hz, 2B, B6,9), −23.0 (s broad, 1B, B1), −7.6 (s broad, 2B, B2,4), −5.0 (s broad, 2B, B7,8) ppm.

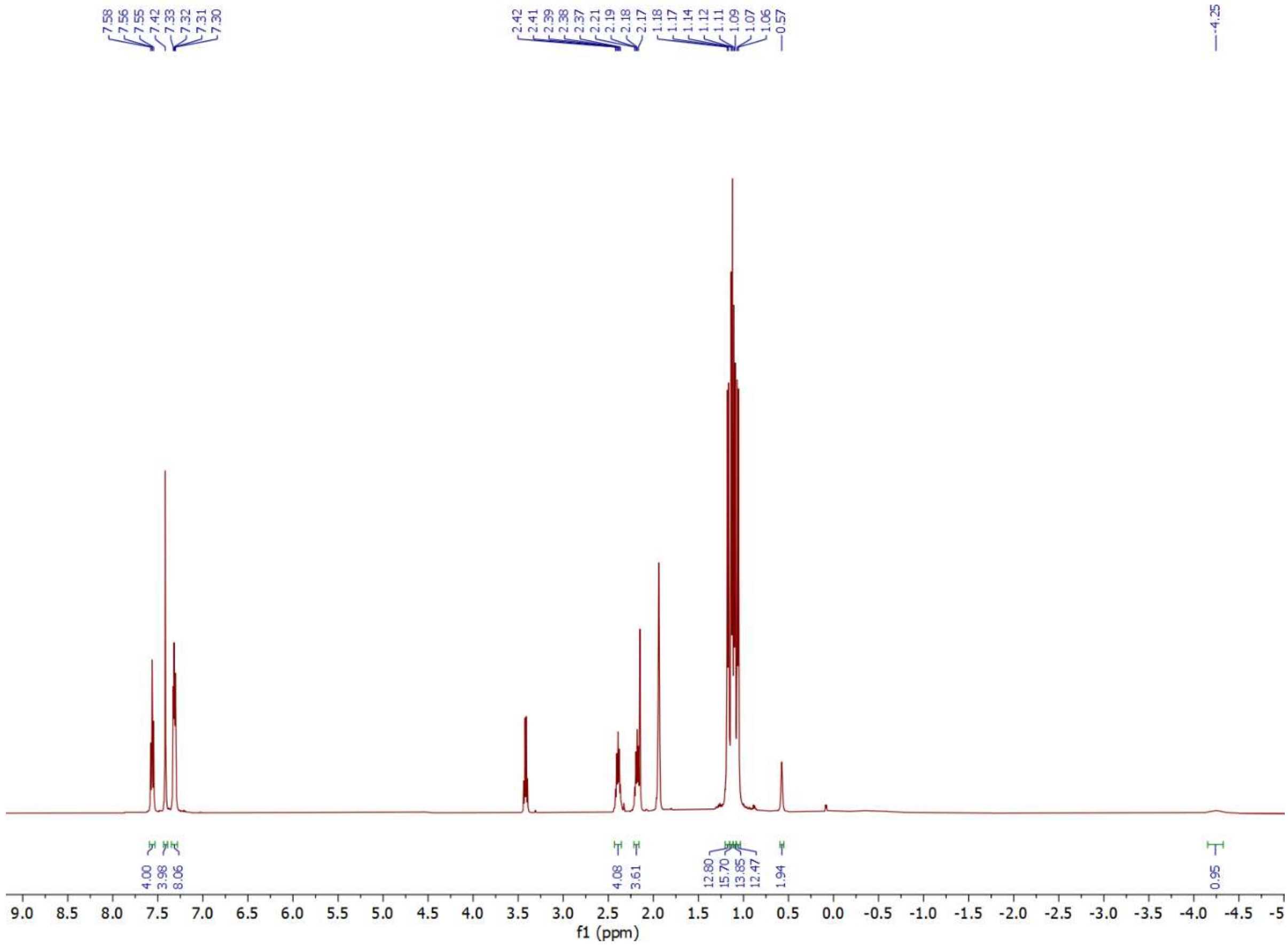

Supplementary Figure S24. The ^{1}H (500.1 MHz) NMR spectrum of *o*-2f in CD_3CN.

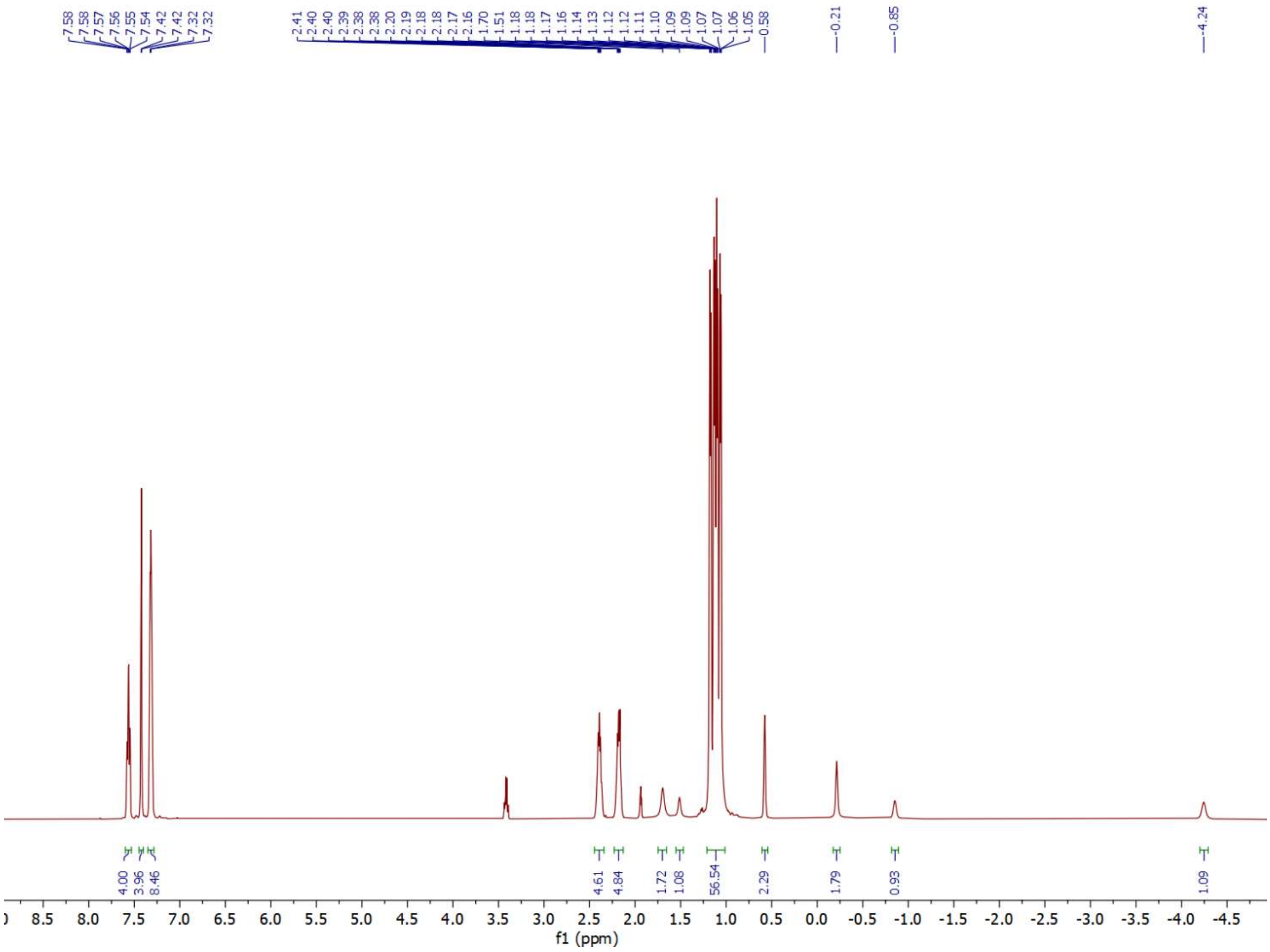

Supplementary Figure S25. The $^{1}H\{^{11}B\}$ (500.1 MHz) NMR spectrum of *o*-2f in CD_3CN.

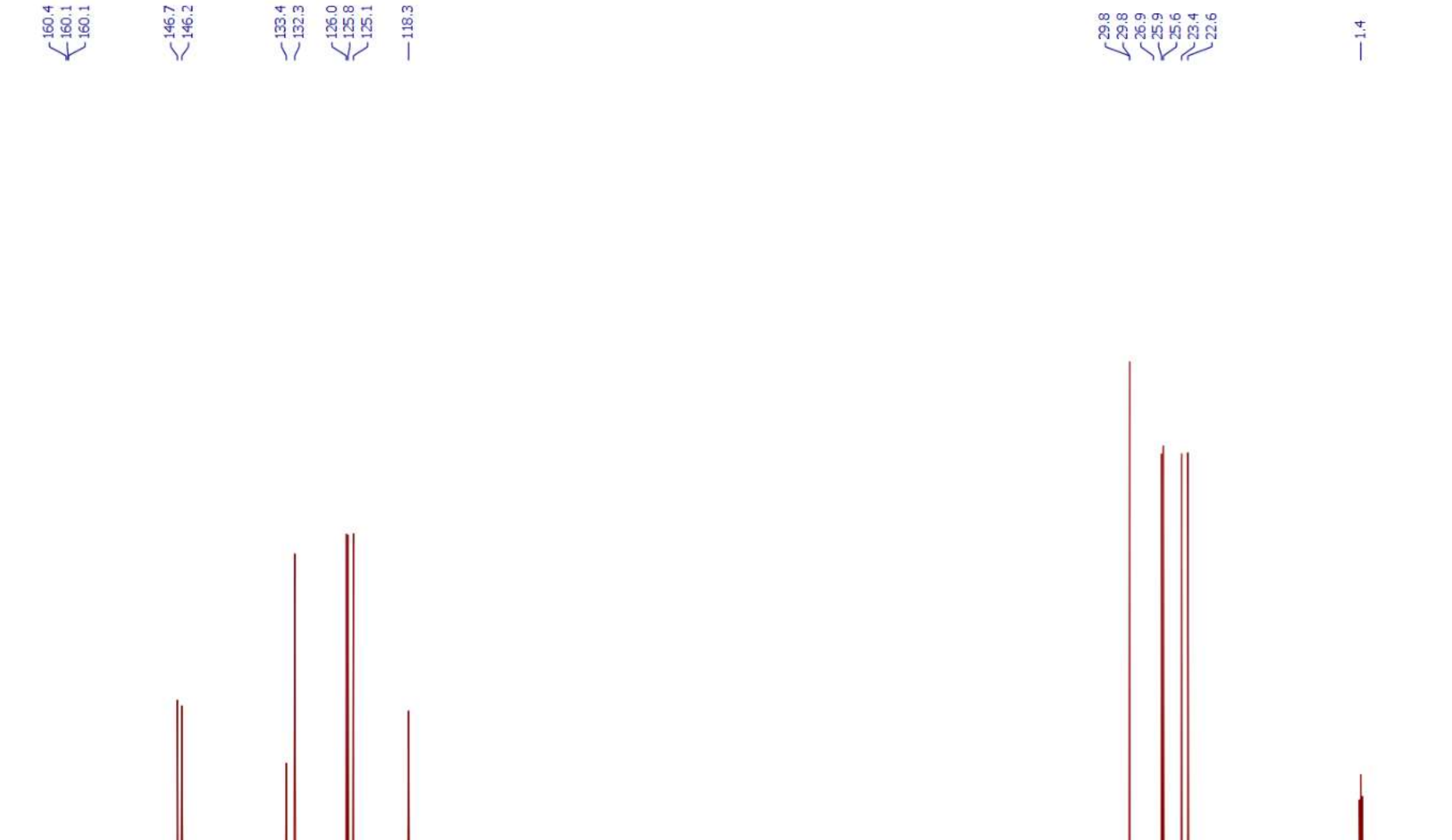

Supplementary Figure S26. The $^{13}C\{^{1}H\}$ (125.8 MHz) NMR spectrum of *o*-2f in CD_3CN.

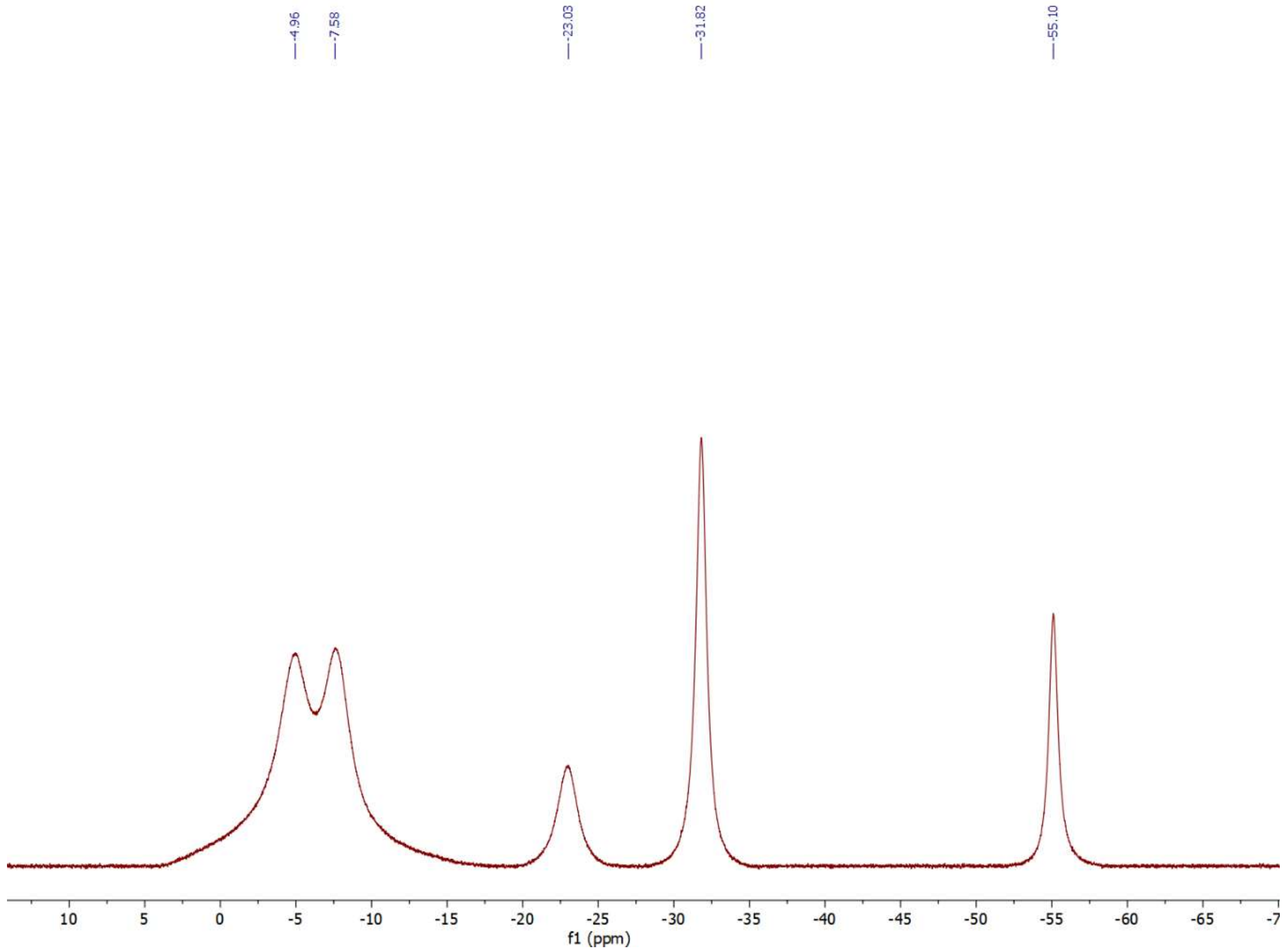

Supplementary Figure S27. The $^{11}B\{^{1}H\}$ (160.4) NMR spectrum of *o*-2f in CD_3CN.

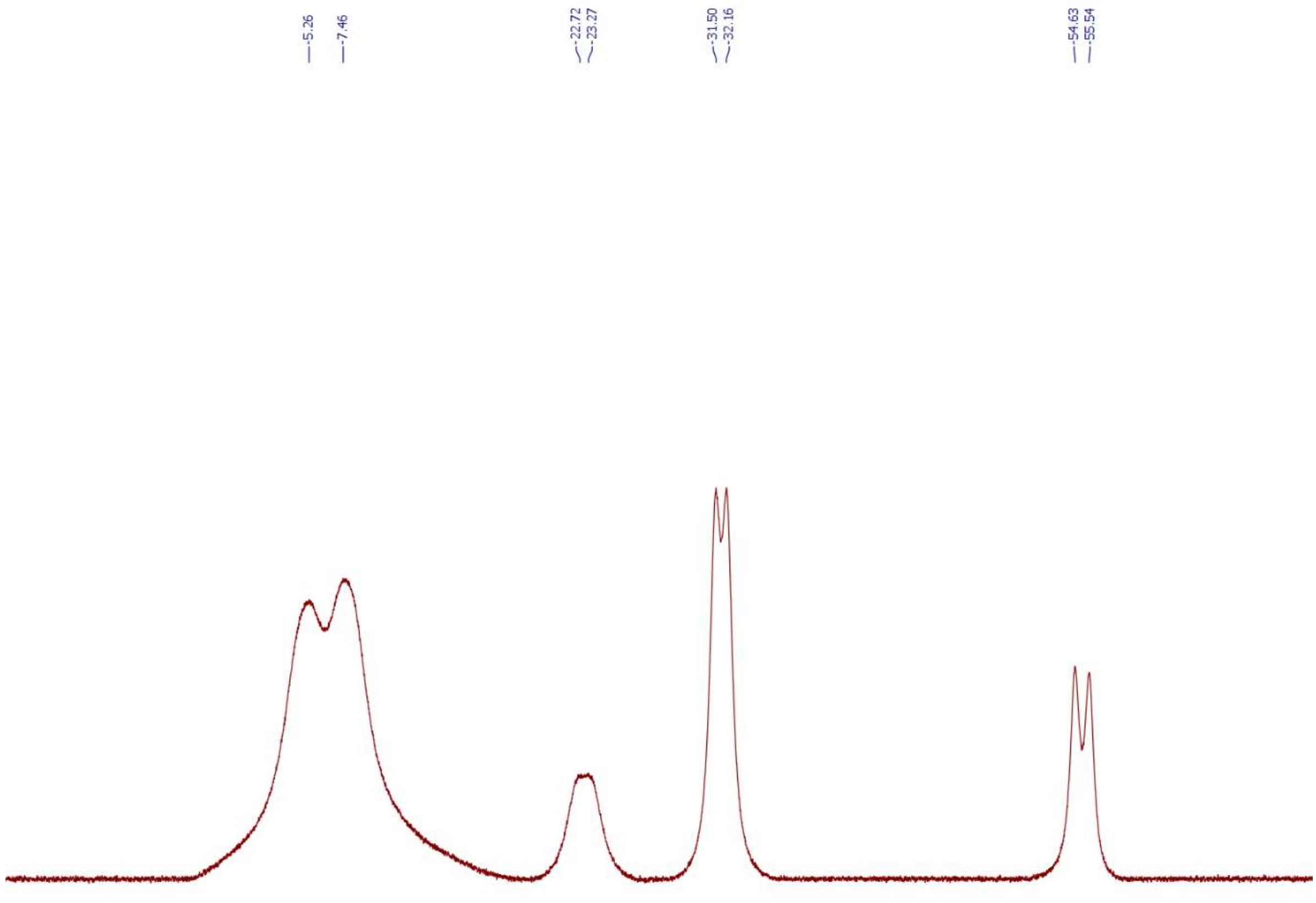

Supplementary Figure S28. The ^{11}B (160.4) NMR spectrum of *o*-2f in CD_3CN.

Synthesis of [6,9-IPr$_2$-5,10-C$_2$B$_8$H$_{11}$]$^+$[NCC(=C(NH$_2$)CHCN)CN]$^-$ (*o*-2f')

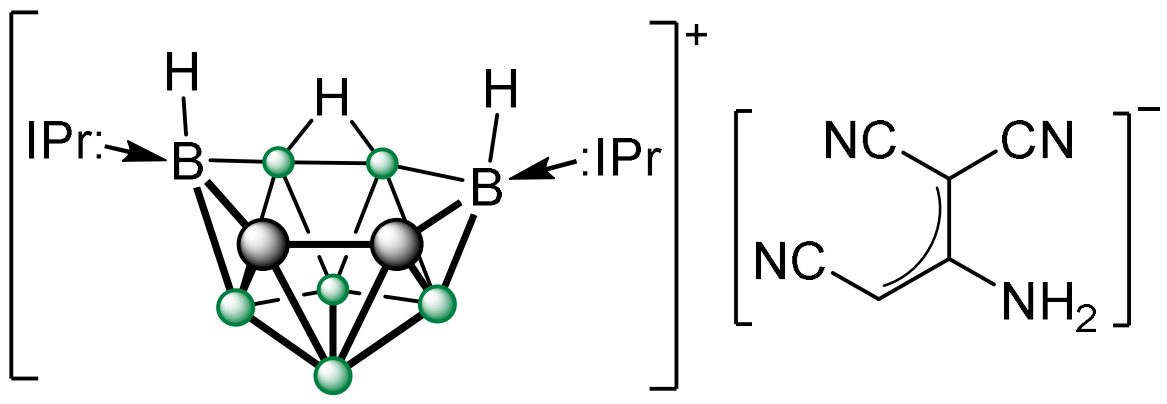

Solution of malononitrile (24 mg, 0.36 mmol) in 1,2-difluorobenzene (5 mL) was added to a stirred solution of *o*-**2** (148 mg, 0.16 mmol) in 1,2-difluorobenzene (5 mL) at room temperature. The reaction mixture was stirred overnight, and the solvent was removed in vacuo. The solid was washed twice with diethyl ether (15 mL) and the resulting colourless powder of *o*-**2f'** was dried in vacuo. The crystals suitable for X-ray diffraction analysis were grown by slow evaporation of DCM/hexane solution of *o*-**2f'** at room temperature. Yield 116 mg, 69%, **Dp** 206 °C. **^{1}H NMR** (25 °C, CD$_3$CN, 500 MHz): δ = −4.18 (s broad, 1H, B*H*B), −0.85 (s, 1H, B3), −0.21 (s, 2H, B6,9), 0.60 (s, 2H, BC*H*), 1.08 (d, ^3J(^{1}H-^{1}H) = 6.8 Hz, 14H, CH(C*H*$_3$)$_2$ + B7,8), 1.12 (d, ^3J(^{1}H-^{1}H) = 6.8 Hz, 12H, CH(C*H*$_3$)$_2$), 1.15 (d, ^3J(^{1}H-^{1}H) = 6.8 Hz, 12H, CH(C*H*$_3$)$_2$), 1.19 (d, ^3J(^{1}H-^{1}H) = 6.8 Hz, 12H, CH(C*H*$_3$)$_2$), 1.51 (s, 1H, B1), 1.70 (s, 2H, B2,4), 2.18 (sept, ^3J(^{1}H-^{1}H) = 6.8 Hz, 4H, C*H*(CH$_3$)$_2$), 2.38 (sept, ^3J(^{1}H-^{1}H) = 6.8 Hz, 4H, C*H*(CH$_3$)$_2$), 3.31 (s, 1H, C*H*), 4.54 (s, 2H, N*H*$_2$), 7.27 (d, ^3J(^{1}H-^{1}H) = 7.8 Hz, 8H, *m*-C$_6$*H*$_3$), 7.43 (s, 4H, NC*H*), 7.55 (t, ^3J(^{1}H-^{1}H) = 7.7 Hz, 4H, *p*-C$_6$*H*$_3$) ppm. **^{13}C{^{1}H} NMR** (25 °C, CD$_3$CN, 125.76 Hz): δ = 22.6 (s, CH(*C*H$_3$)$_2$), 23.3 (s, CH(CH$_3$)$_2$), 25.9 (s, CH(*C*H$_3$)$_2$), 26.1 (s, CH(*C*H$_3$)$_2$), 26.5 (s broad, B*C*H), 29.5 (s, *C*H(CH$_3$)$_2$), 46.6 (s, NC*C*H), 123.0 (s, *C*≡N), 124.6 (s, *C*≡N), 124.8 (s, N*C*H), 125.0 (s, N*C*H), 125.3 (s, *m*-*C*$_6$H$_3$), 132.0 (s, *p*-*C*$_6$H$_3$), 132.8 (s, *ipso*-*C*$_6$H$_3$), 145.6 (s, *o*-*C*$_6$H$_3$), 146.1 (s, *o*-*C*$_6$H$_3$), 160.3 (s broad, B*C*N), 165.0 (s, NC*C*) ppm. **^{11}B NMR** (25 °C, CD$_3$CN, 160.42 MHz): δ = −54.9 (d, ^1J(^{11}B-^{1}H) = 145 Hz, 1B, B3), −31.7 (d, ^1J(^{11}B-^{1}H) = 112 Hz, 2B, B6,9), −22.7 (s broad, 1B, B1), −7.2 (s broad, 2B, B2,4), −4.7 (s broad, 2B, B7,8) ppm.

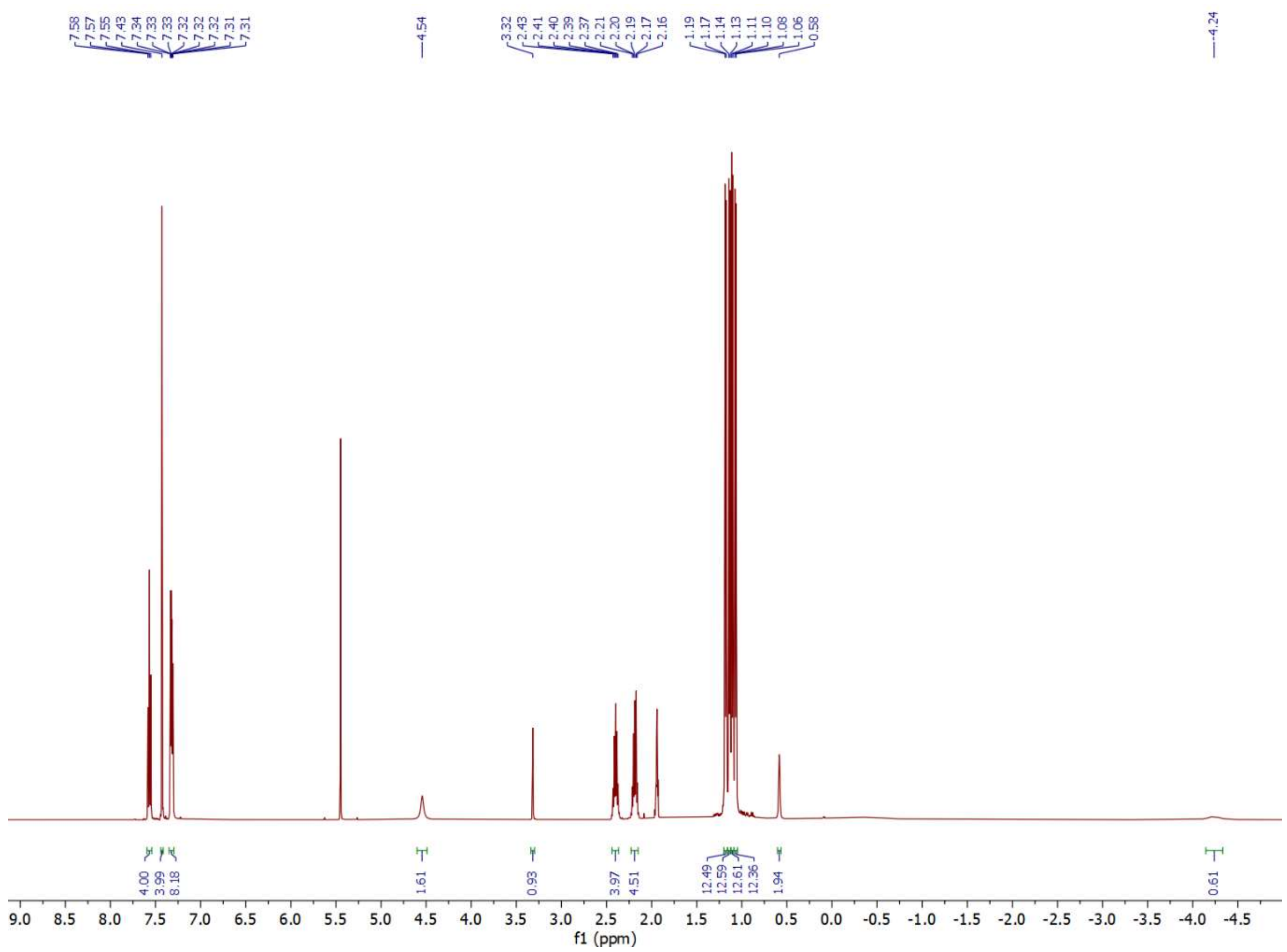

Supplementary Figure S29. The ^{1}H (500.1 MHz) NMR spectrum of *o*-2f' in CD_3CN.

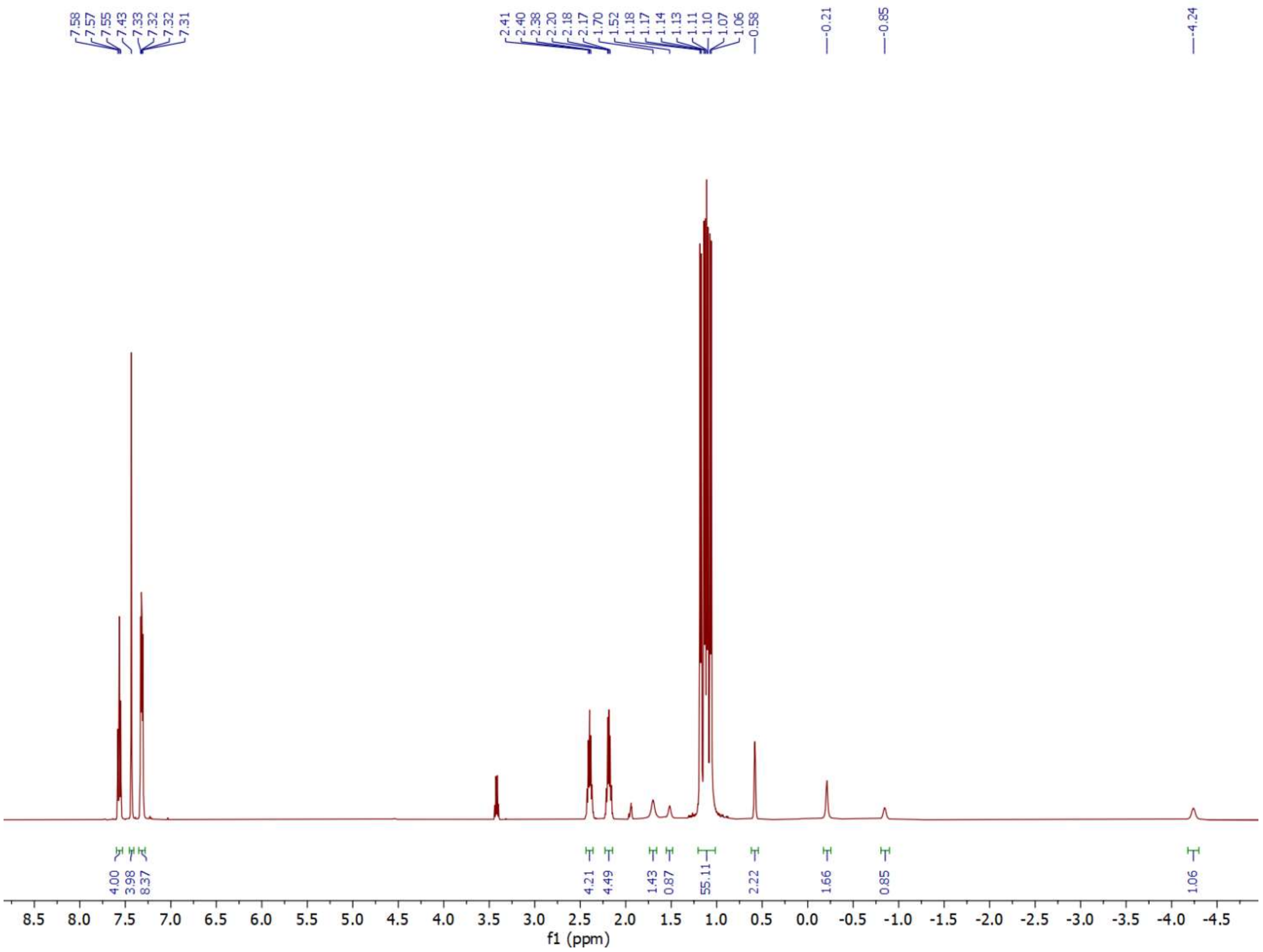

Supplementary Figure S30. The $^{1}H\{^{11}B\}$ (500.1 MHz) NMR spectrum of *o*-2f' in CD_3CN.

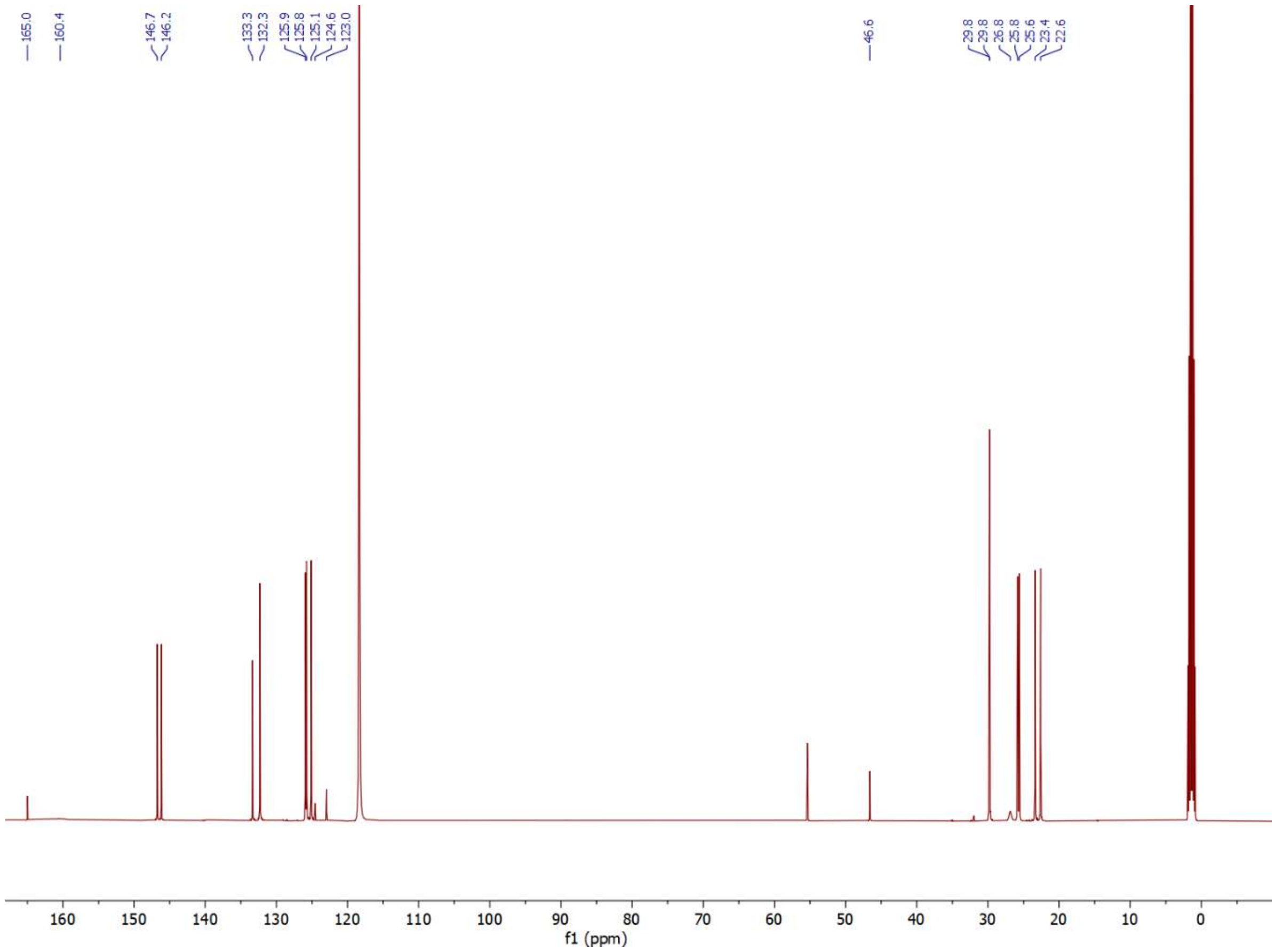

Supplementary Figure S31. The $^{13}C\{^{1}H\}$ (125.8 MHz) NMR spectrum of *o*-2f' in CD_3CN.

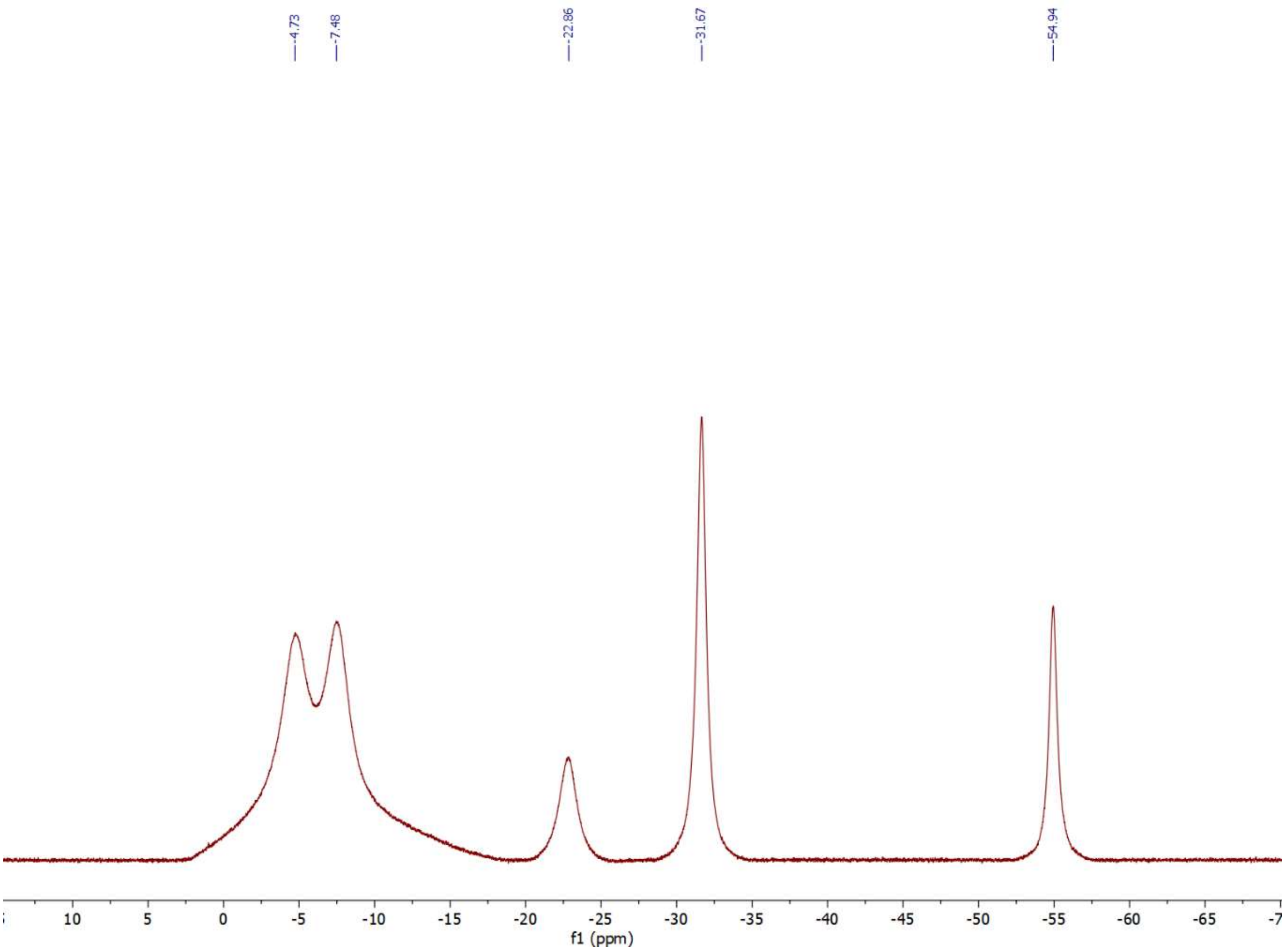

Supplementary Figure S32. The $^{11}B\{^{1}H\}$ (160.4) NMR spectrum of *o*-2f' in CD_3CN.

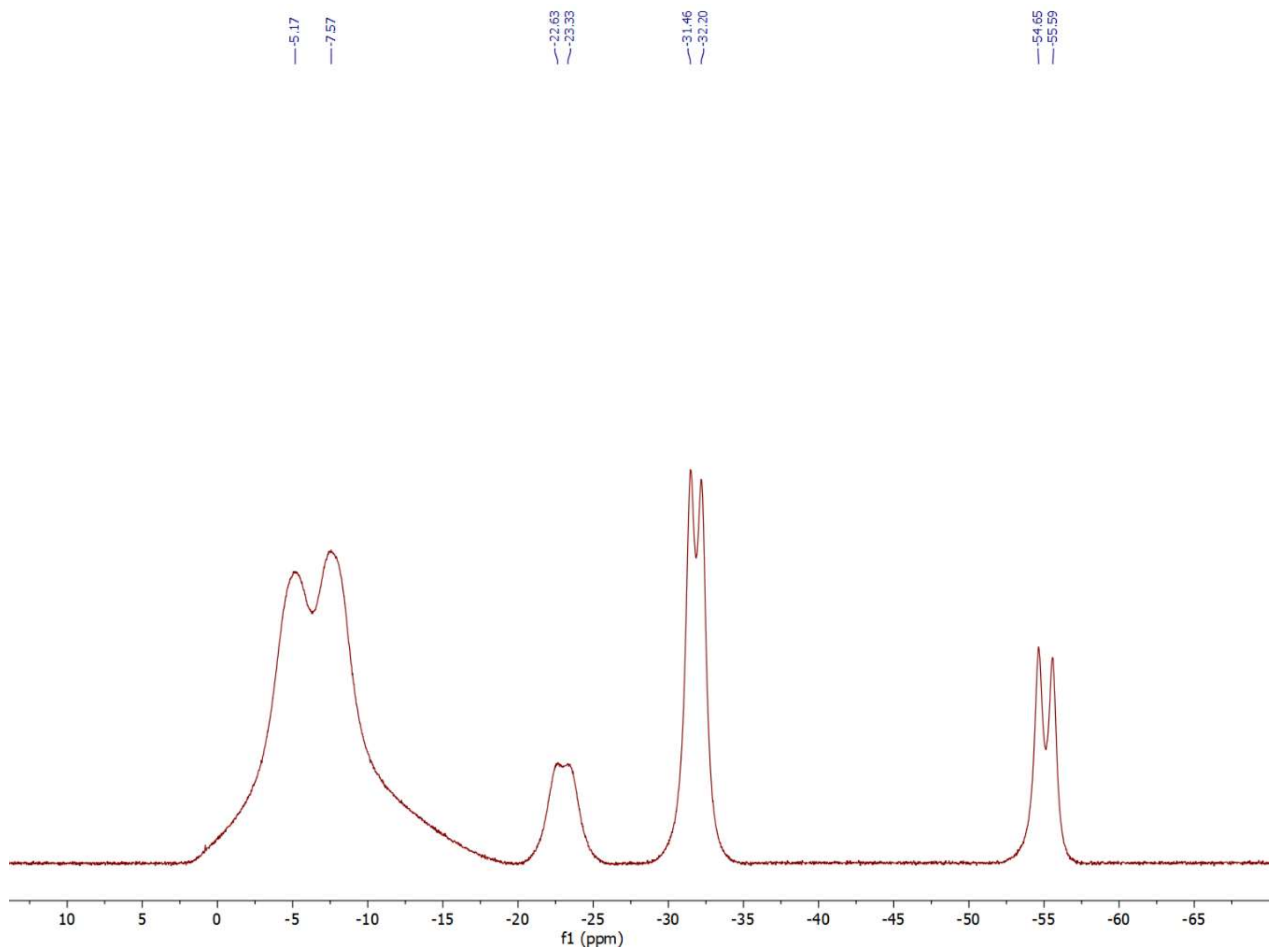

Supplementary Figure S33. The ^{11}B (160.4) NMR spectrum of *o*-2f' in CD_3CN.

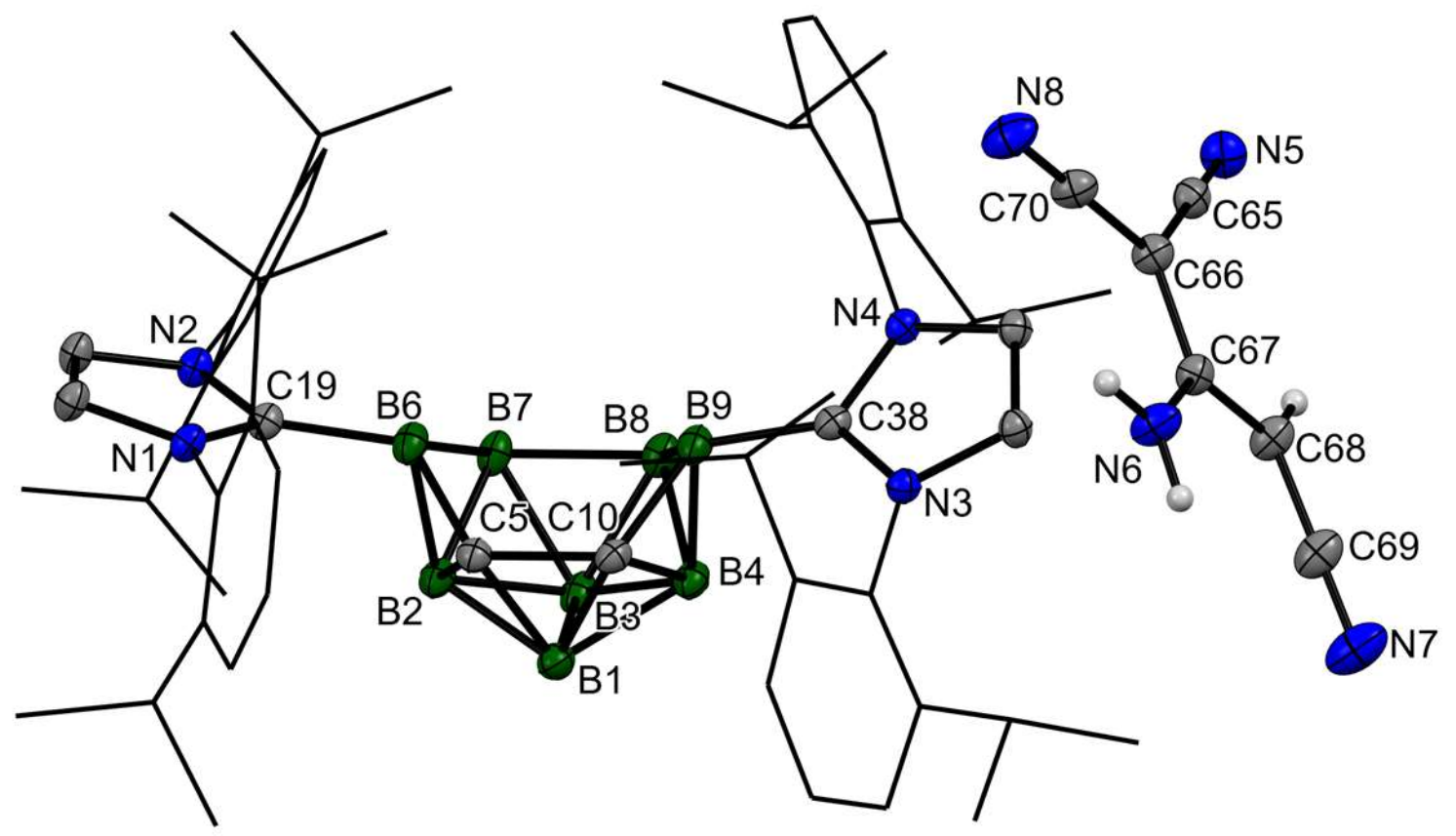

Supplementary Figure S34. The molecular structure of *o*-2f'. ORTEP view, 30% probability level; solvent molecules (dichloromethane) have been omitted for clarity. Selected interatomic distances [Å]: B9-C10 1.594(4), C5-C10 1.540(3), C5-B6 1.594(4), B6-B7 2.022(4), B7-B8 1.842(4), B8-B9 2.020(4), C65-C66 1.408(3), C66-C67 1.226(3), C66-C67 1.437(3), C67-C68 1.379(3), C68-C69 1.407(3), C65-N5 1.158(3), C69-N7 1.152(3), C70-N8 1.153(3), C67-N6 1.355(2).

Supplementary Table S3. Experimental details of *o*-2f'.

Crystal data	
Chemical formula	$C_{56}H_{83}B_8N_4·C_6H_3N_4·2(CH_2Cl_2)$
M_r	1199.71
Crystal system, space group	Triclinic, *P*-1
Temperature (K)	150
a, *b*, *c* (Å)	11.5613(8), 14.7822(11), 20.6455(14)
α, β, γ (°)	92.812(3), 104.083(2), 91.219(3)
V (Å^3)	3416.2(4)
Z	2
Radiation type	Mo*K*α
μ (mm^{-1})	0.22
Crystal size (mm)	0.52 × 0.27 × 0.12
Data collection	
Diffractometer	Bruker D8 - Venture
Absorption correction	Multi-scan<br>*SADABS2016*/2 - Bruker AXS area detector scaling and absorption correction Reference: Krause, L., Herbst-Irmer, R., Sheldrick G.M. & Stalke D., *J. Appl. Cryst.* 2015, 48, 3–10.
T_{min}, T_{max}	0.685, 0.746
No. of measured, independent and observed [$I > 2\sigma(I)$] reflections	180014, 16999, 11979
R_{int}	0.102
$(\sin \theta/\lambda)_{max}$ (Å^{-1})	0.668
Refinement	
$R[F^2 > 2\sigma(F^2)]$, $wR(F^2)$, S	0.057, 0.151, 1.01
No. of reflections	16999
No. of parameters	843
No. of restraints	797
H-atom treatment	H atoms treated by a mixture of independent and constrained refinement
$\Delta\rho_{max}$, $\Delta\rho_{min}$ (e Å^{-3})	0.44, −0.50

Supplementary Table S4. Experimental details of *o*-2f'.

Crystal data	
Chemical formula	$2(C_{56}H_{83}B_8N_4)\cdot 2(C_6H_3N_4)\cdot 3(C_6H_6)$
M_r	2294.05
Crystal system, space group	Monoclinic, $P2_1$
Temperature (K)	150
a, *b*, *c* (Å)	16.4150(4), 21.7082(6), 20.3370(5)
β (°)	103.615(1)
V (Å^3)	7043.2(3)
Z	2
Radiation type	Mo*K*α
μ (mm^{-1})	0.06
Crystal size (mm)	0.52 × 0.44 × 0.27
Data collection	
Diffractometer	Bruker D8 - Venture
Absorption correction	Multi-scan<br>*SADABS2016*/2 - Bruker AXS area detector scaling and absorption correction Reference: Krause, L., Herbst-Irmer, R., Sheldrick G.M. & Stalke D., *J. Appl. Cryst.* 2015, 48, 3–10.
T_{min}, T_{max}	0.644, 0.698
No. of measured, independent and observed [$I > 2\sigma(I)$] reflections	533155, 34879, 30573
R_{int}	0.087
$(\sin \theta/\lambda)_{max}$ (Å^{-1})	0.667
Refinement	
$R[F^2 > 2\sigma(F^2)]$, $wR(F^2)$, S	0.051, 0.146, 1.02
No. of reflections	34879
No. of parameters	1607
No. of restraints	1447
H-atom treatment	H atoms treated by a mixture of independent and constrained refinement
$\Delta\rho_{max}$, $\Delta\rho_{min}$ (e Å^{-3})	0.52, −0.50
Absolute structure	Flack x determined using 13545 quotients [(I+)-(I-)]/[(I+)+(I-)] (Parsons, Flack and Wagner, Acta Cryst. B69 (2013) 249-259).
Absolute structure parameter	0.3(3)

Synthesis of $[6,9\text{-}IPr_2\text{-}5,10\text{-}C_2B_8H_{11}]^+[2\text{-}(CH_2CO(C_8H_5O_2))C_6H_4O]^-$ (*o*-2g)

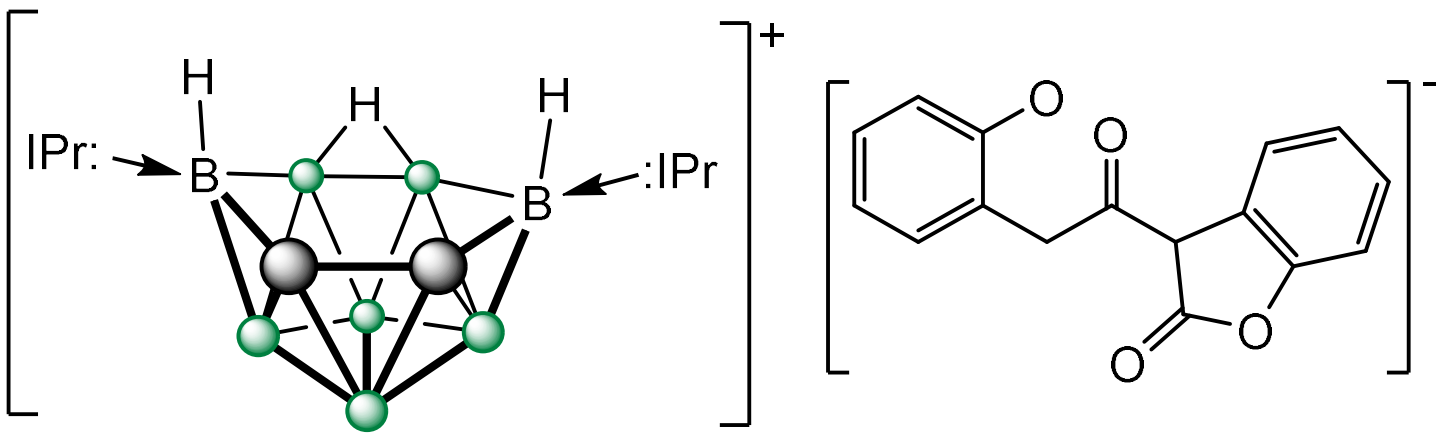

Solution of 2-coumaranone (45 mg, 0.33 mmol) in diethyl ether (5 mL) was added to a stirred solution of *o*-**2** (141 mg, 0.16 mmol) in diethyl ether (5 mL) at room temperature. The reaction mixture was stirred overnight and filtrated. The solid was washed with diethyl ether (15 mL) and the resulting colourless powder of *o*-**2g** was dried in vacuo. The crystals suitable for X-ray diffraction analysis were grown from a saturated solution of *o*-**2g** in methanol at 4 °C. Yield 154 mg, 84%. Dp 224 °C. **^{1}H NMR** (25 °C, CD_3CN, 500 MHz): δ = −4.20 (s broad, 1H, B*H*B), −0.75 (s, 1H, B3), −0.23 (s, 2H, B6,9), 0.58 (s, 2H, BC*H*), 1.07 (d, $^{3}J(^{1}H-^{1}H)$ = 6.8 Hz, 14H, $CH(CH_3)_2$ + B7,8), 1.11 (d, $^{3}J(^{1}H-^{1}H)$ = 6.8 Hz, 12H, $CH(CH_3)_2$), 1.13 (d, $^{3}J(^{1}H-^{1}H)$ = 6.8 Hz, 12H, $CH(CH_3)_2$), 1.18 (d, $^{3}J(^{1}H-^{1}H)$ = 6.8 Hz, 12H, $CH(CH_3)_2$), 1.58 (s, 1H, B1), 1.70 (s, 2H, B2,4), 2.17 (sept, $^{3}J(^{1}H-^{1}H)$ = 6.8 Hz, 4H, $CH(CH_3)_2$), 2.37 (sept, $^{3}J(^{1}H-^{1}H)$ = 6.7 Hz, 4H, $CH(CH_3)_2$), 4.32 (s, 2H, CH_2), 6.69 (t, $^{3}J(^{1}H-^{1}H)$ = 7.4 Hz, 1H, Ar*H*), 6.84 (m, 2H, Ar*H*), 6.89 (m, 1H, Ar*H*), 7.00 (t, $^{3}J(^{1}H-^{1}H)$ = 7.3 Hz, 1H, ArH), 7.12 (s, 4H, NC*H*), 7.25 (d, $^{3}J(^{1}H-^{1}H)$ = 7.8 Hz, 4H, *m*-C_6H_3), 7.26 (d, $^{3}J(^{1}H-^{1}H)$ = 7.8 Hz, 5H, *m*-C_6H_3 + Ar*H*), 7.29 (d, $^{3}J(^{1}H-^{1}H)$ = 7.4 Hz, 1H, Ar*H*), 7.53 (t, $^{3}J(^{1}H-^{1}H)$ = 7.8 Hz, 4H, *p*-C_6H_3), 7.78 (d, $^{3}J(^{1}H-^{1}H)$ = 7.4 Hz, 1H, Ar*H*) ppm. **$^{13}C\{^{1}H\}$ NMR** (25 °C, CD_3CN, 125.76 Hz): δ = 22.6 (s, $CH(CH_3)_2$), 23.3 (s, $CH(CH_3)_2$), 25.8 (s, $CH(CH_3)_2$), 26.1 (s, $CH(CH_3)_2$), 26.4 (s broad, BCH), 29.5 (s, $CH(CH_3)_2$), 39.6 (s, CH_2), 91.6 (s, Ar*C*), 107.9 (s, Ar*C*), 117.4 (s, Ar*C*), 119.5 (s, Ar*C*), 119.9 (s, Ar*C*), 121.1 (s, Ar*C*), 122.3 (s, Ar*C*), 124.8 (s, N*C*H), 125.0 (s, N*C*H), 125.3 (s, *m*-C_6H_3), 126.4 (s, Ar*C*), 127.8 (s, Ar*C*), 131.7 (s, Ar*C*6), 131.9 (s, *p*-C_6H_3), 132.7 (s, *ipso*-C_6H_3), 145.6 (s, *o*-C_6H_3), 146.1 (s, *o*-C_6H_3), 148.7 (s, Ar*C*), 158.2 (s, Ar*C*), 160.7 (s broad, B*C*N), 172.2 (s, Ar*C*), 189.3 (s, Ar*C*) ppm. **^{11}B NMR** (25 °C, CD_3CN, 160.42 MHz): δ = −55.0 (d, $^{1}J(^{11}B-^{1}H)$ = 148 Hz, 1B, B3), −31.9 (d, $^{1}J(^{11}B-^{1}H)$ = 113 Hz, 2B, B6.9), −22.7 (d, $^{1}J(^{11}B-^{1}H)$ = 155 Hz, 1B, B1), −7.4 (s broad, 2B, B2.4), −5.1 (s broad, 2B, B7.8) ppm.

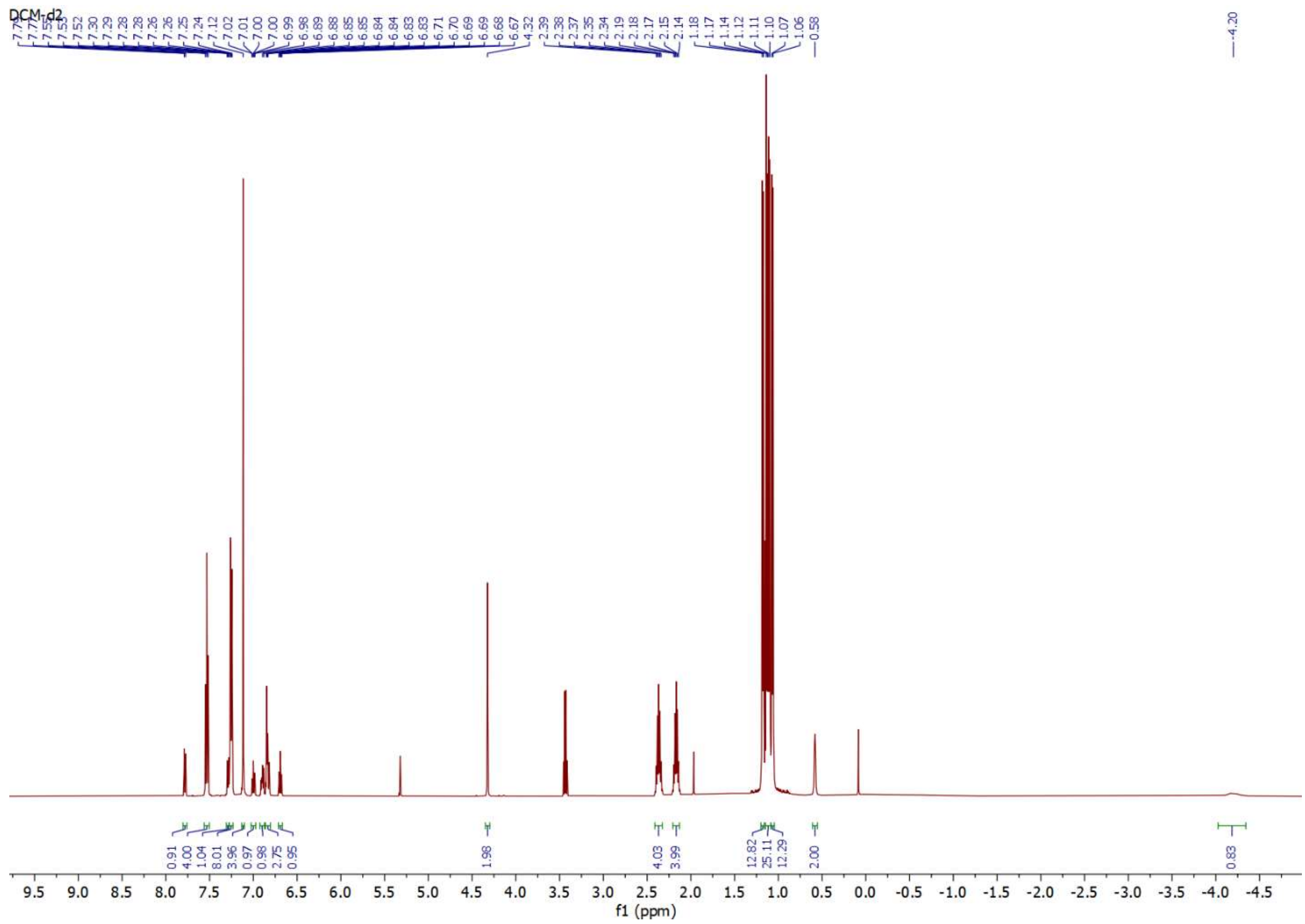

Supplementary Figure S35. The ^{1}H (500.1 MHz) NMR spectrum of *o*-2g in CD_2Cl_2.

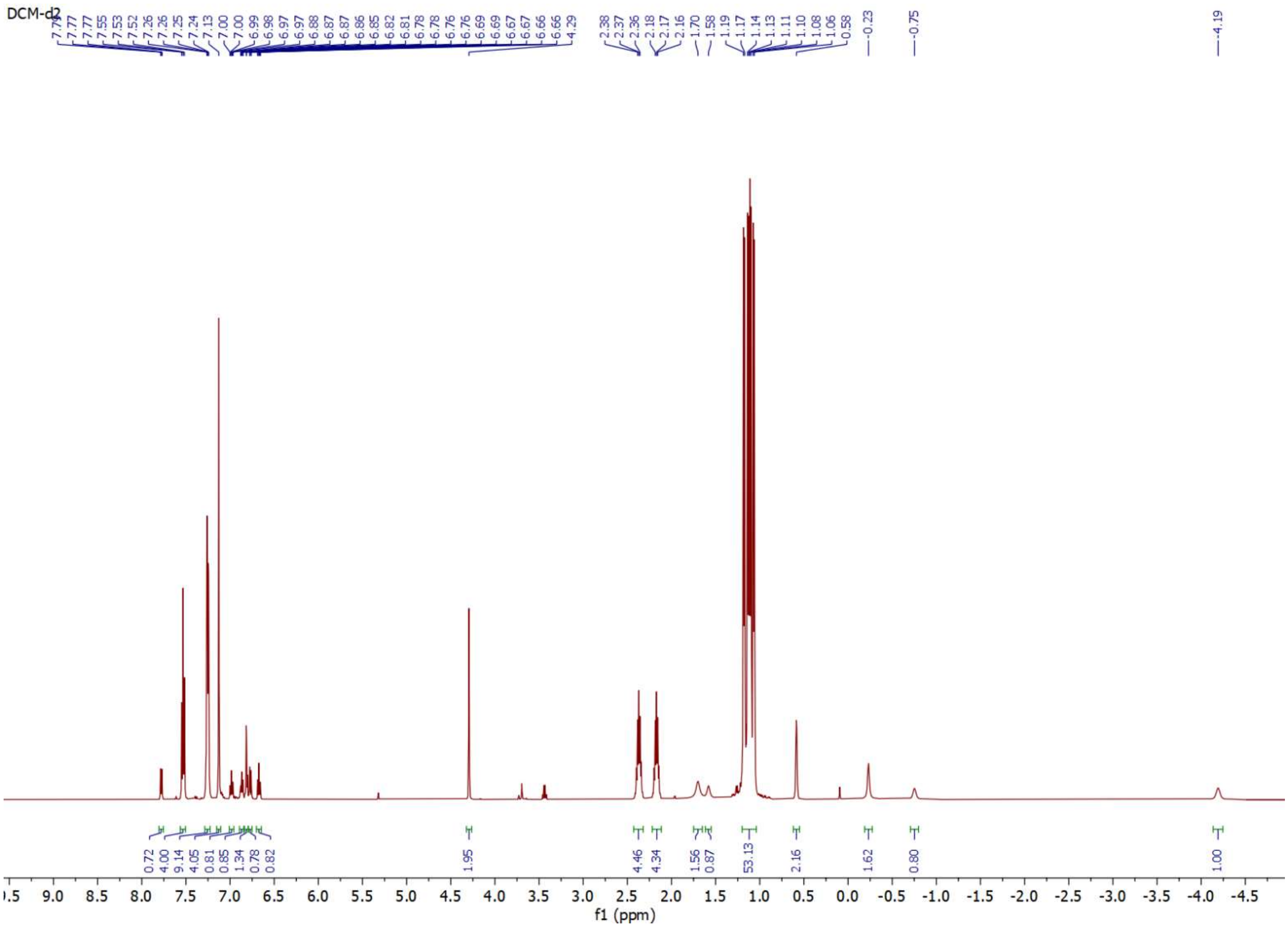

Supplementary Figure S36. The $^{1}H\{^{11}B\}$ (500.1 MHz) NMR spectrum of *o*-2g in CD_2Cl_2.

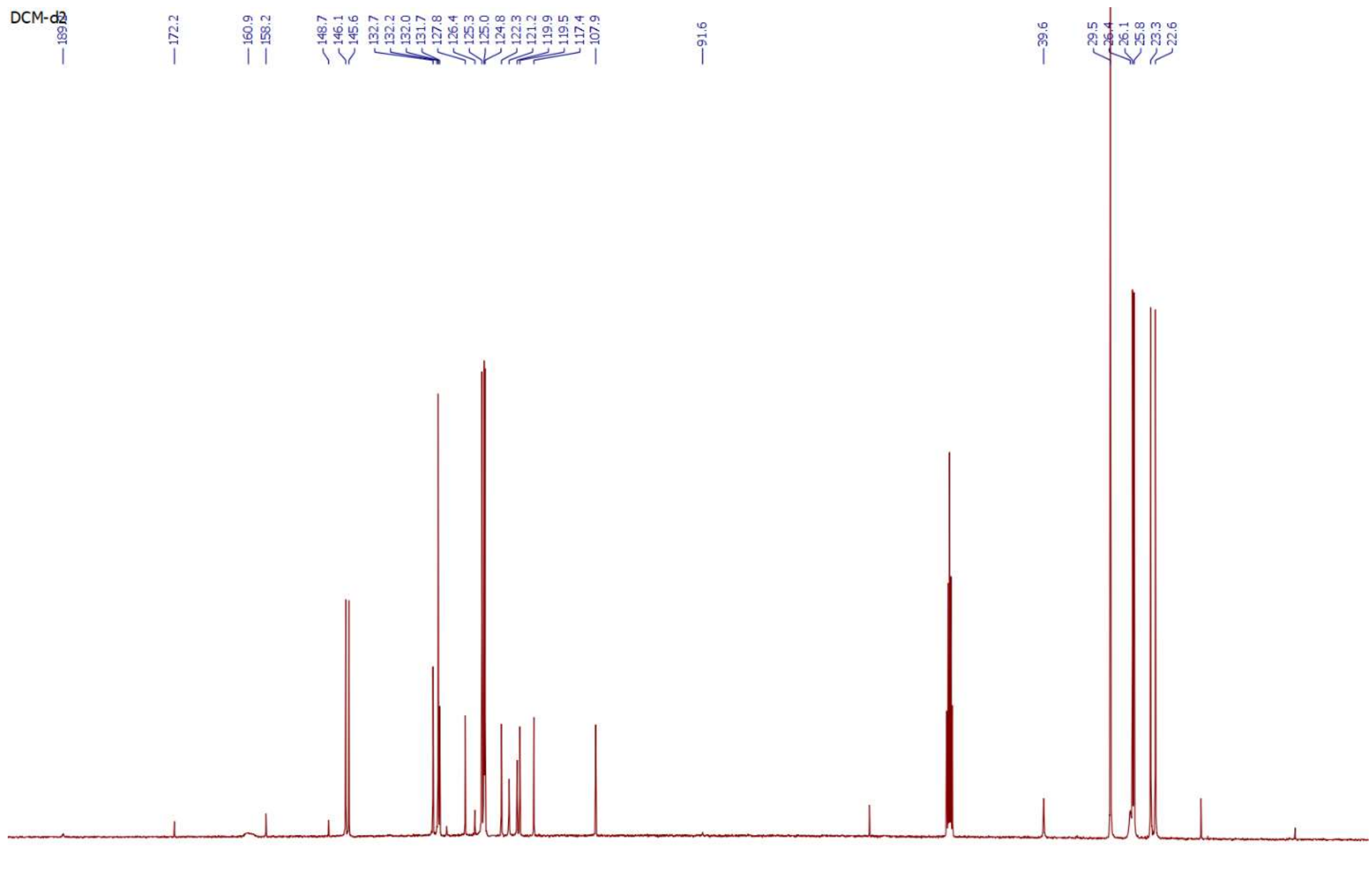

Supplementary Figure S37. The $^{13}C\{^{1}H\}$ (125.8 MHz) NMR spectrum of *o*-2g in CD_2Cl_2.

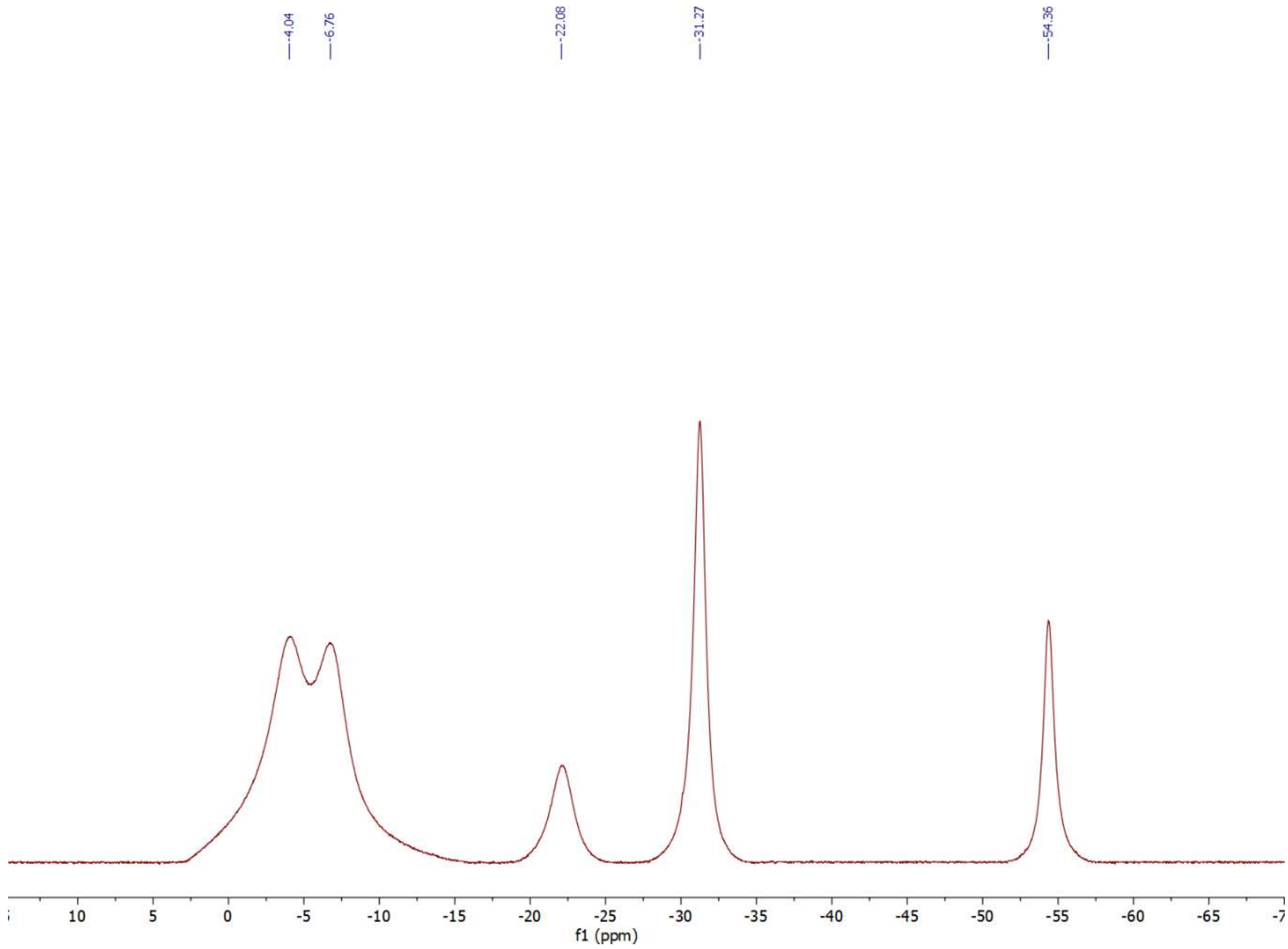

Supplementary Figure S38. The $^{11}B\{^1H\}$ (160.4) NMR spectrum of *o*-2g in CD_2Cl_2.

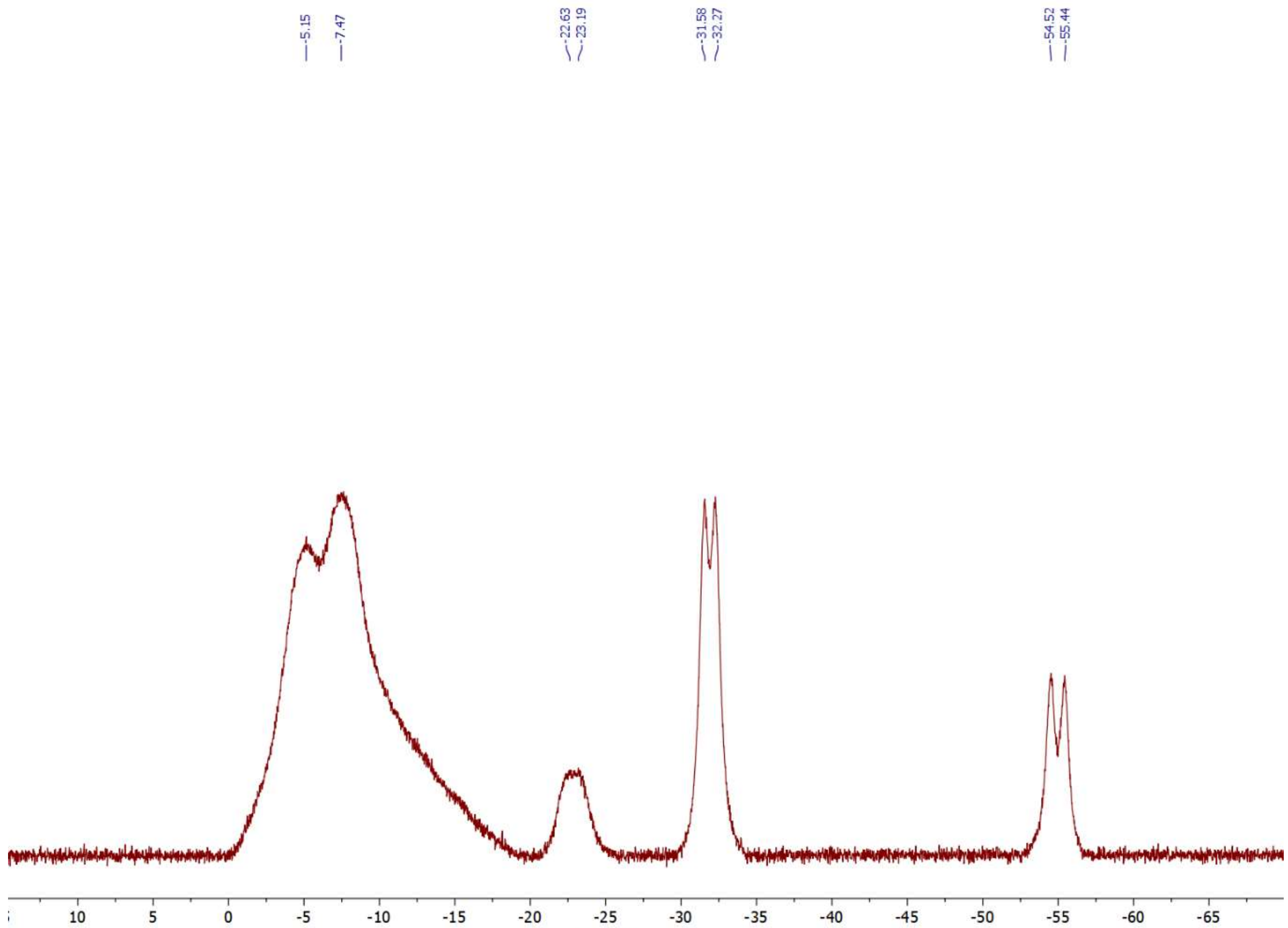

Supplementary Figure S39. The ^{11}B NMR (160.4) spectrum of *o*-2g in CD_2Cl_2.

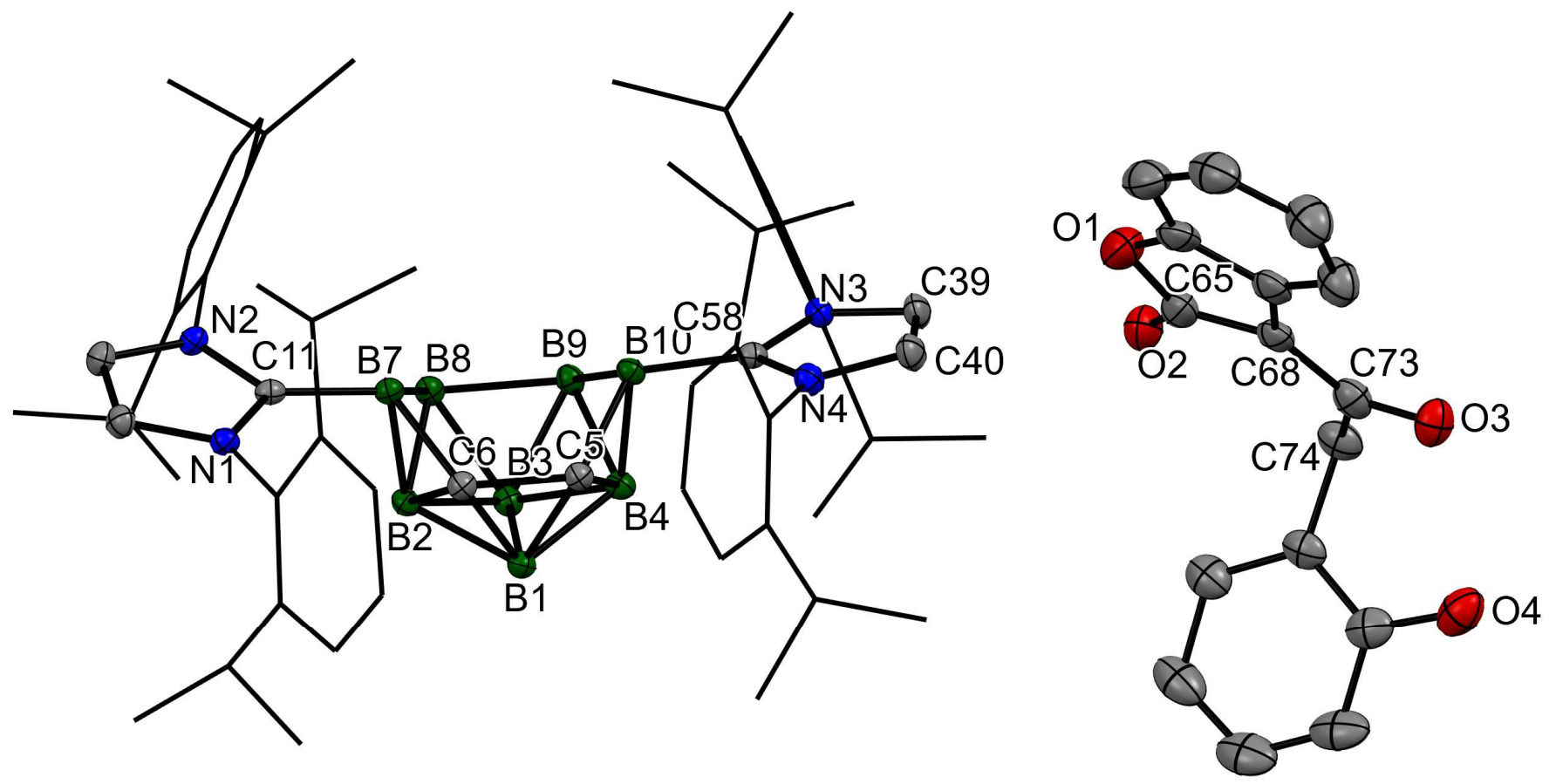

Supplementary Figure S40. The molecular structure of *o*-2g. ORTEP view, 30% probability level; solvent molecule (methanol) has been omitted for clarity. Selected interatomic distances [Å]: B7-C6 1.638(3), C5-C6 1.534(2), C5-B10 1.647(3), B7-B8 1.996(3), B8-B9 1.845(3), B9-B10 1.996(3), C65-C68 1.465(7), C68-C73 1.415(6), C73-C74 1.515(8), C65-O2 1.177(6), C73-O3 1.282(6).

Supplementary Table S5. Experimental details of *o*-2g.

Crystal data	
Chemical formula	$C_{56}H_{83}B_8N_4{\cdot}C_{16}H_{11}O_4{\cdot}CH_4O$
M_r	1198.03
Crystal system, space group	Monoclinic, $P2_1/c$
Temperature (K)	150
a, *b*, *c* (Å)	16.3613(4), 21.5212(5), 20.6014(5)
β (°)	103.644(1)
V (Å^3)	7049.3(3)
Z	4
Radiation type	Mo*K*α
μ (mm^{-1})	0.07
Crystal size (mm)	0.55 × 0.33 × 0.25
Data collection	
Diffractometer	Bruker D8 - Venture
Absorption correction	Multi-scan<br>*SADABS2016*/2 - Bruker AXS area detector scaling and absorption correction
T_{min}, T_{max}	0.724, 0.744
No. of measured, independent and observed [$I > 2\sigma(I)$] reflections	185555, 13055, 10674
R_{int}	0.066
$(\sin \theta/\lambda)_{max}$ (Å^{-1})	0.606
Refinement	
$R[F^2 > 2\sigma(F^2)]$, $wR(F^2)$, S	0.065, 0.184, 0.72
No. of reflections	13055
No. of parameters	1002
No. of restraints	957
H-atom treatment	H atoms treated by a mixture of independent and constrained refinement
	$w = 1/[\sigma^2(F_o^2) + (0.1275P)^2 + 13.2699P]$<br>where $P = (F_o^2 + 2F_c^2)/3$
$\Delta\rho_{max}$, $\Delta\rho_{min}$ (e Å^{-3})	0.67, −0.75

Synthesis of [6,9-IPr$_2$-5,10-C$_2$B$_8$H$_{11}$]$^+$[H$_3$CC(O)CHC(O)CH$_3$]$^-$ (*o*-2h)

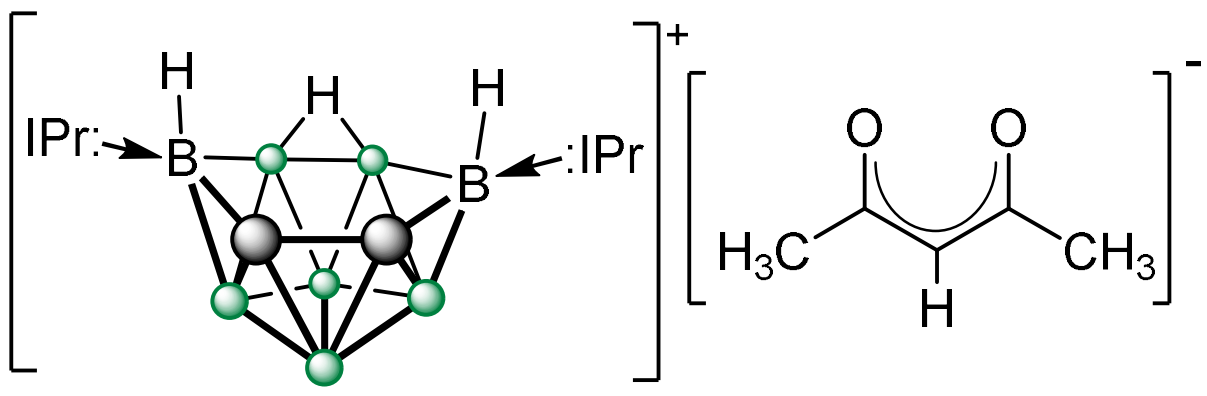

Acetylacetone (54 µL, 0.53 mmol) was added to a stirred solution of *o*-**2** (119 mg, 0.13 mmol) in diethyl ether (5 mL) at room temperature. The resulting suspension was stirred overnight and filtrated. The solid was washed with diethylether (10 mL) and the resulting colourless powder of *o*-**2h** was dried *in vacuo*. Yield 113 mg, 85%. **Dp** 236 °C. **^{1}H NMR** (25 °C, CD$_3$CN, 500 MHz): In the spectra of *o*-**2h**, two sets of signals are present. The minor set corresponds to the parent *o*-**2** and acetylacetone. However, the exchange between acac and acacH is too fast, thus only *o*-**2** could be observed. The signals of *o*-**2** are marked with *. δ = −4.25 (s broad, 1H, B*H*B), −0.85 (s, 1H, B3), −0.22 (s, 2H, B6,9), 0.03 (s, 2H, BC*H**), 0.58 (s, 2H, BC*H*), 1.03 (d, $^3J(^1H\text{-}^1H)$ = 6.8 Hz, 12H, CH(C*H*$_3$)$_2$*), 1.04 (d, $^3J(^1H\text{-}^1H)$ = 6.8 Hz, 12H, CH(C*H*$_3$)$_2$*), 1.06 (d, $^3J(^1H\text{-}^1H)$ = 6.8 Hz, 14H, CH(C*H*$_3$)$_2$ + B7,8), 1.10 (d, $^3J(^1H\text{-}^1H)$ = 6.8 Hz, 12H, CH(C*H*$_3$)$_2$), 1.13 (d, $^3J(^1H\text{-}^1H)$ = 6.8 Hz, 12H, CH(C*H*$_3$)$_2$), 1.17 (d, $^3J(^1H\text{-}^1H)$ = 6.8 Hz, 12H, CH(C*H*$_3$)$_2$), 1.19 (d, $^3J(^1H\text{-}^1H)$ = 6.8 Hz, 12H, CH(C*H*$_3$)$_2$*), 1.20 (d, $^3J(^1H\text{-}^1H)$ = 6.8 Hz, 12H, CH(C*H*$_3$)$_2$*), 1.51 (s, 1H, B1), 1.70 (s, 2H, B2,4), 1.87 (s broad, 6H, C(O)C*H*$_3$), 2.18 (sept, $^3J(^1H\text{-}^1H)$ = 6.8 Hz, 4H, C*H*(CH$_3$)$_2$), 2.39 (sept, $^3J(^1H\text{-}^1H)$ = 6.8 Hz, 4H, C*H*(CH$_3$)$_2$), 2.46 (sept, $^3J(^1H\text{-}^1H)$ = 6.8 Hz, 4H, C*H*(CH$_3$)$_2$*), 2.58 (sept, $^3J(^1H\text{-}^1H)$ = 6.8 Hz, 4H, C*H*(CH$_3$)$_2$), 4.73 (s broad, 1H, C*H*C(O)CH$_3$), 7.03 (s, 4H, NC*H**), 7.22 (d, $^3J(^1H\text{-}^1H)$ = 7.7, 8H, *m*-C$_6$*H*$_3$*), 7.31 (d, $^3J(^1H\text{-}^1H)$ = 7.7 Hz, 4H, *m*-C$_6$*H*$_3$), 7.32 (d, $^3J(^1H\text{-}^1H)$ = 7.7 Hz, 4H, *m*-C$_6$*H*$_3$), 7.44 (s, 4H, NC*H*), 7.44 (t, $^3J(^1H\text{-}^1H)$ = 7.6 Hz, 4H, *p*-C$_6$*H*$_3$*), 7.56 (t, $^3J(^1H\text{-}^1H)$ = 7.8 Hz, 4H, *p*-C$_6$*H*$_3$) ppm. **^{13}C{^{1}H} NMR** (25 °C, CD$_3$CN, 125.76 Hz): δ = 19.6 (s broad, B*C*H*), 22.6 (s, CH(*C*H$_3$)$_2$), 23.2 (s, CH(*C*H$_3$)$_2$*), 23.4 (s, CH(*C*H$_3$)$_2$), 23.6 (s, CH(*C*H$_3$)$_2$*), 25.6 (s, CH(*C*H$_3$)$_2$), 25.7 (s, CH(*C*H$_3$)$_2$*), 25.8 (s, CH(*C*H$_3$)$_2$*), 25.9 (s, CH(*C*H$_3$)$_2$) 26.9 (s broad, B*C*H), 28.7 (s, C(O)*C*H$_3$), 29.6 (s, *C*H(CH$_3$)$_2$*), 29.7 (s, *C*H(CH$_3$)$_2$*), 29.8(s, *C*H(CH$_3$)$_2$, 29.8 (s, *C*H(CH$_3$)$_2$,), 99.9 (s, *C*HC(O)CH$_3$), 124.3 (s, N*C*H*), 124.6 (s, N*C*H*), 124.8 (s, *m*-*C*$_6$H$_3$*), 125.1 (s, *m-C*$_6$H$_3$), 125.8 (s, N*C*H), 126.0 (s, N*C*H)), 130.9 (s, *p-C*$_6$H$_3$*), 132.3 (s, *p-C*$_6$H$_3$*), 133.4 (s, *ipso-C*$_6$H$_3$), 135.6 (s, *ipso-C*$_6$H$_3$*), 146.2 (s, *o-C*$_6$H$_3$), 146.4 (s, *o-C*$_6$H$_3$*), 146.8 (s, *o-C*$_6$H$_3$), 147.1 (s, *o-C*$_6$H$_3$*), 159.3 (s broad, B*C*N), 172.4 (s broad, B*C*N*), 188.8 (s broad, CH*C*(O)CH$_3$) ppm. **^{11}B NMR** (25 °C, CD$_3$CN, 160.42 MHz): δ = −54.9 (d, $^1J(^{11}B\text{-}^1H)$ = 165 Hz, 1B, B3), −50.6 (d, $^1J(^{11}B\text{-}^1H)$ = 136 Hz, 1B, B3*), −35.4 (d, $^1J(^{11}B\text{-}^1H,)$ = 132 Hz, 2B, B6,9*), −34.2 (s broad, 1B, B1*), −31.6 (d, $^1J(^{11}B\text{-}^1H)$ = 119 Hz, 2B, B6,9), −22.8 (d, $^1J(^{11}B\text{-}^1H,)$ = 158 Hz, 1B, B1), −7.3 (s broad, 2B, B2,4 + B2,4*), −4.8 (s broad, 2B, B7,8 + B7,8*) ppm.

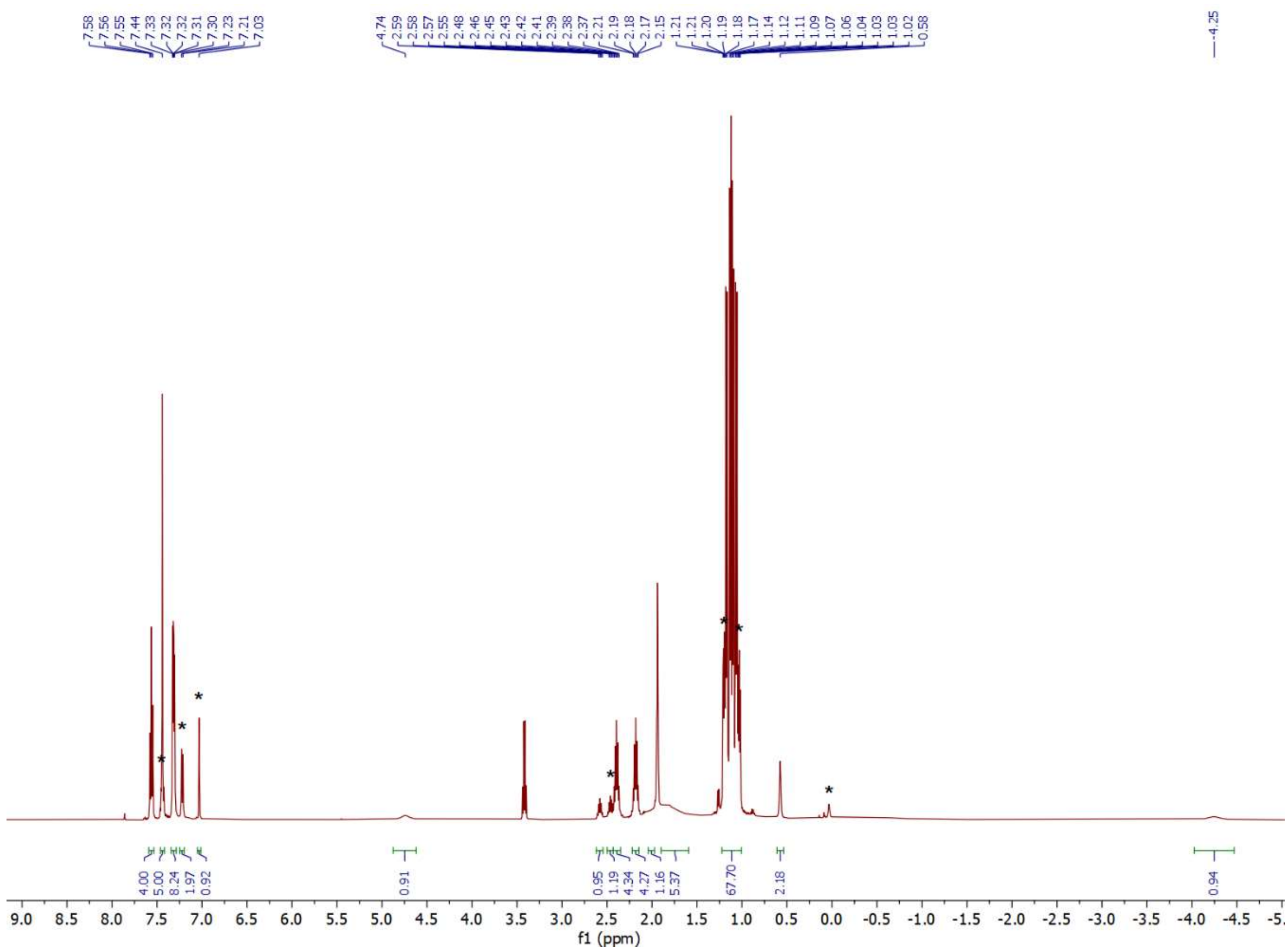

Supplementary Figure S41. The ^1^H (500.1 MHz) NMR spectrum of *o*-2h in CD_3CN. The non-overlapping signals of *o*-2 are marked with *.

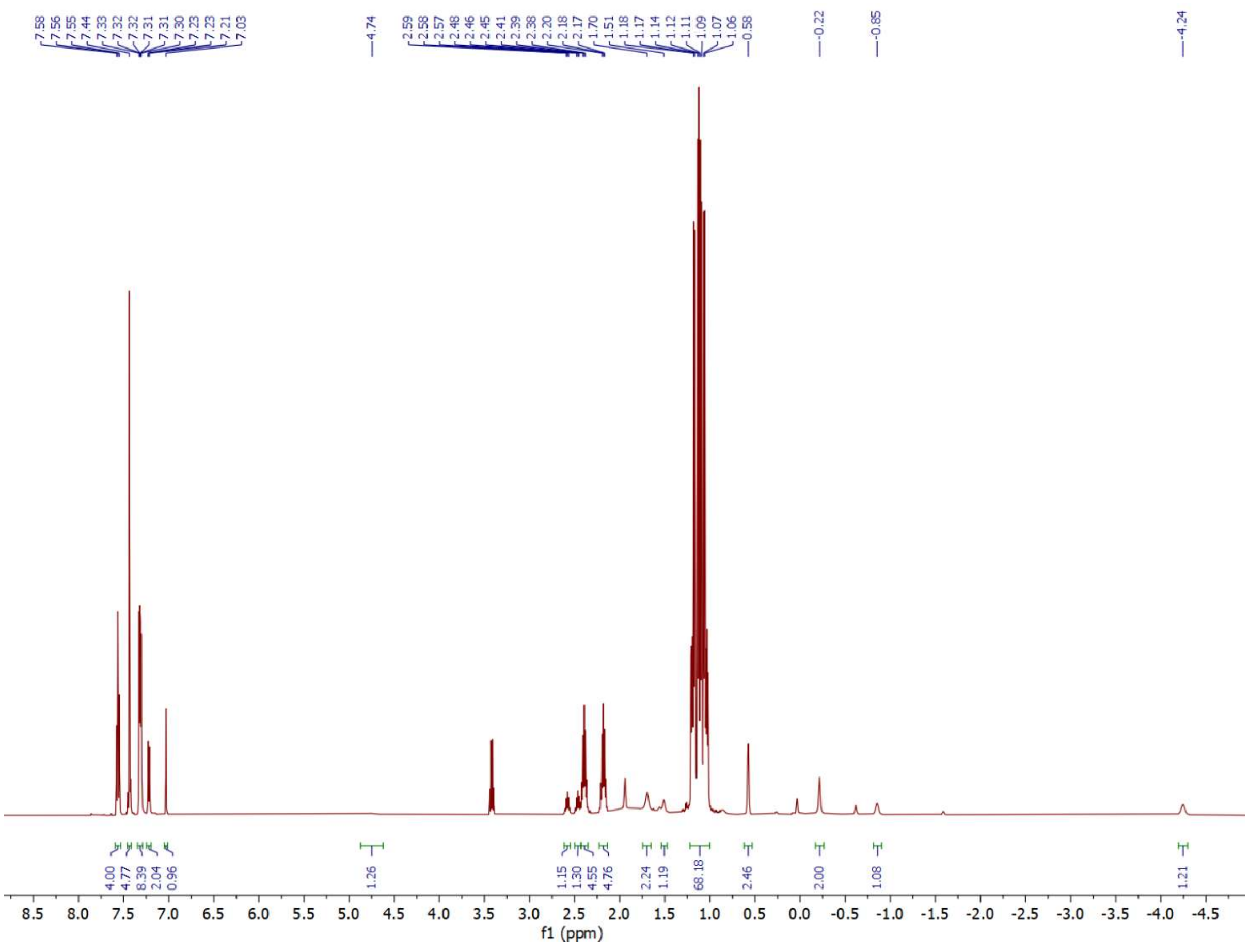

Supplementary Figure S42. The $^{1}H\{^{11}B\}$ (500.1 MHz) NMR spectrum of *o*-2h in CD_3CN.

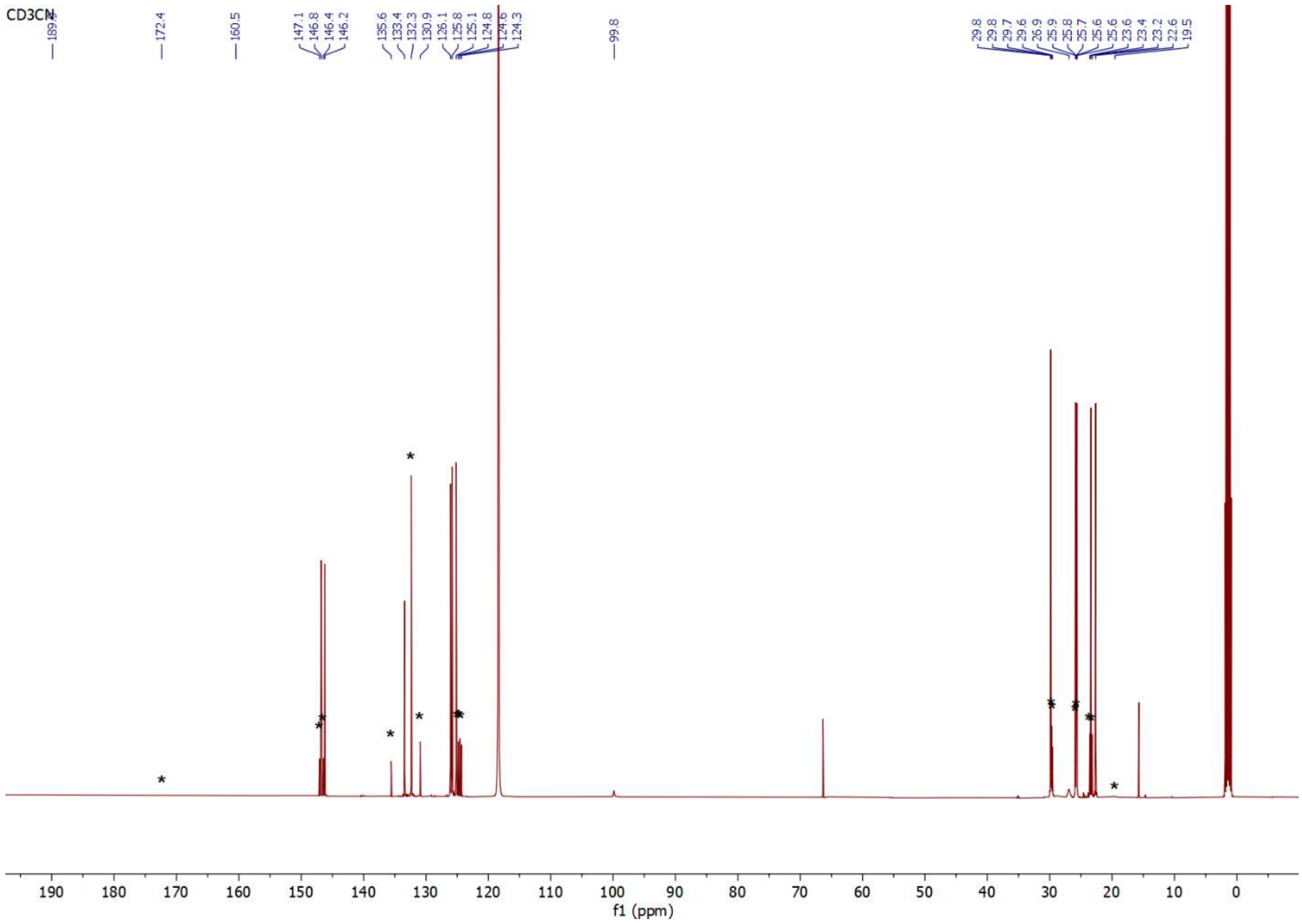

Supplementary Figure S43. The $^{13}C\{^1H\}$ (125.8 MHz) NMR spectrum of *o*-2h in CD_3CN. The non-overlapping signals of *o*-2 are marked with *.

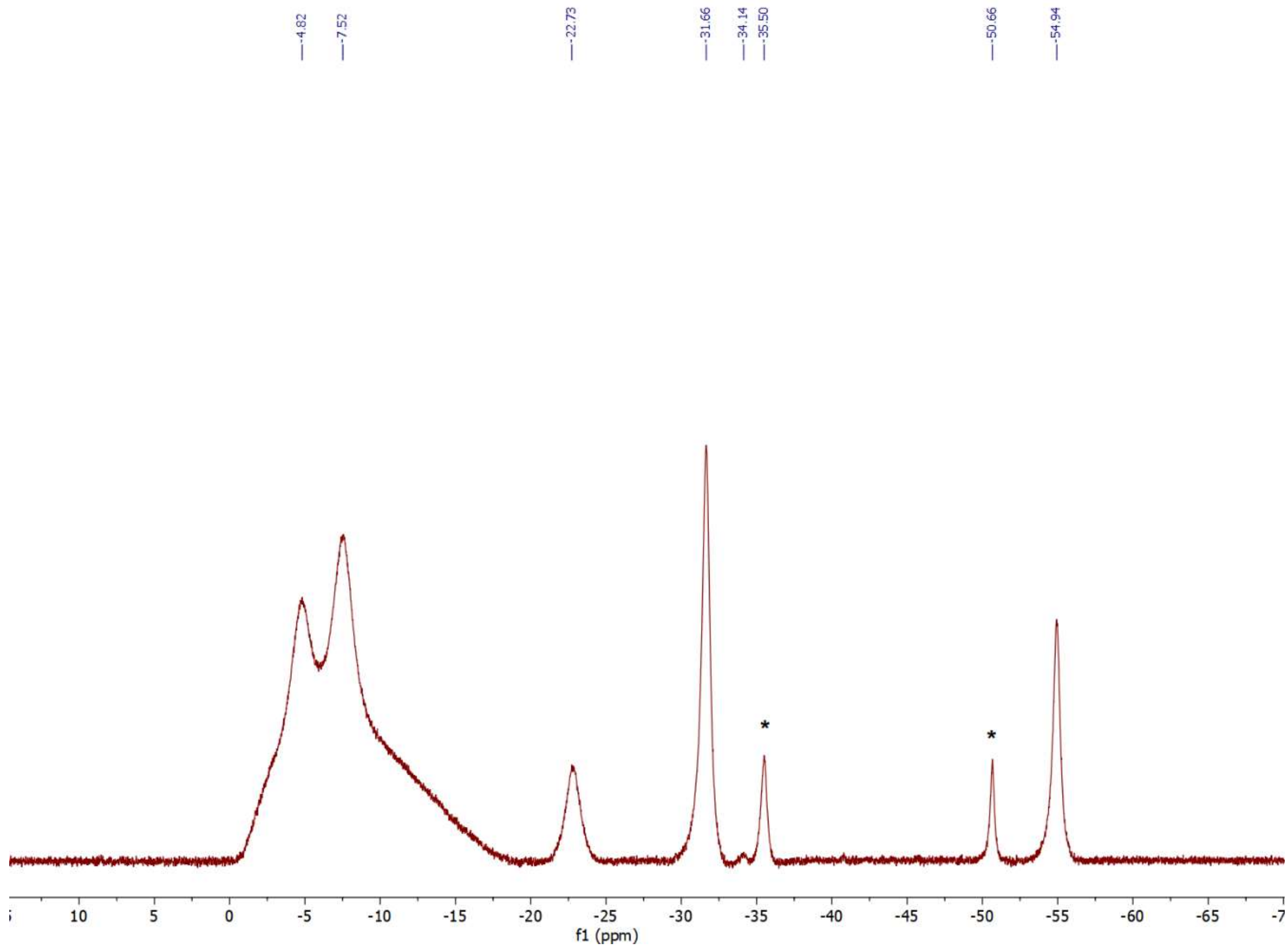

Supplementary Figure S44. The $^{11}B\{^1H\}$ (160.4) NMR spectrum of *o*-2h in CD_3CN. The non-overlapping signals of *o*-2 are marked with *.

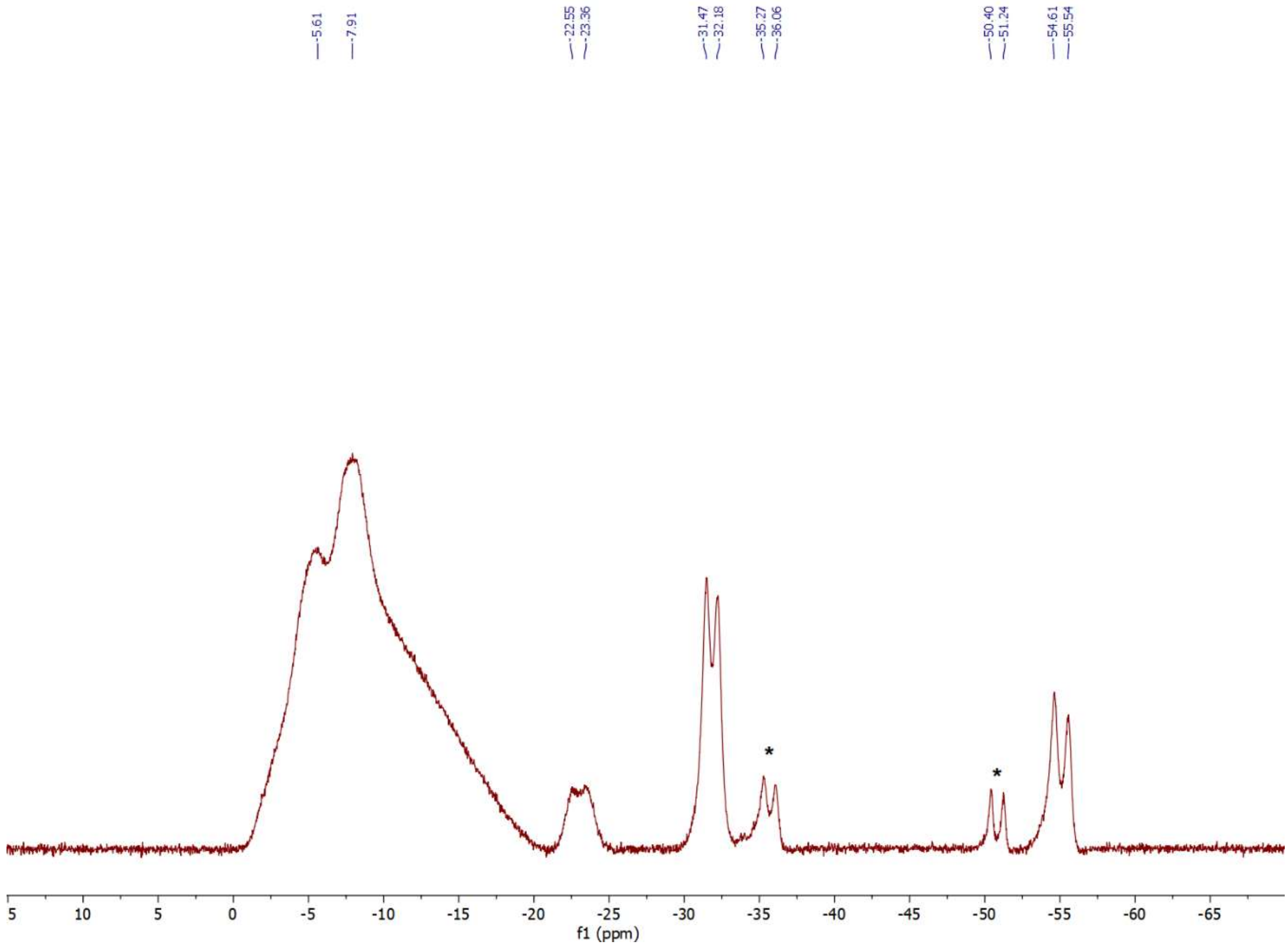

Supplementary Figure S45. The ^{11}B NMR (160.4) spectrum of *o*-2h in CD_3CN. The non-overlapping signals of *o*-2 are marked with *.

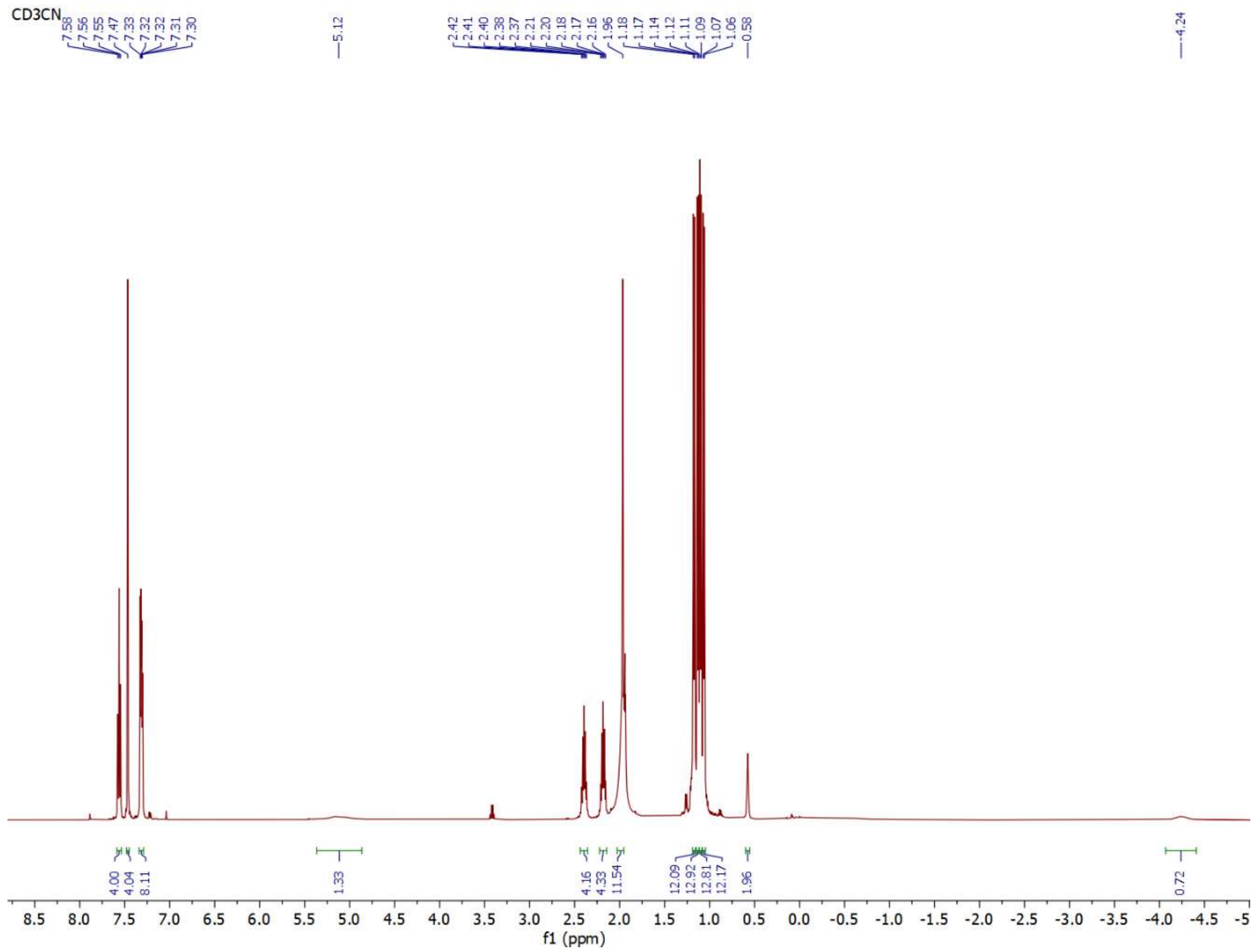

Supplementary Figure S46. The ^{1}H (500.1 MHz) NMR spectrum of the reaction mixture of *o*-2h with one equivalent of acetylacetone in CD_3CN.

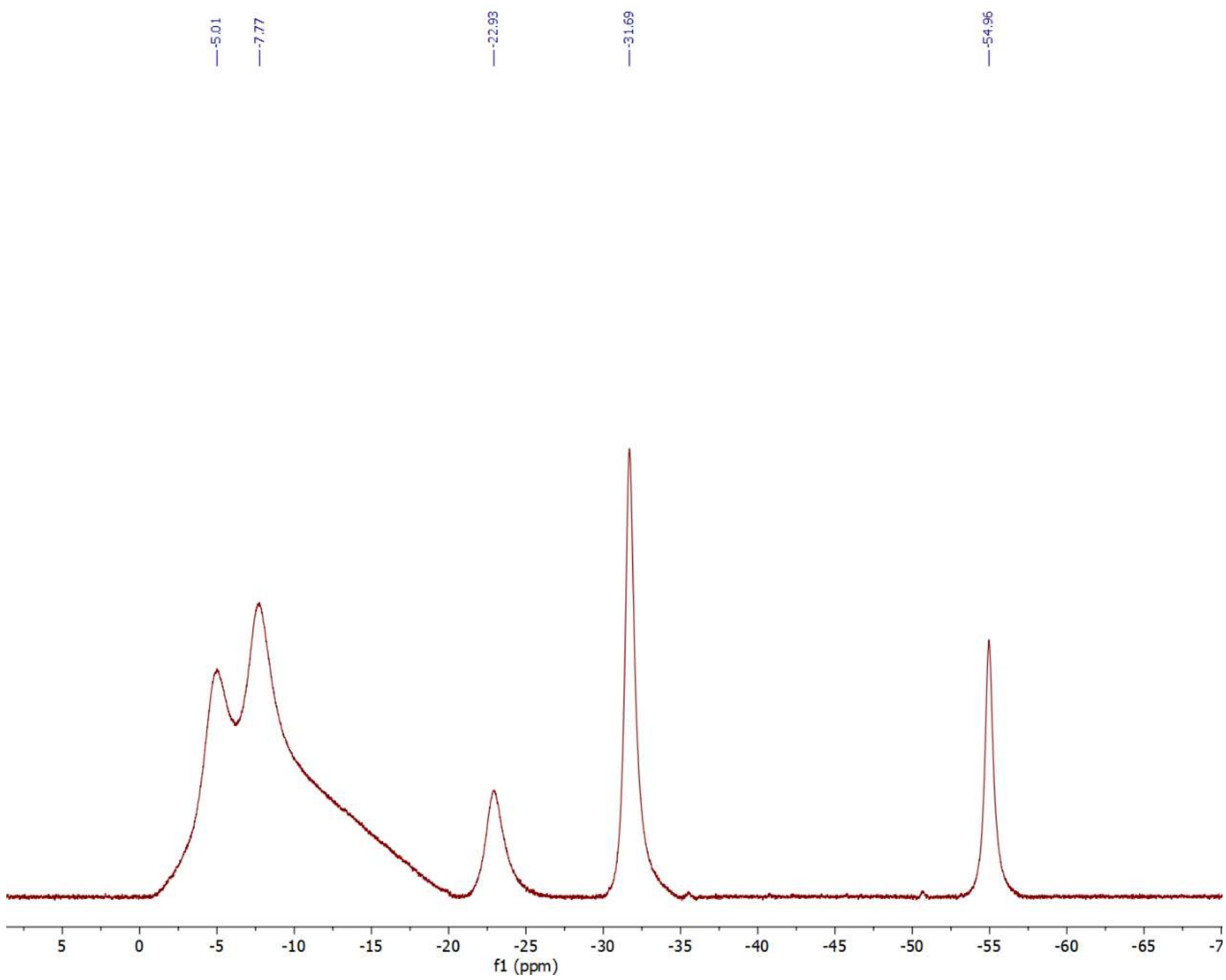

Supplementary Figure S47. The $^{11}B\{^1H\}$ (160.4) NMR spectrum of the reaction mixture of *o*-2h with one equivalent of acetylacetone in CD_3CN.

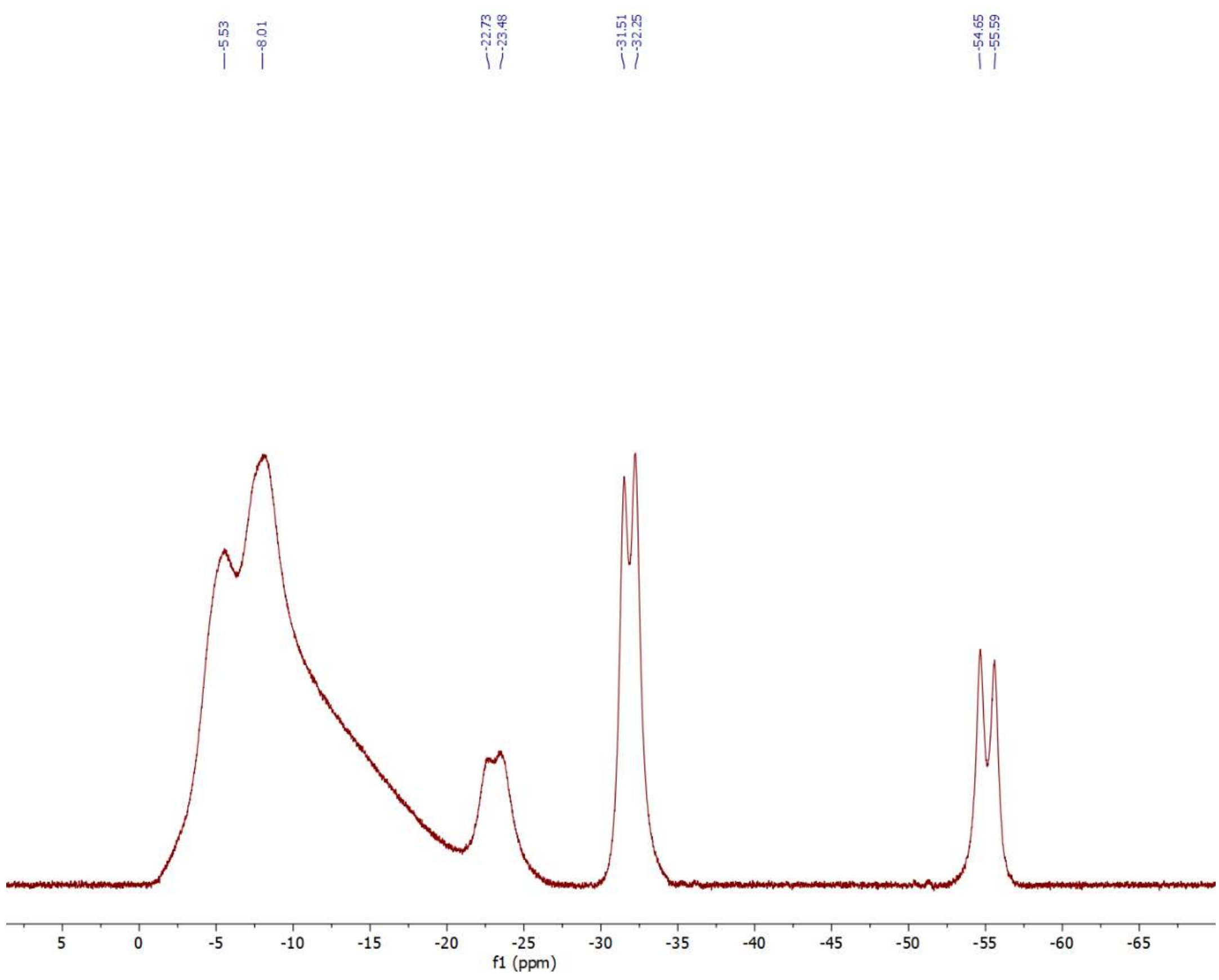

Supplementary Figure S48. The ^{11}B (160.4) NMR spectrum of the reaction mixture of *o*-2h with one equivalent of acetylacetone in CD_3CN.

Synthesis of [6,9-IPr$_2$-5,10-C$_2$B$_8$H$_{11}$]$^+$[PhC(O)CHC(O)Ph]$^-$ (*o*-2i)

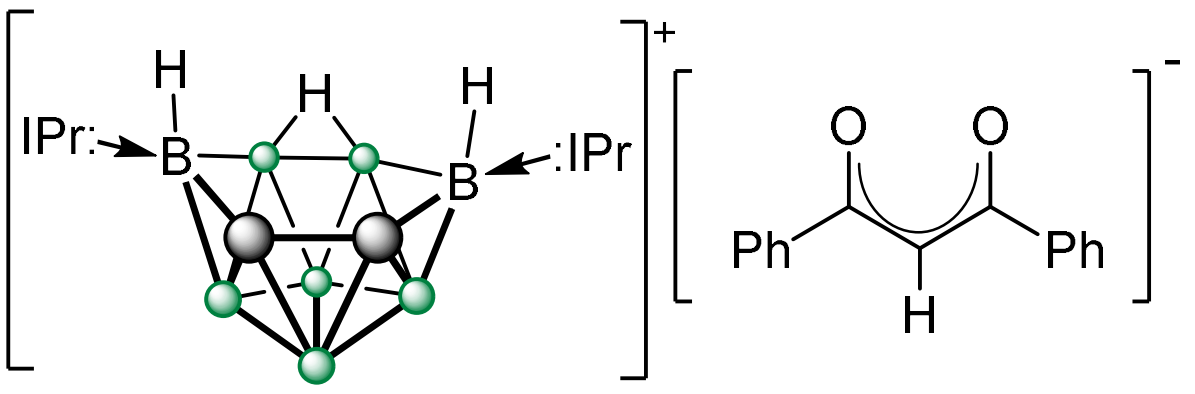

Solution of 1,3-diphenyl-1,3-propanedione (34 mg, 0.15 mmol) in toluene (5 mL) was added to a stirred solution of *o*-**2** (113 mg, 0.13 mmol) in toluene (15 mL) at room temperature. The resulting suspension was stirred overnight and filtrated. The solid was washed with toluene (10 mL) and the resulting colourless powder of *o*-**2i** was dried *in vacuo*. The crystals suitable for X-ray diffraction analysis were grown from a saturated solution of *o*-**2i** in acetonitrile at 4 °C. Yield 77 mg, 55%. **Dp** 186 °C. **^{1}H NMR** (25 °C, CD$_3$CN, 500 MHz):): In the spectra of *o*-**2i**, two sets of signals are present. The minor set corresponds to the parent *o*-**2** and 1,3-diphenyl-1,3-propanedione. However, the exchange between 1,3-diphenyl-1,3-propanedione and its deprotonated form is too fast, thus only *o*-**2** could be observed. The signals of *o*-**2** are marked with *. δ = −4.22 (s broad, 1H, B*H*B), −0.86 (s, 1H, B3), −0.22 (s, 2H, B6,9), 0.04 (s, 2H, BC*H**), 0.57 (s, 2H, BC*H*), 1.03 (d, $^3J(^1H-^1H)$ = 6.8 Hz, 12H, CH(C*H*$_3$)$_2$*), 1.04 (d, $^3J(^1H-^1H)$ = 6.8 Hz, 12H, CH(C*H*$_3$)$_2$*), 1.06 (d, $^3J(^1H-^1H)$ = 6.8 Hz, 14H, CH(C*H*$_3$)$_2$ + B7,8), 1.10 (d, $^3J(^1H-^1H)$ = 6.8 Hz, 12H, CH(C*H*$_3$)$_2$), 1.13 (d, $^3J(^1H-^1H)$ = 6.8 Hz, 12H, CH(C*H*$_3$)$_2$), 1.17 (d, $^3J(^1H-^1H)$ = 6.8 Hz, 12H, CH(C*H*$_3$)$_2$), 1.20 (d, $^3J(^1H-^1H)$ = 6.8 Hz, 12H, CH(C*H*$_3$)$_2$*), 1.21 (d, $^3J(^1H-^1H)$ = 6.8 Hz, 12H, CH(C*H*$_3$)$_2$*), 1.51 (s, 1H, B1), 1.69 (s, 2H, B2,4), 2.18 (sept, $^3J(^1H-^1H)$ = 6.8 Hz, 4H, C*H*(CH$_3$)$_2$), 2.39 (sept, $^3J(^1H-^1H)$ = 6.8 Hz, 4H, C*H*(CH$_3$)$_2$), 2.47 (sept, $^3J(^1H-^1H)$ = 6.8 Hz, 4H, C*H*(CH$_3$)$_2$*), 2.58 (sept, $^3J(^1H-^1H)$ = 6.8 Hz, 4H, C*H*(CH$_3$)$_2$), 6.15 (broad s, 1H, C*H*C(O)Ph), 7.03 (s, 4H, NC*H**), 7.22 (d, $^3J(^1H.1H)$ = 7.7, 8H, *m*-C$_6$*H*$_3$*), 7.24-7.35 (m, 14H, *m*-C$_6$*H*$_3$ + *m*-C$_6$*H*$_5$ + *p*-C$_6$*H*$_5$), 7.44 (s, 4H, NC*H*), 7.44 (t, $^3J(^1H-^1H)$ = 7.8 Hz, 4H, *p*-C$_6$*H*$_3$*), 7.56 (t, $^3J(^1H-^1H)$ = 7.8 Hz, 4H, *p*-C$_6$*H*$_3$), 7.80 (s broad, 4H, *o*-C$_6$*H*$_5$) ppm. **^{13}C{^{1}H} NMR** (25 °C, CD$_3$CN, 125.76 Hz): δ = 19.5 (broad s, B*C*H*), 22.6 (s, CH(*C*H$_3$)$_2$), 23.2 (s, CH(*C*H$_3$)$_2$*), 23.4 (s, CH(*C*H$_3$)$_2$), 23.5 (s, CH(*C*H$_3$)$_2$*), 25.6 (s, CH(*C*H$_3$)$_2$), 25.7 (s, CH(*C*H$_3$)$_2$*), 25.7 (s, CH(*C*H$_3$)$_2$*), 25.8 (s, CH(*C*H$_3$)$_2$) 26.9 (broad s, B*C*H), 29.6 (s, *C*H(CH$_3$)$_2$*), 29.7 (s, *C*H(CH$_3$)$_2$*), 29.8 (s, *C*H(CH$_3$)$_2$, 91.8 (s, *C*HC(O)Ph), 124.3 (s, *m*-*C*$_6$H$_3$*), 124.6 (s, N*C*H*), 124.8 (s, N*C*H*), 125.1 (s, *m*-*C*$_6$H$_3$), 125.8 (s, N*C*H), 126.0 (s, N*C*H), 127.8 (s, *o*-*C*$_6$H$_5$), 128.5 (*m*-*C*$_6$H$_5$), 129.4 (*p*-*C*$_6$H$_5$), 130.9 (*ipso*-*C*$_6$H$_5$), 132.3 (s, *p*-*C*$_6$H$_3$), 133.4 (s, *ipso*-*C*$_6$H$_3$), 135.6 (CH*C*(O)Ph), 146.2 (s, *o*-*C*$_6$H$_3$), 146.4 (s, *o*-*C*$_6$H$_3$*), 146.8 (s, *o*-*C*$_6$H$_3$), 147.1 (s, *o*-*C*$_6$H$_3$*), 160.1 (broad s, B*C*N), 183.1 (broad s, B*C*N*) ppm. **^{11}B NMR** (25 °C, CD$_3$CN, 160.42 MHz): δ = −55.0 (d, $^1J(^{11}B-^1H)$ = 147 Hz, 1B, B3), −50.7 (d, $^1J(^{11}B-^1H)$ = 110 Hz, 1B, B3*), −35.5 (d, $^1J(^{11}B-^1H)$ = 116 Hz, 2B, B6,9*), −34.2 (broad s, 1B, B1*), −31.6 (d, $^1J(^{11}B-^1H)$ = 115 Hz, 2B, B6,9), −22.8 (d, $^1J(^{11}B-^1H)$ = 149 Hz, 1B, B1), −7.5 (broad s, 2B, B2,4 + B2,4*), −4.8 (broad s, 2B, B7,8 + B7,8*) ppm.

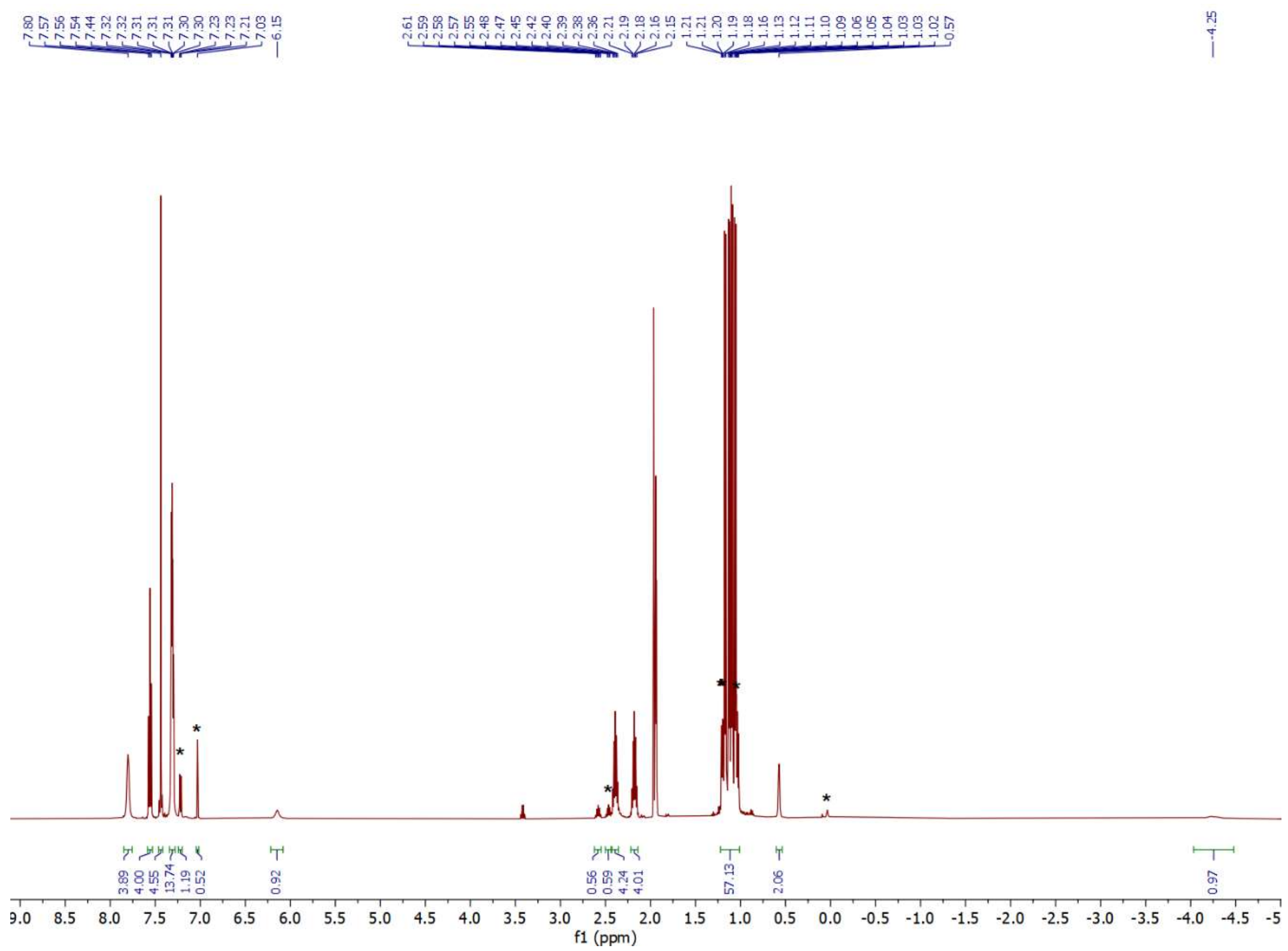

Supplementary Figure S49. The ^{1}H (500.1 MHz) NMR spectrum of *o*-**2i in CD_3CN. The non-overlapping signals of *o*-2 are marked with *.**

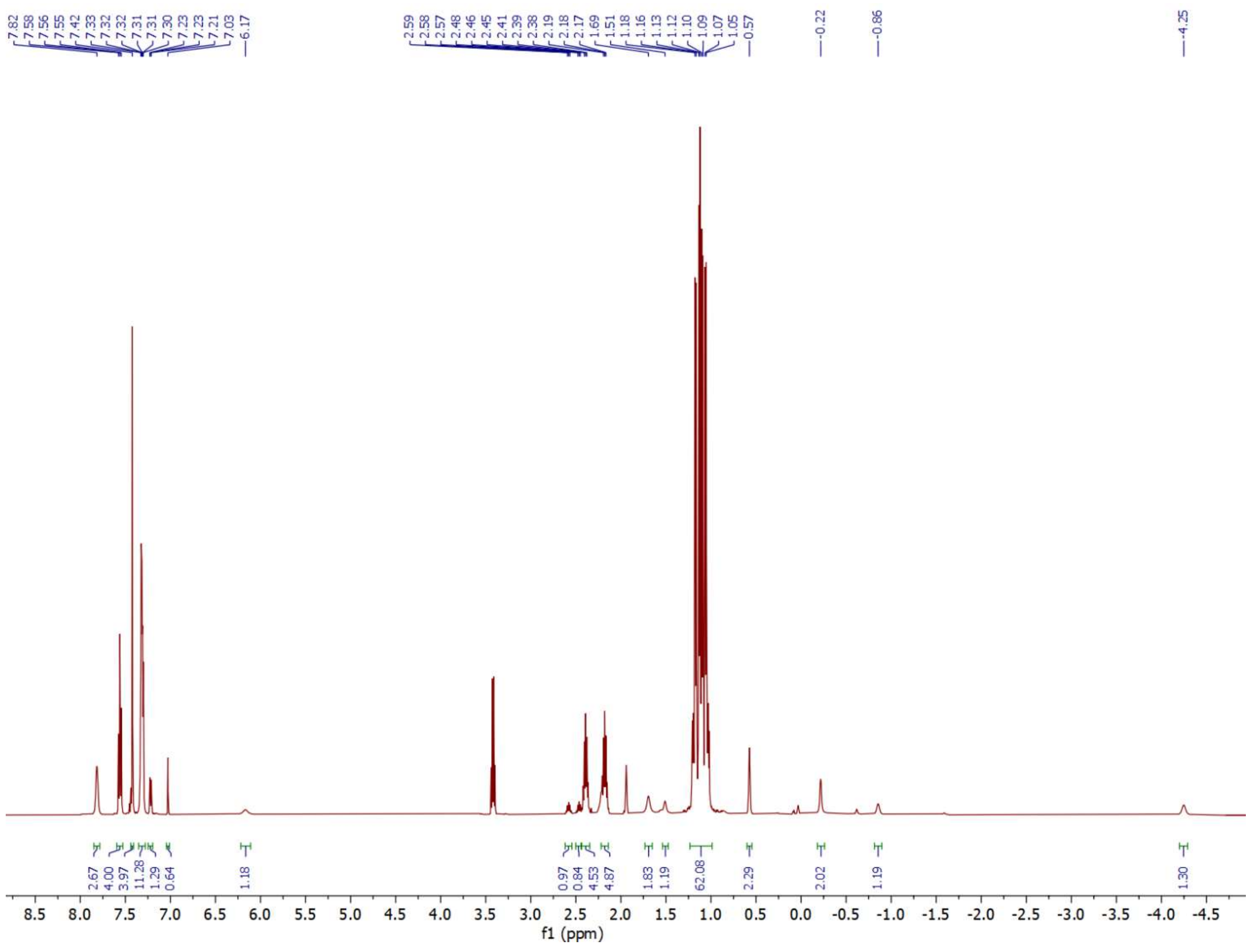

Supplementary Figure S50. The ^{1}H{^{11}B} (500.1 MHz) NMR spectrum of *o*-2i in CD_3CN.

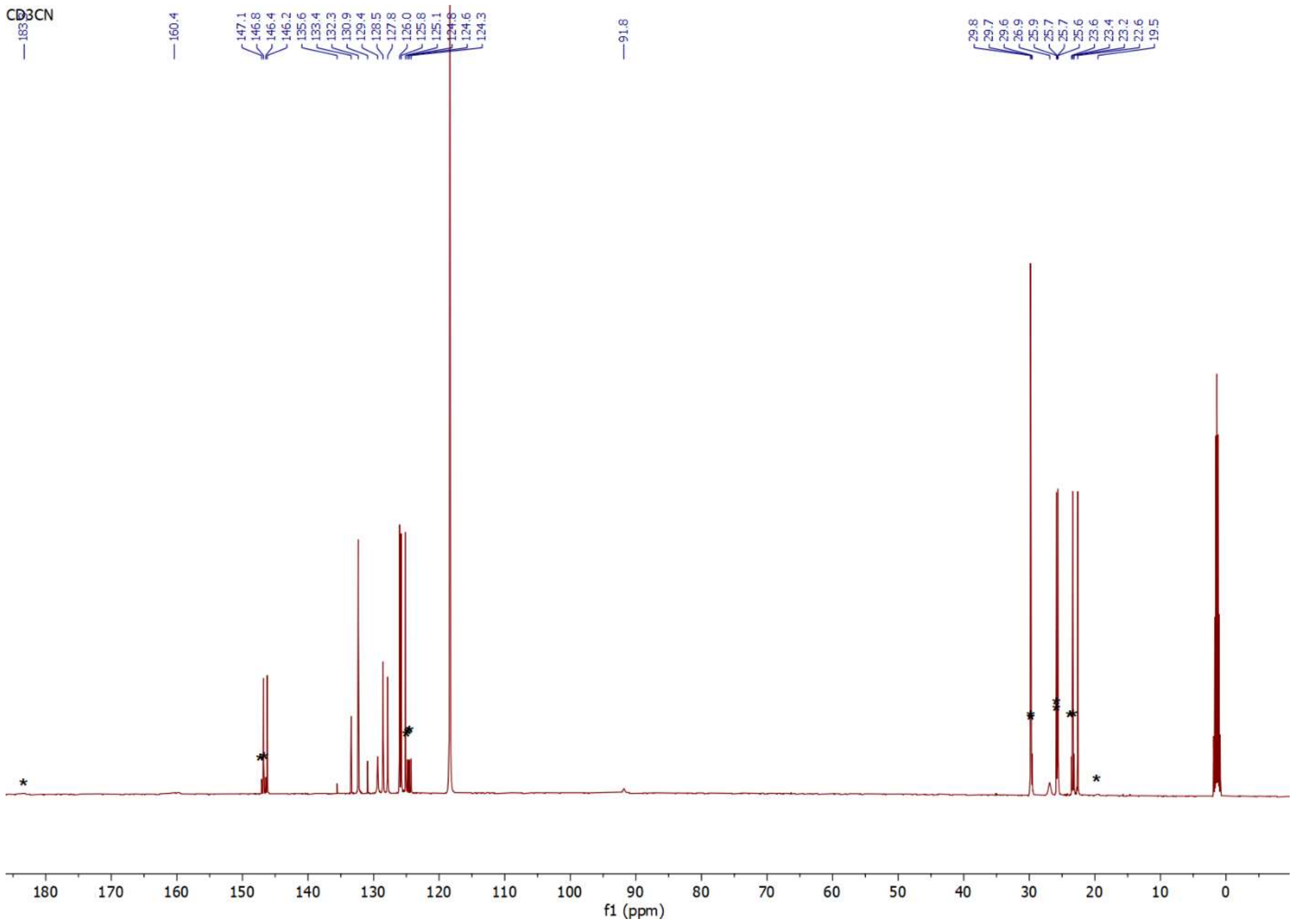

Supplementary Figure S51. The $^{13}C\{^1H\}$ (125.8 MHz) NMR spectrum of *o*-2i in CD_3CN. The non-overlapping signals of *o*-2 are marked with *.

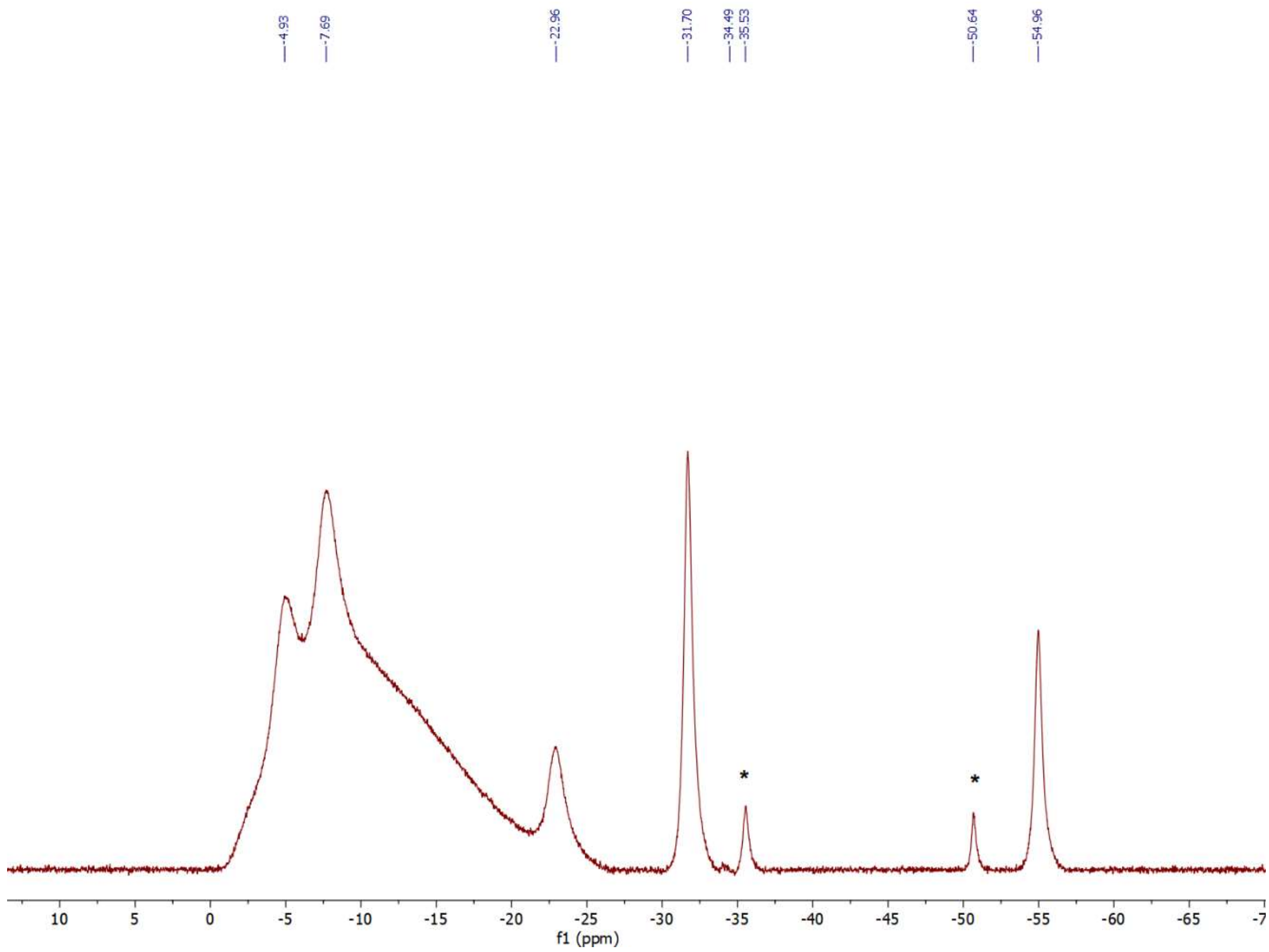

Supplementary Figure S52. The $^{11}B\{^1H\}$ (160.4) NMR spectrum of *o*-2i in CD_3CN. The non-overlapping signals of *o*-2 are marked with *.

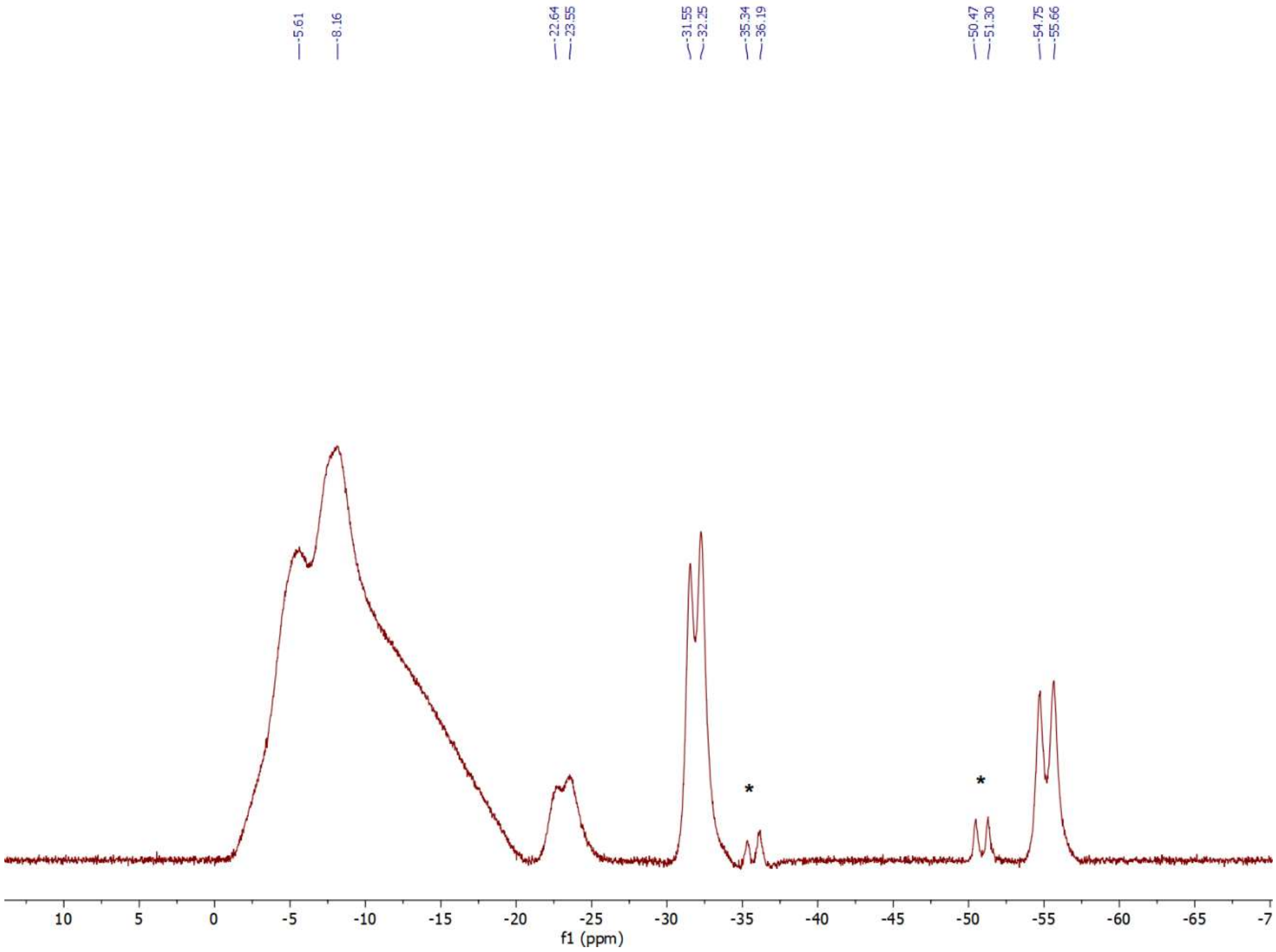

Supplementary Figure S53. The ^{11}B (160.4) NMR spectrum of *o*-2i in CD_3CN. The non-overlapping signals of *o*-2 are marked with *.

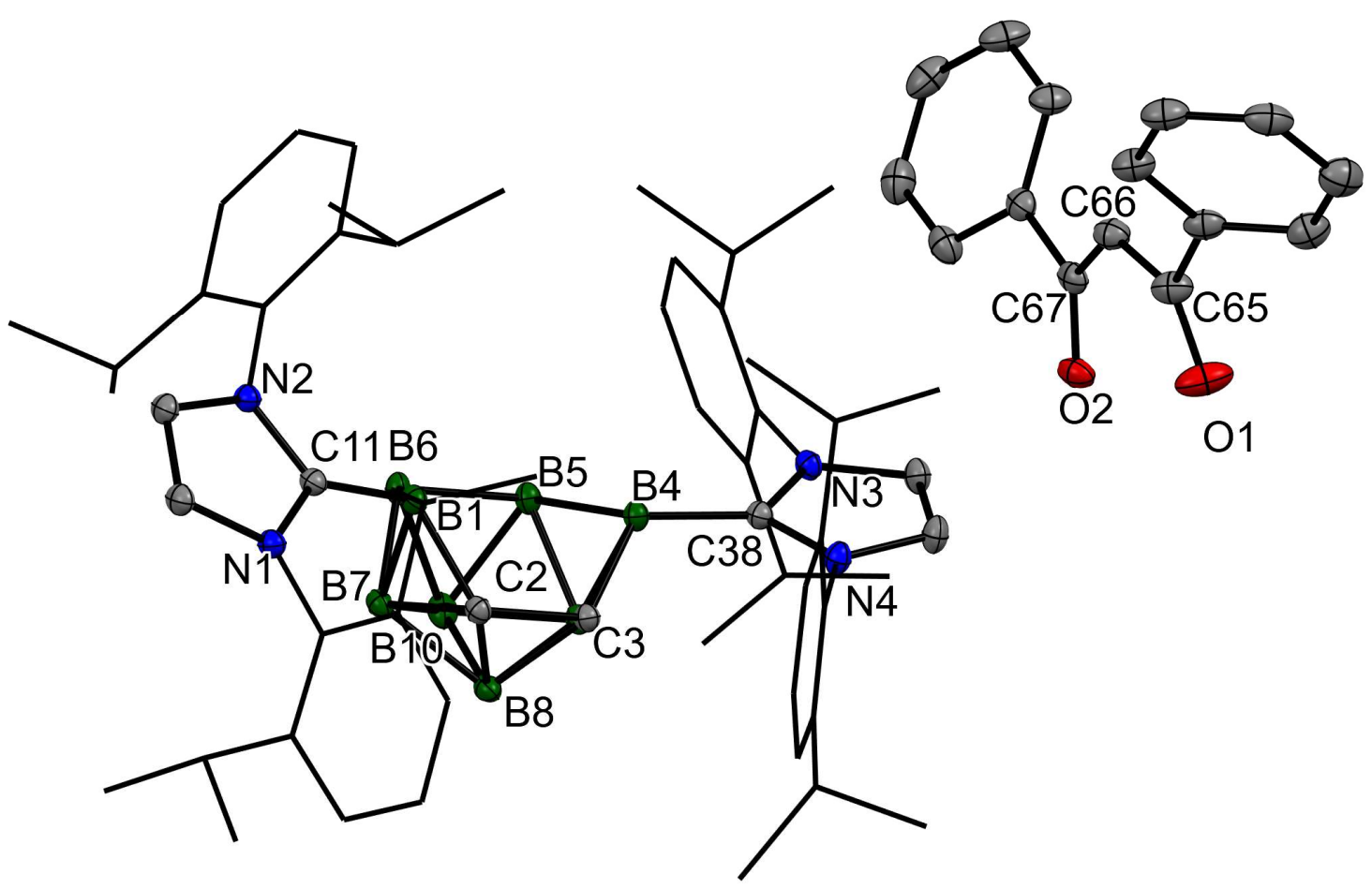

Supplementary Figure S54. The molecular structure of *o*-2i. ORTEP view, 30% probability level; solvent molecule (acetonitrile) has been omitted for clarity. Selected interatomic distances [Å]: B1-C2 1.6224(19), C2-C3 1.5371(17), C3-B4 1.6358(18), B1-B6 2.015(2), B4-B5 1.976(2), B5-B6 1. 1.838(2), C65-C66 1.416(2), C66-C67 1.4027(19), C67-O2 1.2629(16), C65-O1 1.2432(18).

Supplementary Table S6. Experimental details of *o*-2i.

Crystal data	
Chemical formula	$C_{56}H_{83}B_8N_4{\cdot}C_{15}H_{11}O_2{\cdot}C_2H_3N$
M_r	1163.03
Crystal system, space group	Triclinic, *P*-1
Temperature (K)	150
a, *b*, *c* (Å)	11.0639(4), 16.4575(5), 20.5432(7)
α, β, γ (°)	88.623(1), 77.242(1), 77.441(1)
V ($Å^3$)	3559.8(2)
Z	2
Radiation type	Mo*K*α
μ (mm^{-1})	0.06
Crystal size (mm)	0.47 × 0.26 × 0.13
Data collection	
Diffractometer	Bruker D8 - Venture
Absorption correction	Multi-scan<br>*SADABS2016*/2 - Bruker AXS area detector scaling and absorption correction Reference: Krause, L., Herbst-Irmer, R., Sheldrick G.M. & Stalke D., *J. Appl. Cryst.* 2015, 48, 3–10.
T_{min}, T_{max}	0.708, 0.746
No. of measured, independent and observed [$I > 2\sigma(I)$] reflections	248488, 16332, 13691
R_{int}	0.060
$(\sin \theta/\lambda)_{max}$ ($Å^{-1}$)	0.650
Refinement	
$R[F^2 > 2\sigma(F^2)]$, $wR(F^2)$, S	0.048, 0.134, 1.03
No. of reflections	16332
No. of parameters	897
No. of restraints	877
H-atom treatment	H atoms treated by a mixture of independent and constrained refinement
$\Delta\rho_{max}$, $\Delta\rho_{min}$ (e $Å^{-3}$)	0.63, −0.59

Synthesis of $[6,9\text{-IPr}_2\text{-}5,10\text{-}C_2B_8H_{10}D]^+[CD_3O]^-$ (*o*-2j)

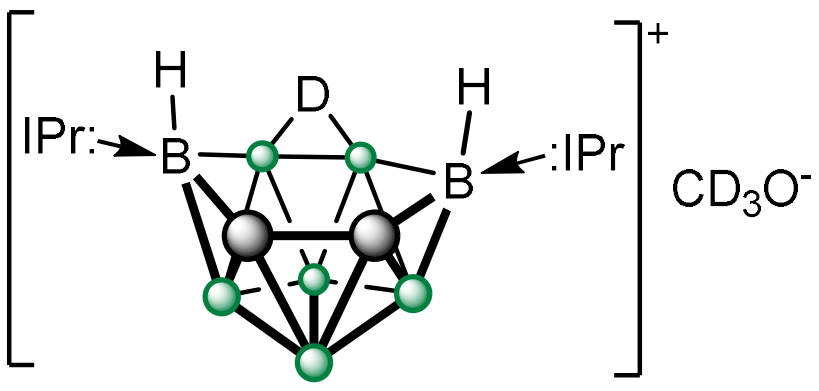

Compound *o*-**2** (15 mg, 0.017 mmol) was dissolved in deuterated methanol. The NMR spectra were measured immediately. **^{1}H NMR** (25 °C, CD_2Cl_2, 500 MHz): δ = −0.77 (s, 1H, B3), −0.17 (s, 2H, B6,9), 0.49 (s, 2H, BC*H*), 0.98 (d, $^3J(^1H\text{-}^1H)$ = 6.8 Hz, 12H, CH(C*H*$_3$)$_2$), 1.01 (d, $^3J(^1H\text{-}^1H)$ = 6.8 Hz, 14H, CH(C*H*$_3$)$_2$ + B7,8), 1.06 (d, $^3J(^1H\text{-}^1H)$ = 6.8 Hz, 12H, CH(C*H*$_3$)$_2$), 1.09 (d, $^3J(^1H\text{-}^1H)$ = 6.8 Hz, 12H, CH(C*H*$_3$)$_2$), 1.53 (s, 1H, B1), 1.72 (s, 2H, B2,4), 2.11 (sept, $^3J(^1H\text{-}^1H)$ = 6.8 Hz, 4H, C*H*(CH$_3$)$_2$), 2.30 (sept, $^3J(^1H\text{-}^1H)$ = 6.8 Hz, 4H, C*H*(CH$_3$)$_2$), 7.20 (d, $^3J(^1H\text{-}^1H)$ = 7.7 Hz, 4H, *m*-C$_6$*H*$_3$), 7.21 (d, $^3J(^1H\text{-}^1H)$ = 8.0 Hz, 4H, *m*-C$_6$*H*$_3$), 7.44 (t, $^3J(^1H\text{-}^1H)$ = 7.8 Hz, 4H, *p*-C$_6$*H*$_3$), 7.56 (s, 4H, NC*H*) ppm. **^{13}C{^{1}H} NMR** (25 °C, CD_2Cl_2, 125.76 Hz): δ = 22.9 (s, CH(*C*H$_3$)$_2$), 23.6 (s, CH(*C*H$_3$)$_2$), 25.7 (s, CH(*C*H$_3$)$_2$), 25.9 (s, CH(*C*H$_3$)$_2$), 27.0 (s broad, B*C*H), 30.2 (s, *C*H(CH$_3$)$_2$), 30.3 (s, *C*H(CH$_3$)$_2$), 125.4 (s, *m*-*C*$_6$H$_3$), 125.9 (s, N*C*H), 126.5 (s, N*C*H), 132.5 (s, *p*-*C*$_6$H$_3$), 134.0 (s, *ipso*-*C*$_6$H$_3$), 146.4 (s, *o*-*C*$_6$H$_3$), 147.0 (s, *o*-*C*$_6$H$_3$), 161.0 (s broad, B*C*N) ppm. **^{11}B NMR** (25 °C, CD_3CN, 160.42 MHz): δ = −55.0 (d, $^1J(^{11}B\text{-}^1H,)$ = 150 Hz, 1B, B3), −31.7 (d, $^1J(^{11}B\text{-}^1H,)$ = 108 Hz, 2B, B6,9), −22.7 (d, $^1J(^{11}B\text{-}^1H,)$ = 124 Hz, 1B, B1), −7.7 (s broad, 2B, B2,4), −4.8 (s broad, 2B, B7,8) ppm.

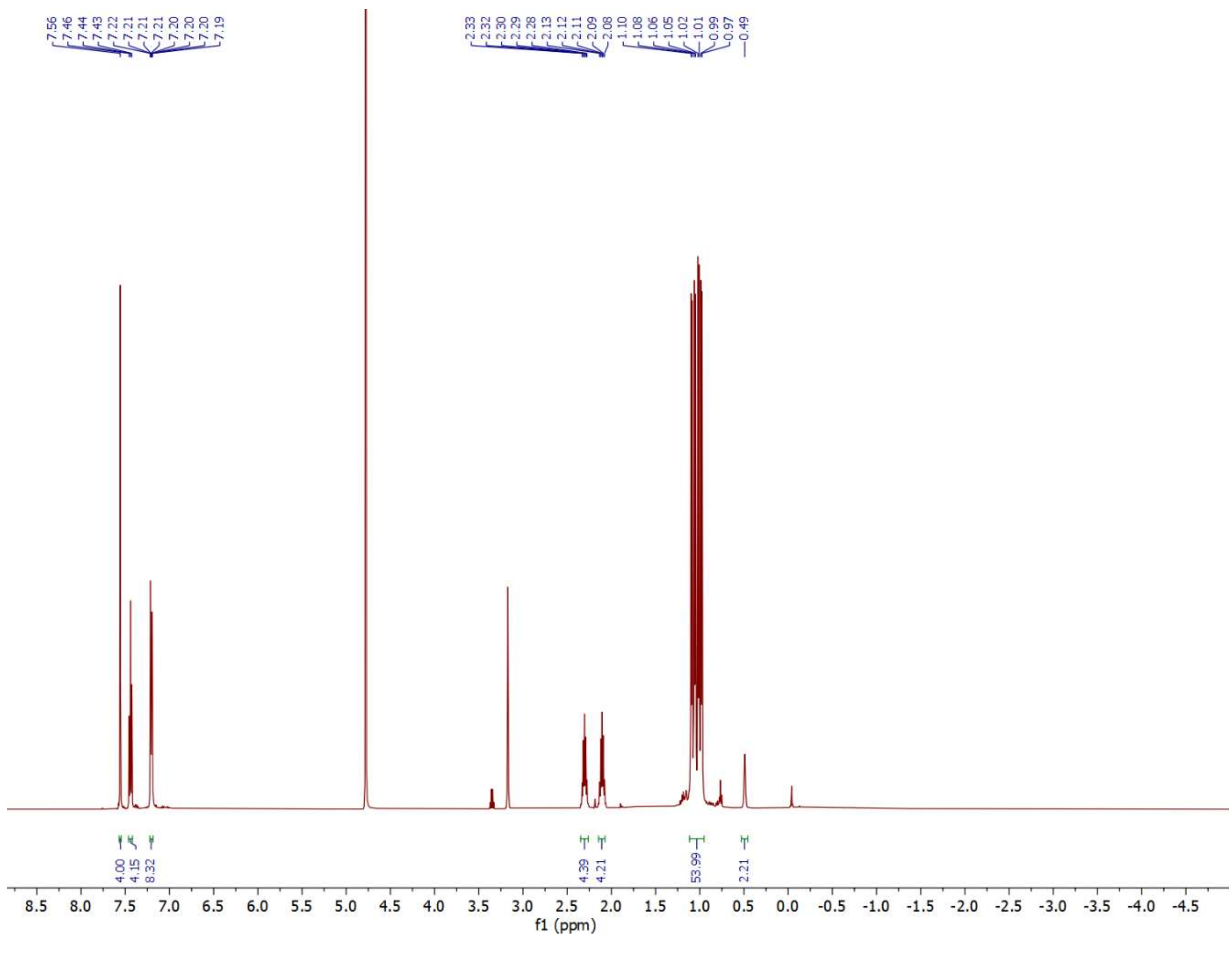

Supplementary Figure S55. The ^{1}H (500.1 MHz) NMR spectrum of *o*-2j in CD_3OD.

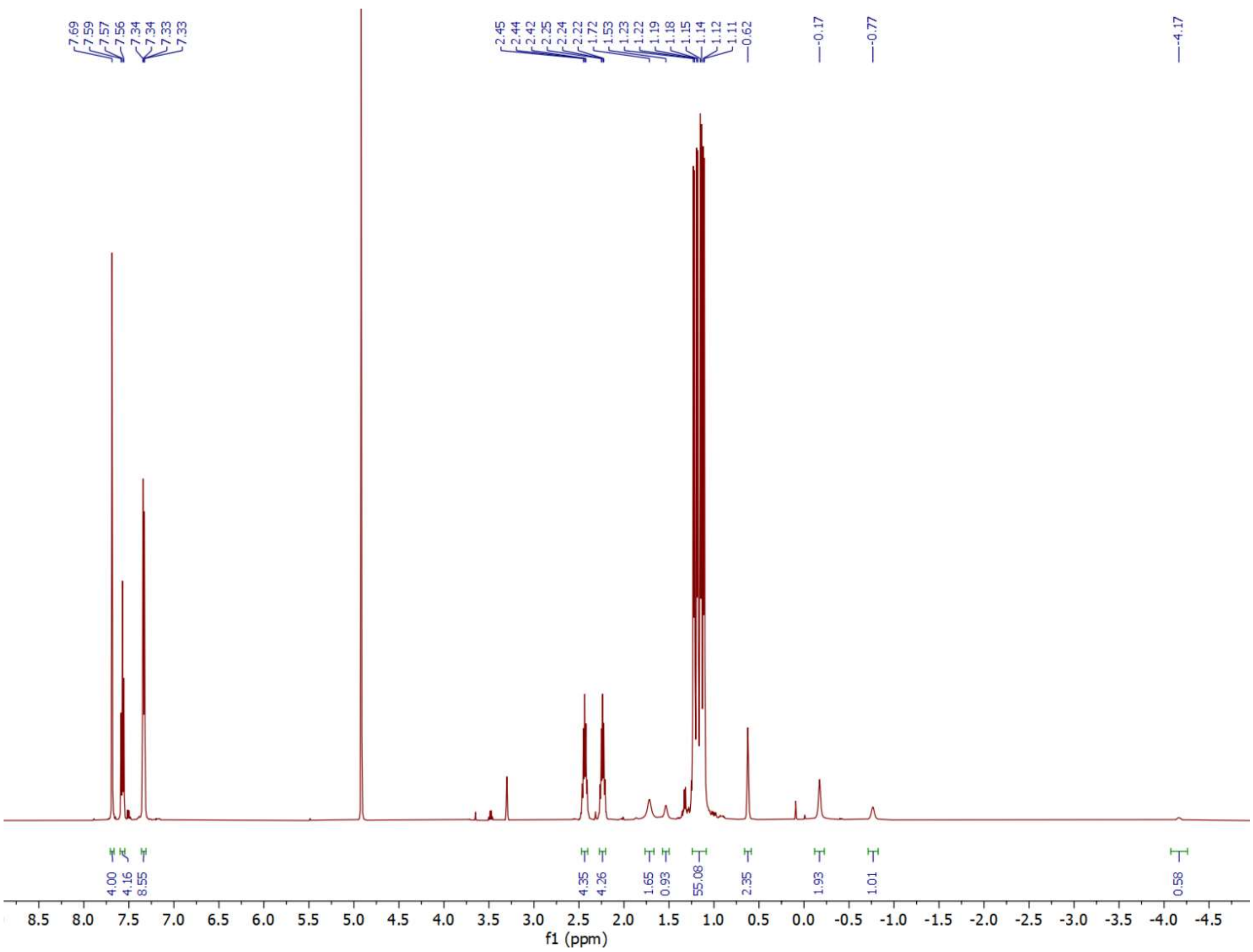

Supplementary Figure S56. The $^{1}H\{^{11}B\}$ (500.1) NMR spectrum of *o*-2j in CD_3OD.

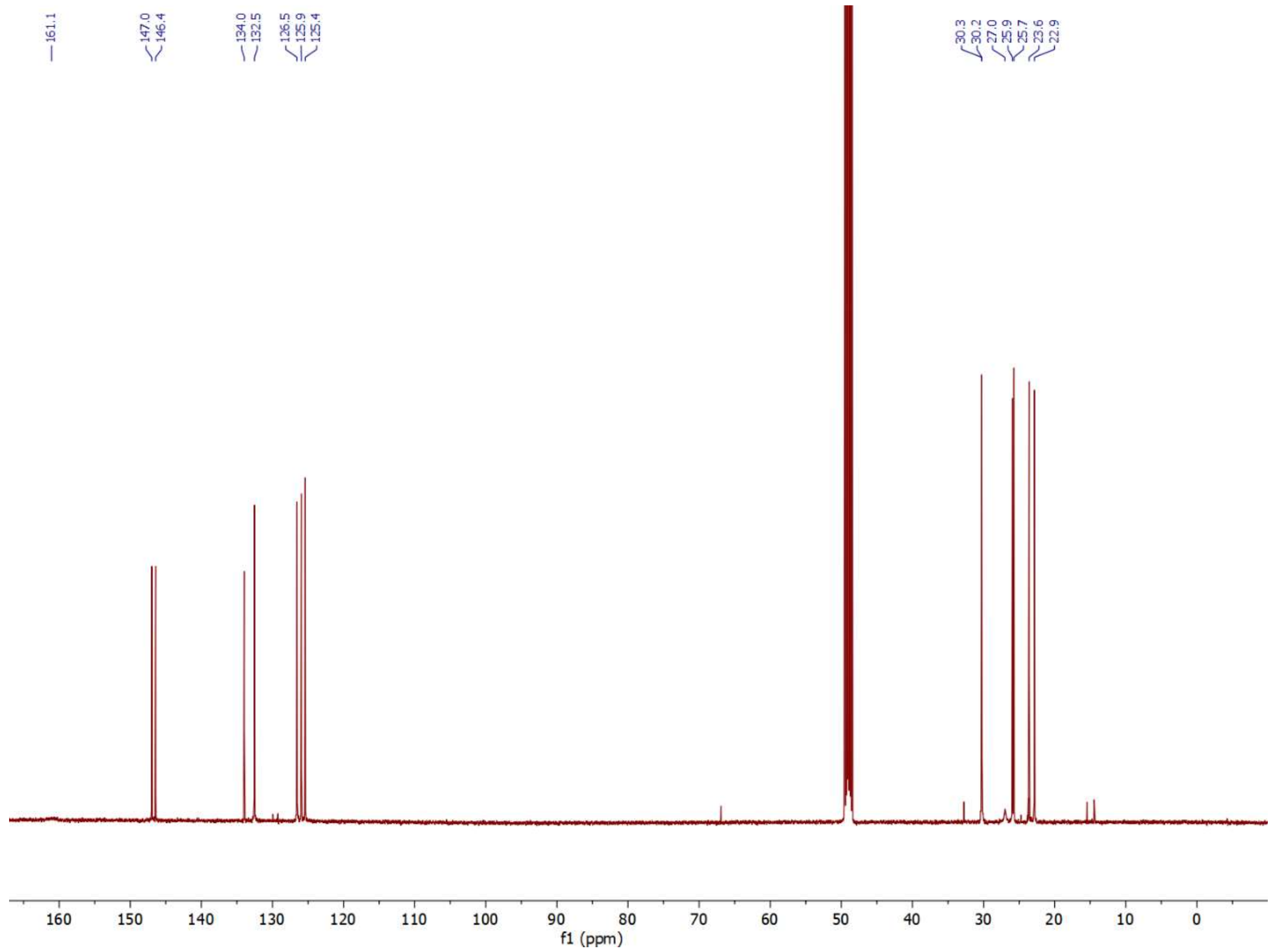

Supplementary Figure S57. The $^{13}C\{^{1}H\}$ (125.8 MHz) NMR spectrum of *o*-2j in CD_3OD.

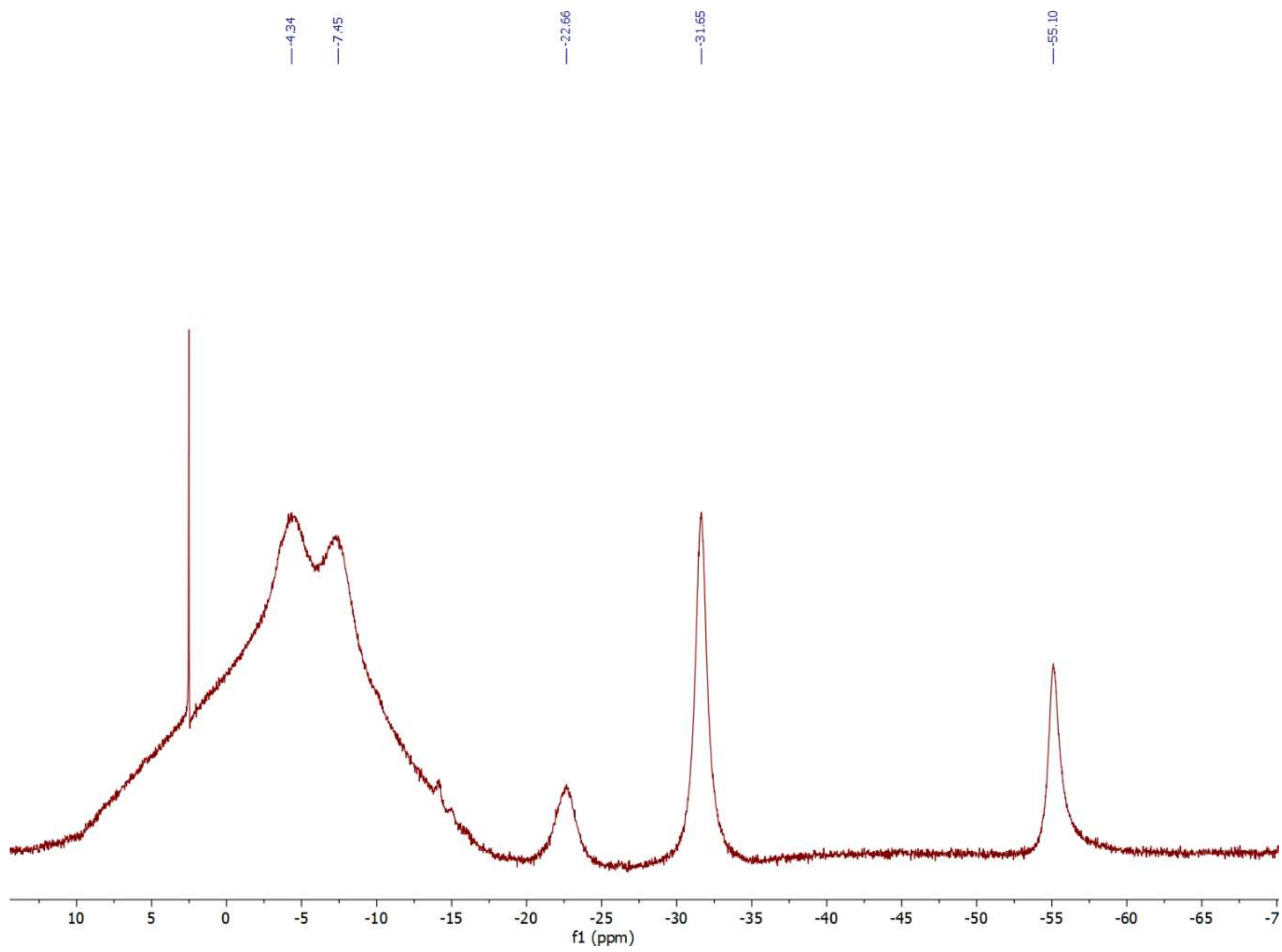

Supplementary Figure S58. The $^{11}B\{^{1}H\}$ (160.4) NMR spectrum of *o*-2j in CD_3OD.

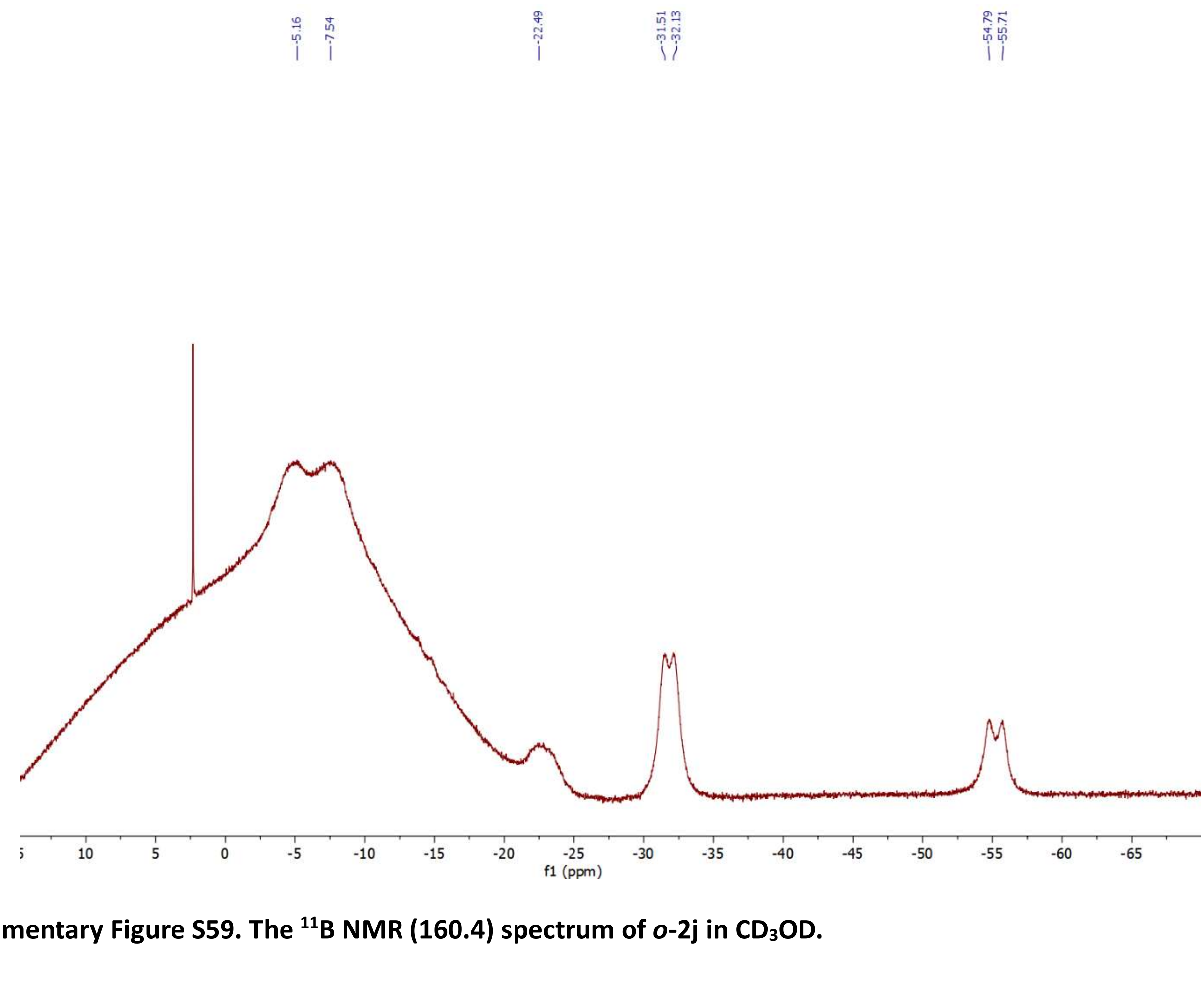

Supplementary Figure S59. The ^{11}B NMR (160.4) spectrum of *o*-2j in CD_3OD.

Reactions with dimethyl malonate and nitromethane

Carborane *o*-**2** (20 mg, 0.022 mmol) was dissolved in deuterated acetonitrile (0.5 ml) and 1/3/10 equivalents of $CH_2(COOMe)_2$ or $MeNO_2$ were added. The $^{11}B\{^1H\}$ NMR spectra (**Supplementary Figures S60–62 and S64–66**) were measured immediately. Next, *o*-**2** (20 mg, 0.022 mmol)) was dissolved in $CH_2(COOMe)_2$ or $MeNO_2$ (0.5 mL) and one drop of deuterated acetonitrile was added. The $^{11}B\{^1H\}$ NMR spectra (**Supplementary Figures S63 and S67**) were measured immediately. Because of the overlaps with the reagent (solvent), ^1H and ^{13}C NMR spectra were not measured.

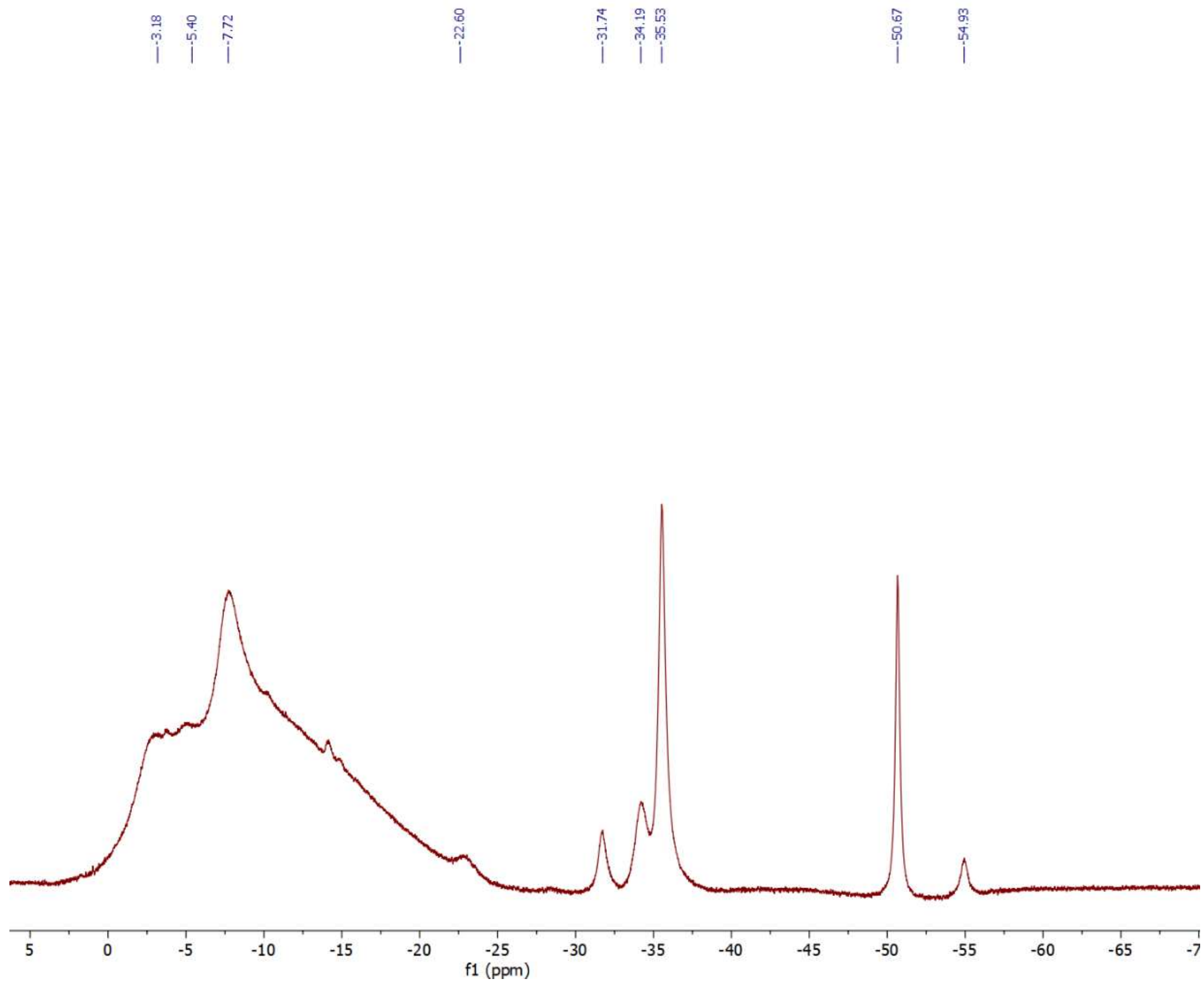

Supplementary Figure S60. The $^{11}B\{^1H\}$ (160.4) NMR spectrum of the reaction mixture of *o*-2 with one equivalent of dimethyl malonate in CD_3CN.

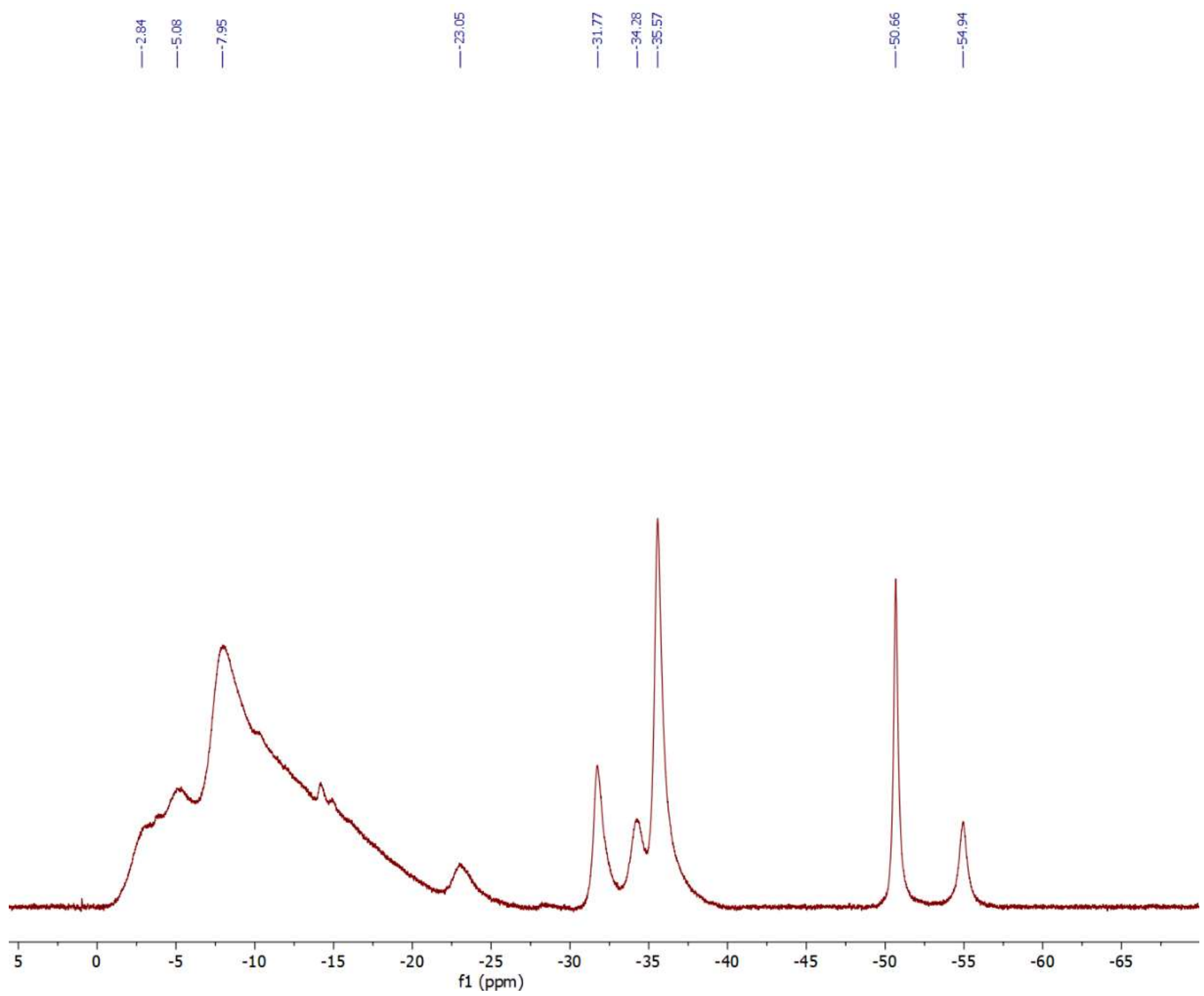

Supplementary Figure S61. The $^{11}B\{^1H\}$ (160.4) NMR spectrum of the reaction mixture of *o*-2 with three equivalents of dimethyl malonate in CD_3CN.

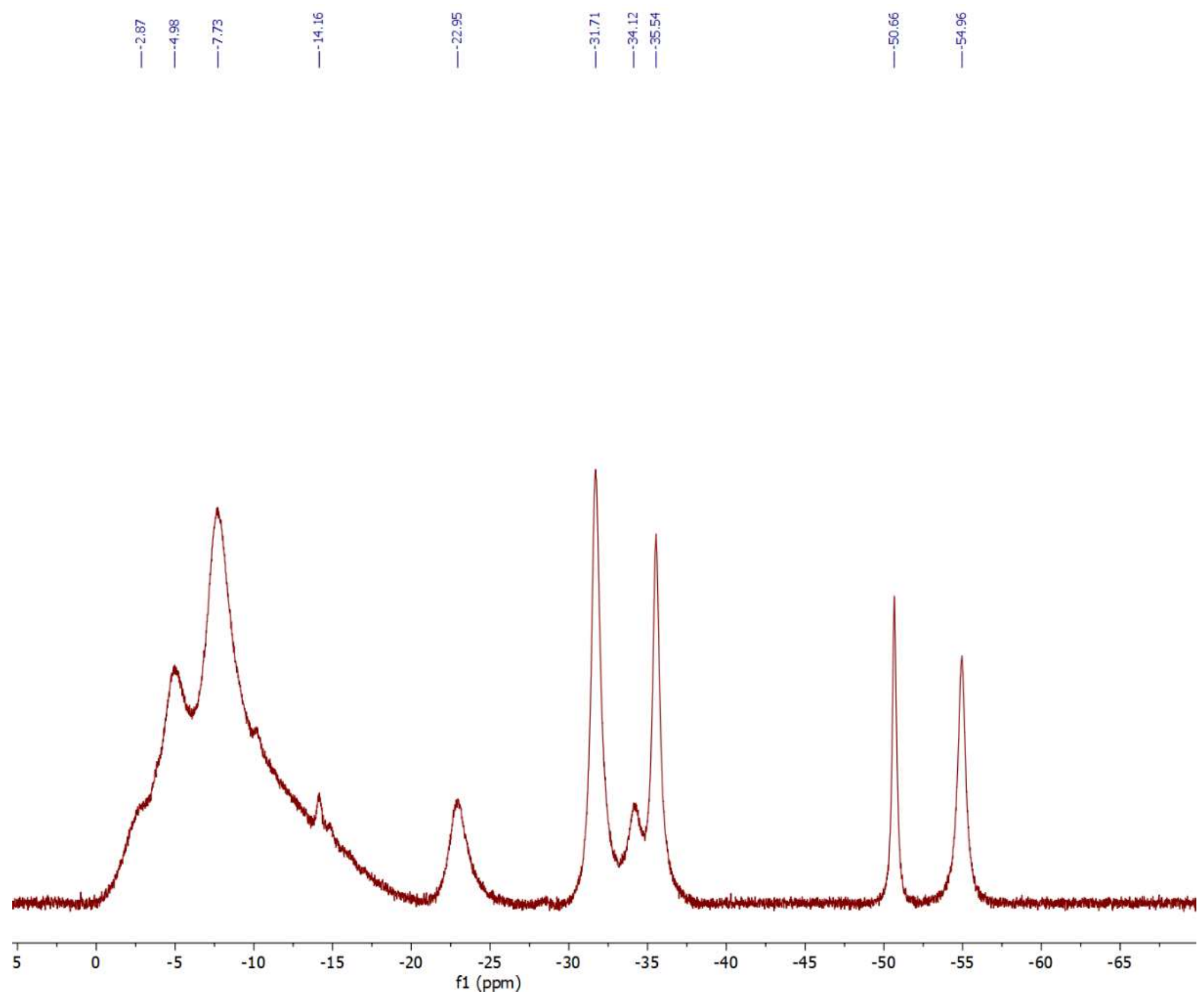

Supplementary Figure S62. The $^{11}B\{^1H\}$ (160.4) NMR spectrum of the reaction mixture of *o*-2 with ten equivalents of dimethyl malonate in CD_3CN.

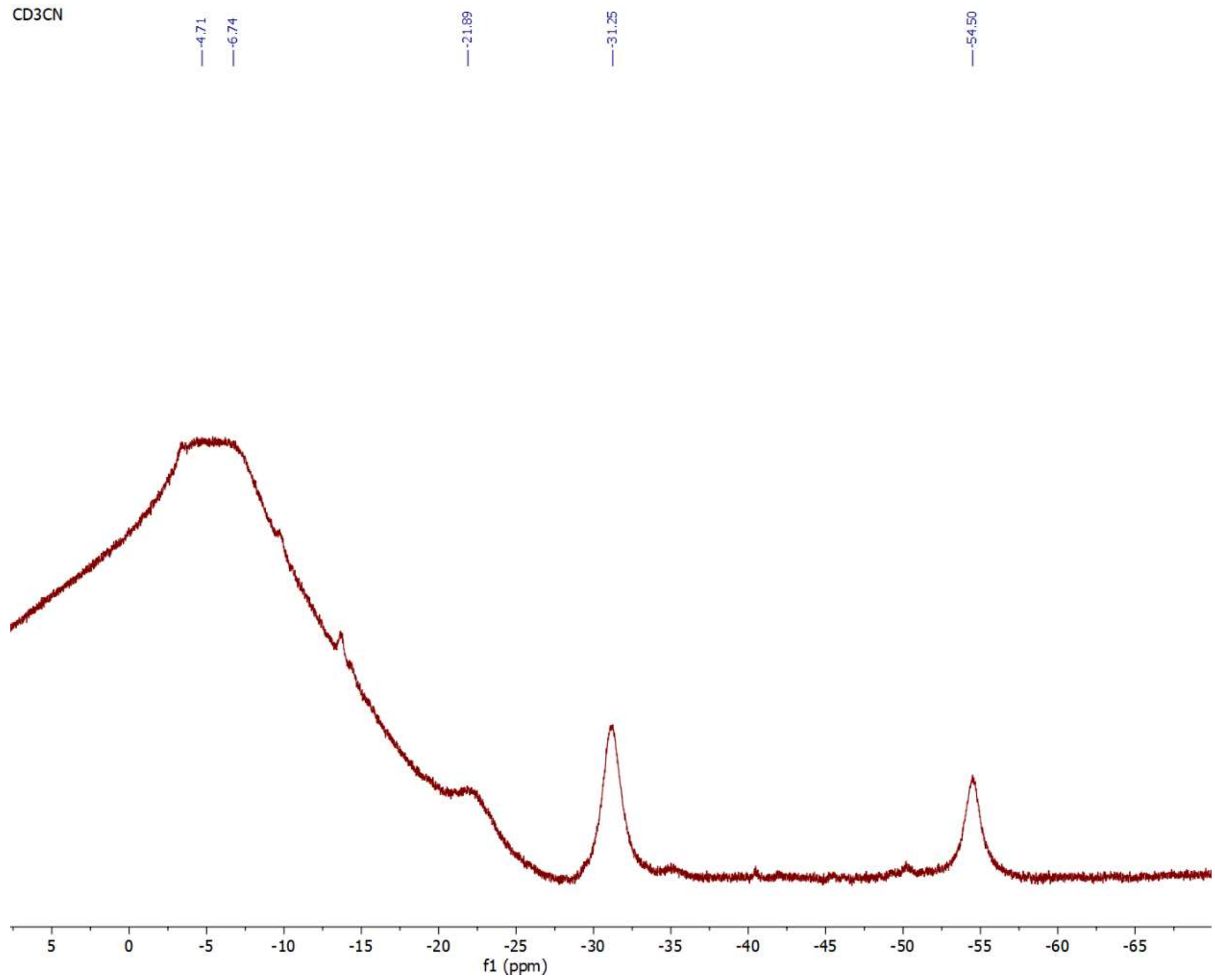

Supplementary Figure S63. The $^{11}B\{^1H\}$ (160.4) NMR spectrum of *o*-2 dissolved in dimethyl malonate (*o*-2k) in CD_3CN.

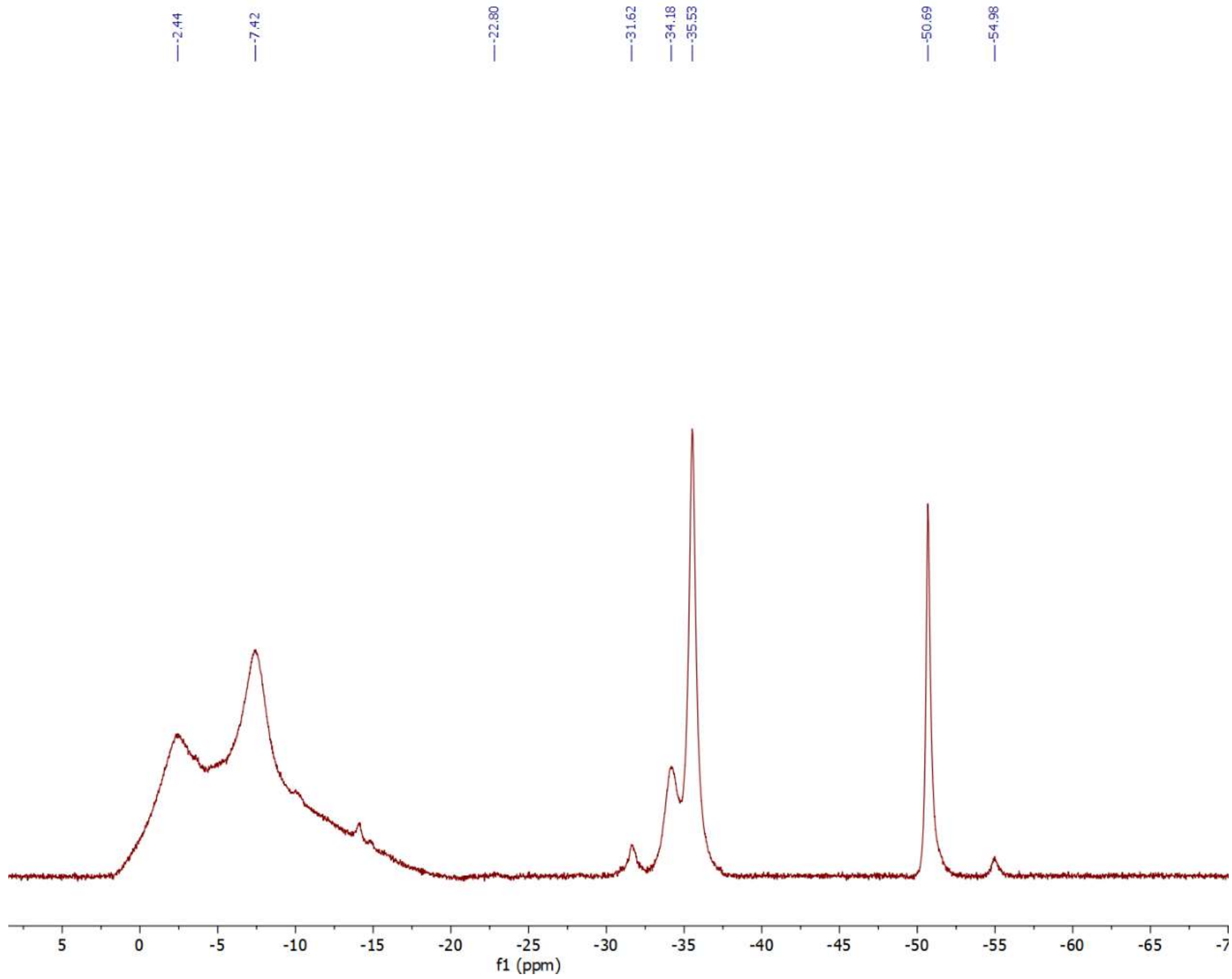

Supplementary Figure S64. The $^{11}B\{^1H\}$ (160.4) NMR spectrum of the reaction mixture of *o*-2 with one equivalent of nitromethane in CD_3CN.

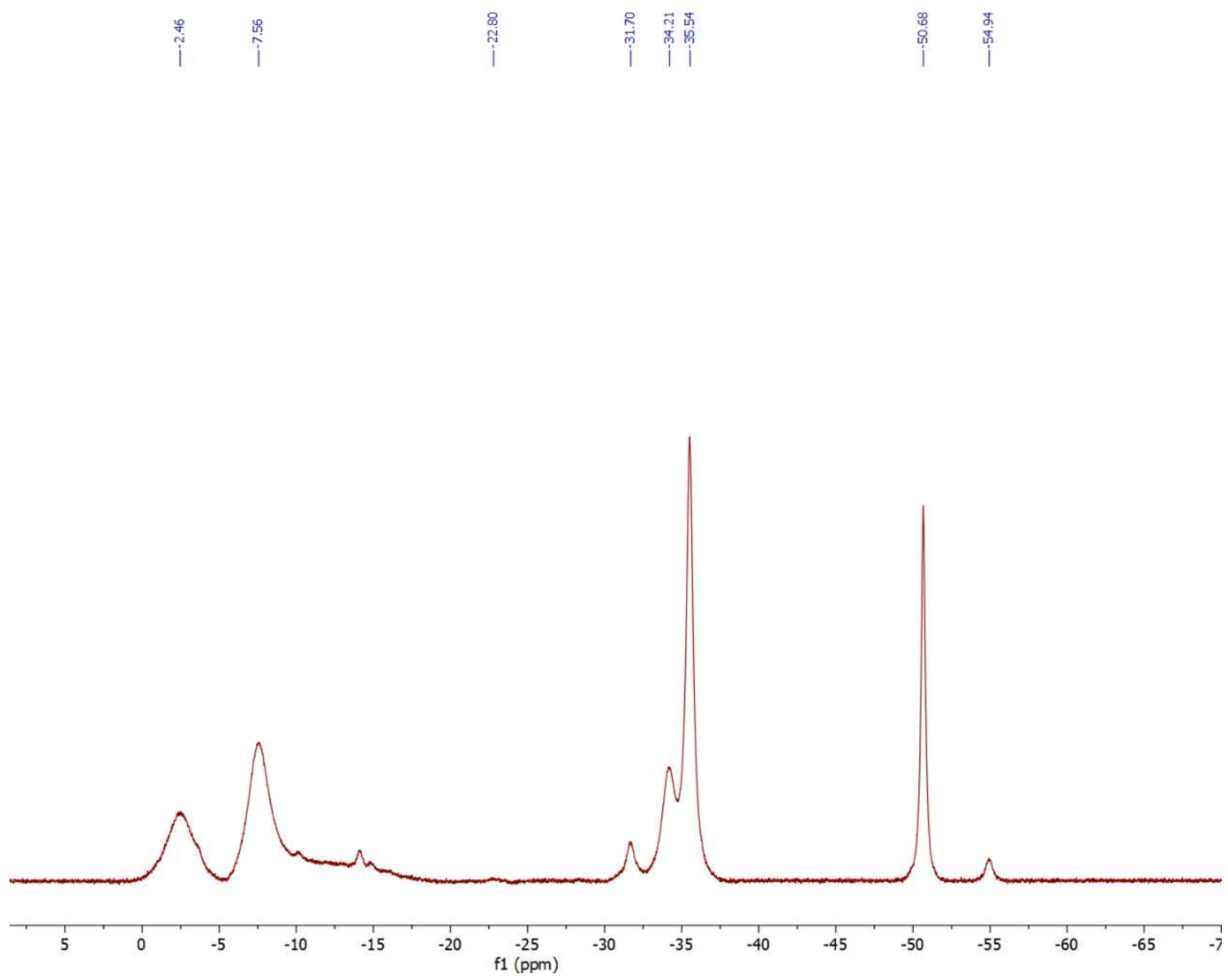

Supplementary Figure S65. The $^{11}B\{^{1}H\}$ (160.4) NMR spectrum of the reaction mixture of *o*-2 with three equivalents of nitromethane in CD_3CN.

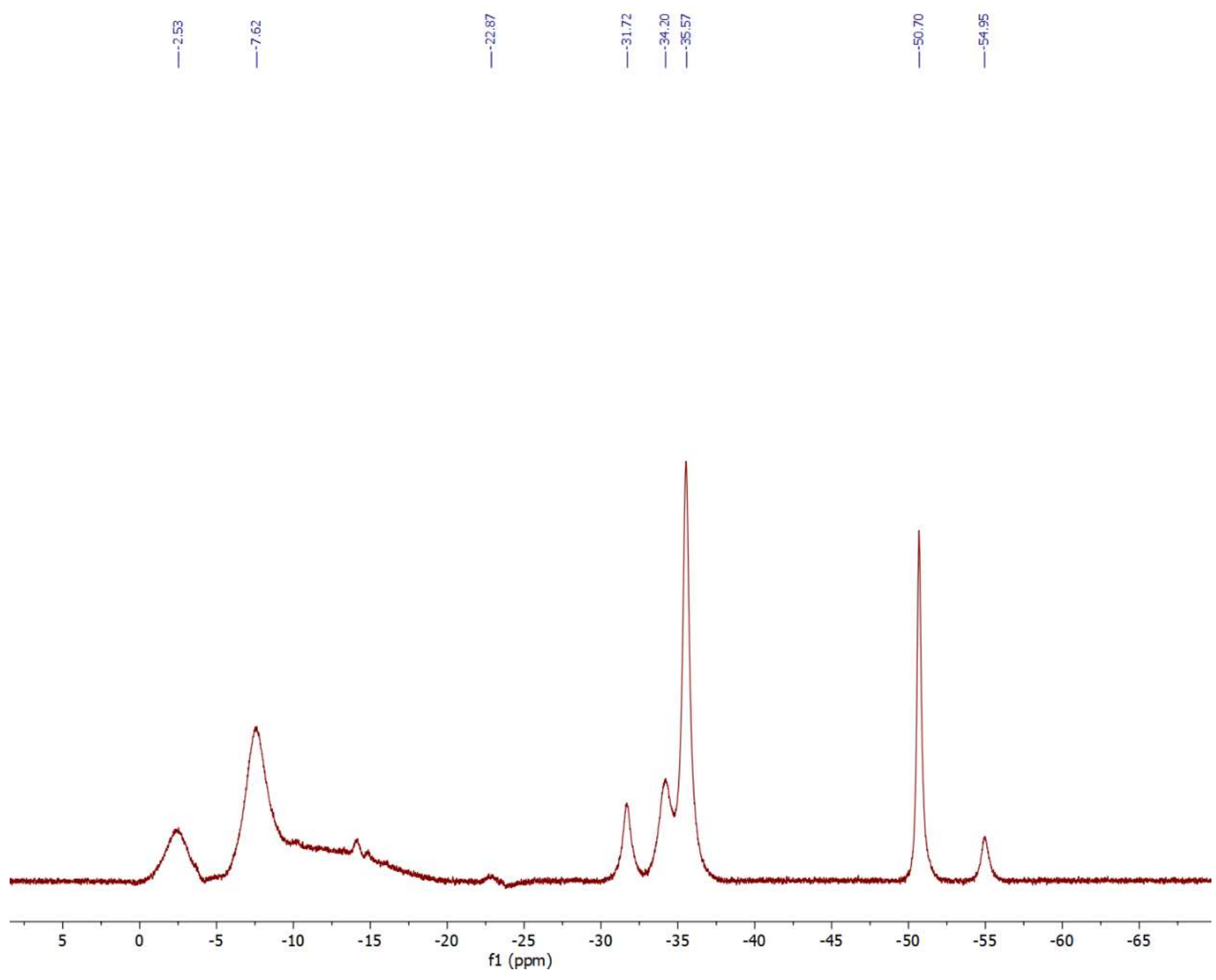

Supplementary Figure S66. The $^{11}B\{^{1}H\}$ (160.4) NMR spectrum of the reaction mixture of *o*-2 with ten equivalents of nitromethane in CD_3CN.

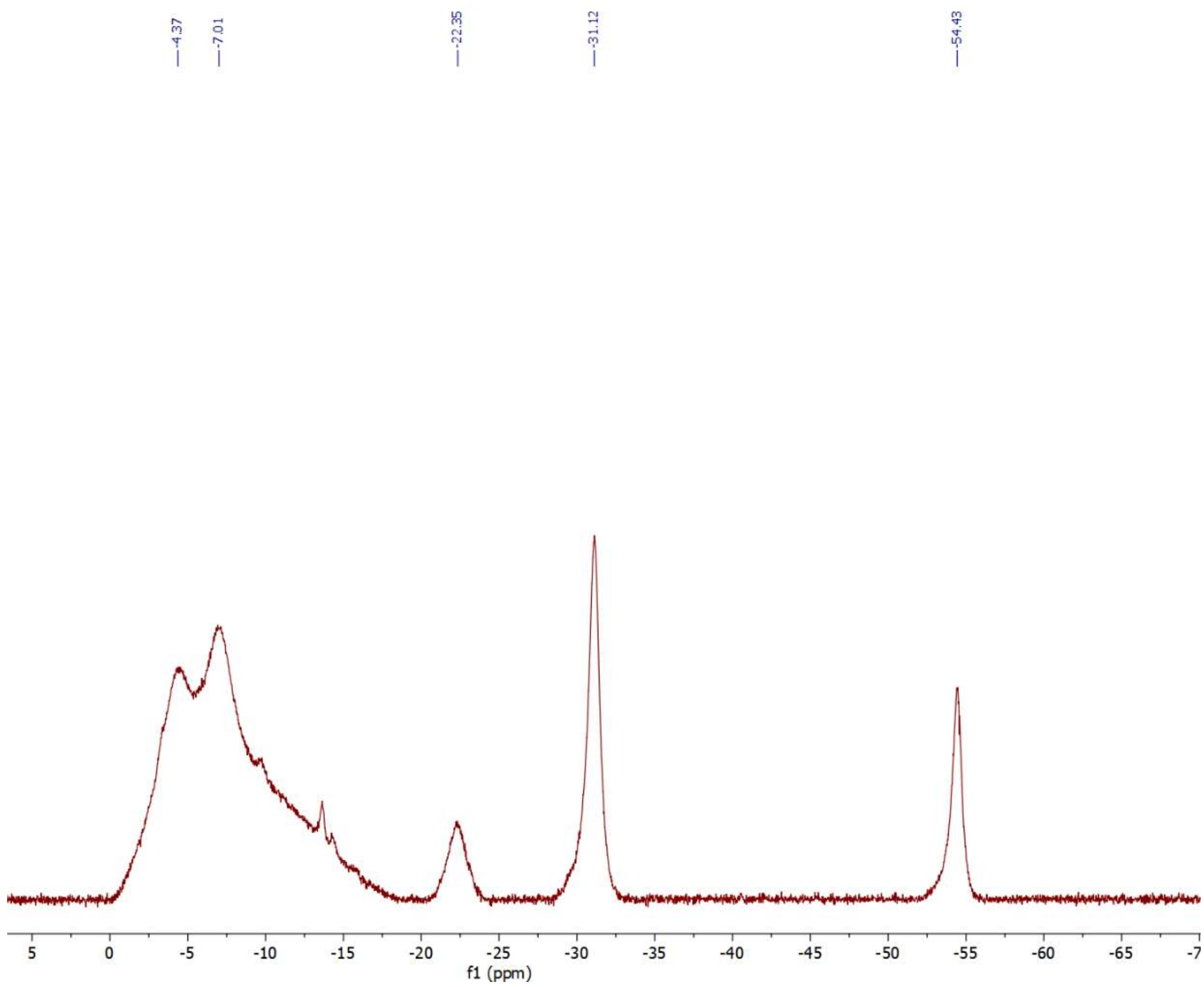

Supplementary Figure S67. The $^{11}B\{^1H\}$ (160.4) NMR spectrum of *o*-2 dissolved in nitromethane (*o*-2l) in CD_3CN.

Synthesis of $[6,9\text{-}IPr_2\text{-}5,10\text{-}C_2B_8H_{10}D]^+OD^-$ (*o*-2w)

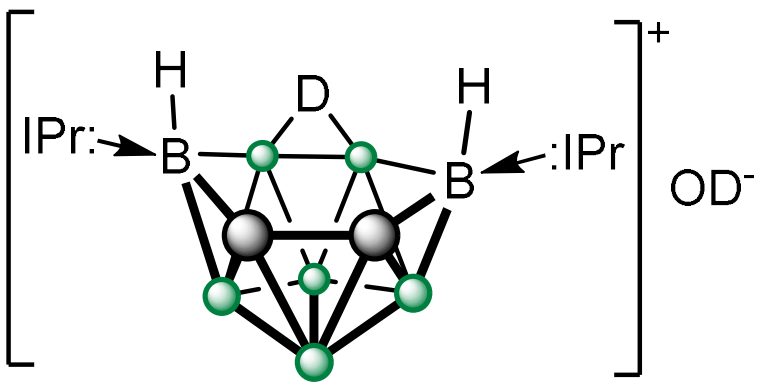

Compound *o*-**2** (15 mg, 0.017 mmol) was dissolved in deuterated acetonitrile (0.25 mL) and D_2O (0.25 mL) was added dropwise while stirring. White precipitate was formed, which dissolves after two hours of stirring. The NMR spectra were measured. **^{1}H NMR** (25 °C, CD_3CN, 500 MHz): δ = 0.50 (s, 2H, BC*H*), 0.98 (d, $^3J(^1H\text{-}^1H)$ = 6.8 Hz, 12H, CH(C*H*$_3$)$_2$), 1.02 (d, $^3J(^1H\text{-}^1H)$ = 6.8 Hz, 12H, CH(C*H*$_3$)$_2$), 1.05 (d, $^3J(^1H\text{-}^1H)$ = 6.8 Hz, 12H, CH(C*H*$_3$)$_2$), 1.09 (d, $^3J(^1H\text{-}^1H)$ = 6.8 Hz, 12H, CH(C*H*$_3$)$_2$), 2.11 (sept, $^3J(^1H\text{-}^1H)$ = 6.8 Hz, 4H, C*H*(CH$_3$)$_2$), 2.31 (sept, $^3J(^1H\text{-}^1H)$ = 6.8 Hz, 4H, C*H*(CH$_3$)$_2$), 7.23 (d, $^3J(^1H\text{-}^1H)$ = 7.7 Hz, 4H, *m*-C$_6$*H*$_3$), 7.25 (d, $^3J(^1H\text{-}^1H)$ = 8.0 Hz, 4H, *m*-C$_6$*H*$_3$), 7.38 (s, 4H, NC*H*), 7.49 (t, $^3J(^1H\text{-}^1H)$ = 7.8 Hz, 4H, *p*-C$_6$*H*$_3$) ppm. **^{13}C{^{1}H} NMR** (25 °C, CD_3CN, 125.76 Hz): δ = 21.5 (s, CH(*C*H$_3$)$_2$), 22.3 (s, CH(*C*H$_3$)$_2$), 24.6 (s, CH(*C*H$_3$)$_2$), 24.8 (s, CH(*C*H$_3$)$_2$), 25.7 (s broad, B*C*H), 28.68 (s, *C*H(CH$_3$)$_2$), 28.69 (s, *C*H(CH$_3$)$_2$), 124.0 (s, *m*-*C*$_6$H$_3$), 124.7 (s, N*C*H), 124.9 (s, N*C*H), 131.3 (s, *p*-*C*$_6$H$_3$), 132.3 (s, *ipso*-*C*$_6$H$_3$), 145.1 (s, *o*-*C*$_6$H$_3$), 145.6 (s, *o*-*C*$_6$H$_3$), 159.0 (s broad, B*C*N) ppm. **^{11}B NMR** (25 °C, CD_3CN, 160.42 MHz): δ = −55.1 (d, $^1J(^{11}B\text{-}^1H,)$ = 147 Hz, 1B, B3), −31.9 (d, $^1J(^{11}B\text{-}^1H,)$ = 118 Hz, 2B, B6,9), −23.2 (s broad, 1B, B1), −8.3 (s broad, 2B, B2,4), −5.3 (s broad, 2B, B7,8) ppm.

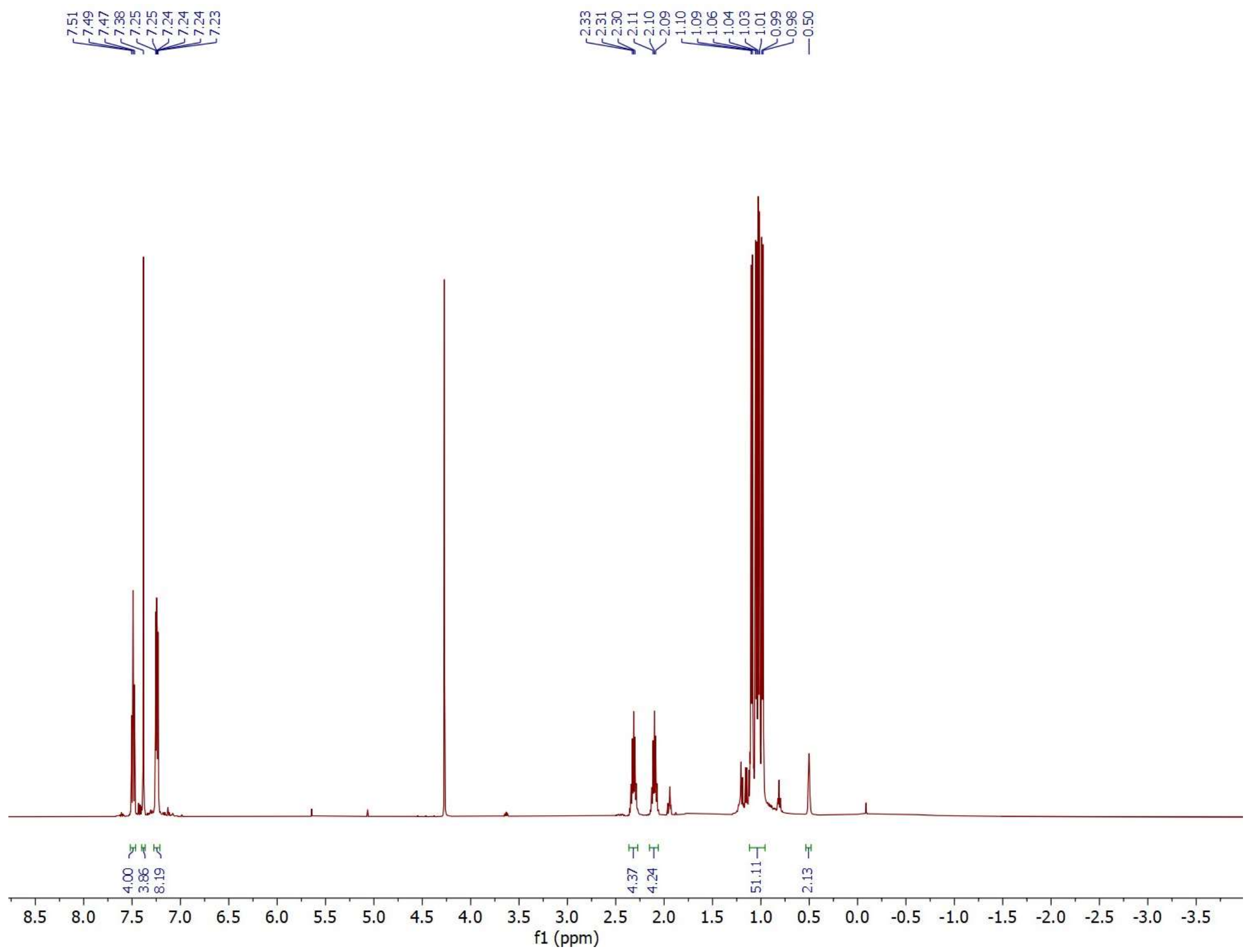

Supplementary Figure S68. The ^{1}H (500.1 MHz) NMR spectrum of *o*-2w in CD_3CN.

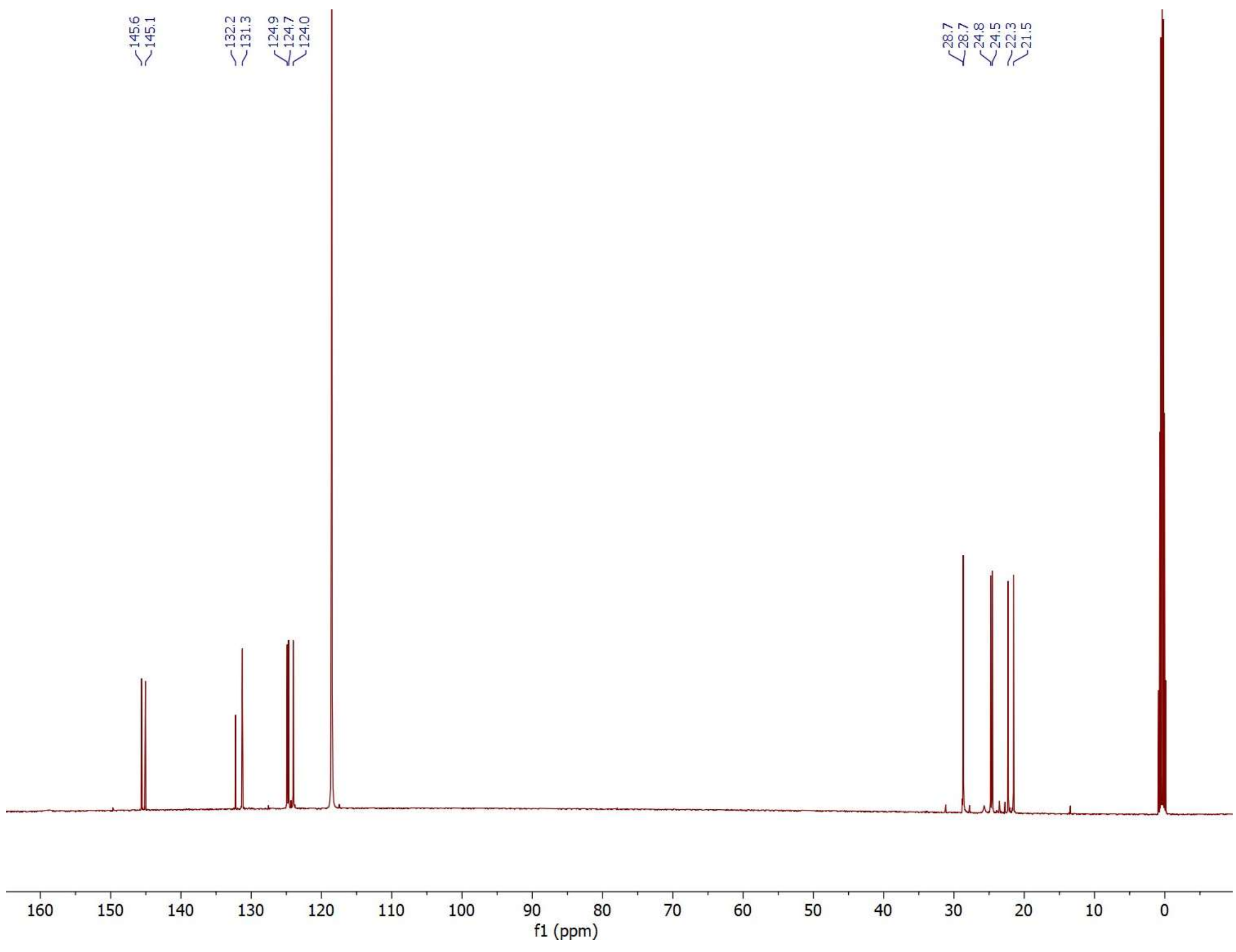

Supplementary Figure S69. The $^{13}C\{^{1}H\}$ (125.8 MHz) NMR spectrum of *o*-2w in CD_3CN.

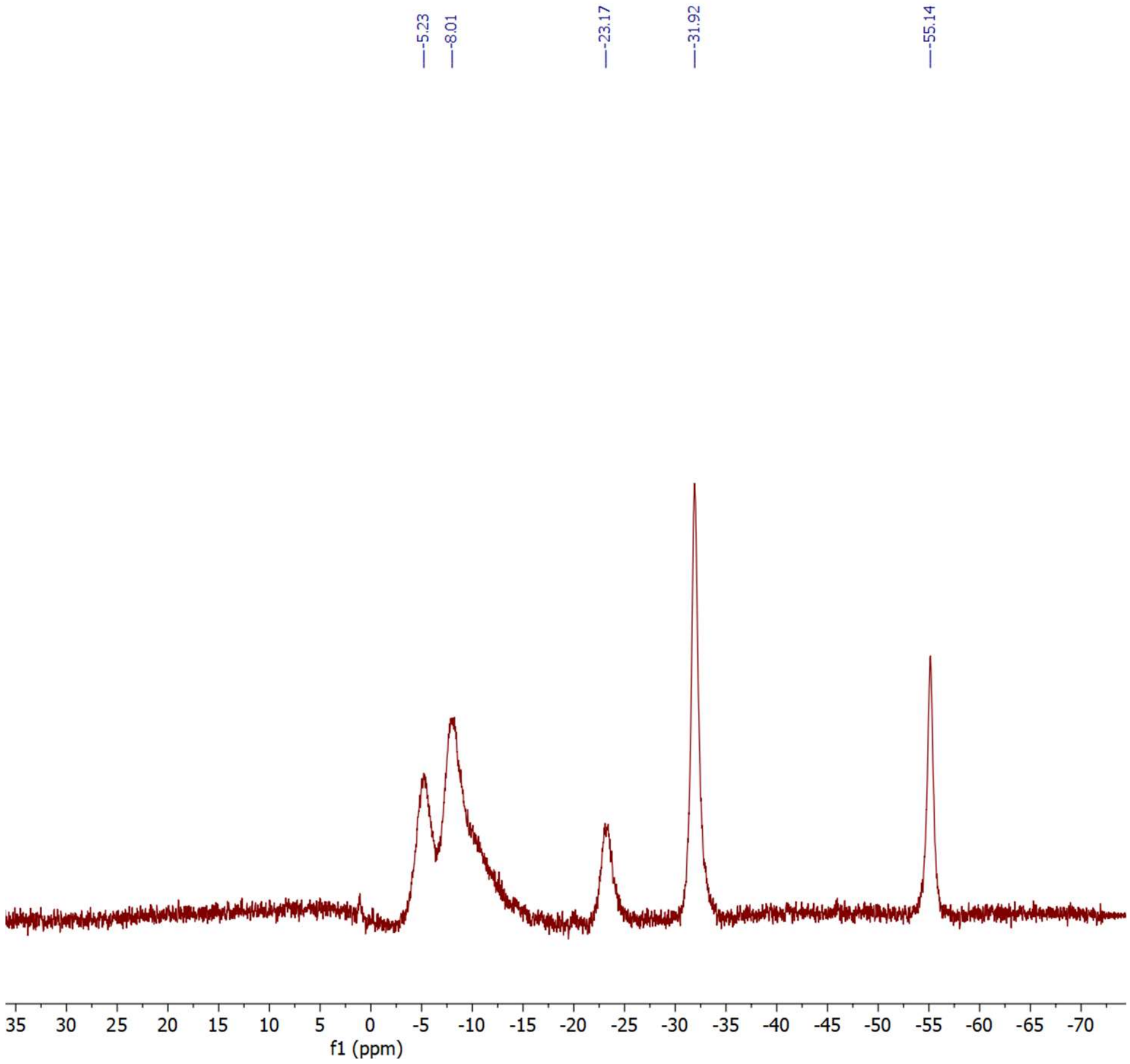

Supplementary Figure S70. The $^{11}B\{^{1}H\}$ (160.4) NMR spectrum of *o*-2w in CD_3CN.

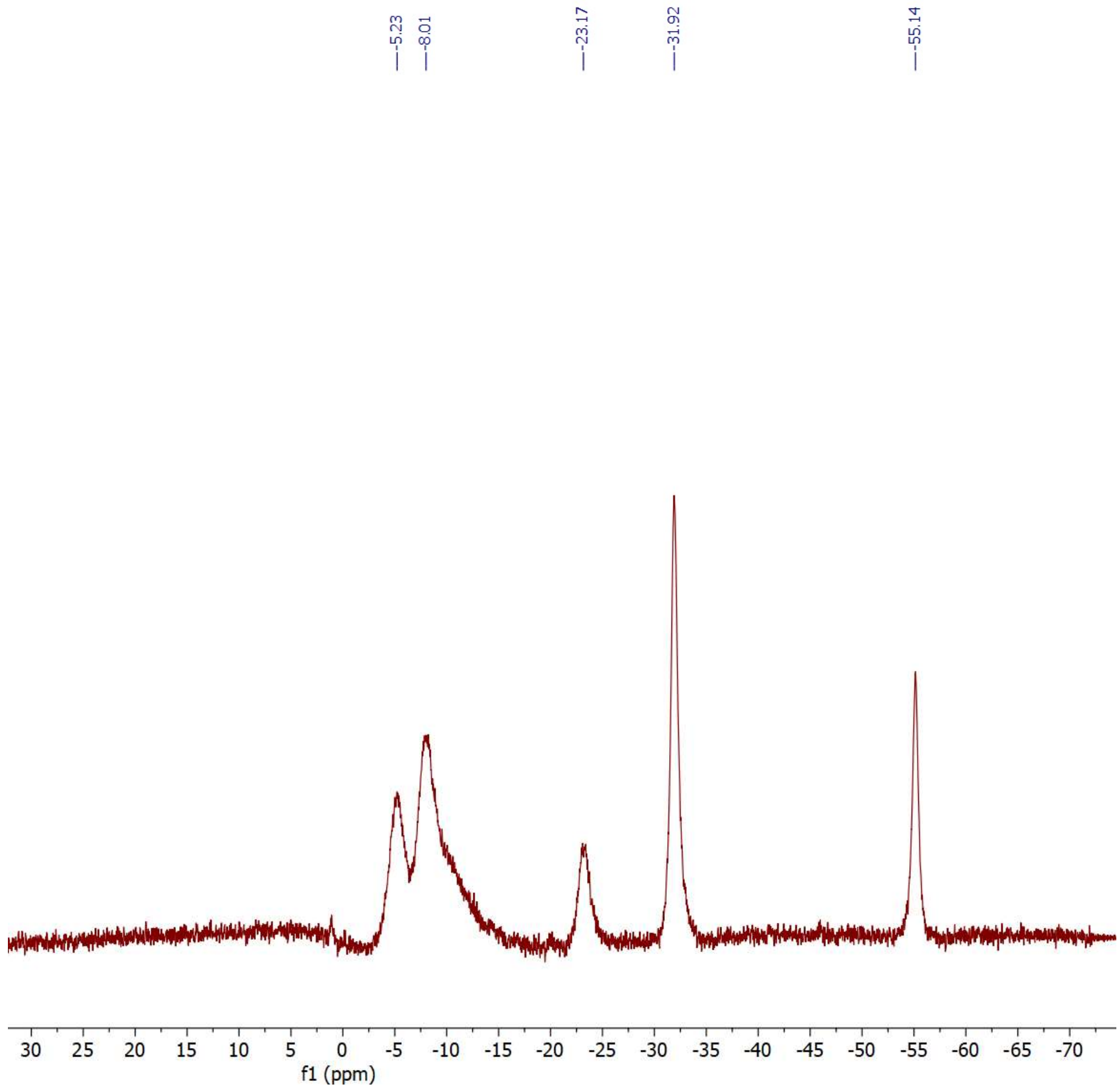

Supplementary Figure S71. The ^{11}B (160.4) NMR spectrum of *o*-2w in CD_3CN.

General procedure of testing the basicity of *o*-2

Deprotonation of *o*-2H+

The solution of *o*-**2a** in deuterated acetonitrile (0.5 mL) was treated by the addition of neat **BTMG**, **MTBD**, **P_1-H** or **P_1-*t*Bu**. The NMR spectra were recorded immediately.

Protonation of *o*-2

The solution of **BTMG**·HCl, **MTBD**H·HCl or **P_1-H**·HCl in deuterated acetonitrile (0.5 mL) were treated by the addition of equimolar amount of *o*-**2**. The NMR spectra were recorded immediately.

Catalytic experiments

Aldol condensation catalysed by carborane *o*-2 (Procedure C)

The catalyst *o*-**2** was introduced into a flame-dried Schlenk tube and dissolved in anhydrous CD_3CN (0.5 ml). Subsequently, butanal (180 µl, 2.0 mmol) was added, followed by the internal standard toluene (35 µl, 0.33 mmol). The reaction mixture was rapidly homogenized before measuring the ^1H NMR spectra to accurately determine the integral ratio between the standard and the substrate. The yield was determined by comparing the integral intensity of the internal standard (toluene, δ 7.11–7.28 ppm) with the signal of the product, 2-ethylhex-2-enal (δ = 9.32 ppm). Yields are based on two independent experiments differing less than 3 %. The analogous reactions catalysed by MTBD and P_1-H were conducted under the same conditions.

Supplementary Table S7. Aldol condensation catalysed by *o*-2, MTBD and P_1-H.

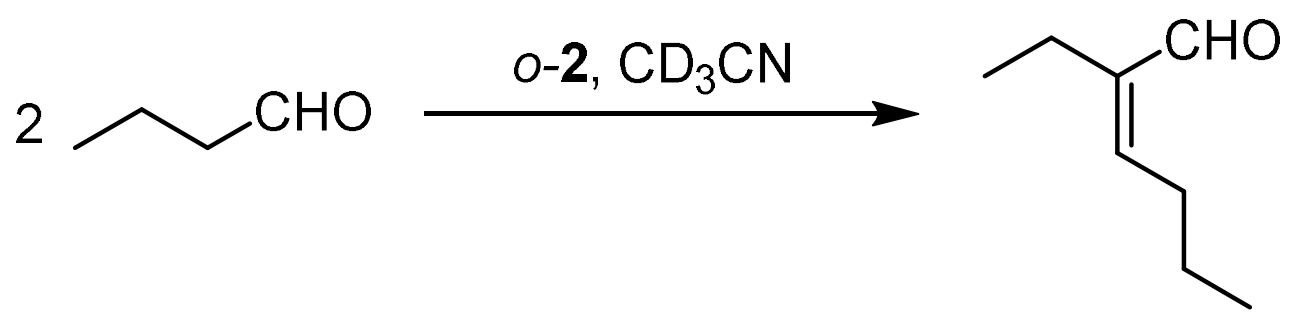

Entry	Catalyst	Cat. loading (mol%)	H_2O (mol%)	Temperature (°C)	Time (h)	Yield (%)[a]
**1**	*o*-**2**	10	0	21	1	<5
**2**	*o*-**2**	10	0	21	24	32
**3**	*o*-**2**	2	0	21	1	24
**4**	*o*-**2**	2	0	21	2	92
**5**	*o*-**2**	1	0	21	2	71
**6**	*o*-**2**	1	0	21	24	97
**7**	*o*-**2**	10	20[b]	21	2	24
**8**	*o*-**2**	10	20[b]	21	24	90
**9**	*o*-**2**	2	20[b]	21	2	32
**10**	*o*-**2**	2	20[b]	21	24	99
**11**	*o*-**2**	2	0	30	1	57
**12**	*o*-**2**	2	0	30	2	95
**13**	**MTBD**	1	0	21	2	<5
**14**	**MTBD**	1	0	21	24	76
**15**	**MTBD**	5	0	21	2	<5
**16**	**MTBD**	5	0	21	24	98
**17**	**P_1-H**	1	0	21	2	<5
**18**	**P_1-H**	1	0	21	24	<5
**19**	**P_1-H**	5	0	21	2	25
**20**	**P_1-H**	5	0	21	24	97

[a] determined by ^1H NMR; [b] water was added with the catalyst

Cross-aldol condensation of butanal and benzaldehyde (Procedure D)

The appropriate amount of catalyst *o*-**2** was introduced into a flame-dried Schlenk tube followed by the addition of anhydrous CD_3CN (0.50 ml). The resulting catalyst solution was then transferred into a flame-dried NMR tube. Subsequently, butanal (90 µl, 1.0 mmol) was added, followed by benzaldehyde (112 µl, 1.1 mmol). The reaction mixture was rapidly homogenized, transferred into an oil bath and heated at required temperature for required time. Then, the ^{1}H NMR spectrum was acquired. Conversion was determined by comparing the integral intensity of the CHO groups of butanal (δ = 9.68 ppm) with the signal of the product (δ = 9.52 ppm) and 2-ethylhex-2-enal (δ = 9.32 ppm). Conversion and selectivity were based on two independent experiments differing by less than 3 %.

Supplementary Table S8. Cross-aldol condensation catalysed by the *o*-2.

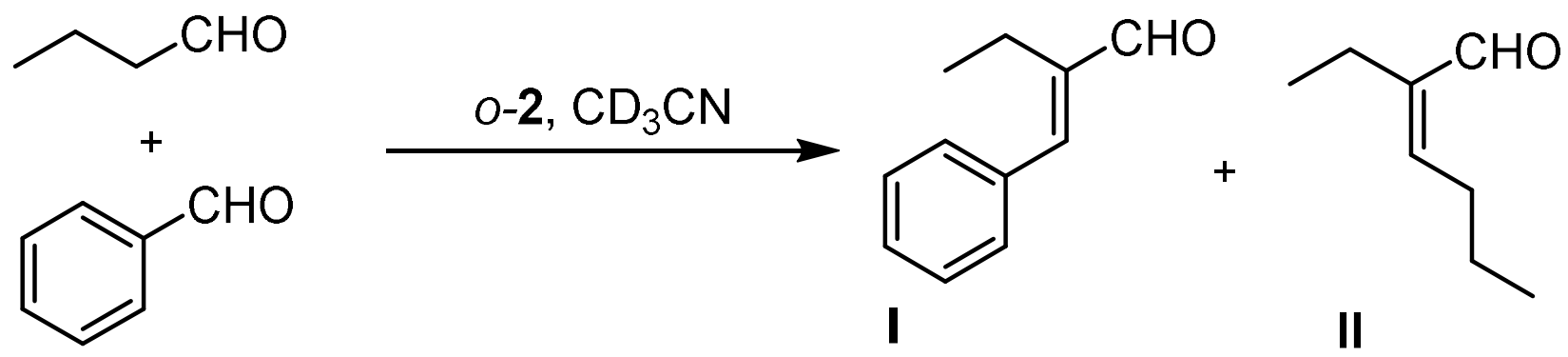

Entry	Cat. loading (mol%)	Temperature (°C)	Time (h)	Conversion[a]	I:II[a]
**1**	2	50	3	99	69:31
**2**	1	50	3	96	65:35
**3**	1	30	2	80	71:29
**4**	1	30	20	98	73:27

[a] determined by ^{1}H NMR

Knoevenagel condensation catalysed by carborane *o*-2, MTBD and P_1-H (Procedure E)

In secured Schlenk flask, malononitrile (3 mmol; 208 mg) was dissolved in CD3CN (0.5 ml) before addition of benzaldehyde (3 mmol; 304 µl). After mixing, the base (5, 10 or 20 mol%) was added and vigorously stirred for 10 sec before transfer of the reaction mixture to the NMR tube. NMR yield is based on integration of CHO signals of starting benzaldehyde and PhCH signal of product (and/or CH_2CN signals of side product in the case MTBD and P_1-H catalysts).

Supplementary Table S9. Knoevenagel condensation catalysed by *o*-2, MTBD and P_1-H.

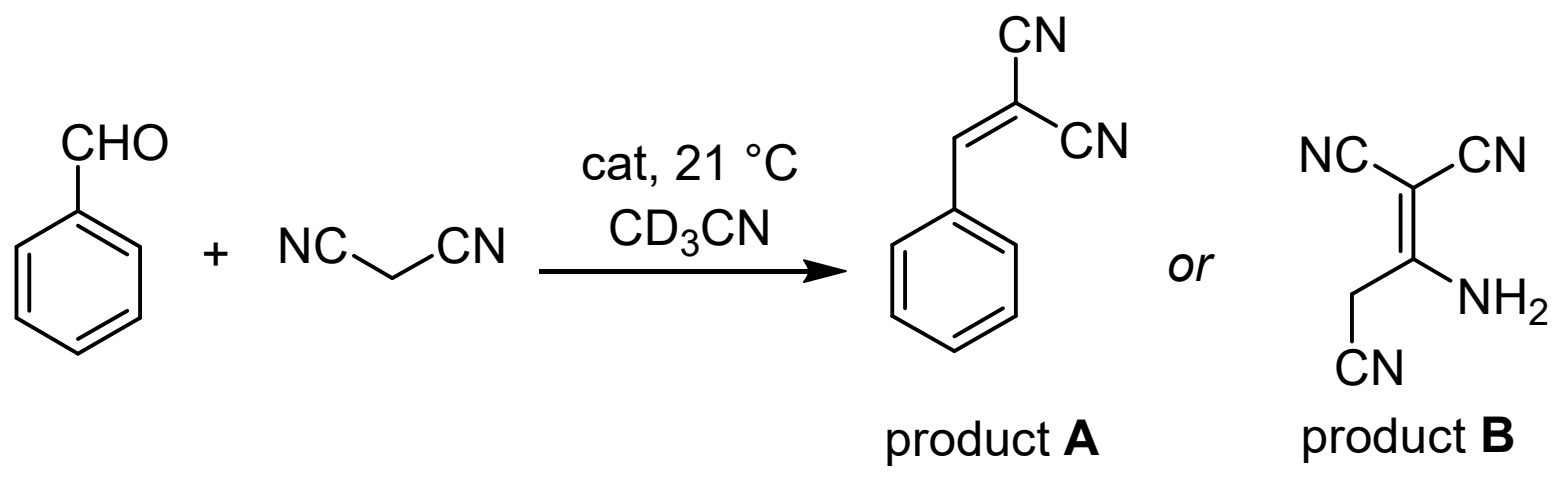

Entry	Catalyst	Cat. loading (mol%)	Conversion[a] (%)	Time (h)	A/B ratio[a] (%)
**1**	***o*-2**	0.5	60	24	60/0
**2**	***o*-2**	0.5	90	48	90/0
**3**	***o*-2**	1	21	2	65/0
**4**	***o*-2**	1	99	16	99/0
**5**	***o*-2**	3	79	2	79/0
**6**	***o*-2**	3	97	6	97/0
**7**	***o*-2**	5	99	2	99/0
**8**	**MTBD**	5	56	2	53/3
**9**	**MTBD**	5	94	24	89/5
**10**	**MTBD**	10	61	1	56/5
**11**	**MTBD**	10	65	2	59/6
**12**	**MTBD**	20	97	1	87/10
**13**	**MTBD**	20	88	2	78/10
**14**	**P_1-H**	10	85	1	76/9
**15**	**P_1-H**	10	91	2	79/12
**16**	**P_1-H**	20	80	1	60/20
**17**	**P_1-H**	20	82	2	62/20

[a] determined by ^{1}H NMR

Michael reaction catalysed by carborane *o*-2 (Procedure F)

General procedure for the catalytic Michael addition

The carborane *o*-**2** (1–4 mol%) was dissolved in 0.5 ml of dried solvent (CD_3CN, CD_2Cl_2, THF-d_8 or DMSO-d_6). Subsequently, the Michael donor (n eq) and the corresponding Michael acceptor (1.0 eq) were added. The reaction progress was monitored by ^{1}H-NMR analysis in the time intervals from 1 h to 7 d at the given temperature.

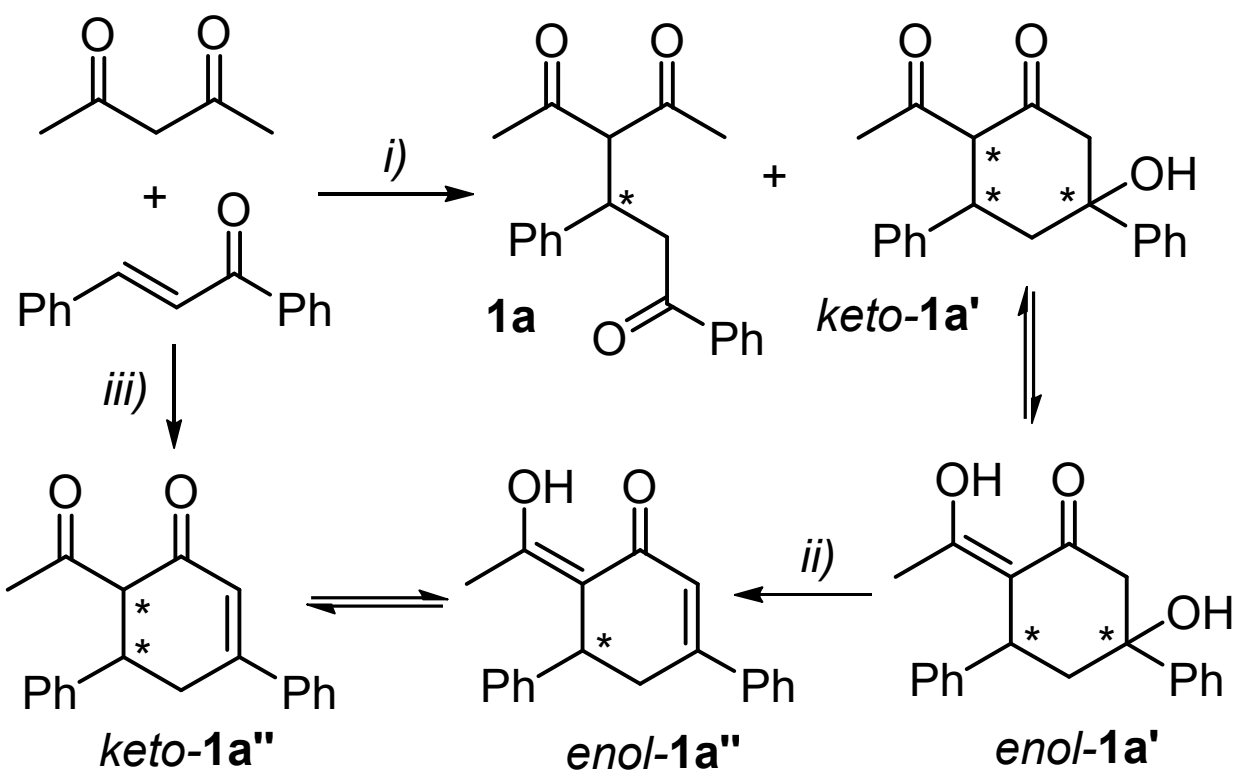

Supplementary Figure S72. Addition of acetylacetone to *trans*-chalcone. The model Michael addition of acetylacetone to trans-chalcone and subsequent dehydration to the Robinson annulation product *keto*-1a''. Conditions: i) 1–4 mol. % *o*-2; ii) 80 °C, two days, or I_2 (1.1 eq), RT, two days; iii) 1–4 mol. %, 80 °C, two days.

Supplementary Table S10. Optimization of the catalyst loading.

n (eq)	*o*-**2** (mol%)	Solvent	T [°C]	Time	1a/1a’ [a]	Conversion of *trans*-chalcone[a]
1.0	1	CD_3CN	25	1 h	100/0	23%
1.0	1	CD_3CN	25	3 h	95/5	48%
1.0	2	CD_3CN	25	1 h	91/9	53%
1.0	2	CD_3CN	25	3 h	84/16	72%
1.0	3	CD_3CN	25	1 h	84/16	70%
1.0	3	CD_3CN	25	3 h	71/29	77%
1.0	3	CD_3CN	25	3 d	9/91	99%
1.5	3	CD_3CN	25	3 h	90/10	70%
1.5	3	CD_3CN	25	1 d	66/44	90%
1.0	4	CD_3CN	25	3 h	49/51	75%

Note: [a]The final composition of the crude mixture (in the form of molar ratio **1a**/**1a’**) and conversion of *trans*-chalcone (according to the catalyst *o*-**2** as an internal standard) was determined using ^{1}H-NMR analysis.

Supplementary Table S11. Optimization of the reaction temperature.

n (eq)	*o*-**2** (mol%)	Solvent	T [°C]	Time	1a/1a' [a]	Conversion of *trans*-chalcone[a]
1.0	3	CD_3CN	30	1 h	89/11	67%
1.0	3	CD_3CN	30	3 h	73/27	79%
1.0	3	CD_3CN	30	1 d	22/78	79%
1.0	3	CD_3CN	40	1 h	77/23	78%
1.0	3	CD_3CN	40	3 h	51/49	81%
1.0	3	CD_3CN	40	1 d	18/82	91%
1.0	3	CD_3CN	50	1 h	54/46	88%
1.0	3	CD_3CN	50	3 h	31/69	90%
1.0	3	CD_3CN	50	1 d	17/83	94%
1.0	3	CD_3CN	60	1 h	37/63	85%
1.0	3	CD_3CN	60	3 h	26/74	85%
1.0	3	CD_3CN	60	1 d	27/73	86%

Note: [a]The final composition of the crude mixture (in the form of molar ratios **1a**/**1a'**) and conversion of *trans*-chalcone (according to the catalyst *o*-**2** as an internal standard) was determined using ^{1}H-NMR analysis.

Supplementary Table S12. Optimization of the solvent.

n (eq)	*o*-**2** (mol%)	Solvent	T [°C]	Time	1a/1a' [a]	Conversion of *trans*-chalcone[a]
1.0	3	CD_2Cl_2	25	2 h	98/2	31%
1.0	3	CD_2Cl_2	25	5 h	94/6	44%
1.0	3	CD_2Cl_2	25	1 d	91/9	61%
1.0	3	THF-d_8	25	2 h	65/35	68%
1.0	3	THF-d_8	25	5 h	55/45	78%
1.0	3	THF-d_8	25	1 d	23/77	88%
1.0	3	DMSO-d_6	25	2 h	94/6	48%
1.0	3	DMSO-d_6	25	5 h	88/12	75%
1.0	3	DMSO-d_6	25	1 d	39/61	89%

Note: [a]The final composition of the crude mixture (in the form of molar ratios **1a**/**1a'**) and conversion of *trans*-chalcone (according to the catalyst *o*-**2** as an internal standard) was determined using ^{1}H-NMR analysis.

4-acetyl-1,3-diphenylhexane-1,5-dione (1a)

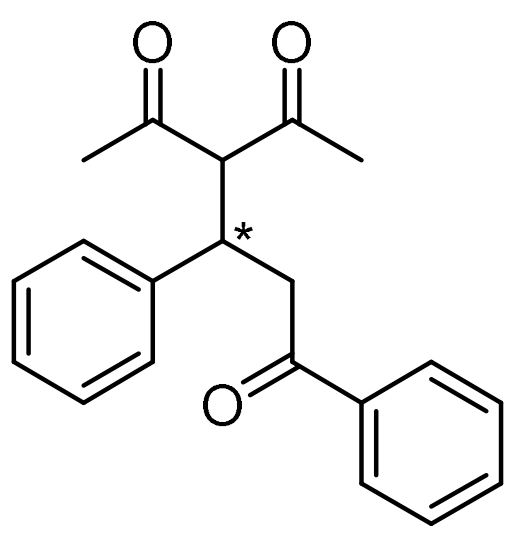

The compound **1a**, prepared by **Procedure F**, was isolated after FLC (SiO_2, eluent: Hex/EtOAc = 2/1) and subsequent crystallization from Hex/EtOAc. The spectral data are in agreement with those reported in the literature.[S11] The literature reports a ^{1}H-NMR ($CDCl_3$, 300 MHz) spectrum of the compound **1a**, keto/enol mixture of 2-acetyl-5-hydroxy-5-(4-nitrophenyl)-3-phenylcyclohexan-1-one and its isolated enol form (27 mg, 5%), which were used as a reference for our structural analysis. **^{1}H NMR** (25 °C, CD_3CN, 500 MHz): δ = 7.84 (d, $J(^1H-^1H)$ = 7.70 Hz, 2H), 7.57 (t, $J(^1H-^1H)$ = 7.35 Hz, 1H), 7.45 (t, $J(^1H-^1H)$ = 7.70 Hz, 2H), 7.20–7.26 (m, 4H), 7.13–7.18 (m, 1H), 4.45 (d, $J(^1H-^1H)$ = 11.0 Hz, 1H), 4.05

(ddd, $J(^1H-^1H)$ = 11.0 Hz, $J'(^1H-^1H)$ = 10.6 Hz, $J''(^1H-^1H)$ = 3.6 Hz, 1H), 3.42 (dd, $J(^1H-^1H)$ = 16.5 Hz, $J'(^1H-^1H)$ = 10.6 Hz, 1H), 3.14 (dd, $J(^1H-^1H)$ = 16.5 Hz, $J'(^1H-^1H)$ = 3.6 Hz, 1H), 2.23 (s, 3H), 1.85 (s, 3H) ppm.

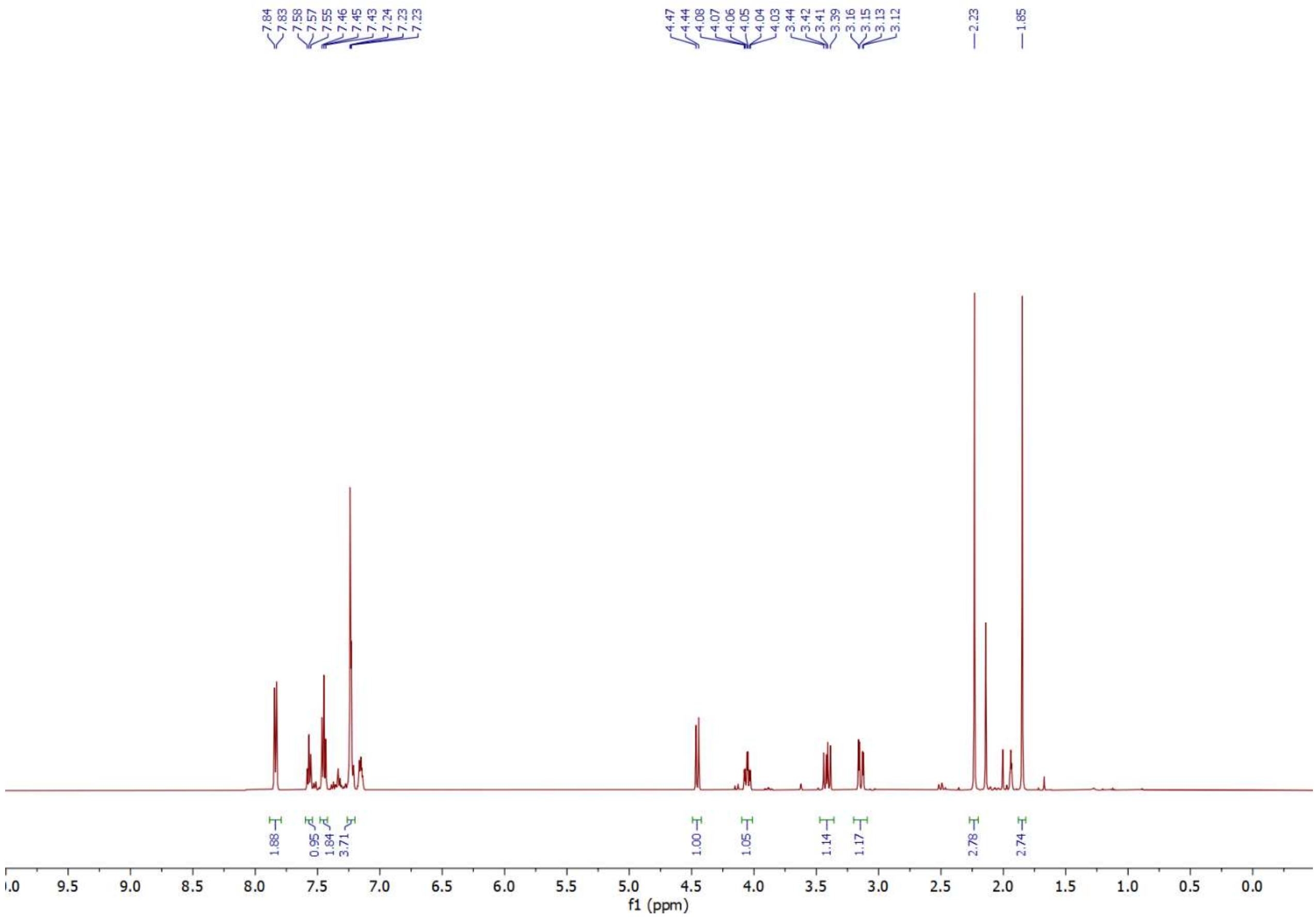

Supplementary Figure S73. ^{1}H (500.1 MHz) NMR spectrum of 1a.

2-acetyl-5-hydroxy-3,5-diphenylcyclohexan-1-one (*keto*-1a')

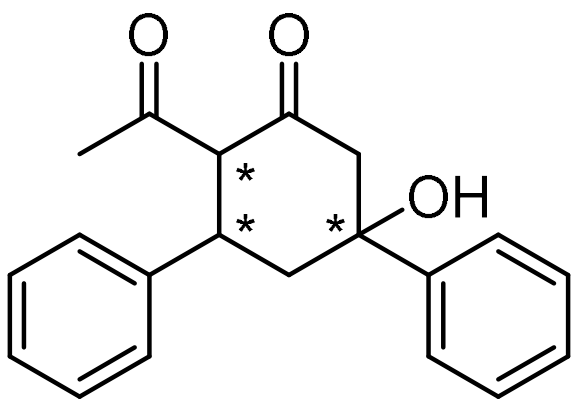

Compounds was prepared by **Procedure F**, **^{1}H NMR** (25 °C, CD_3CN, 400 MHz): δ = 7.55–7.19 (m, 10H), 4.14 (d, $J(^1H-^1H)$ = 12.3 Hz, 1H), 3.89 (ddd, $J(^1H-^1H)$ = 13.0 Hz, $J'(^1H-^1H)$ = 12.4 Hz, $J''(^1H-^1H)$ = 3.9 Hz, 1H), 3.15 (d, $J(^1H-^1H)$ = 13.9 Hz, 1H), 2.45–2.54 (m, 2H), 2.06 (ddd, $J(^1H-^1H)$ = 13.9 Hz, $J'(^1H-^1H)$ = 3.9 Hz, $J''(^1H-^1H)$ = 2.6 Hz, 1H), 2.01 (s, 3H) ppm. The compound *keto*-**1a'** was not isolated. Its ^{1}H NMR spectrum was assigned based on the analysis of the crude product. When the chromatography isolation is performed, the yield decreased to 23% (116 mg) due to the enolization during the separation.

(*Z*)-5-hydroxy-2-(1-hydroxyethylidene)-3,5-diphenylcyclohexan-1-one (*enol*-1a')

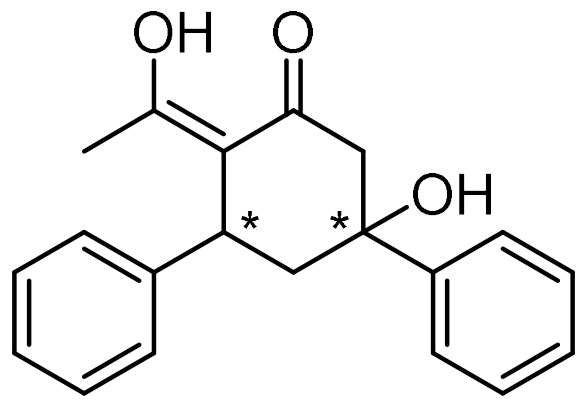

The compound *enol*-**1a'**, prepared by **Procedure F**, was isolated after FLC (SiO_2, eluent: Hex/EtOAc = 2/1) and subsequent crystallization from Hex/EtOAc (130mg, 25%).**^{1}H NMR** (25 °C, CD_3CN, 500 MHz): δ = 7.49 (d, $J(^{1}H-^{1}H)$ = 7.70 Hz, 2H), 7.30–7.37 (m, 4H), 7.20–7.30 (m, 4H), 4.13 (dd, $J(^{1}H-^{1}H)$ = 11.0 Hz, $J'(^{1}H-^{1}H)$ = 6.50 Hz, 1H), 3.52 (s, 1H), 3.06 (d, $J(^{1}H-^{1}H)$ = 18.1 Hz, 1H), 2.51 (dd, $J(^{1}H-^{1}H)$ = 18.1 Hz, $J'(^{1}H-^{1}H)$ = 3.30 Hz, 1H), 2.23 (ddd, $J(^{1}H-^{1}H)$= 13.8 Hz, $J'(^{1}H-^{1}H)$ = 6.50 Hz, $J''(^{1}H-^{1}H)$ = 3.30 Hz, 1H), 1.91–2.03 (m, 1H), 1.68 (s, 3H) ppm.

6-acetyl-3,5-diphenyl-2-cyclohexen-1-one (1a'')

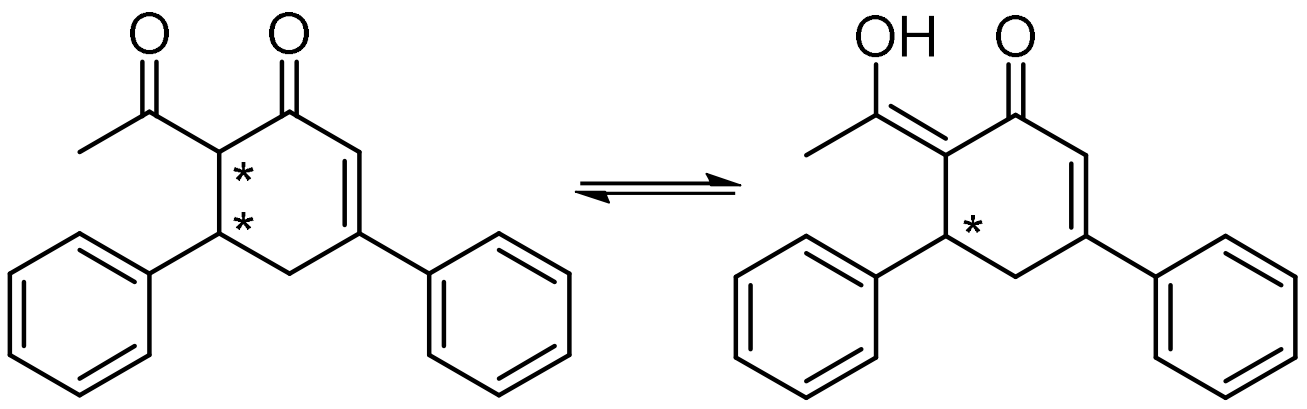

The compound **1a''**, prepared by **Procedure F**, was isolated after FLC (SiO_2, eluent: Hex/EtOAc = 8/1) and subsequent crystallization from Hex/EtOAc (554 mg, 62%) in the form of single-crystals suitable for X-ray diffraction analysis.**^{1}H NMR** (25 °C, $CDCl_3$, 500 MHz): *enol* form δ = 7.16 – 7.58 (m, 10H), 6.51 (d, $J(^{1}H-^{1}H)$ = 2.78 Hz, 1H), 4.15 (dd, $J(^{1}H-^{1}H)$ = 8.42 Hz, $J'(^{1}H-^{1}H)$ = 1.45 Hz, 1H), 3.32 (ddd, $J(^{1}H-^{1}H)$ = 17.2 Hz, $J'(^{1}H-^{1}H)$ = 8.42 Hz, $J''(^{1}H-^{1}H)$ = 2.78 Hz, 1H), 3.04 (dd, $J(^{1}H-^{1}H)$ = 17.2 Hz, $J'(^{1}H-^{1}H)$ = 1.45 Hz, 1H), 2.02 (s, 3H) ppm; *keto* form δ = 7.16 – 7.58 (m, 10H), 6.55 (d, $J(^{1}H-^{1}H)$ = 2.00 Hz, 1H), 3.97 (d, $J(^{1}H-^{1}H)$ = 12.0 Hz, 1H), 3.86 (ddd, $J(^{1}H-^{1}H)$ = 12.0 Hz, $J'(^{1}H-^{1}H)$ = 10.7 Hz, $J''(^{1}H-^{1}H)$ = 4.65 Hz, 1H), 3.16 (dd, $J(^{1}H-^{1}H)$ = 18.1 Hz, $J'(^{1}H-^{1}H)$ = 4.65 Hz, 1H), 3.00 (ddd, $J(^{1}H-^{1}H)$ = 18.1 Hz, $J'(^{1}H-^{1}H)$ = 10.7 Hz, $J''(^{1}H-^{1}H)$ = 4.65 Hz, 1H), 2.16 (s, 3H) ppm.

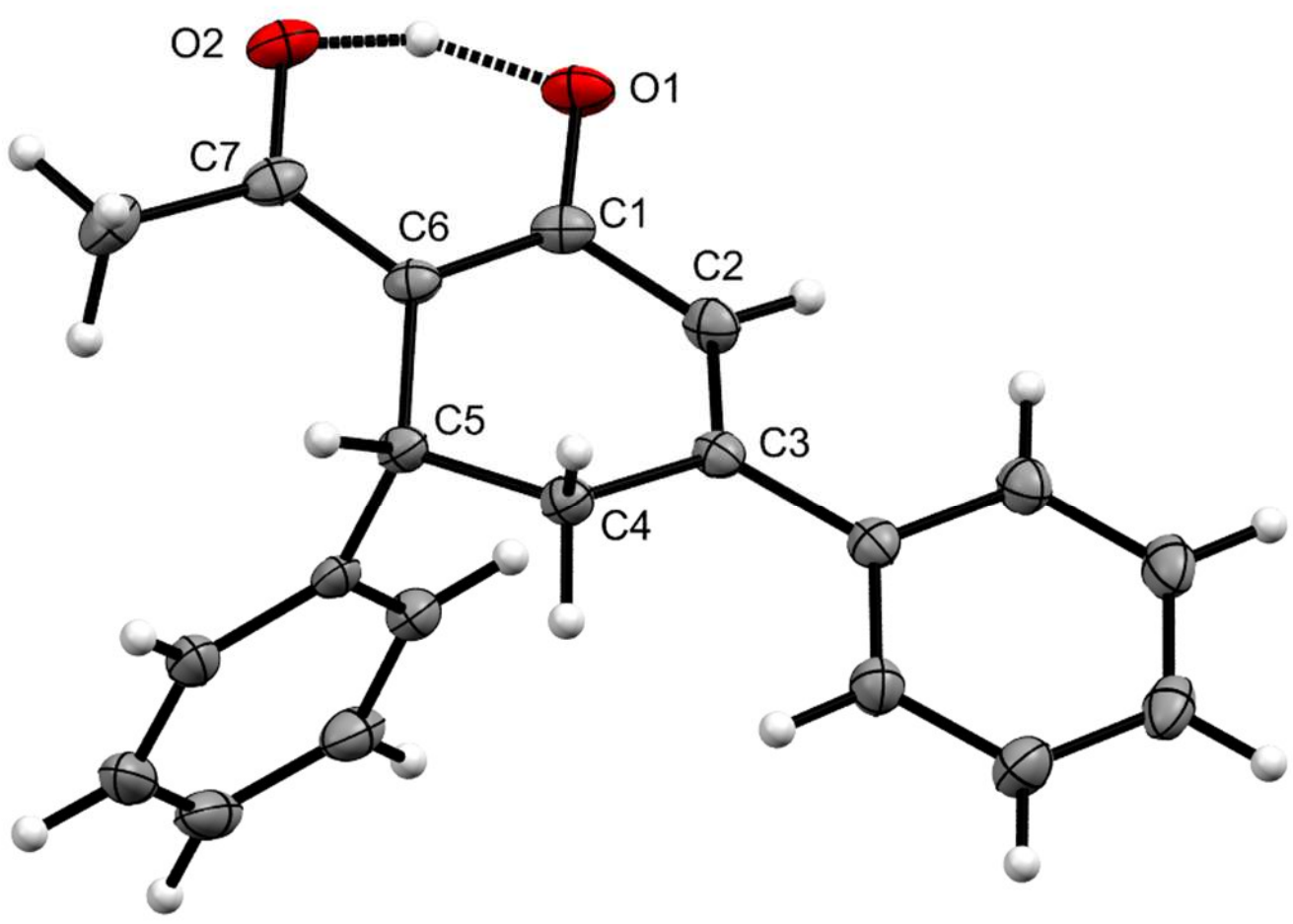

Supplementary Figure S74. The molecular structure of *enol*-1a''. ORTEP view, 30% probability level. Selected interatomic distances [Å]: C1-C2 1.454(3), C2-C3 1.5371(17), C3-C4 1.501(3), C4-C5 1.536(3), C5-C6 1.514(3), C6-C7 1.407(3), C1-C6 1.395(3), C7-O2 1.292(2), C1-O1 1.301(2).

Supplementary Table S13. Experimental details of *enol*-1a''.

Crystal data	
Chemical formula	$C_{20}H_{18}O_2$
M_r	290.34
Crystal system, space group	Monoclinic, $P2_1/c$
Temperature (K)	150
a, *b*, *c* (Å)	14.1683(17), 6.2114(8), 17.696(2)
β (°)	109.116(5)
V (Å^3)	1471.4(3)
Z	4
Radiation type	MoKα
μ (mm^{-1})	0.08
Crystal size (mm)	0.59 × 0.28 × 0.02
Data collection	
Diffractometer	Bruker D8 – Venture
Absorption correction	Multi-scan<br>*SADABS2016*/2 - Bruker AXS area detector scaling and absorption correction
T_{min}, T_{max}	0.631, 0.746
No. of measured, independent and observed [$I > 2\sigma(I)$] reflections	19638, 2737, 1847
R_{int}	0.096
$(\sin \theta/\lambda)_{max}$ (Å^{-1})	0.606
Refinement	
$R[F^2 > 2\sigma(F^2)]$, $wR(F^2)$, S	0.049, 0.117, 0.89
No. of reflections	2737
No. of parameters	203
No. of restraints	171
H-atom treatment	H atoms treated by a mixture of independent and constrained refinement
$\Delta\rho_{max}$, $\Delta\rho_{min}$ (e Å^{-3})	0.20, −0.20

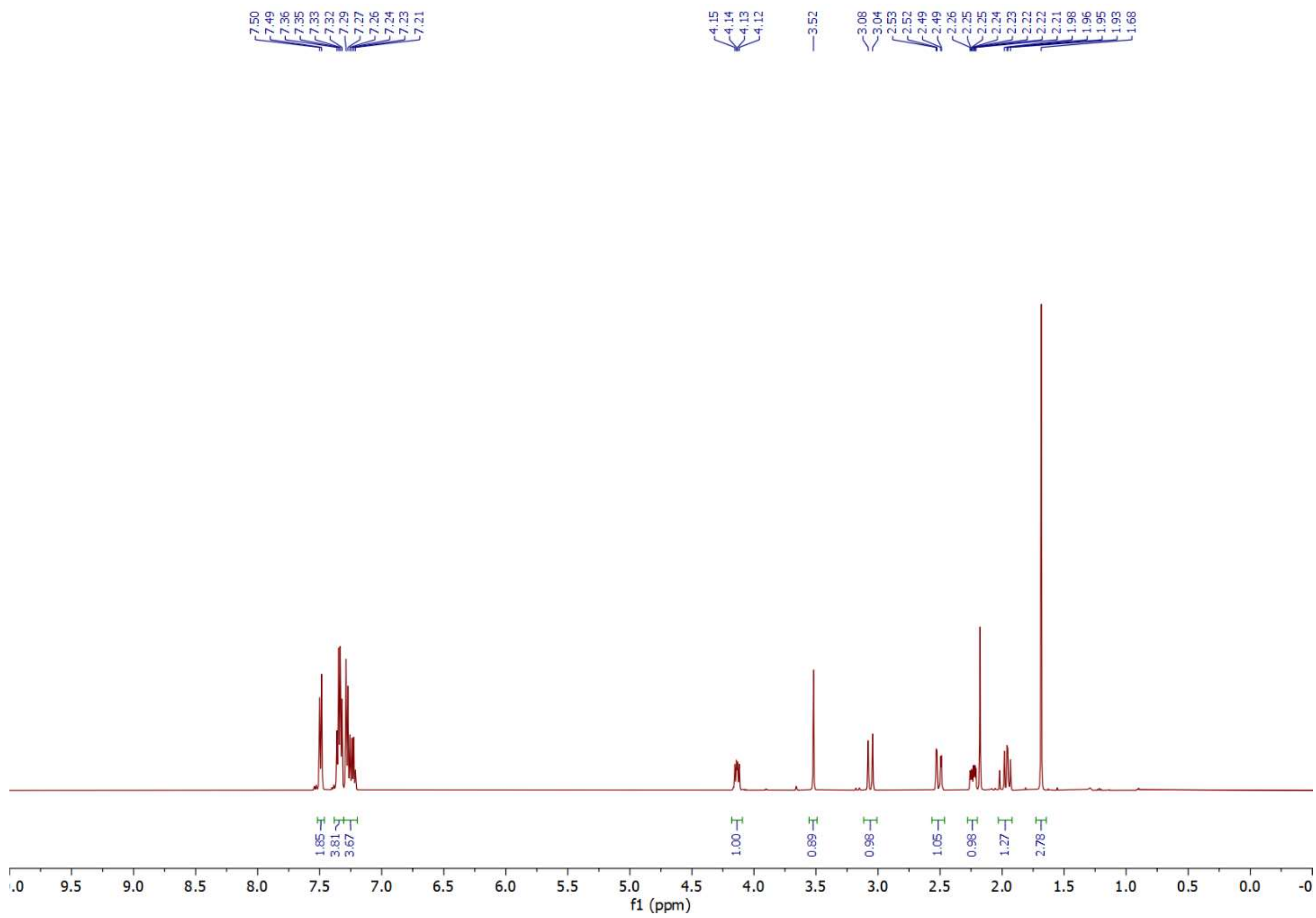

Supplementary Figure S75. ^{1}H (500.1 MHz) NMR spectrum of 1a'.

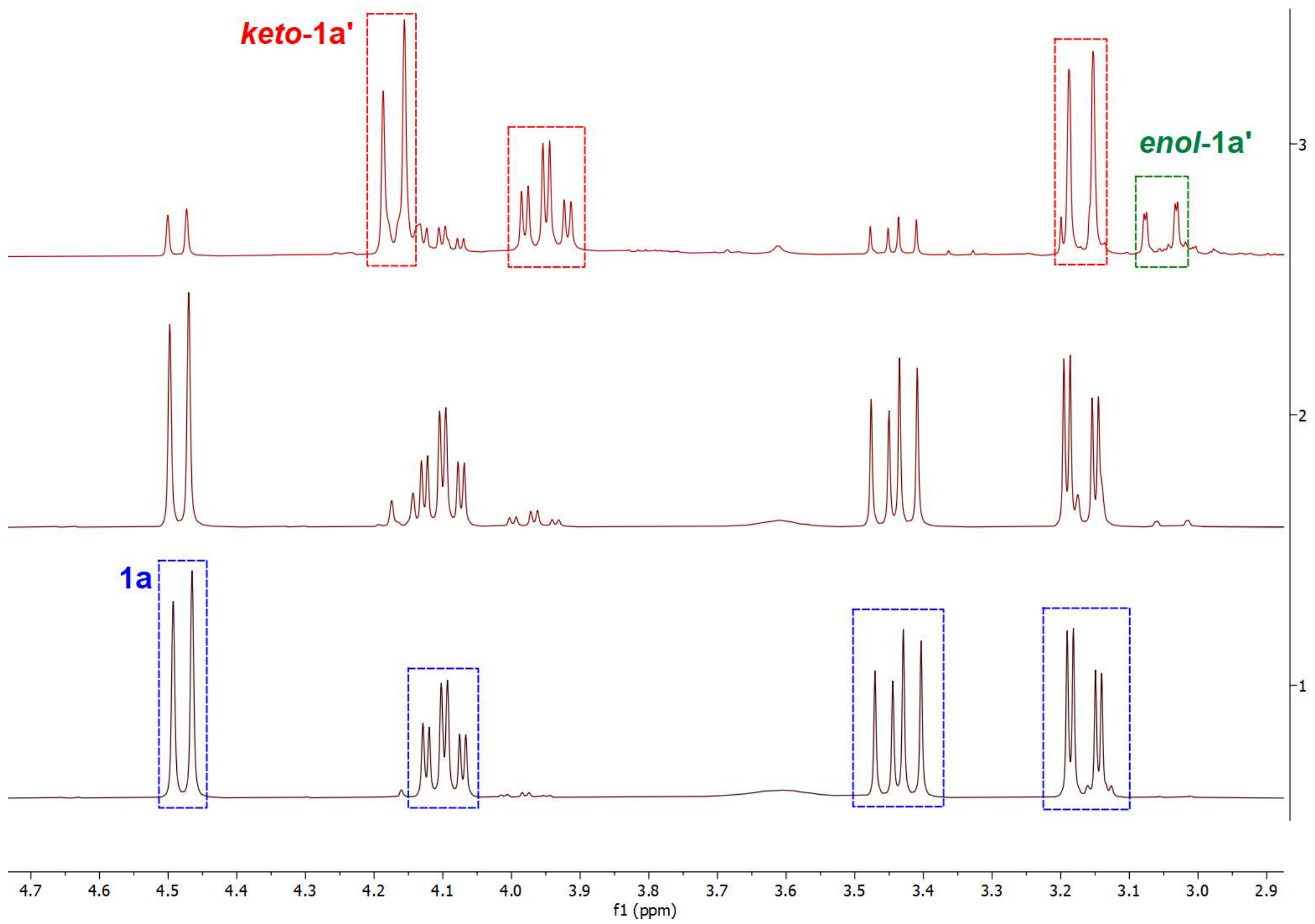

Supplementary Figure S76. ^{1}H (500.1 MHz) NMR analysis of the acetylacetone addition to *trans*-chalcone. Mapping of the formation of 1a and its transformation to *keto*-1a'/ *enol*-1a' (the reaction progress after 1 h, 3 h and 3 d is depicted from the bottom to the top).

Scope of Michael reaction (Procedure G)

According to the obtained optimization data and our practical experiences with the model reaction, we concluded that a subsequent reaction scope will be carried out using 3 mol% of *o*-**2**, in CD_3CN at room temperature with ^{1}H-NMR progress control after 1 h, 1 d and 7 d. Individual reactions were set as NMR experiments. The more detailed information are summarized in the Supplementary Tables S14–15 below.

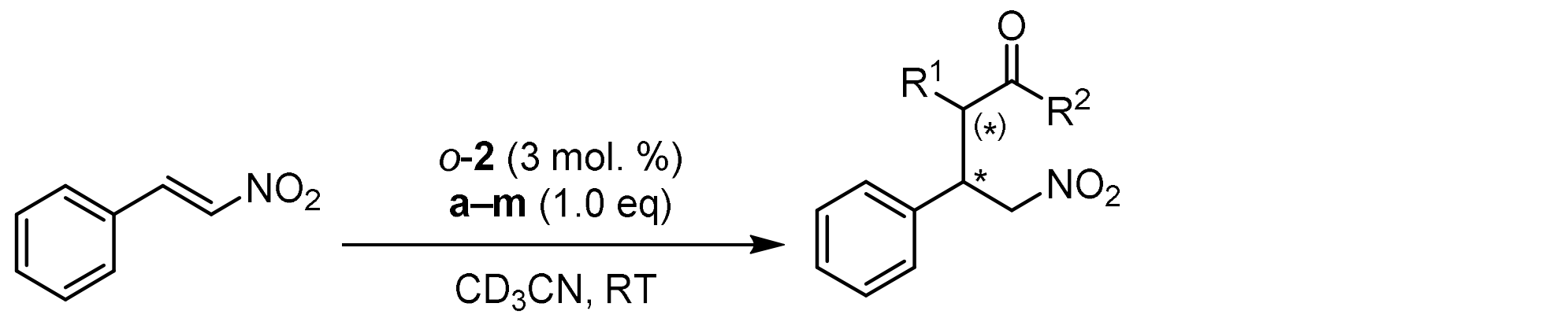

2a–2j

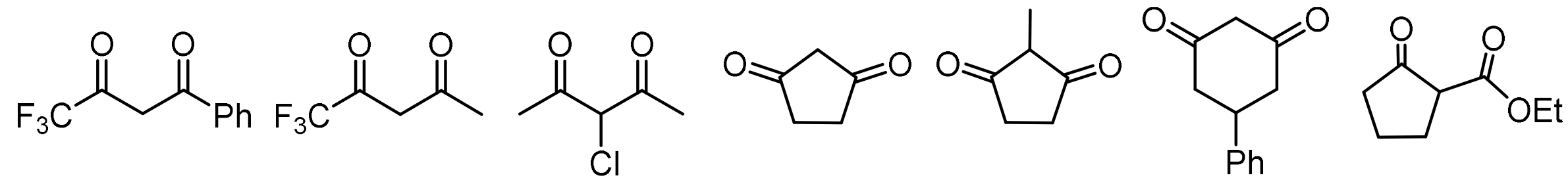

	a	b	c	d	e	f	g
Yield/Time	**26%/4d**	**57%/1d**	**63%/7d**	**99%/6d**	**24%/1h**	**64%/1d**	**99%/1h**
pK_A	6.35[S15]	6.69[S15]	6.77[S15]	4.4[S17]	4.7[S17]	4.92[S17]	12.0[S15]

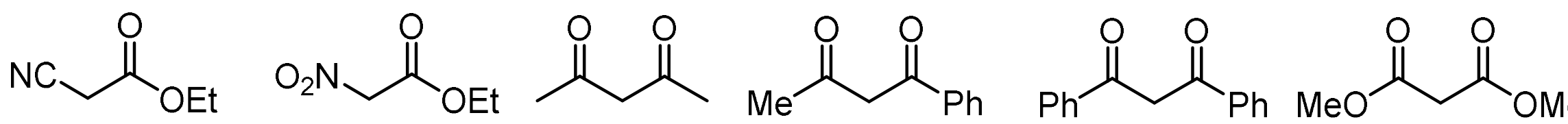

	h	i	j	k	l	m
Yield/Time	**57%/1h**	**99%/1d**	**99%/1h**	**0%**	**0%**	**0%**
pK_A	13.1[S18]	9.1[S18]	13.3[S18]	14.2[S18]	13.4[S18]	15.9[S18]

Supplementary Figure S77. Michael addition of various donors to β-nitrostyrene. The final conversions (in bold) of β-nitrostyrene were determined according to the catalyst o-2 as an internal standard using ^{1}H-NMR analysis. The published values of pK_A of donors g–m (in bold) were estimated in DMSO[S15] or predicted computationally[S12,S14].

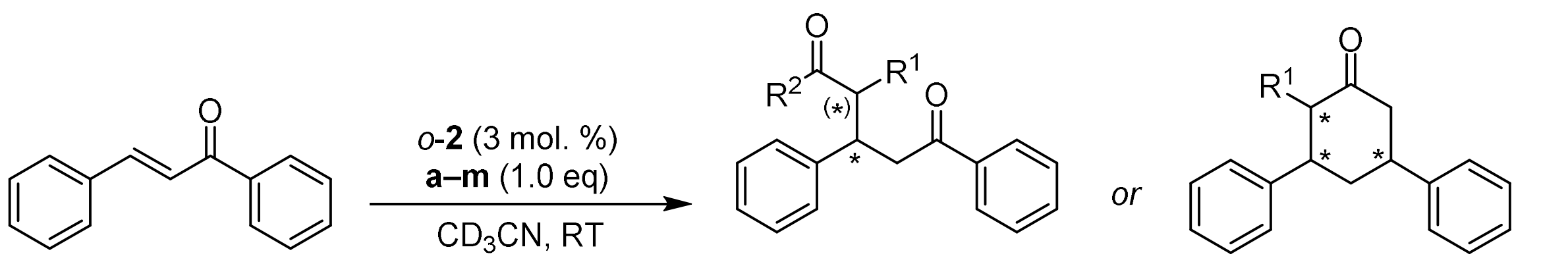

3g–3m **3i′–3l′**

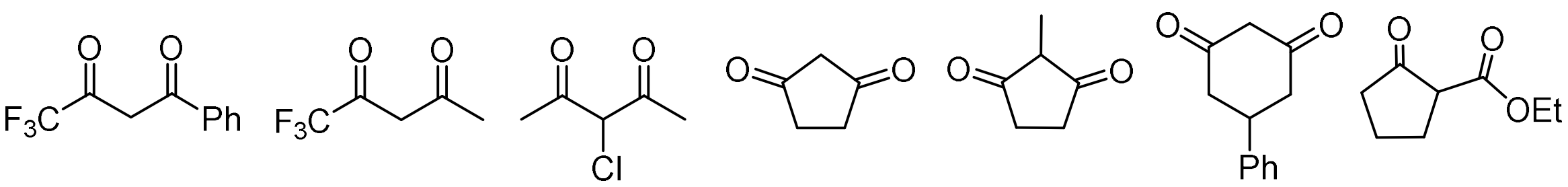

	a	b	c	d	e	f	g
Yield/Time	**0%**	**0%**	**0%**	**0%**	**0%**	**0%**	**82%/1h**
pK_A	6.35[S15]	6.69[S15]	6.77[S15]	4.4[S17]	4.7[S17]	4.92[S17]	12.0[S15]

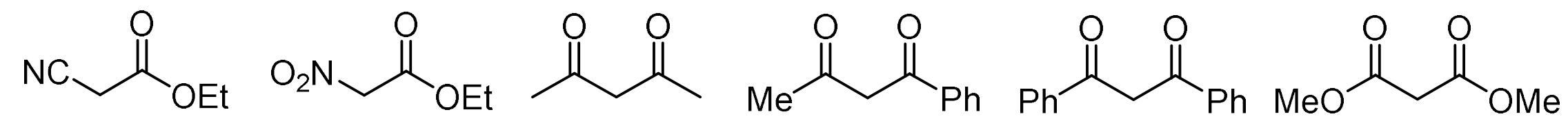

	h	i	j	k	l	m
Yield/Time	**99%/1h**	**99%/4d**	**99%/3d**	**90%/1h**	**60%/5d**	**99%/2h**
pK_A	13.1[S18]	9.1[S18]	13.3[S18]	14.2[S18]	13.4[S18]	15.9[S18]

Supplementary Figure S78. Michael addition of various donors to β-nitrostyrene. The final conversions (in bold) of β-nitrostyrene were determined according to the catalyst *o*-2 as an internal standard using ^{1}H-NMR analysis. The published values of pK_A of donors g–m (in bold) were estimated in DMSO[S15] or predicted computationally[S12,S14].

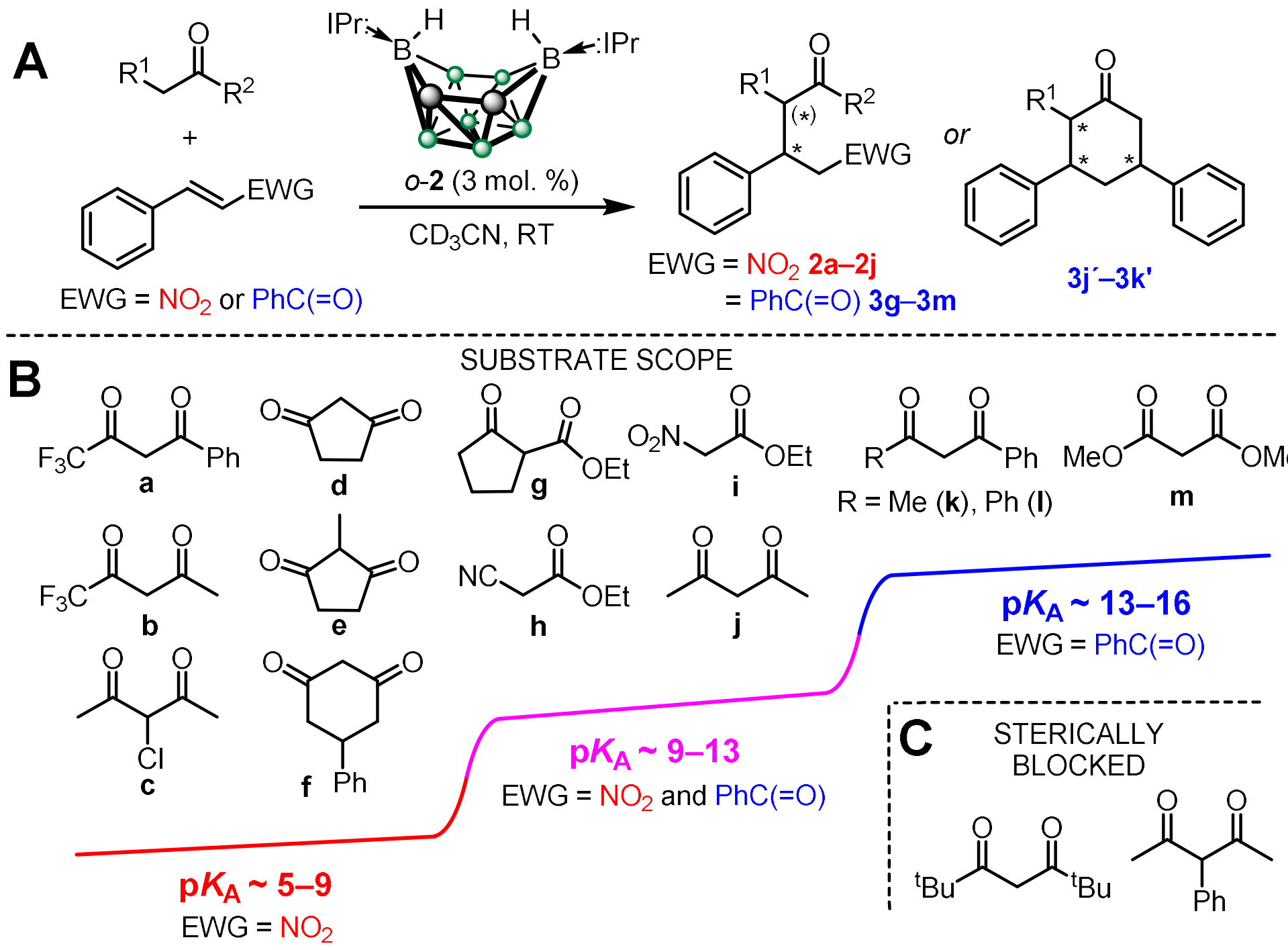

Supplementary Figure S79. The studied Michael addition on β-nitrostyrene and *trans*-chalcone (A) using *o*-2 as a catalyst. The relationship between the reaction outcome and the acidity of the Michael donor (B) is demonstrated on the pK_A of the used acids a–m. Despite a reasonable acidity, some reagents were not suitable for steric reasons (C).

Supplementary Table S14. Michael addition reaction of β-nitrostyrene - summary.

Donor	Result[a]	Structure the product(s)
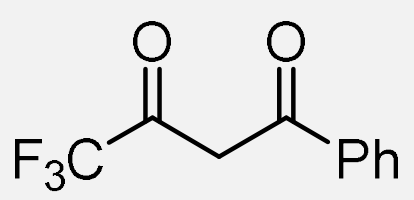	**2a** formed with 26% conversion of β-nitrostyrene after 4 d.[S12]	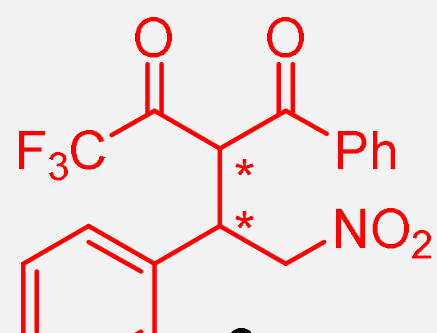 **2a**
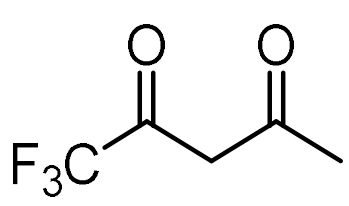	**2b** formed with 57% conversion of β-nitrostyrene after 24 h. Observed decomposition of **2b** after 7 d.[b,c]	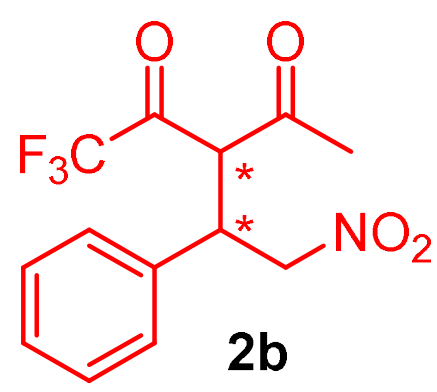 **2b**
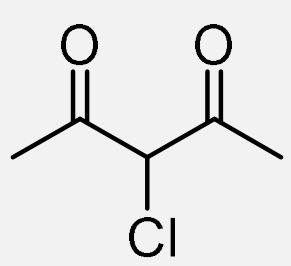	**2c** formed with 63% conversion of β-nitrostyrene after 7 d.[b,d]	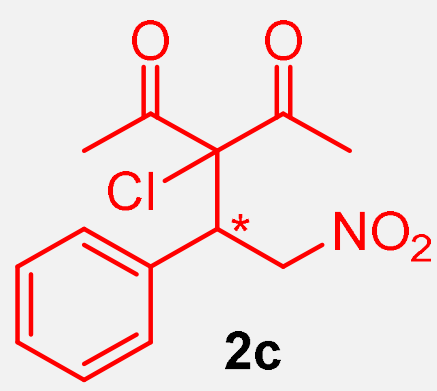 **2c**

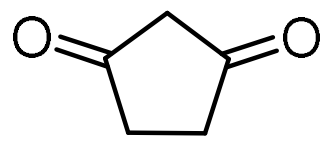	**2d** formed with 99% conversion of β-nitrostyrene after 6 d.[S13,d]	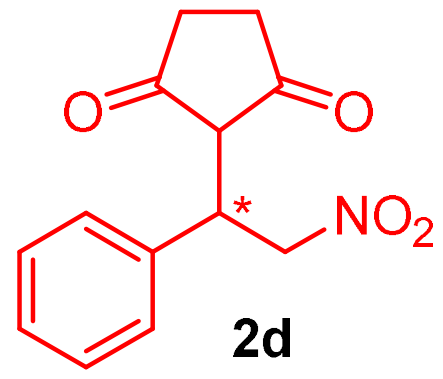 **2d**
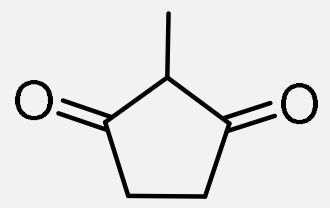	**2e** formed with 24% conversion of β-nitrostyrene after 1 h. Observed full transformation of **2e** to **2e'** with 75% conversion of β-nitrostyrene after 6 d.[S14,S19]	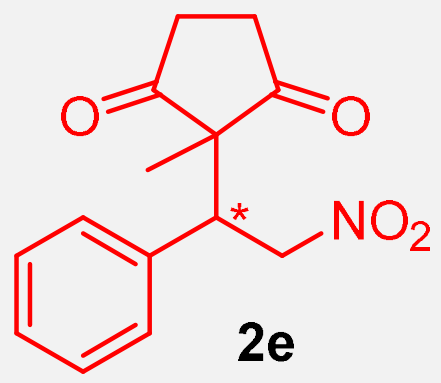 **2e** 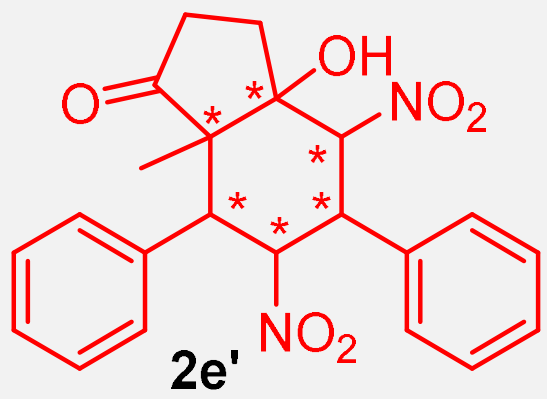 **2e'**
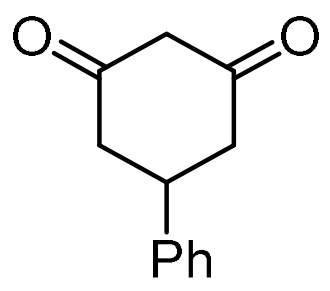	**2f** formed with 63% conversion of β-nitrostyrene after 1 d. Observed decomposition of **2f** after 4 d.[b,d]	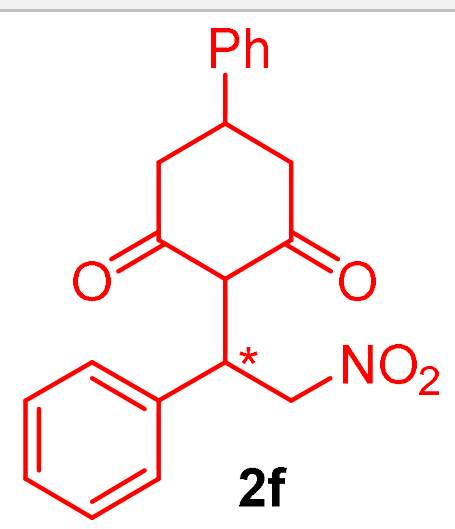 **2f**
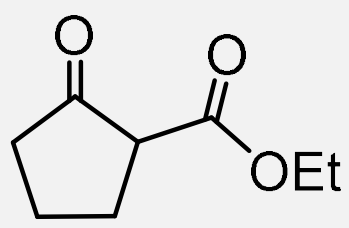	**2g** formed with 99% conversion after 1 h.	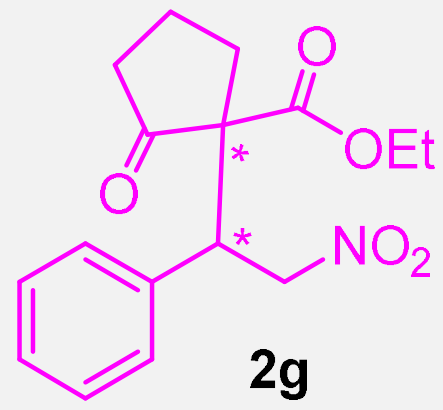 **2g**
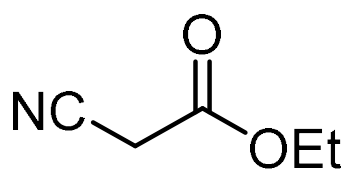	**2h** formed after 4 h. Conversion of β-nitrostyrene cannot be determined because of its partial polymerisation. Calculated conversion of ethyl 2-cyanoacetate was 56 %.[S12,d]	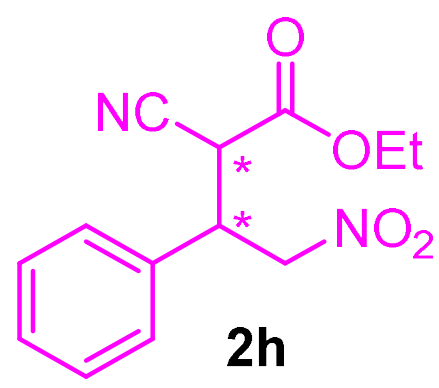 **2h**
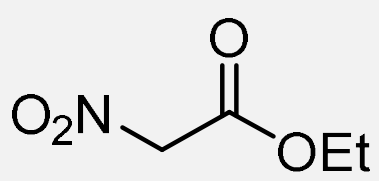	**2i** formed with 99% conversion after 24 h. Observed mild decomposition of **2i** after 6 d.[S10,d]	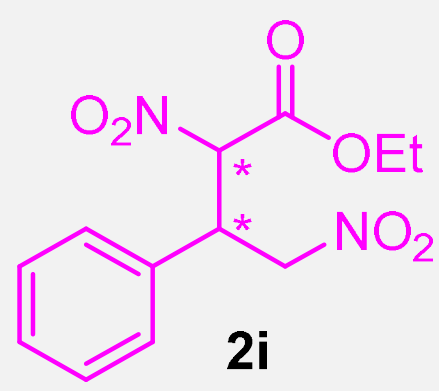 **2i**
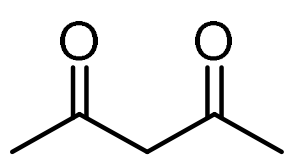	**2j** formed with 99% conversion after 1 h.[S11]	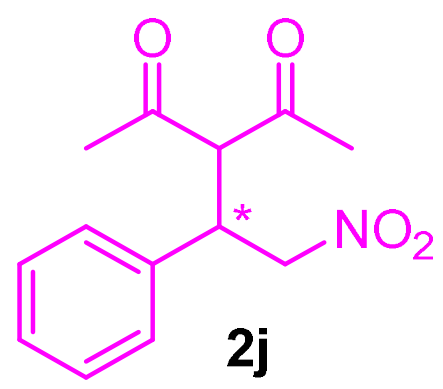 **2j**
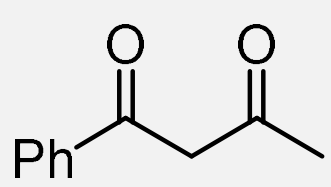	β-nitrostyrene polymerisation	

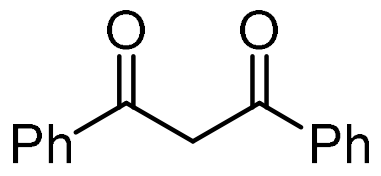	β-nitrostyrene polymerisation
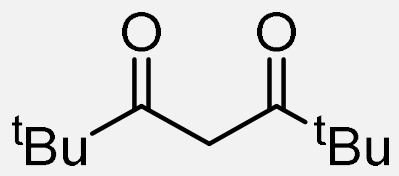	β-nitrostyrene polymerisation
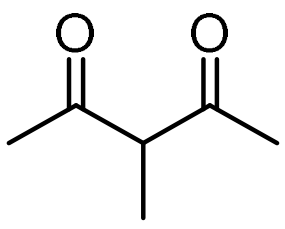	β-nitrostyrene polymerisation
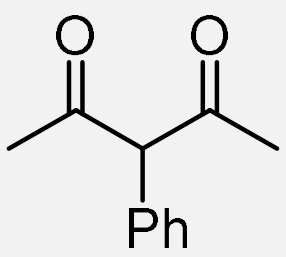	β-nitrostyrene polymerisation
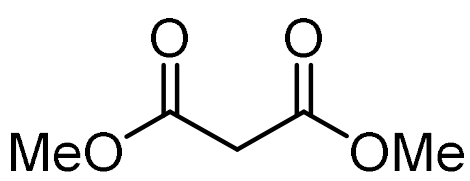	β-nitrostyrene polymerisation

Note: [a]The final conversion of β-nitrostyrene was determined according to the catalyst *o*-**2** as an internal standard using ^{1}H-NMR analysis. [b]The compound is not described in literature and its identification is based on ^{1}H-NMR analysis of the reaction mixture. [c]5 mol.% of the catalyst *o*-**2** was used in the reaction. [d]Reaction was carried out in DMSO-d_6.

Supplementary Table S15. Michael addition reaction of *trans*-chalcone - summary of the scope.

Donor	Result[a]	Structure of the product(s)
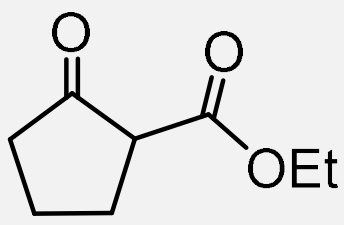	**3g** formed with 82% conversion after 1 h.	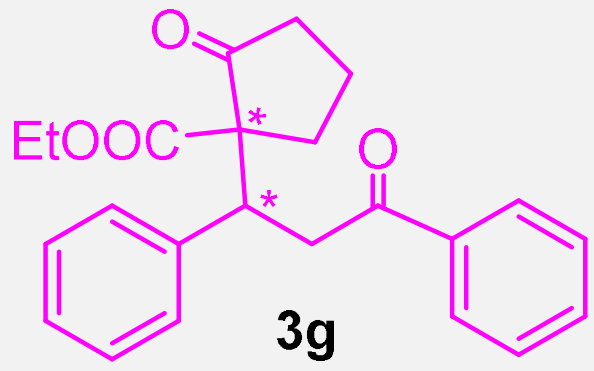 **3g**
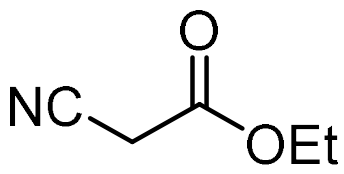	**3h** formed with 99% conversion of *trans*-chalcone after 24 h. Observed decomposition of **3h** after 5 d.[S20]	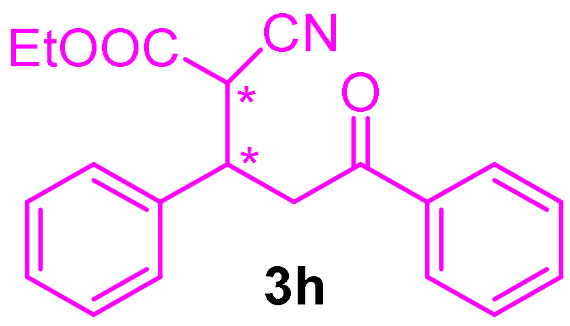 **3h**
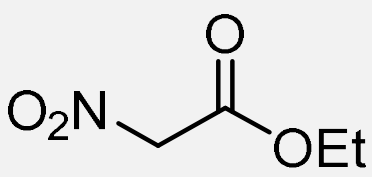	**3i** formed with 83% conversion of *trans*-chalcone after 4 d.[S19]	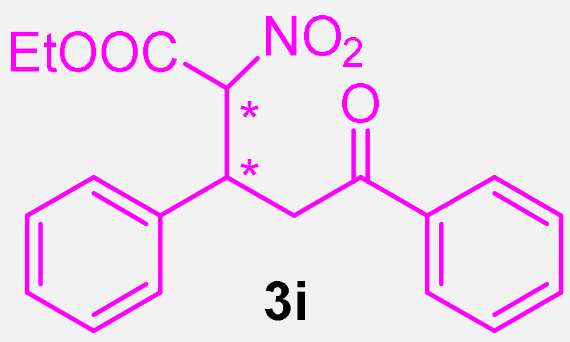 **3i**
	**3j = 1a** formed dominantly with 70% conversion of *trans*-chalcone after 1 h. **3j = 1a** transformed to **1a'** with 99% conversion of trans-chalcone after 3 d. **1a''** formed subsequently at increased temperature (80 °C) or after addition of I_2 at RT.[S7]	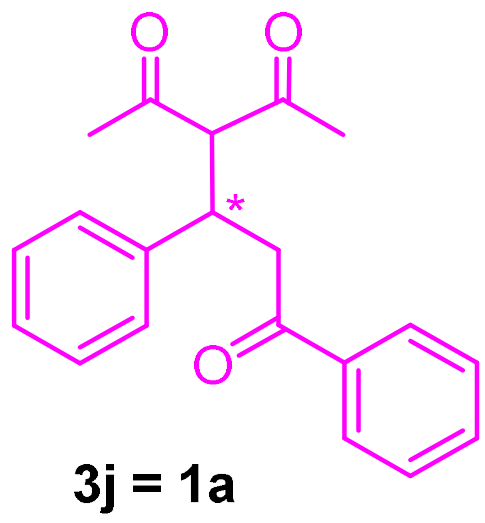 **3j = 1a** 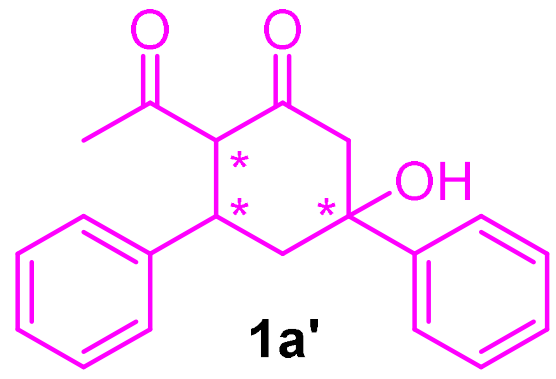 **1a'** *keto / enol* 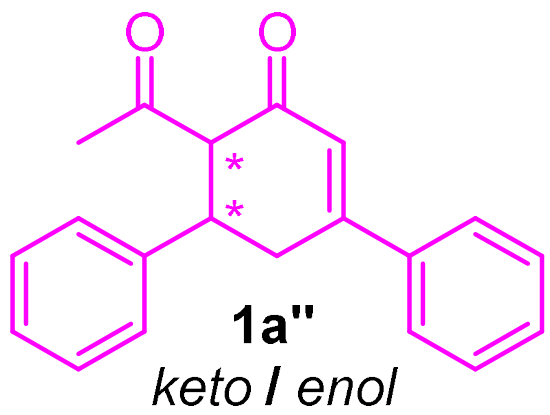 **1a''** *keto / enol*
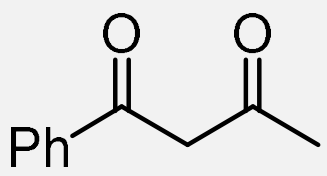	**3k** formed after 1 h. Observed incipient transformation of **3k** to **3k'** after 1 d. **3k'** formed as a dominant product with 90% conversion of *trans*-chalcone after 7 d.[S7]	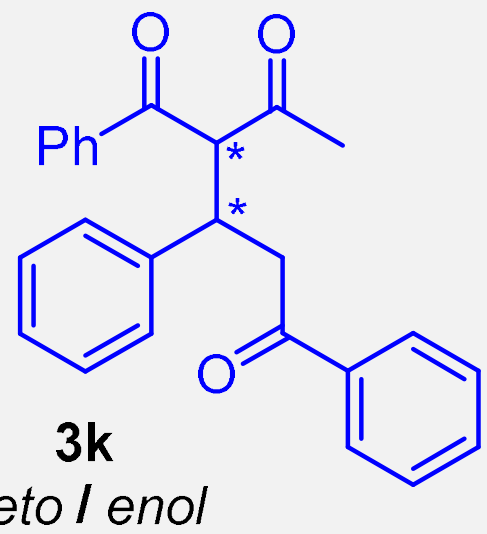 **3k** *keto / enol* 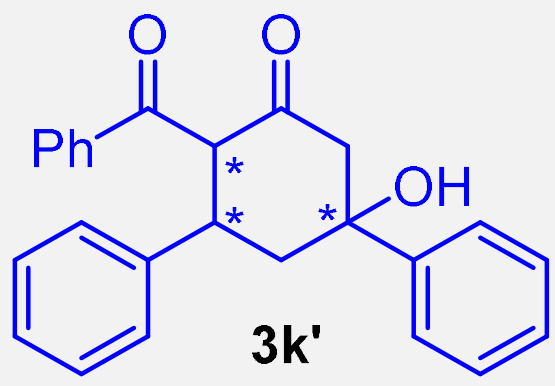 **3k'**
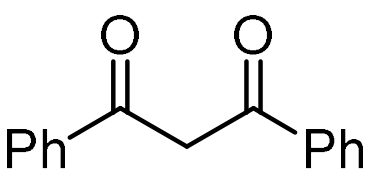	**3l** formed with 60% conversion of *trans*-chalcone after 5 d.[S20]	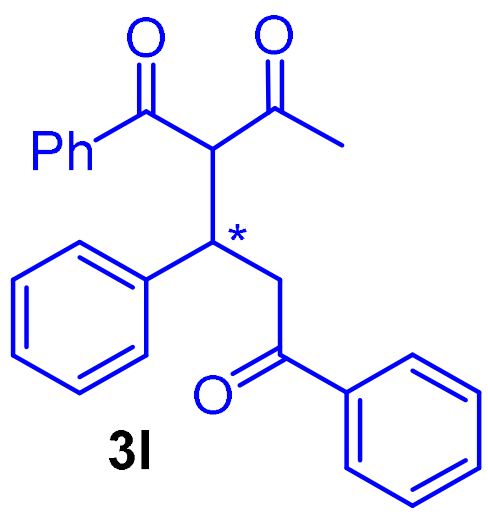 **3l**

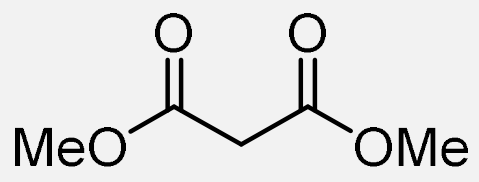	**3m** formed with 99% conversion of *trans*-chalcone after 2 h.[S20]	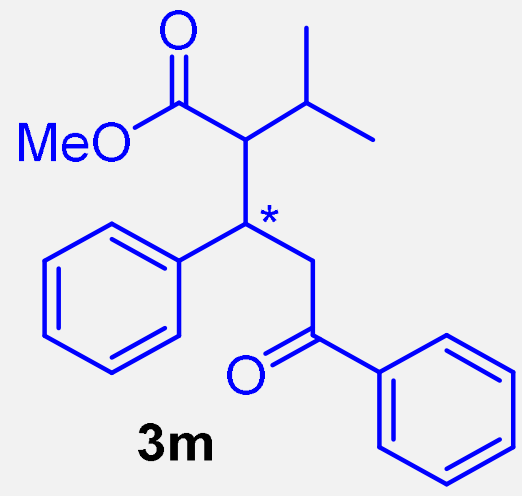 **3m**
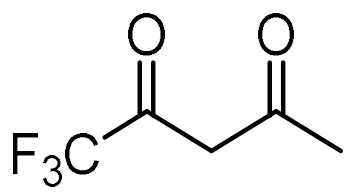	No reaction	
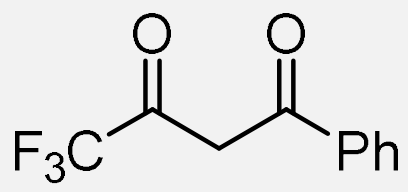	No reaction	
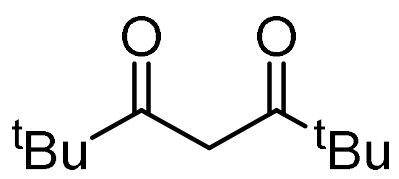	No reaction	
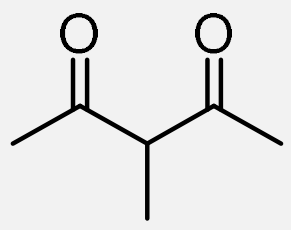	No reaction	
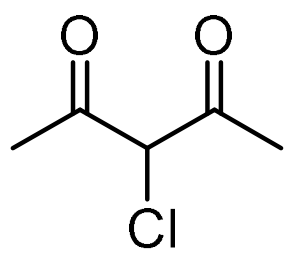	No reaction	
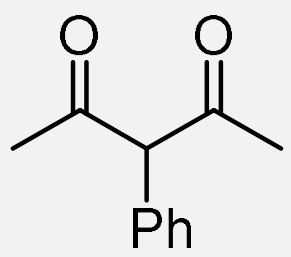	No reaction	
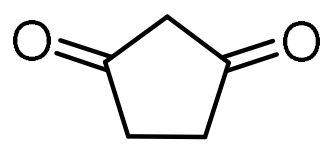	No reaction	

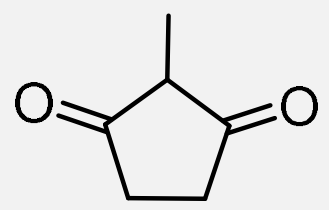	No reaction
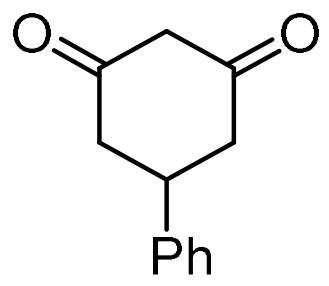	No reaction

Note: [a]The final conversion of *trans*-chalcone was determined according to the catalyst *o*-**2** as an internal standard using ^{1}H-NMR analysis.

Catalytic conjugated addition reaction of pyrazolones with nitrostyrene derivatives (Procedure H)

To the vial equipped with magnetic stir bar was added catalyst *o*-**2** (0.9 mg, 0.005 mmol, 0.05 equiv.), followed by addition of olefin derivative **5a**–**f** (0.2 mmol, 2 equiv.), DCM (1 mL) and pyrazolone derivative **4d**–**e** (0.10 mmol, 1 equiv.). Reaction mixture was then stirred at room temperature. After full reaction was reached, reaction mixture was directly loaded on the plug of silica and corresponding product **6a**–**h** was isolated using the mixture hexane:ethyl acetate.

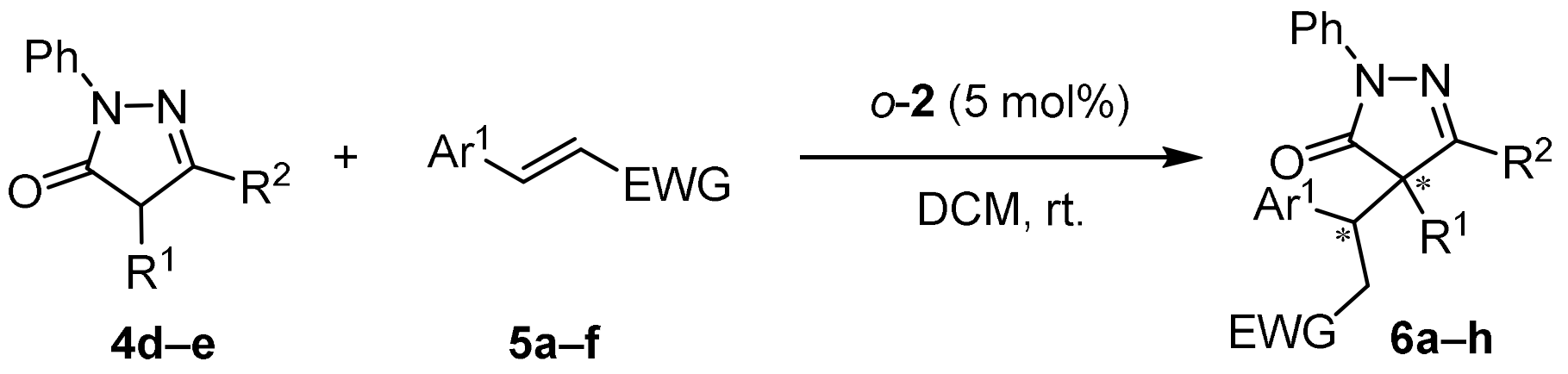

Supplementary Figure S80. Addition of pyrazolones 4d–e to nitrostyrene derivatives 5a–f.

4-Benzyl-5-methyl-4-(2-nitr*o*-1-phenylethyl)-2-phenyl-2,4-dihydr*o*-3*H*-pyrazol-3-one (6a)[S19]

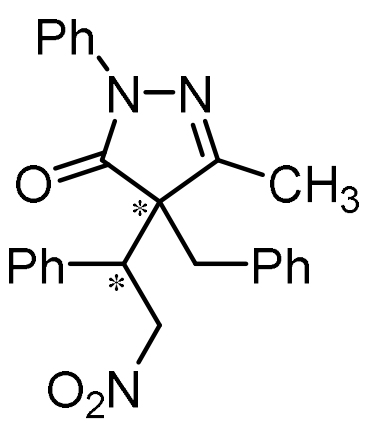

Compound was prepared and isolated by **Procedure H**. Colorless oil. Yield 95% (39 mg), *Dr*. 8:1. **^{1}H NMR of major diastereoisomer**: (25 °C, $CDCl_3$, 600 MHz): δ = 7.42–7.40 (m, 2H), 7.36–7.28 (m, 3H), 7.24–7.17 (m, 10H), 5.37–5.30 (m, 2H), 3.98 (dd, J(^{1}H-^{1}H) = 10.1 Hz, J'(^{1}H-^{1}H) = 4.6 Hz, 1H), 3.52 (d, J(^{1}H-^{1}H) = 13.1 Hz, 1H), 3.07 (d, J(^{1}H-^{1}H) = 13.2 Hz, 1H), 2.03 (s, 3H) ppm; **^{13}C NMR of major diastereoisomer**: (25 °C, $CDCl_3$, 150.9 MHz): δ = 173.8, 160.0, 137.0, 135.0, 133.7, 129.2 (2C), 129.1 (2C), 128.9 (2C), 128.7 (2C), 128.6, 127.9, 127.8, 127.7, 126.0, 120.1 (2C), 76.2, 62.5, 48.3, 40.0, 15.1 ppm. **^{1}H NMR of minor diastereoisomer**: (25 °C, DMS*O*-d_6, 600 MHz): δ = 7.57–7.53 (m, 2H), 7.48 (dd, J(^{1}H-^{1}H) = 8.7 Hz, J'(^{1}H-^{1}H) = 1.2 Hz, 2H), 7.42 (dd, J(^{1}H-^{1}H) = 8.6 Hz, J'(^{1}H-^{1}H) = 1.3 Hz, 1H), 7.34–7.29 (m, 4H), 7.24–7.16 (m, 1H), 7.14–7.10 (m, 3H), 7.02 (dd, J(^{1}H-^{1}H) = 6.7, 2.8 Hz, 2H), 4.92 (dd, J(^{1}H-^{1}H) = 12.9 Hz, J'(^{1}H-^{1}H)= 11.0 Hz, 1H), 4.64 (dd, J(^{1}H-^{1}H) = 12.9 Hz, J'(^{1}H-^{1}H) = 4.1 Hz, 1H), 4.01 (dd, J(^{1}H-^{1}H) = 11.7 Hz, J'(^{1}H-^{1}H) = 4.6 Hz, 1H), 3.01 (d, J(^{1}H-^{1}H) = 13.5 Hz, 1H), 2.86 (d, J(^{1}H-^{1}H) = 13.6 Hz, 1H), 2.27 (s, 3H) ppm. **^{13}C NMR of minor diastereoisomer** (25 °C, $CDCl_3$, 100.61 MHz): δ = 172.9, 160.0, 137.0, 133.7, 129.9, 129.1 (6C), 129.0 (2C), 128.6 (2C), 127.9, 127.8, 125.9, 119.9 (2C), 76.2, 61.6, 47.6, 39.8, 15.3 ppm. LRMS (ESI) m/z: calcd for $C_{25}H_{23}$ N_3 O_3Na $[M+Na]^+$ 436.2; found 436.2.

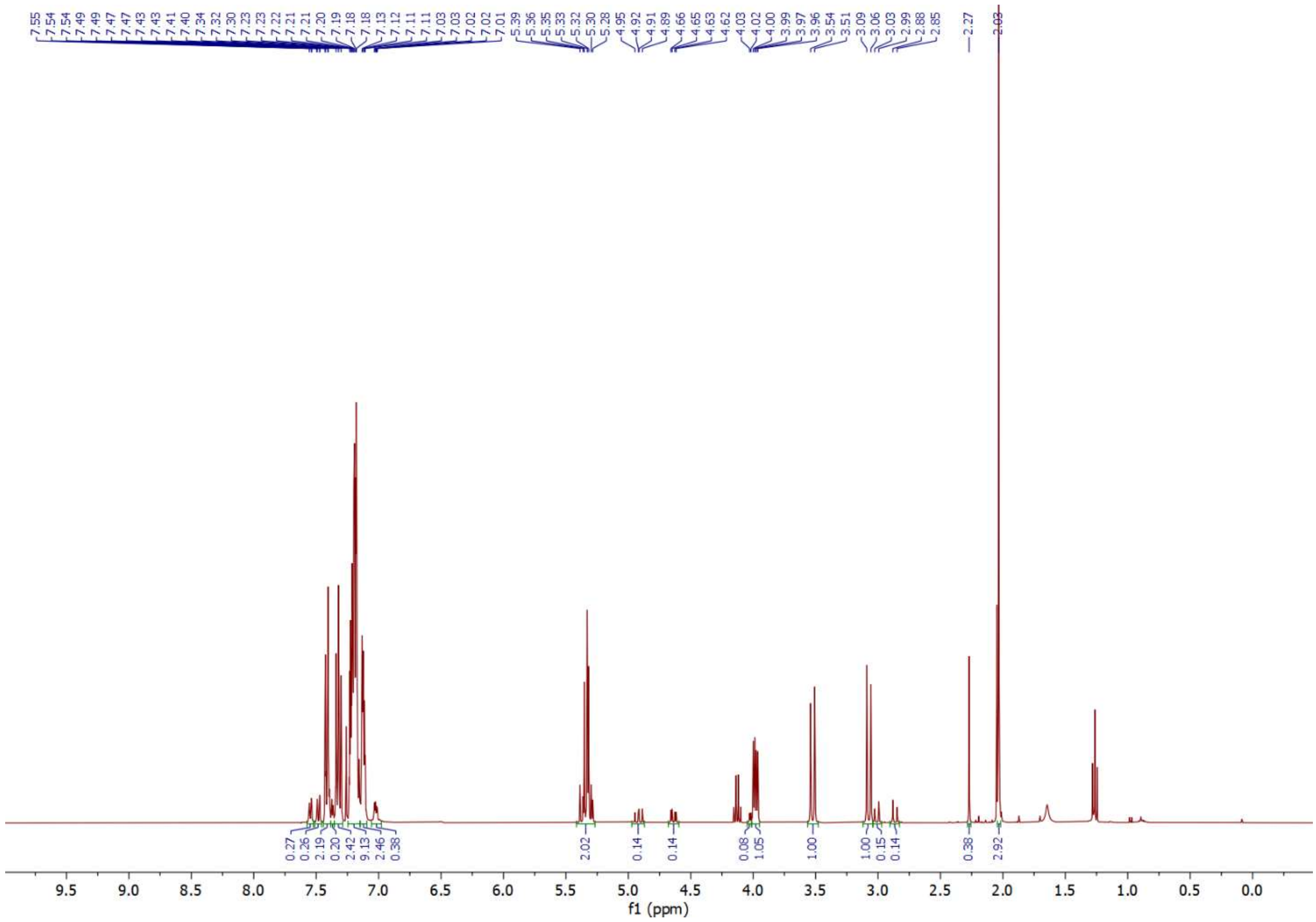

Supplementary Figure S81. The ^{1}H (600.1 MHz) NMR spectrum of 6a.

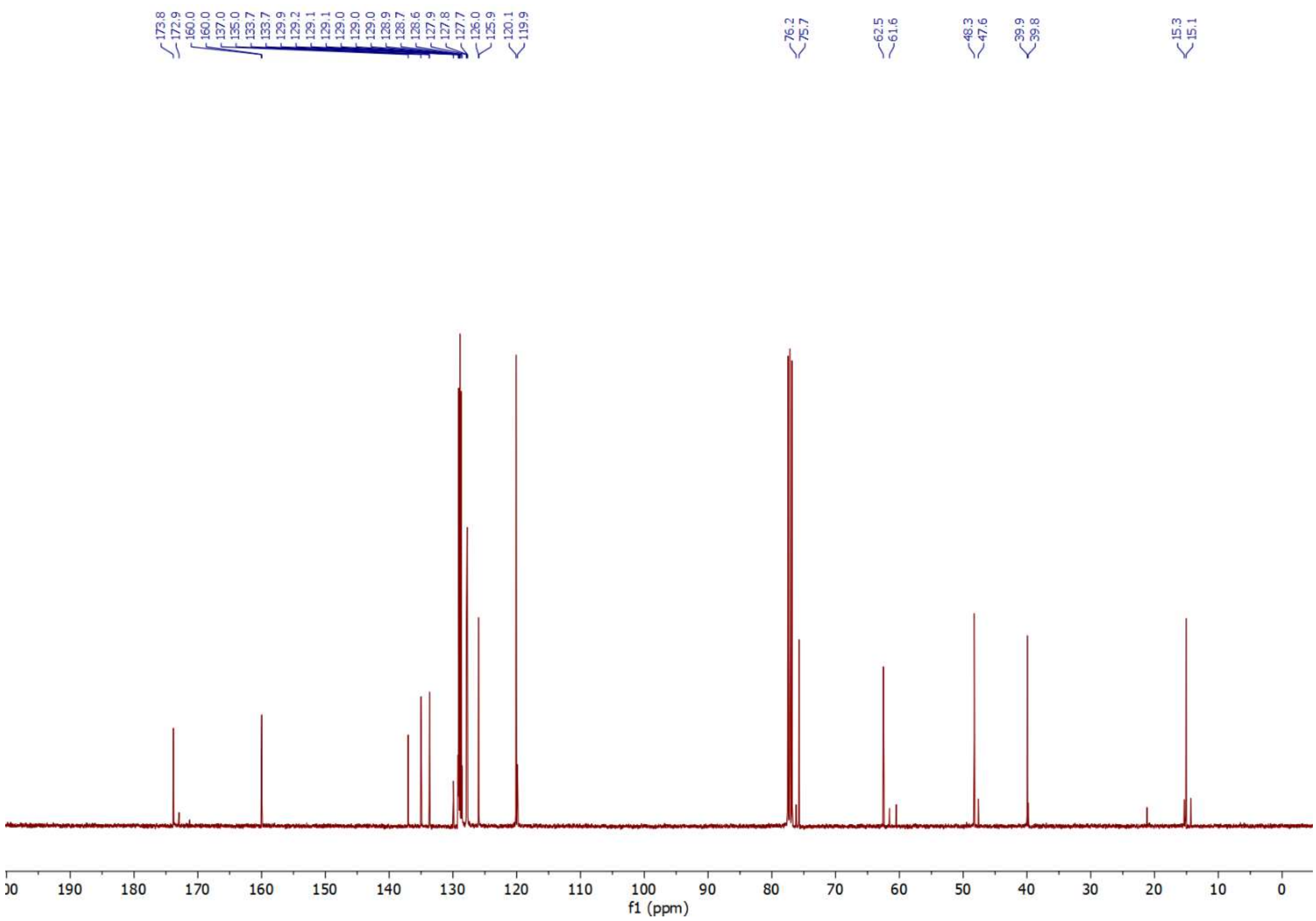

Supplementary Figure S82. The $^{13}C\{^{1}H\}$ (150.9 MHz) NMR spectrum of 6a.

4-Benzyl-5-methyl-4-(1-(4-methoxyphenyl)-2-nitroethyl)-2-phenyl-2,4-dihydr*o*-3*H*-pyrazol-3-one (6b)

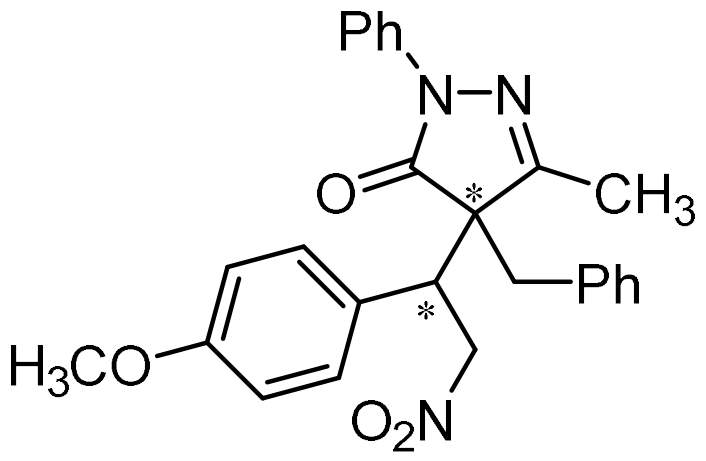

Compound was prepared and isolated by **Procedure H**. Colorless oil. Yield 82% (36 mg), *Dr*. 7:1. **^{1}H NMR of major diastereoisomer**: (25 °C, $CDCl_3$, 400 MHz): δ = 7.46–7.40 (m, 2H), 7.35–7.29 (m, 2H), 7.22–7.16 (m, 4H), 7.13–7.07 (m, 4H), 6.76–6.69 (m, 2H), 5.32 (dd, *J*(^{1}H-^{1}H) = 13.3 Hz, *J*´(^{1}H-^{1}H) = 10.4 Hz, 1H), 5.25 (dd, *J*(^{1}H-^{1}H) = 13.3 Hz, *J*´(^{1}H-^{1}H) = 4.4 Hz, 1H), 3.92 (dd, *J*(^{1}H-^{1}H) = 10.5 Hz, *J*´(^{1}H-^{1}H) = 4.4 Hz, 1H), 3.70 (s, 3H), 3.50 (d, *J*(^{1}H-^{1}H) = 13.2 Hz, 1H), 3.05 (d, *J*(^{1}H-^{1}H) = 13.2 Hz, 1H), 2.03 (s, 3H) ppm. **^{13}C NMR of major diastereoisomer**: (25 °C, $CDCl_3$, 150.9 MHz): δ = 173.9, 160.1, 159.9, 137.1, 133.8, 129.1 (2C), 128.9 (4C), 128.7 (2), 127.9, 126.8, 126.0, 120.1 (2C), 114.5 (2C), 75.9, 62.7, 55.3, 47.7, 40.0, 15.1 ppm. **^{1}H NMR of minor diastereoisomer**: (25 °C, $CDCl_3$, 400 MHz): δ = 7.53–7.41 (m, 3H), 7.36–7.29 (m, 2H), 7.21–7.15 (m, 3H), 7.14–7.07 (m, 4H), 7.04–7.00 (m, 1H), 6.92–6.90 (m, 1H), 4.85 (dd, *J*(^{1}H-^{1}H) = 12.7 Hz, 11.2 Hz, 1H), 4.58 (dd, *J*(^{1}H-^{1}H) = 12.7 Hz, 4.1 Hz, 1H), 3.98–3.88 (m, 1H), 3.80 (s, 3H), 3.00 (d, *J*(^{1}H-^{1}H) = 13.4 Hz, 1H), 2.83 (d, *J*(^{1}H-^{1}H) = 13.5 Hz, 1H), 2.27 (s, 3H) ppm. **^{13}C NMR of minor diastereoisomer** (25 °C, $CDCl_3$, 100.61 MHz): δ = 173.0, 160.2, 160.0, 137.1, 133.8, 129.1 (2C), 128.9 (4C), 128.6 (2C), 127.7, 125.9, 125.4, 119.8 (2C), 114.3 (2C), 75.5, 61.7, 55.4, 47.0, 39.9, 15.3 ppm. LRMS (ESI) m/z: calcd for $C_{26}H_{25}$ N_3 O_4Na $[M+Na]^+$ 466.2; found 466.2.

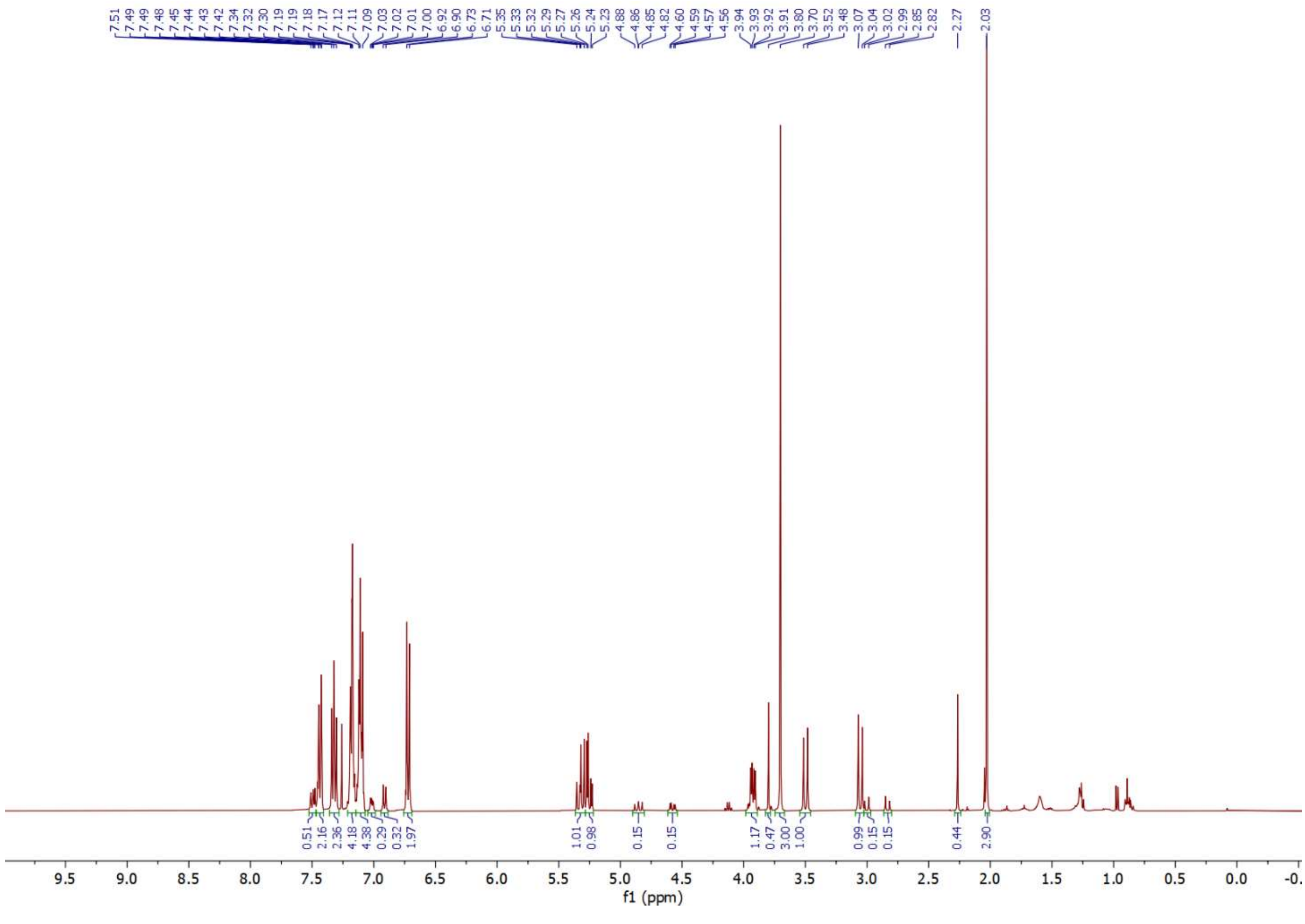

Supplementary Figure S83. The ^{1}H (400.1 MHz) NMR spectrum of 6b.

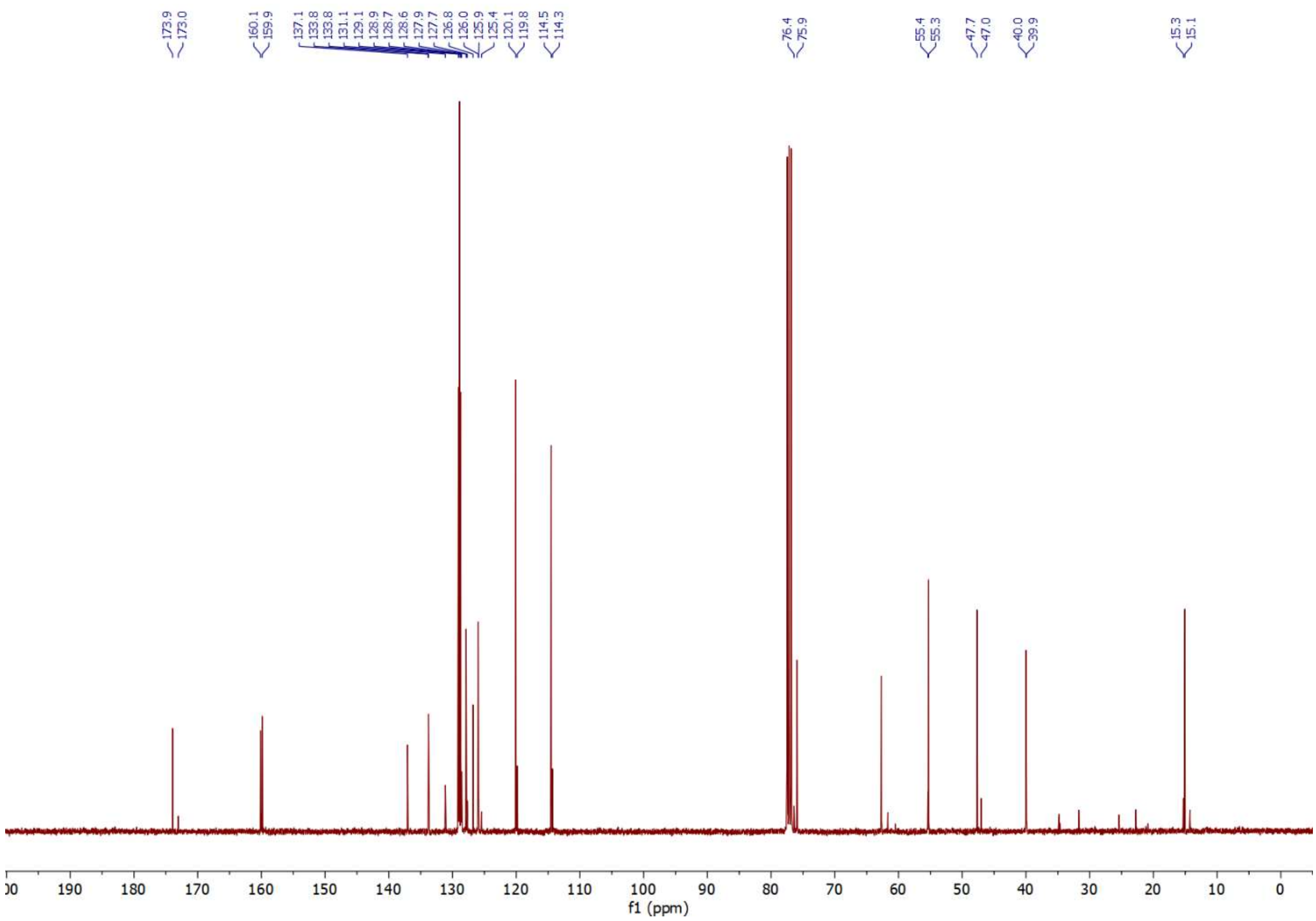

Supplementary Figure S84. The $^{13}C\{^{1}H\}$ (100.6 MHz) NMR spectrum of 6b.

4-Benzyl-5-methyl-4-(1-(4-bromophenyl)-2-nitroethyl)-2-phenyl-2,4-dihydr*o*-3*H*-pyrazol-3-one (6c)

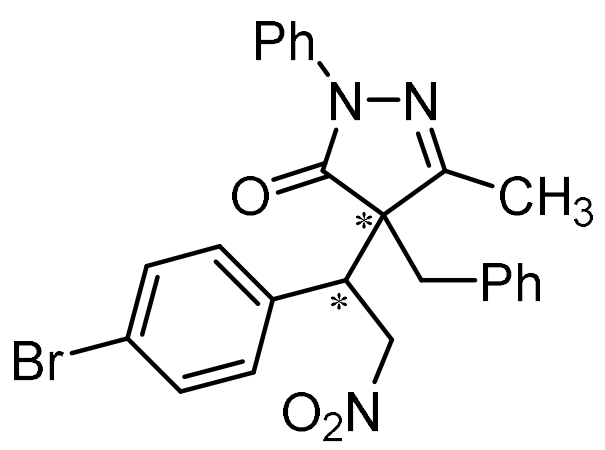

Compound was prepared and isolated by **Procedure H**. Colorless oil. Yield 92% (45 mg), *Dr*. 6:1. **^{1}H NMR of major diastereoisomer**: (25 °C, $CDCl_3$, 400 MHz): δ = 7.44–7.40 (m, 2H), 7.38–7.33 (m, 4H), 7.22–7.15 (m, 4H), 7.14–7.04 (m, 4H), 5.33 (dd, $J(^1H-^1H)$ = 13.6 Hz, $J'(^1H-^1H)$ = 10.3 Hz, 1H), 5.27 (dd, $J(^1H-^1H)$ = 13.6 Hz, $J'(^1H-^1H)$ = 4.5 Hz, 1H), 3.95 (dd, $J(^1H-^1H)$ = 10.3 Hz, $J'(^1H-^1H)$ = 4.6 Hz, 1H), 3.51 (d, $J(^1H-^1H)$ = 13.1 Hz, 1H), 3.05 (d, $J(^1H-^1H)$ = 13.2 Hz, 1H), 2.03 (s, 3H) ppm. **^{13}C NMR of major diastereoisomer**: (25 °C, $CDCl_3$, 150.9 MHz): δ = 173.6, 159.7, 136.9, 134.1, 133.4 (2C), 132.4, 129.4 (2C), 129.1 (2C), 129.0 (2C), 128.8 (2C), 128.0, 126.2, 123.3, 120.0 (2C), 75.4, 62.3, 47.6, 39.9, 15.1 ppm.

^{1}H NMR of minor diastereoisomer: (25 °C, $CDCl_3$, 400 MHz): δ = 7.54–7.52 (m, 2H), 7.51–7.45 (m, 2H), 7.35–7.32 (m, 4H), 7.32–7.31 (m, 2H), 7.21–7.18 (m, 1H), 7.14–7.09 (m, 2H), 7.03–6.98 (m, 1H), 4.84 (dd, $J(^1H-^1H)$ = 12.9 Hz, $J'(^1H-^1H)$ = 11.2 Hz, 1H), 4.53 (dd, $J(^1H-^1H)$ = 12.9 Hz, $J'(^1H-^1H)$ = 4.0 Hz, 1H), 3.95 (dd, $J(^1H-^1H)$ = 10.3 Hz, $J'(^1H-^1H)$ = 4.6 Hz, 1H), 2.96 (d, $J(^1H-^1H)$ = 13.4 Hz, 1H), 2.81 (d, $J(^1H-^1H)$ = 13.4 Hz, 1H), 2.29 (s, 3H) ppm. **^{13}C NMR of minor diastereoisomer** (25 °C, $CDCl_3$, 100.61 MHz): δ = 172.7, 159.8, 136.9, 132.7, 132.2 (2C), 131.7, 129.4 (2C), 129.0 (2C), 128.9 (2C), 128.6 (2C), 127.8, 126.1, 123.4, 119.9 (2C), 76.1, 61.2, 47.0, 40.1, 15.2 ppm. LRMS (ESI) m/z: calcd for $C_{25}H_{22}BrN_3$ N_3Na $[M+Na]^+$ 514.1; found 514.2.

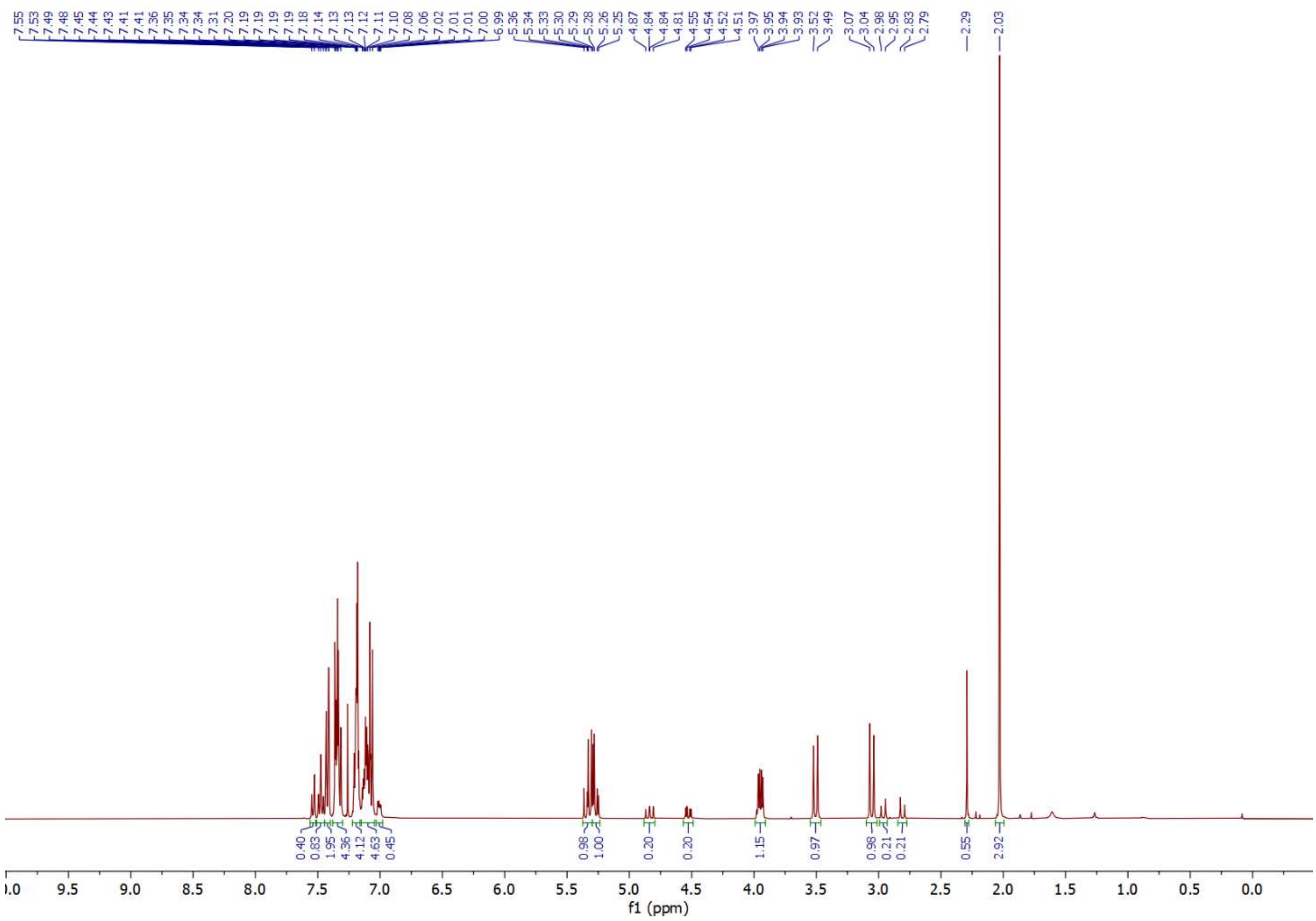

Supplementary Figure S85. The ^{1}H (400.1 MHz) NMR spectrum of 6c.

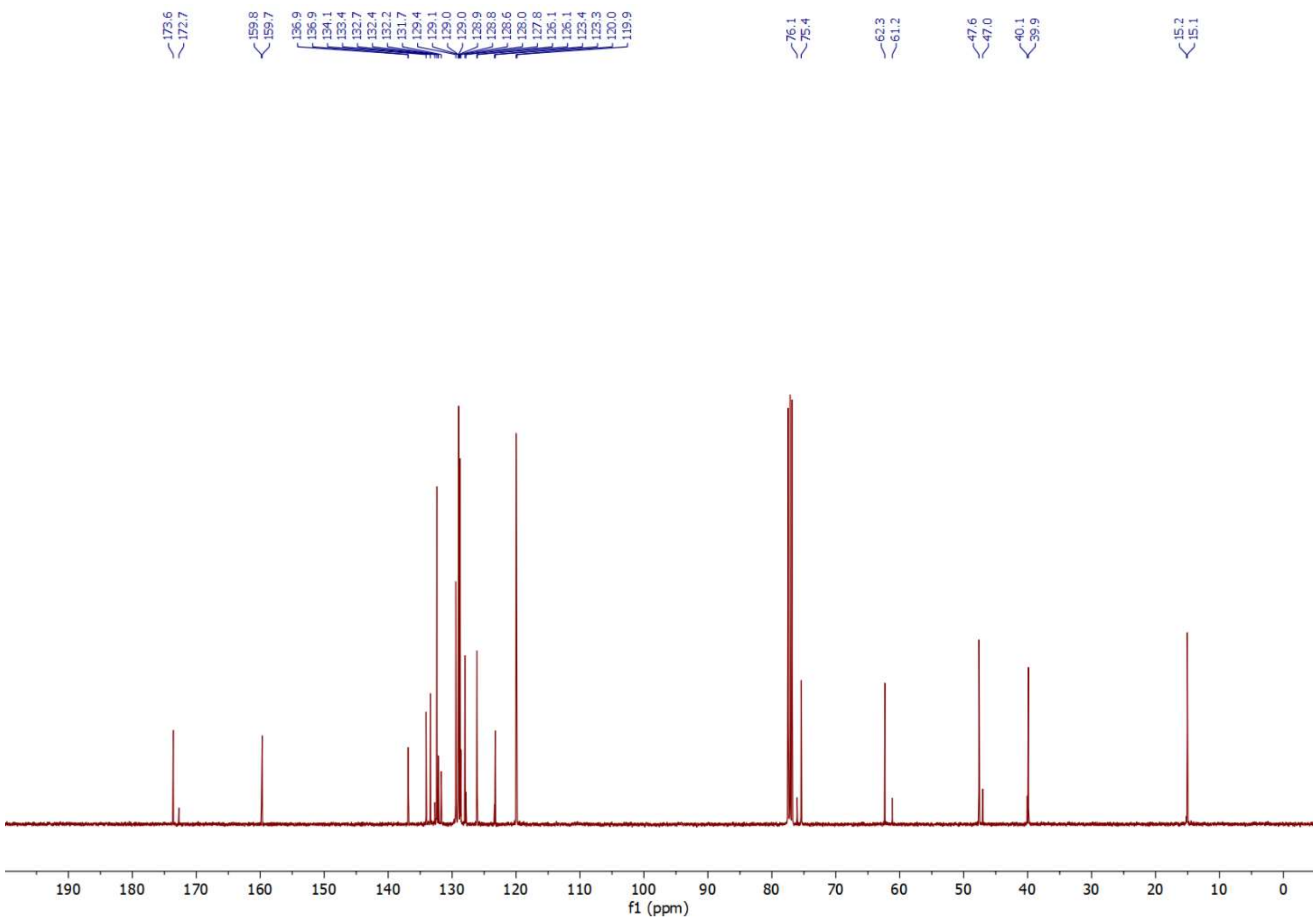

Supplementary Figure S86. The ^{13}C{^{1}H} (100.6 MHz) NMR spectrum of 6c.

4-Benzyl-5-methyl-4-(1-(4-chlorophenyl)-2-nitroethyl)-2-phenyl-2,4-dihydr*o*-3*H*-pyrazol-3-one (6d)

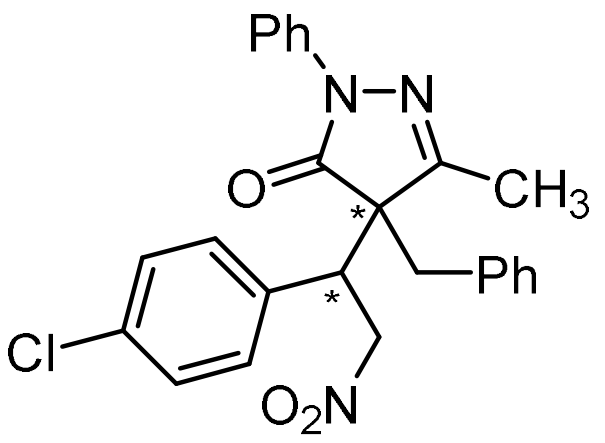

Compound was prepared and isolated by **Procedure H**. Colorless oil. Yield 96% (43 mg), *Dr*. 5:1. **^{1}H NMR of major diastereoisomer**: (25 °C, $CDCl_3$, 400 MHz): δ = 7.44–7.40 (m, 2H), 7.35–7.31 (m, 2H), 7.22–7.17 (m, 6H), 7.15–7.10 (m, 4H), 5.34 (dd, $J(^1H-^1H)$ = 13.5 Hz, $J'(^1H-^1H)$ = 10.3 Hz, 1H), 5.27 (dd, $J(^1H-^1H)$ = 13.6 Hz, $J'(^1H-^1H)$ = 4.5 Hz, 1H), 3.96 (dd, $J(^1H-^1H)$ = 10.3 Hz, $J'(^1H-^1H)$ = 4.6 Hz, 1H), 3.51 (d, $J(^1H-^1H)$ = 13.2 Hz, 1H), 3.06 (d, $J(^1H-^1H)$ = 13.2 Hz, 1H), 2.03 (s, 3H) ppm. **^{13}C NMR of major diastereoisomer**: (25 °C, $CDCl_3$, 100.61 MHz): δ = 173.6, 159.7, 136.9, 135.1, 133.5, 133.4, 129.4 (2C), 129.1 (4C), 129.0 (2C), 128.8 (2C), 128.0, 126.2, 120.0 (2C), 75.5, 62.4, 47.6, 39.9, 15.1 ppm.

^{1}H NMR of minor diastereoisomer: (25 °C, $CDCl_3$, 400 MHz): δ = 7.54–7.51 (m, 2H), 7.50–7.47 (m, 2H), 7.40–7.36 (m, 2H), 7.35–7.31 (m, 3H), 7.16–7.08 (m, 3H), 7.04–6.99 (m, 2H), 4.84 (dd, $J(^1H-^1H)$ = 12.9 Hz, $J'(^1H-^1H)$ = 11.2 Hz, 1H), 4.53 (dd, $J(^1H-^1H)$ = 13.0 Hz, $J'(^1H-^1H)$ = 4.0 Hz, 1H), 3.96 (dd, $J(^1H-^1H)$ = 10.3 Hz, $J'(^1H-^1H)$ = 4.6 Hz, 1H), 2.96 (d, $J(^1H-^1H)$ = 13.5 Hz, 1H), 2.81 (d, $J(^1H-^1H)$ = 13.4 Hz, 1H), 2.29 (s, 3H) ppm. **^{13}C NMR of minor diastereoisomer** (25 °C, $CDCl_3$, 100.61 MHz): δ = 172.8, 159.8, 136.9, 135.2, 132.2, 131.4, 129.2 (2C), 129.1, 129.0 (4C), 128.6 (2C), 127.8, 126.2, 126.1, 119.9 (2C), 76.1, 61.3, 47.0, 40.1, 15.2 ppm. LRMS (ESI) m/z: calcd for $C_{25}H_{22}ClN_3NaO_3$ $[M+Na]^+$ calc for 470.1; found 470.1.

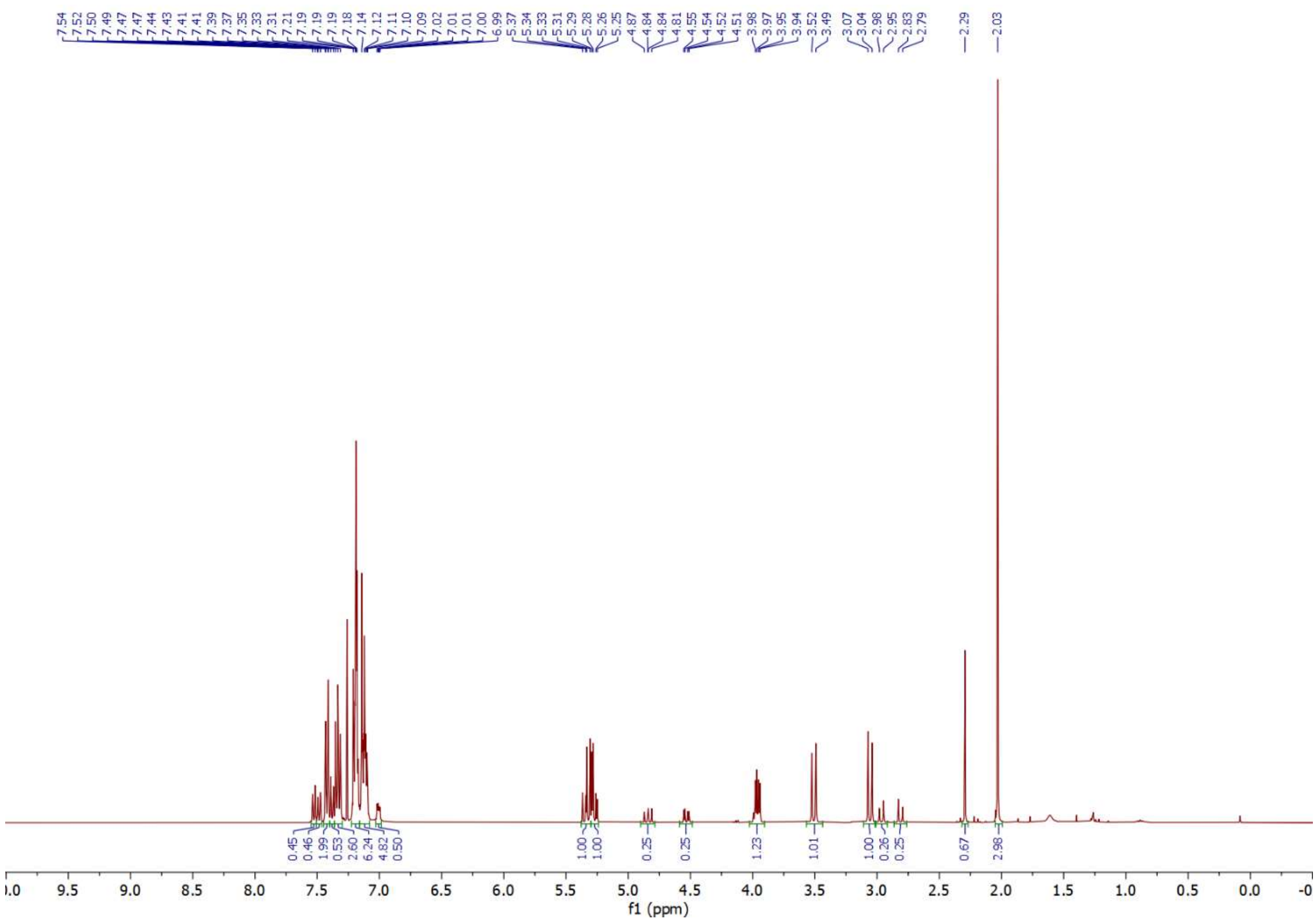

Supplementary Figure S87. The ^{1}H (600.1 MHz) NMR spectrum of 6d.

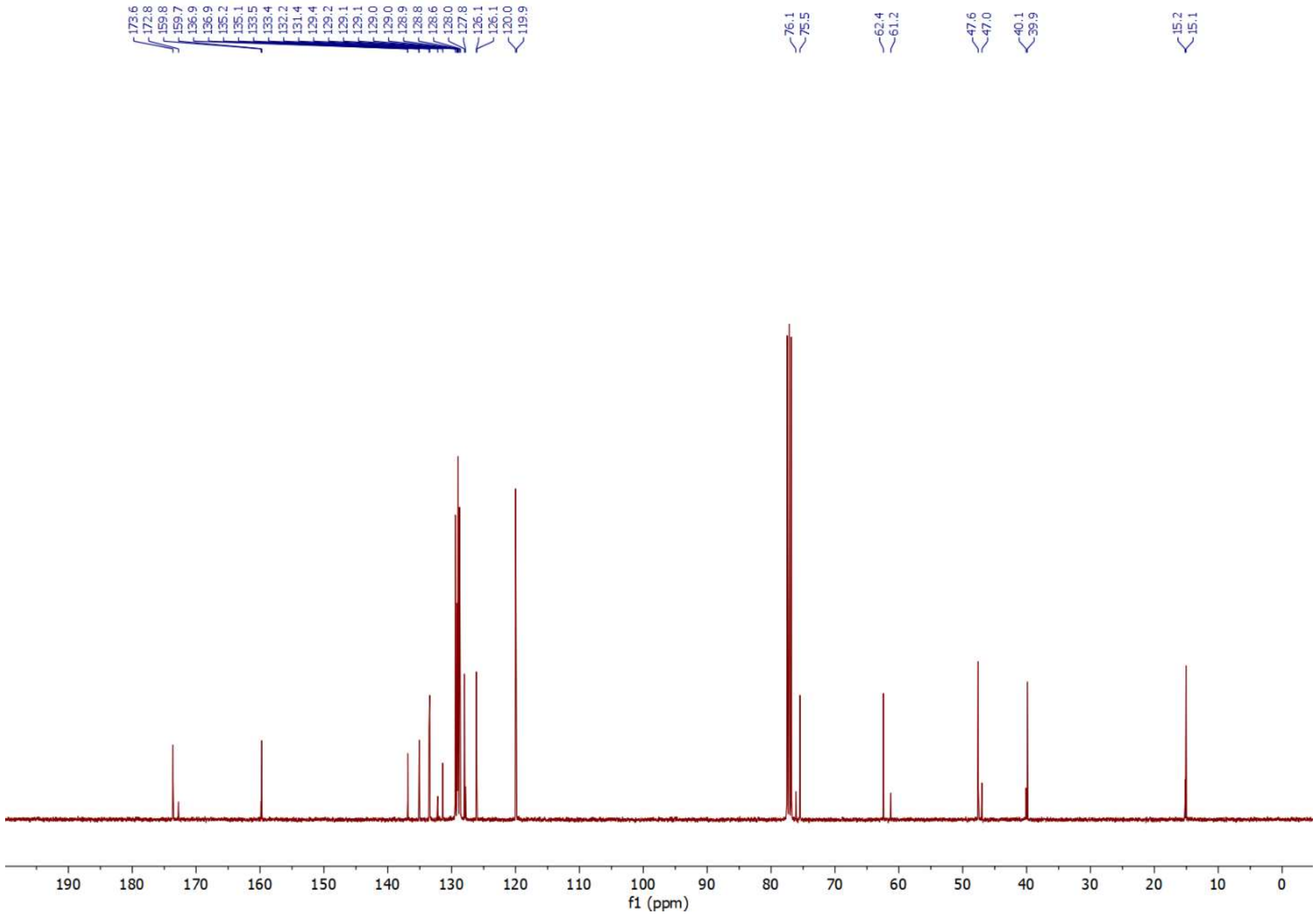

Supplementary Figure S88. The $^{13}C\{^1H\}$ (150.9 MHz) NMR spectrum of 6d.

4-(1-(4-Benzyl-3-methyl-5-oxo-1-phenyl-4,5-dihydro-1*H*-pyrazol-4-yl)-2-nitroethyl)benzonitrile (6e)

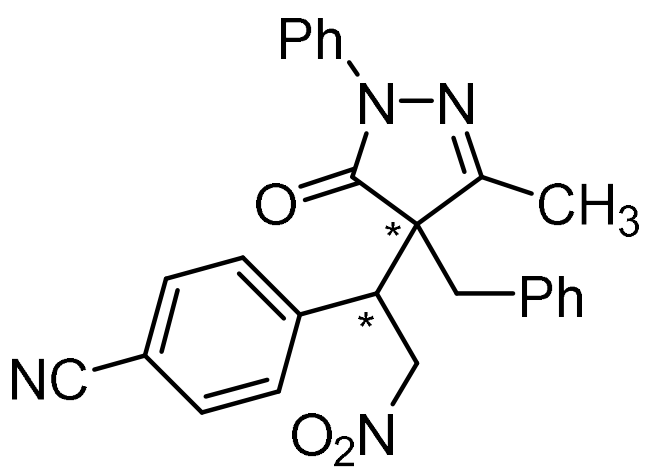

Compound prepared according to the standard **Procedure H** after 16 h, as white semi-solid (40 mg, 91%); Dr 2:1. **^{1}H NMR of major diastereoisomer** (25 °C, 400 MHz, $CDCl_3$) δ = 7.79–7.67 (m, 2H), 7.55–7.50 (m, 2H), 7.42–7.38 (m, 2H), 7.37–7.30 (m, 3H), 7.21–7.18 (m, 3H), 7.12 – 7.08 (m, 2H), 5.39 (dd, $J(^1H-^1H)$ = 13.8 Hz, $J'(^1H-^1H)$ = 10.6 Hz, 2H), 5.30 (dd, $J(^1H-^1H)$ = 13.9 Hz, $J'(^1H-^1H)$ = 4.3 Hz, 1H), 4.04 (dd, $J(^1H-^1H)$ = 10.7 Hz, $J'(^1H-^1H)$ = 4.3 Hz, 1H), 3.53 (d, $J(^1H-^1H)$ = 13.2 Hz, 1H), 3.07 (d, $J(^1H-^1H)$ = 13.2 Hz, 1H), 2.04 (s, 3H) ppm; **^{13}C NMR of major diastereoisomer** (25 °C, 101 MHz, $CDCl_3$) δ = 173.3, 159.3, 140.3, 136.7, 133.1, 132.9 (2C), 129.1 (2C), 129.0 (2C), 128.9 (2C), 128.7 (2C), 128.1, 126.3, 119.8 (2C), 118.0, 113.3, 74.9, 62.3, 47.9, 39.7, 15.1 ppm.

^{1}H NMR of minor diastereoisomer (25 °C, 400 MHz, $CDCl_3$) δ = 7.48–7.44 (m, 2H), 7.37–7.30 (m, 4H), 7.23–7.20 (m, 3H), 7.16–7.09 (m, 3H), 7.03–6.96 (m, 2H), 4.87 (dd, $J(^1H-^1H)$ = 13.2 Hz, $J'(^1H-^1H)$ = 11.3 Hz, 1H), 4.53 (dd, $J(^1H-^1H)$ = 13.2 Hz, $J'(^1H-^1H)$ = 3.8 Hz, 1H), 4.07 – 4.04 (m, 1H), 2.91 (d, $J(^1H-^1H)$ = 13.3 Hz, 1H), 2.82 (d, $J(^1H-^1H)$ = 13.3 Hz, 1H), 2.33 (s, 3H) ppm; **^{13}C NMR of minor diastereoisomer** (25 °C, 101 MHz, $CDCl_3$) δ = 172.4, 159.4, 139.1, 136.7, 133.0, 132.6 (2C), 130.9 (2C), 129.0 (2C), 128.9 (2C), 128.7 (2C), 128.0, 126.2, 119.8 (2C), 118.0, 113.3, 75.7, 61.0, 47.4, 40.2, 15.2 ppm. FT-IR (ATR): ν= 3033, 2921, 2233, 1695, 1554, 1376 cm^{-1}; HRMS (ESI) m/z calcd for $C_{26}H_{23}N_4O_3$ $[M+H]^+$ 439.1765, found 439.1769.

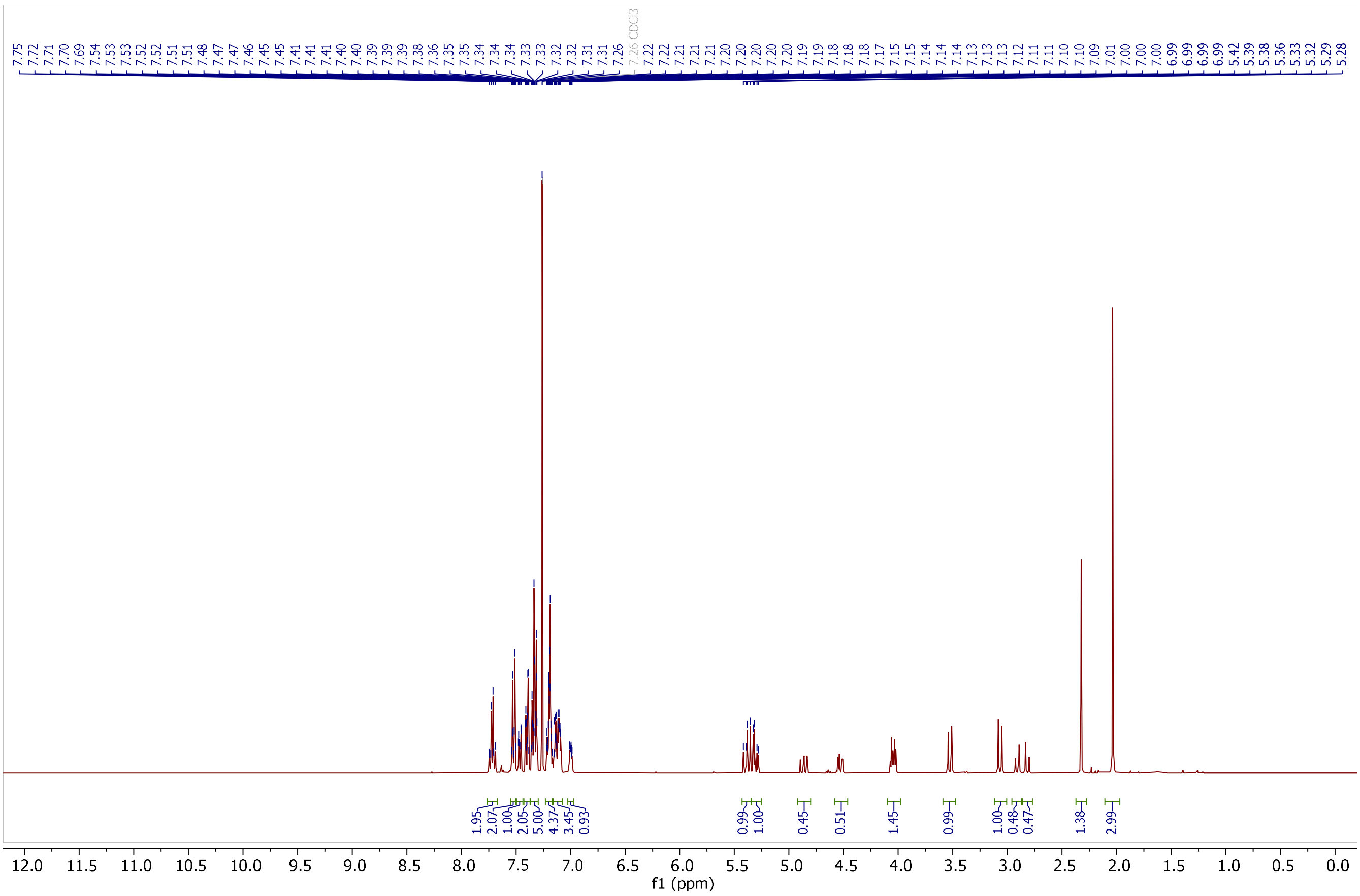

Supplementary Figure S89. The ^{1}H (400.1 MHz) NMR spectrum of 6e.

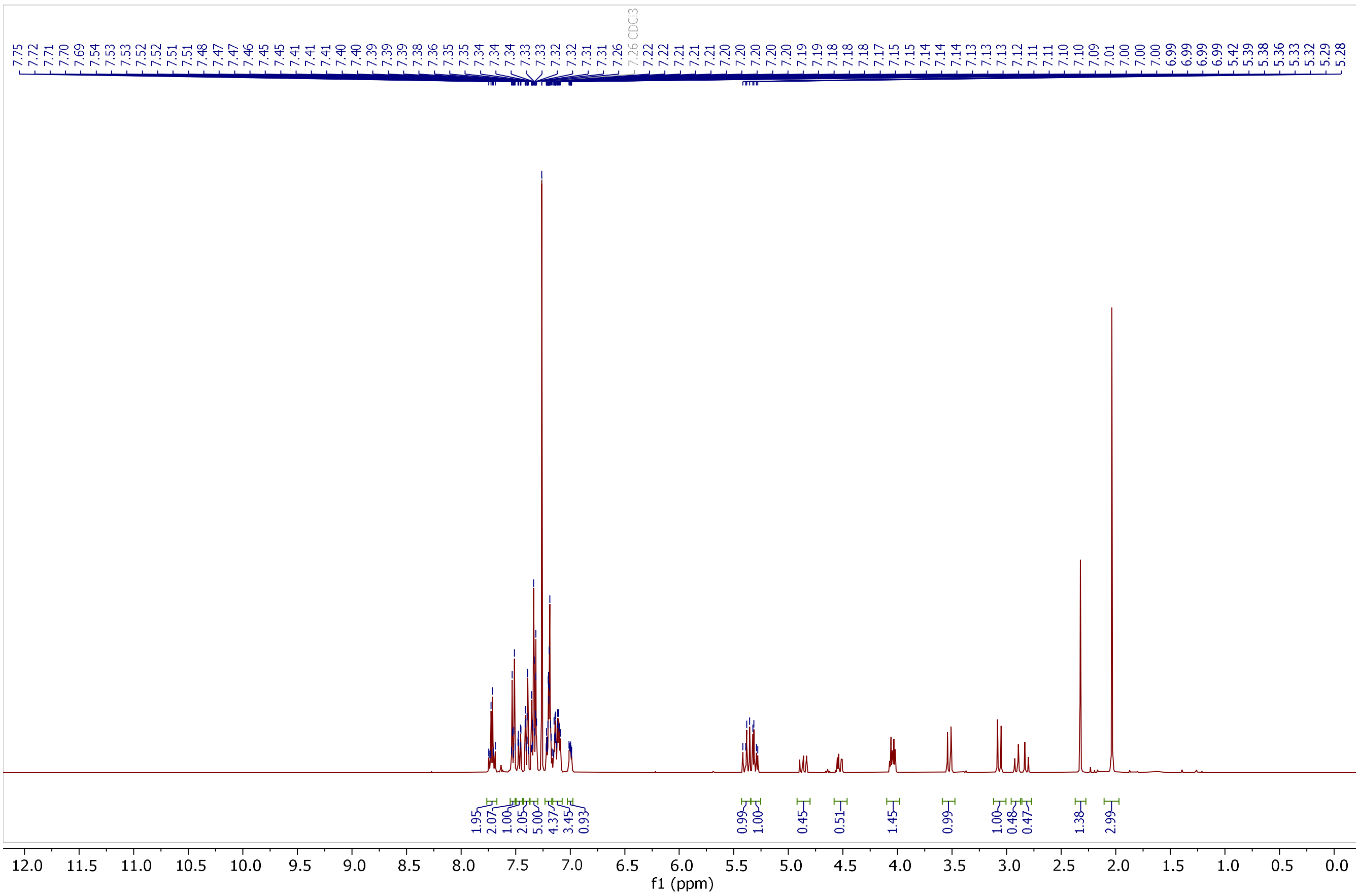

Supplementary Figure S90. The $^{13}C\{^{1}H\}$ (100.6 MHz) NMR spectrum of 6e.

Methyl 4-(1-(4-benzyl-3-methyl-5-oxo-1-phenyl-4,5-dihydro-1*H*-pyrazol-4-yl)-2-nitroethyl)benzoate (6f)

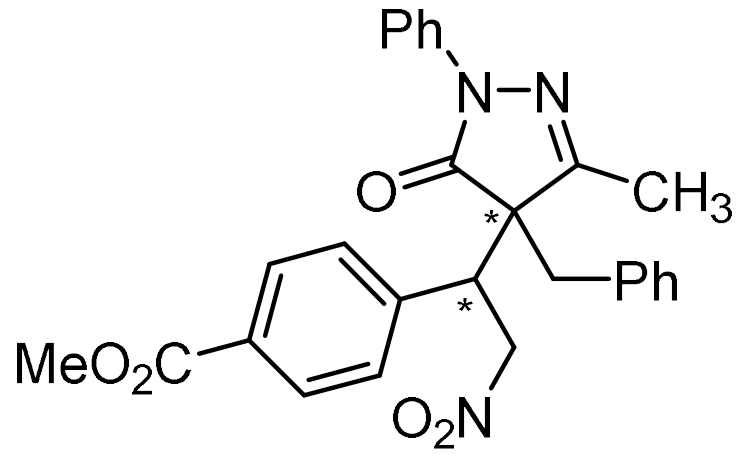

Compound prepared according to the standard **Procedure H** after 16 h as white semi-solid (40 mg, 85%); Dr 3.5:1. **^{1}H NMR of major diastereoisomer** (25 °C, 400 MHz, $CDCl_3$) δ = 7.93–7.84 (m, 2H), 7.44–7.37 (m, 2H), 7.36–7.27 (m, 5H), 7.21–7.07 (m, 5H), 5.45–5.27 (m, 1H), 4.04 (dd, J(^{1}H-^{1}H) = 10.0 Hz, J'(^{1}H-^{1}H) = 4.8 Hz, 1H), 3.86 (s, 3H), 3.53 (d, J(^{1}H-^{1}H) = 13.1 Hz, 1H), 3.07 (d, J(^{1}H-^{1}H) = 13.2 Hz, 1H), 2.02 (s, 3H) ppm; **^{13}C NMR of major diastereoisomer** (25 °C, 101 MHz, $CDCl_3$) δ = 173.6, 166.3, 159.6, 140.1, 136.8, 133.4, 130.4 (2C), 129.1 (2C), 129.0 (4C), 128.8 (2C), 128, 127.9, 126.1, 120.0 (2C), 75.4, 62.3, 52.3, 48.0, 39.9, 15.1 ppm.

^{1}H NMR of minor diastereoisomer (25 °C, 400 MHz, $CDCl_3$) δ = 8.07 (d, J(^{1}H-^{1}H) = 8.5 Hz, 2H), 7.66 (d, J(^{1}H-^{1}H) = 8.4 Hz, 2H), 7.52–7.44 (m, 2H), 7.14–7.07 (m, 6H), 7.03–6.97 (m, 2H), 4.91 (dd, J(^{1}H-^{1}H) = 13.0, J'(^{1}H-^{1}H) = 11.1 Hz, 1H), 4.59 (dd, J(^{1}H-^{1}H) = 13.1 Hz, J'(^{1}H-^{1}H) = 4.0 Hz, 1H), 4.08 – 4.03 (m, 1H), 3.92 (s, 3H), 2.95 (d, J(^{1}H-^{1}H) = 13.4 Hz, 1H), 2.84 (d, J(^{1}H-^{1}H) = 13.4 Hz, 1H), 2.30 (s, 3H) ppm; **^{13}C NMR of minor diastereoisomer** (25 °C, 101 MHz, $CDCl_3$) δ = 172.7, 166.6, 159.7, 138.8, 136. 9, 133.4, 130.8, 130.1 (2C), 129.0 (2C), 128.9 (3C), 128.6 (2C), 128.0, 127.9, 126.1, 119.8 (2C), 75.9, 61.2, 52.3, 47.4, 39.9, 15.3 ppm. FT-IR (ATR): ν= 2951, 2921, 1716, 1695, 1552, 1279 cm^{-1}; HRMS (ESI) m/z calcd for $C_{27}H_{26}N_3O_5$ $[M+H]^+$ 472.1867, found 472.1872.

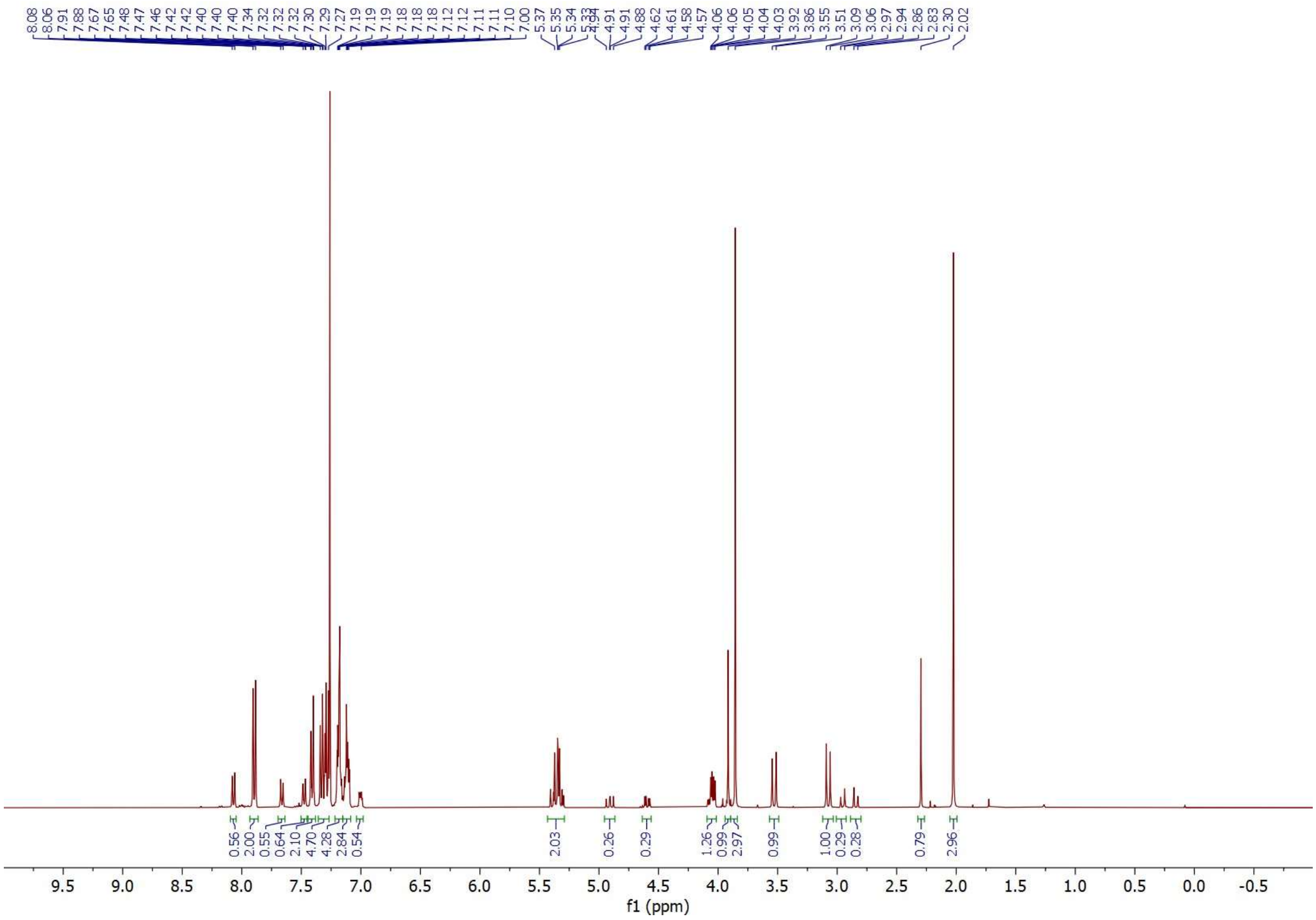

Supplementary Figure S91. The ^{1}H (400.1 MHz) NMR spectrum of 6f.

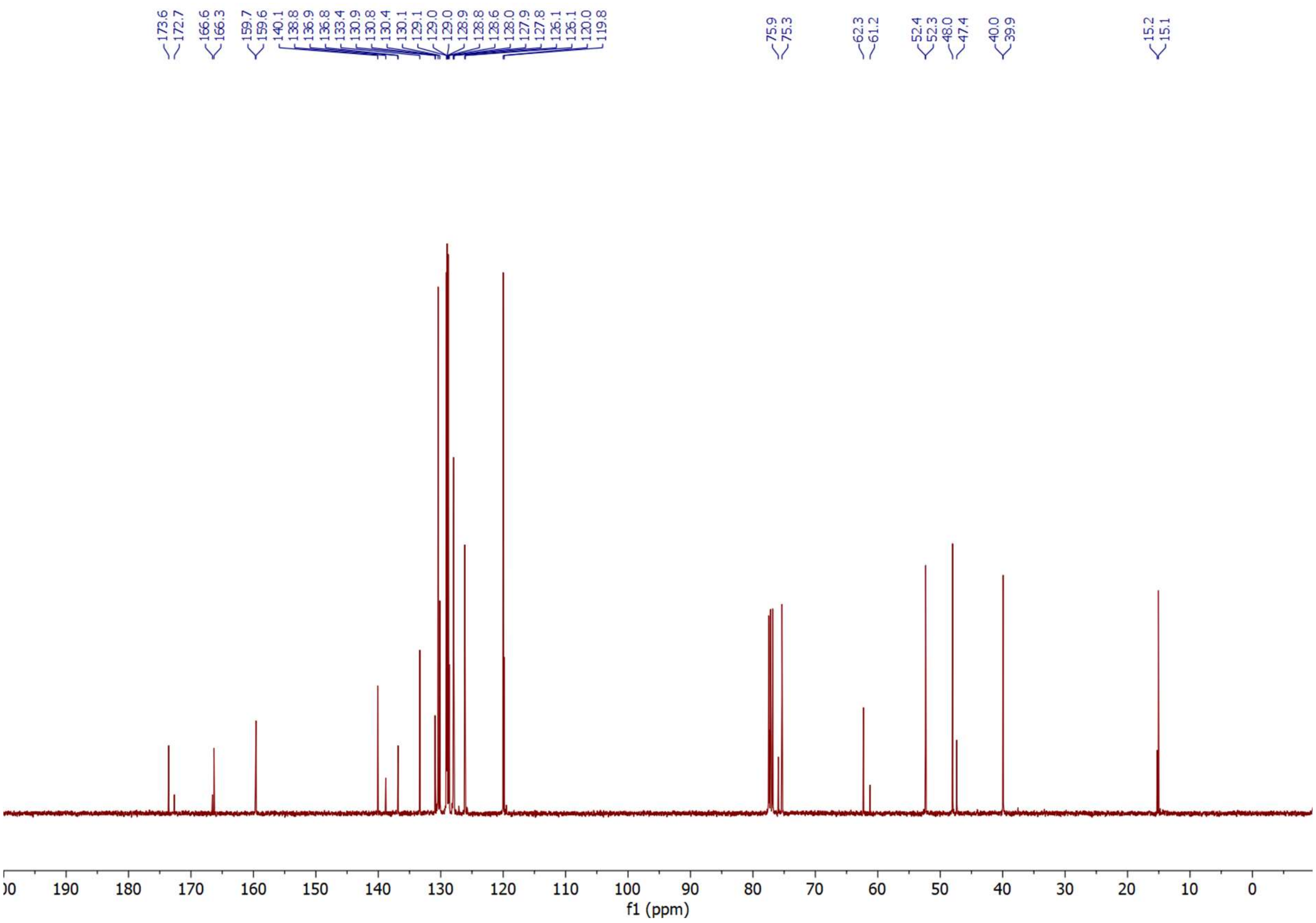

Supplementary Figure S92. The $^{13}C\{^1H\}$ (100.6 MHz) NMR spectrum of 6f.

4-Benzyl-5-fenyl-4-(1-(phenyl)-2-nitroethyl)-2-phenyl-2,4-dihydr*o*-3*H*-pyrazol-3-one (6g)

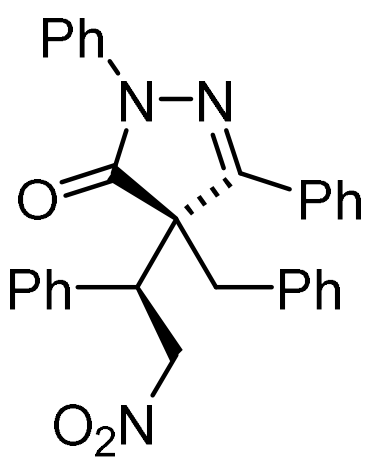

Compound was prepared and isolated by **Procedure H**. White semi-solid. Yield 94% (45 mg), *Dr*. 10:1. m. p. 156-158°C; **^{1}H NMR of major diastereoisomer:** (25 °C, $CDCl_3$, 600 MHz): δ = 7.88 (d, *J*(^{1}H-^{1}H) = 7.9 Hz, 2H), 7.55–7.48 (m, 3H), 7.45–7.40 (m, 2H), 7.33 (t, *J*(^{1}H-^{1}H) = 7.9 Hz, 2H), 7.20 (t, *J*(^{1}H-^{1}H) = 7.4 Hz, 1H), 7.14–7.06 (m, 2H), 7.05–6.95 (m, 6H), 6.87 (d, *J*(^{1}H-^{1}H) = 7.5 Hz, 2H), 5.55 (dd, *J*(^{1}H-^{1}H) = 13.8 Hz, *J´*(^{1}H-^{1}H) = 4.8 Hz, 1H), 5.41 (dd, *J*(^{1}H-^{1}H) = 13.8 Hz, *J´*(^{1}H-^{1}H) = 9.6 Hz, 1H), 4.54 (dd, *J*(^{1}H-^{1}H) = 9.6 Hz, *J´*(^{1}H-^{1}H) = 4.8 Hz, 1H), 3.66 (d, *J*(^{1}H-^{1}H) = 13.1 Hz, 1H), 3.62 (d, *J*(^{1}H-^{1}H) = 13.2 Hz, 1H) ppm. **^{13}C NMR of major diastereoisomer**: (25 °C, $CDCl_3$, 150.9 MHz): δ = 174.2, 157.5, 136.9, 134.6, 133.5, 131.4, 130.7, 129.4 (2C), 129.1 (2C), 128.9 (2C), 128.7, 128.6 (2C), 128.4 (2C), 128.0 (2C), 127.7, 126.7 (2C), 126.3, 120.5 (2C), 75.2, 62.6, 48.1, 40.5 ppm. **^{1}H NMR of minor diastereoisomer**: (25 °C, $CDCl_3$, 600 MHz): δ = 8.07 (d, *J*(^{1}H-^{1}H) = 7.8 Hz, 1H), 7.62–7.58 (m, 3H), 7.55–7.53 (m, 4H), 7.39–7.35 (m, 4H), 7.20 (t, *J*(^{1}H-^{1}H) = 7.4 Hz, 1H), 7.11–7.1 (m, 1H), 7.05–6.96 (m, 2H), 6.87 (d, *J*(^{1}H-^{1}H) = 7.6 Hz, 2H), 6.94 (d, *J*(^{1}H-^{1}H) = 7.1 Hz, 2H), 4.90 (dd, *J*(^{1}H-^{1}H) = 13.0 Hz, *J´*(^{1}H-^{1}H) = 11.2 Hz, 1H), 4.62 (dd, *J*(^{1}H-^{1}H) = 13.0 Hz, *J´*(^{1}H-^{1}H) = 4.4 Hz, 1H), 4.50 (dd, *J*(^{1}H-^{1}H) = 11.2 Hz, *J´*(^{1}H-^{1}H) = 4.4 Hz, 1H), 3.48 (d, *J*(^{1}H-^{1}H) = 13.7 Hz, 1H), 3.16 (d, *J*(^{1}H-^{1}H) = 13.6 Hz, 1H) ppm. **^{13}C NMR of minor diastereoisomer**: (25 °C, $CDCl_3$, 150.9 MHz): δ = 173.3, 157.7, 137.0, 133.6, 131.2, 131.1, 130.1 (2C), 129.4 (2C), 129.1 (2C), 129.0, 128.9 (2C), 128.8 (2C), 128.2, 128.3 (2C), 127.6, 127.1 (2C), 126.2, 120.2 (2C), 76.1, 61.4, 48.0, 41.5 ppm. LRMS (ESI) m/z: calcd for $C_{30}H_{25}N_3NaO_3$ $[M+Na]^+$ 498.2; found 498.2. Compound **6g** was isolated in single-crystalline form. The material was dissolved in DCM, and crystallization was initiated by the addition of i-PrOH at room temperature.

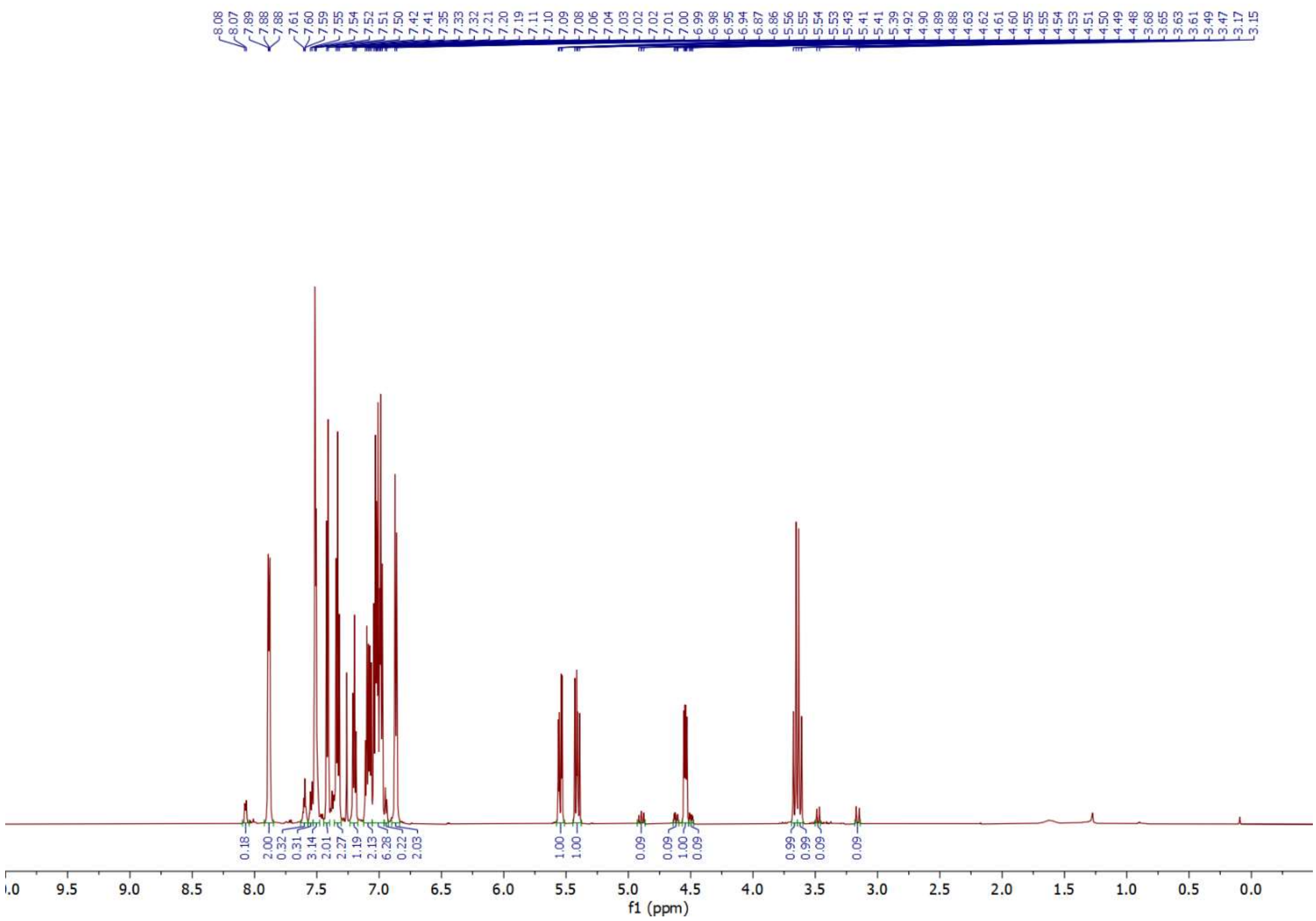

Supplementary Figure S93. The ^{1}H (600.1 MHz) NMR spectrum of 6g.

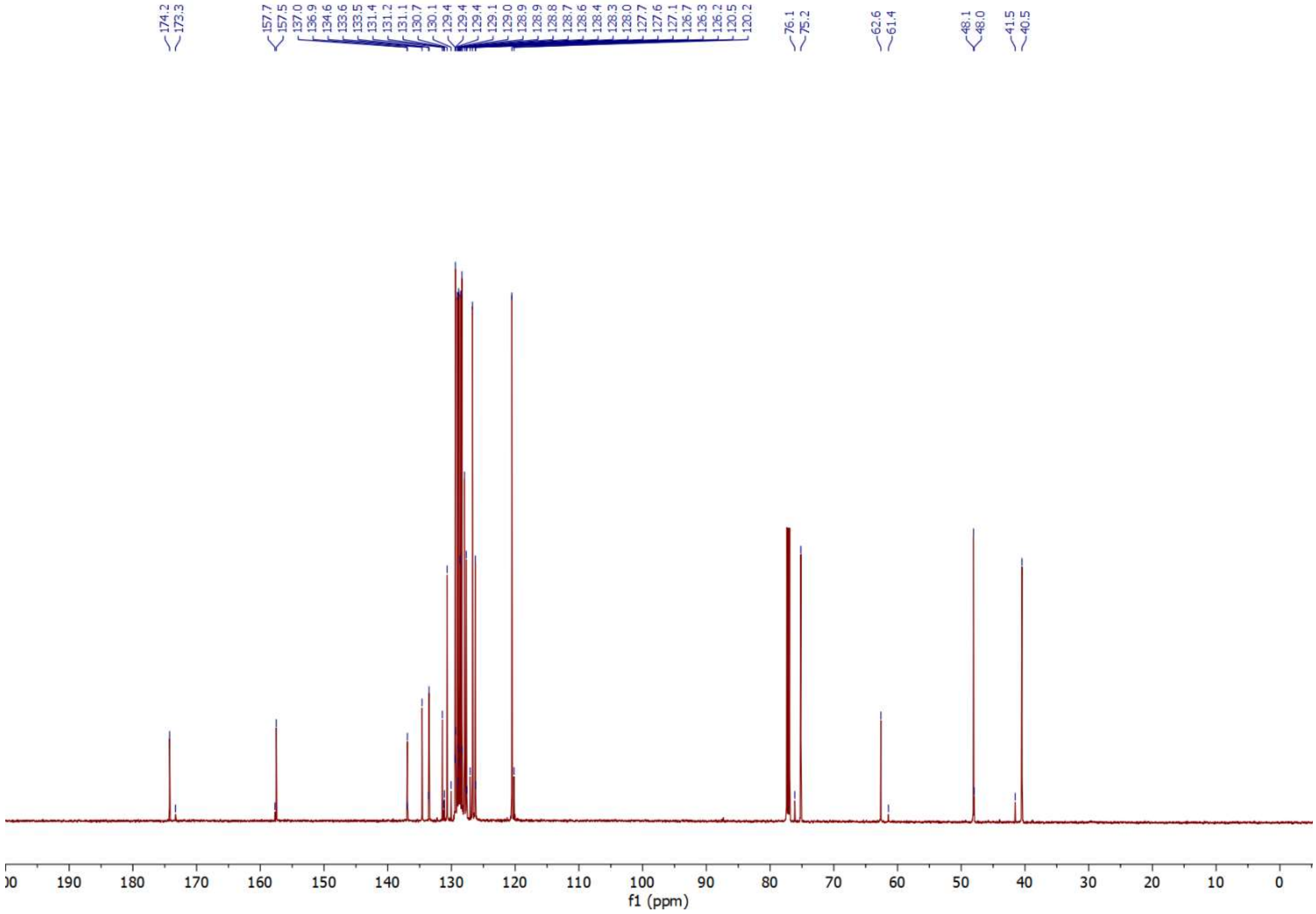

Supplementary Figure S94. The $^{13}C\{^{1}H\}$ (150.9 MHz) NMR spectrum of 6g.

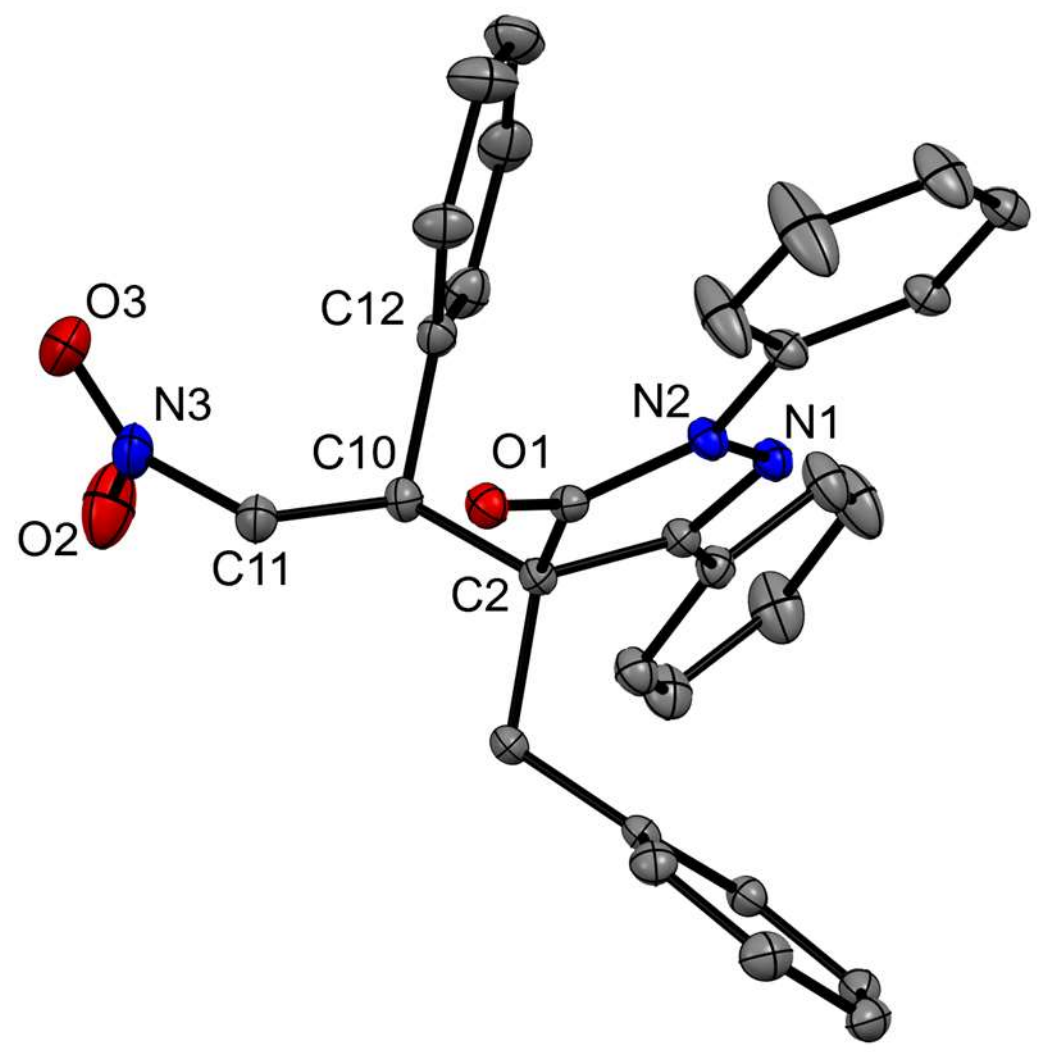

Supplementary Figure S95. The molecular structure of 6g. ORTEP view, 30% probability level. Selected interatomic distances [Å]: C10-C11 1.5267(18), C2-C10 1.5643(16), C10-C12 1.5291(17), C11-N3 1.5058(18). Selected angles[°]: N3-C11-C10 108.35(11), C11-C10-C12 112.13(10), C11-C10-C2 112.37(10).

Supplementary Table S16. Experimental details of 6g.

Crystal data	
Chemical formula	$C_{30}H_{25}N_3O_3$
M_r	475.53
Crystal system, space group	Triclinic, *P*-1
Temperature (K)	150
a, *b*, *c* (Å)	9.1775(2), 10.1248(3), 13.8189(4)
α, β, γ (°)	75.252(1), 79.542(1), 72.810(1)
V ($Å^3$)	1178.41(6)
Z	2
Radiation type	Mo*K*α
μ (mm^{-1})	0.09
Crystal size (mm)	0.59 × 0.30 × 0.27
Data collection	
Diffractometer	Bruker D8 - Venture
Absorption correction	Multi-scan<br>*SADABS2016*/2 - Bruker AXS area detector scaling and absorption correction Reference: Krause, L., Herbst-Irmer, R., Sheldrick G.M. & Stalke D., *J. Appl. Cryst.* 2015, 48, 3–10.
T_{min}, T_{max}	0.598, 0.746
No. of measured, independent and observed [$I > 2\sigma(I)$] reflections	52585, 5818, 4787
R_{int}	0.068
$(\sin \theta/\lambda)_{max}$ ($Å^{-1}$)	0.667
Refinement	
$R[F^2 > 2\sigma(F^2)]$, $wR(F^2)$, S	0.047, 0.128, 1.03
No. of reflections	5818
No. of parameters	325
No. of restraints	288
H-atom treatment	H-atom parameters constrained
$\Delta\rho_{max}$, $\Delta\rho_{min}$ (e $Å^{-3}$)	0.34, −0.26

4-Benzyl-3-methyl-4-(3-oxo-1,3-diphenylpropyl)-1-phenyl-2,4-dihydro-3H-pyrazol-3-one (6h)

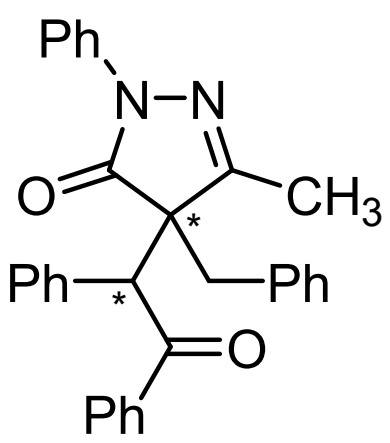

Compound was prepared and isolated by **Procedure H**. 10 mol% of catalyst *o*-**2** was used. Colorless oil. Yield 96% (44 mg), *Dr*. 2:1. **^{1}H NMR of major diastereoisomer**: (25 °C, $CDCl_3$, 600 MHz): δ = 7.84–7.79 (m, 2H), 7.67–7.61 (m, 2H), 7.60–7.54 (m, 2H), 7.52–7.50 (m, 1H), 7.40–7.32 (m, 5H), 7.20–7.09 (m, 6H), 7.08–7.03 (m, 2H), 3.99 (dd, $J(^1H-^1H)$ = 10.6 Hz, $J'(^1H-^1H)$ = 2.9 Hz, 1H), 3.80 (dd, $J(^1H-^1H)$ = 17.2 Hz, $J'(^1H-^1H)$ = 10.6 Hz, 1H), 2.94 (dd, $J(^1H-^1H)$ = 18.0Hz, $J'(^1H-^1H)$ = 2.9 Hz, 1H), 2.29 (s, 3H) ppm. **^{13}C NMR of major diastereoisomer**: (25 °C, $CDCl_3$, 150.9 MHz): δ = 197.1, 174.2, 161.8, 137.9, 137.3, 136.4, 134.7, 133.5, 128.9 (4C), 128.7 (2C), 128.4 (2C), 128.3 (4C), 128.1 (2C), 127.8, 127.3, 125.5, 119.7 (2C), 63.0, 44.3, 40.5, 39.6, 15.2 ppm. **^{1}H NMR of minor diastereoisomer:** (25 °C, $CDCl_3$, 600 MHz): δ = 8.07–8.01 (m, 2H), 7.47–7.45 (m, 2H), 7.37–7.32 (m, 9H), 7.29–7.23 (m, 2H), 7.19–7.16 (m, 5H), 4.18 (dd, $J(^1H-^1H)$ = 18.0 Hz, $J'(^1H-^1H)$ = 4.1 Hz, 1H), 4.09 (dd, $J(^1H-^1H)$ = 8.4 Hz, $J'(^1H-^1H)$ = 4.1 Hz, 1H), 3.94 (dd, $J(^1H-^1H)$ = 18.1 Hz, $J'(^1H-^1H)$ = 8.4 Hz, 1H), 3.51 (d, $J(^1H-^1H)$ = 13.5 Hz, 1H), 3.01 (d, $J(^1H-^1H)$ = 13.5 Hz, 1H), 2.11 (s, 3H) ppm. **^{13}C NMR of minor diastereoisomer:** (25 °C, $CDCl_3$, 150.9 MHz): δ = 198.5, 174.8, 161.7, 139.6, 137.9, 137.1, 136.7, 134.7, 130.3 (2C), 129.0 (2C), 128.8 (2C), 128.7 (2C), 128.6 (2C), 128.5 (2C), 127.9 (2C), 127.7, 127.4, 125.7, 120.2 (2C), 63.2, 45.0, 40.0, 38.1, 15.2 ppm. IR (ATR) ν_{max} = 3030, 1687, 1595, 1498, 1448, 1365, 1261, 1084 cm^{-1}; HRMS (ESI) m/z: calcd for $C_{32}H_{28}N_2NaO_2$ $[M+H]^+$ 495.2043; found 495.2041.

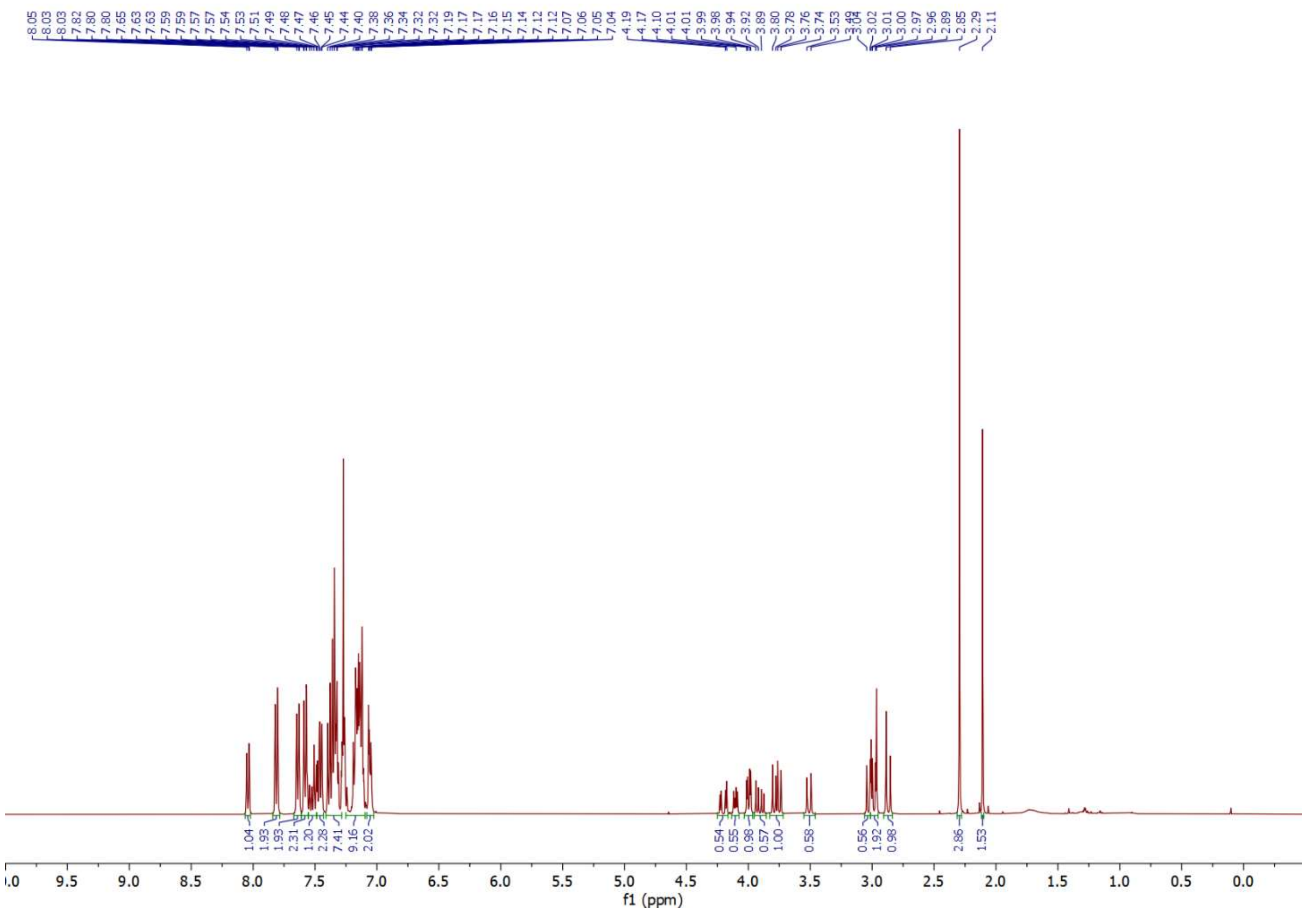

Supplementary Figure S96. The ^{1}H (600.1 MHz) NMR spectrum of 6h.

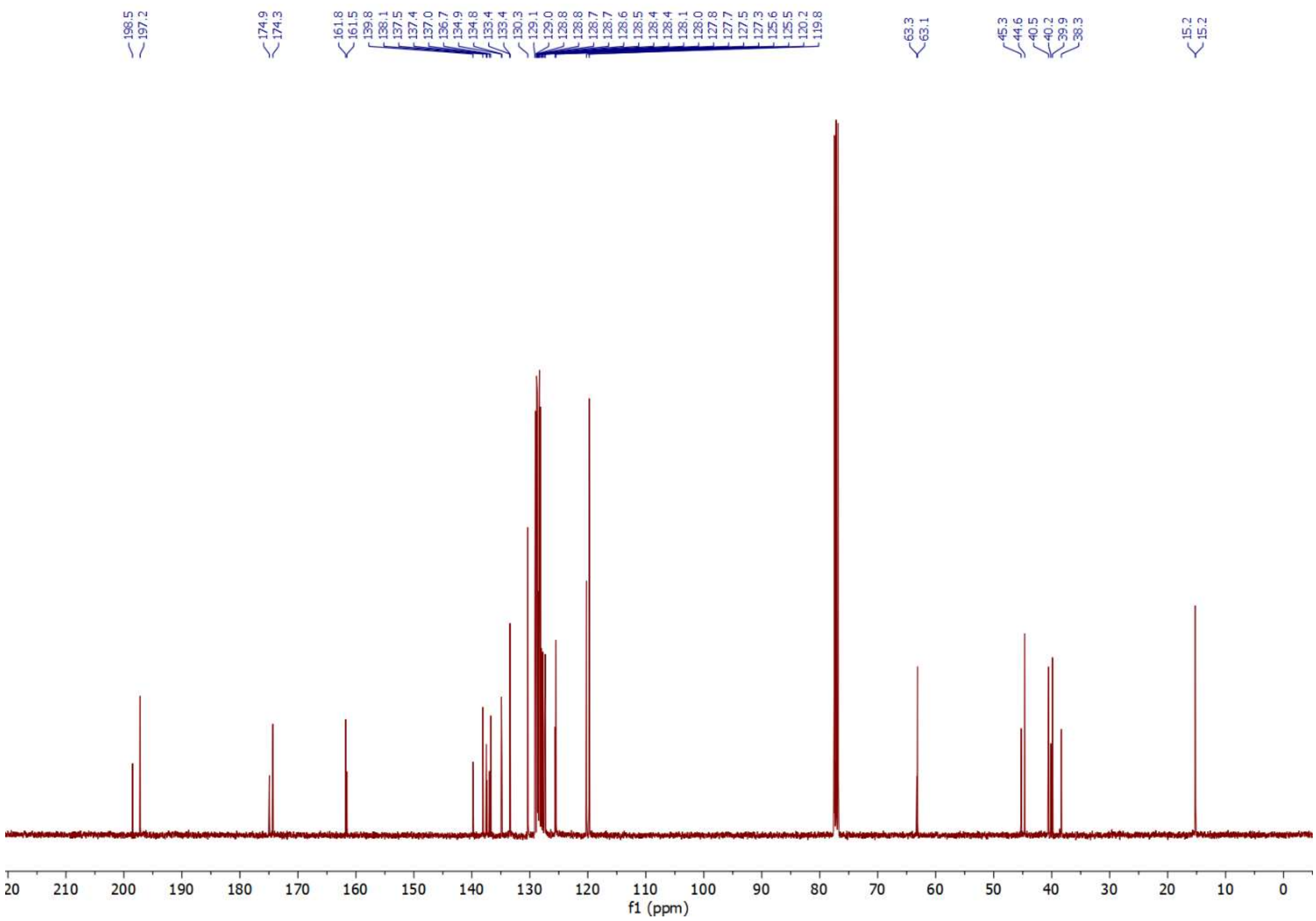

Supplementary Figure S97. The $^{13}C\{^{1}H\}$ (150.9 MHz) NMR spectrum of 6h.

Catalytic spirocyclization reaction (Procedure I)

To the vial equipped with magnetic stir bar was added catalyst *o*-**2** (0.9 mg, 0.001 mmol, 0.01 equiv.), followed by addition of corresponding dienone **7a**–**f** (0.15 mmol, 1.5 equiv.), dichloromethane (1 mL) and pyrazolone derivative **4a**–**c** (0.10 mmol, 1 equiv.). Reaction mixture was then sealed and heated to 45 °C for appropriate time (5–20 hours). After full conversion was reached, reaction mixture was directly loaded on the plug of silica a corresponding product **8a**–**h** was isolated using the mixture hexane:ethyl acetate.

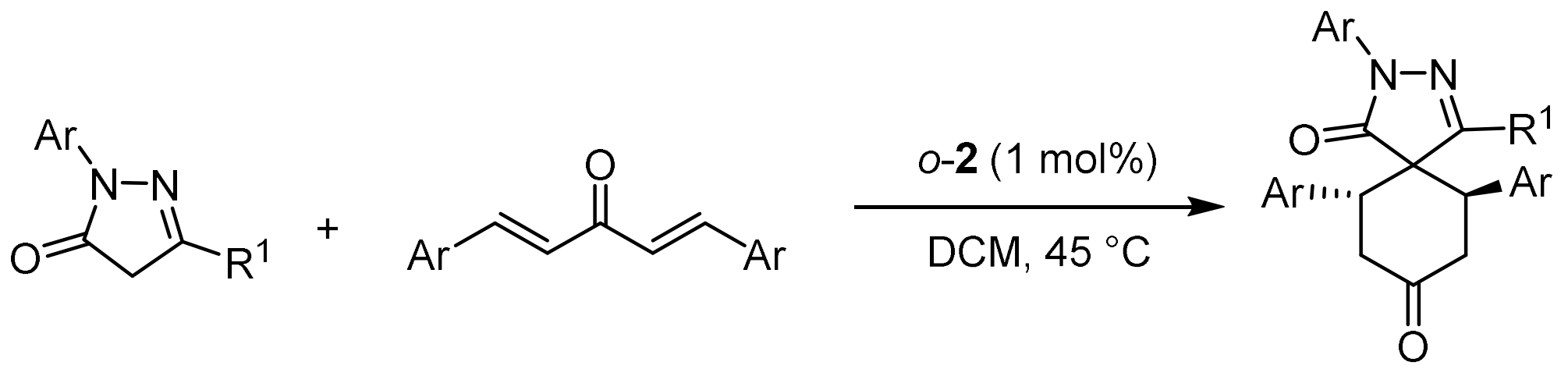

Supplementary Figure S98. Addition of pyrazolones 4a-c to dienones 7a-f.

3´-Methyl-1´,2,6-triphenyl-1*H*-spiro[cyclohexane-1,4´-pyrazole]-4,5´(4*H*)-dione (8a)[S17]

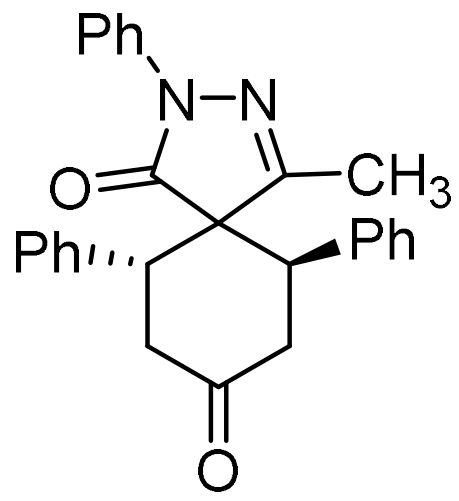

Compound was prepared and isolated by **Procedure I**. Amorphous solid. Yield 88% (36 mg), *Dr*. > 20:1. ^{1}**H NMR** (25 °C, $CDCl_3$, 400 MHz): δ = 7.51–7.45 (m, 2H), 7.37–7.28 (m, 5H), 7.23–7.10 (m, 8H), 3.98–3.82 (m, 2H), 3.67 (dd, $J(^1H\text{-}^1H)$ = 14.3 Hz, $J'(^1H\text{-}^1H)$ = 3.2 Hz, 1H), 3.38 (dd, $J(^1H\text{-}^1H)$ = 16.4 Hz, $J'(^1H\text{-}^1H)$ = 5.1 Hz, 1H), 2.98 (dd, $J(^1H\text{-}^1H)$ = 16.4 Hz, $J'(^1H\text{-}^1H)$ = 9.4 Hz, 1H), 2.73–2.55 (m, 1H), 1.67 (s, 3H) ppm. ^{13}**C NMR** (25 °C, $CDCl_3$, 100.61 MHz): δ = 209.4, 174.9, 160.7, 138.7, 137.3, 136.7, 129.1 (2C), 128.9 (2C), 128.8 (2C), 128.2(2C), 127.8 (4C), 125.8, 120.0 (2C), 61.9, 44.3, 42.6, 41.4, 40.7, 15.8 ppm. LRMS (ESI) m/z: $[M+H]^+$calcd for $C_{27}H_{25}N_2O_2$ 409.2; found 409.2.

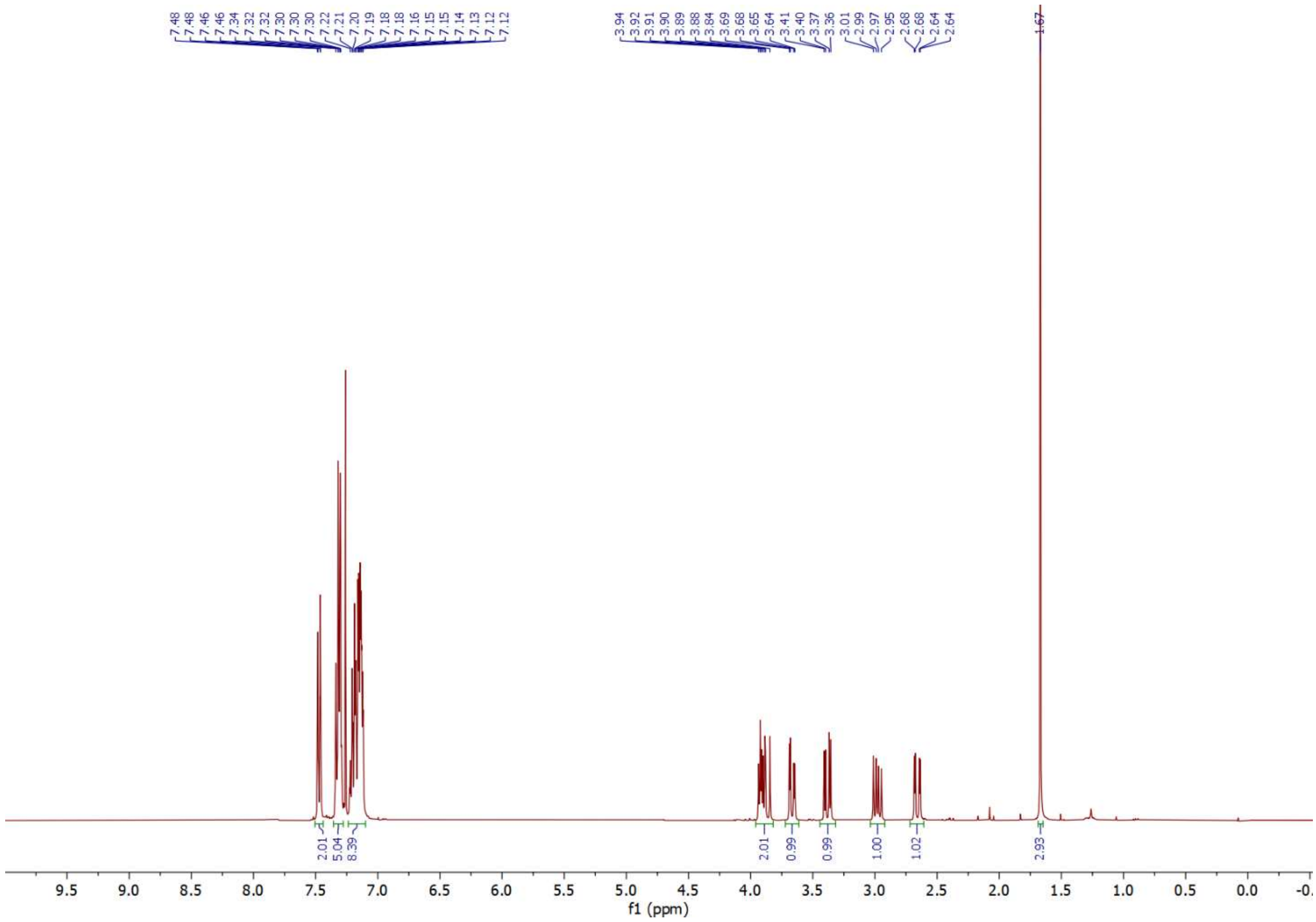

Supplementary Figure S99. The ^{1}H (400.1 MHz) NMR spectrum of 8a.

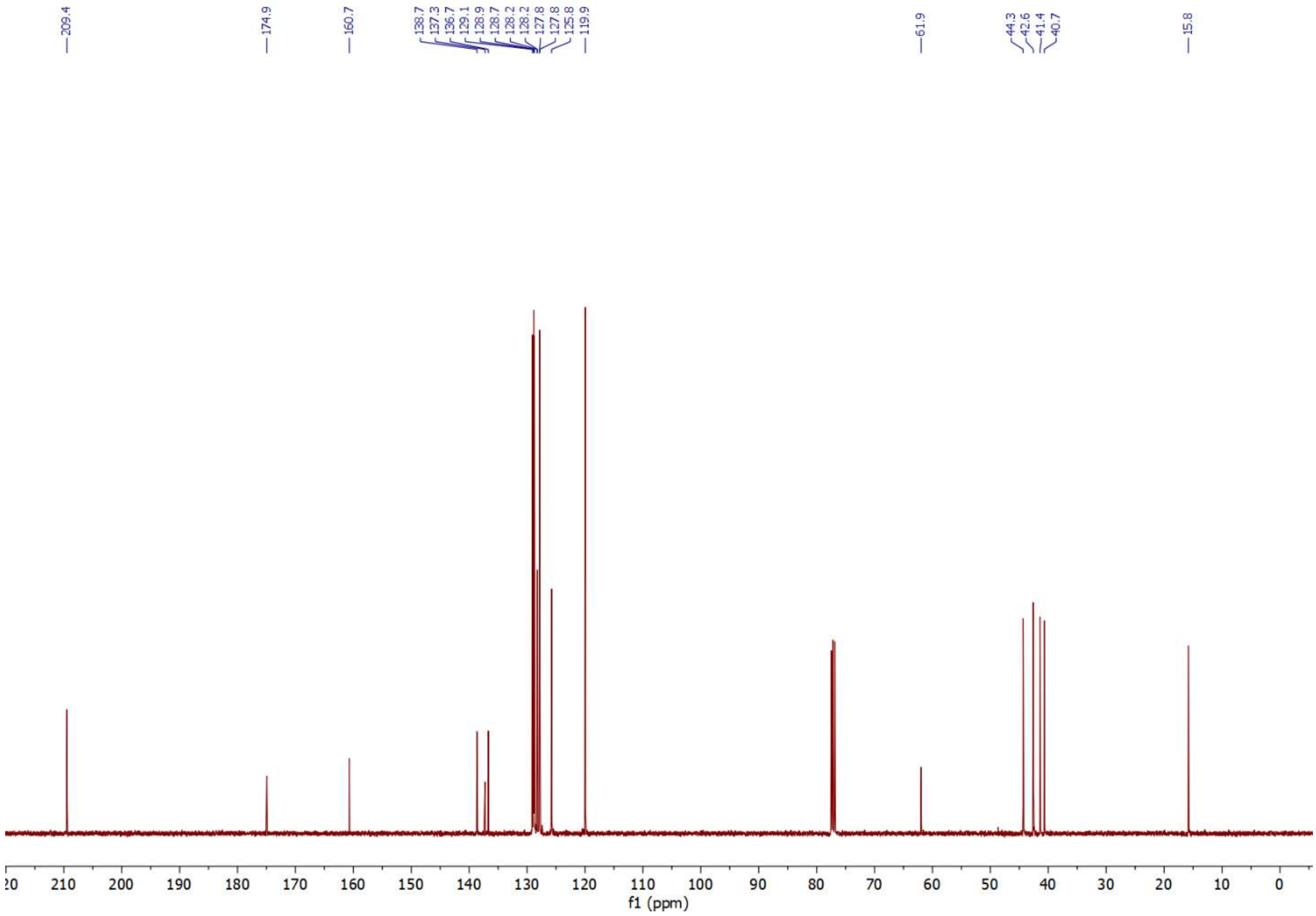

Supplementary Figure S100. The $^{13}C\{^{1}H\}$ (100.6 MHz) NMR spectrum of 8a.

3´-Methyl-2,6-bis(4-methylphenyl)-1´-phenyl-1*H*-spiro[cyclohexane-1,4´-pyrazole]-4,5´(4*H*)-dione (8b)

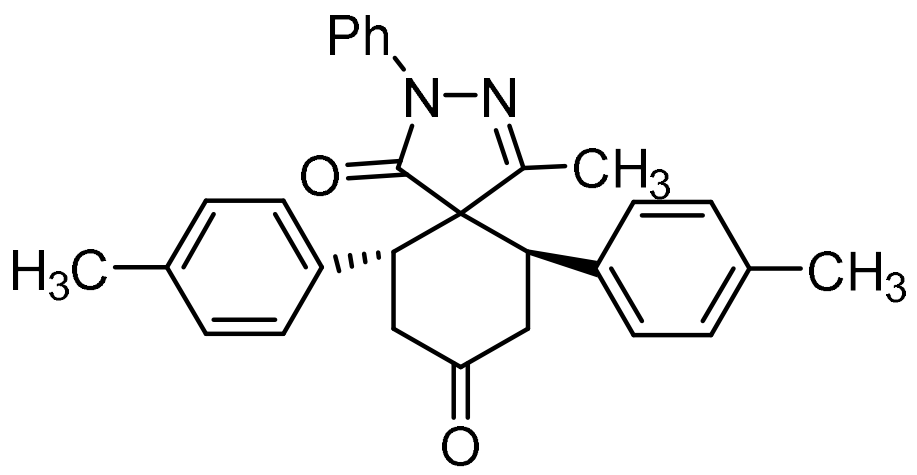

Compound was prepared and isolated by **Procedure I**. Amorphous solid. Yield 65% (28 mg), *Dr*. > 20:1. **^{1}H NMR** (25 °C, $CDCl_3$, 400 MHz): δ 7.56–7.47 (m, 2H), 7.39–7.30 (m, 2H), 7.21–7.14 (m, 1H), 7.12–7.10 (m, 2H), 7.05–6.97 (m, 6H), 3.90–3.80 (m, 2H), 3.63 (dd, $J(^1H\text{-}^1H)$ = 14.3 Hz, $J'(^1H\text{-}^1H)$ = 3.3 Hz, 1H), 3.40 (dd, $J(^1H\text{-}^1H)$ = 16.3 Hz, $J'(^1H\text{-}^1H)$ = 5.3 Hz, 1H), 2.91 (dd, $J(^1H\text{-}^1H)$ = 16.3 Hz, $J'(^1H\text{-}^1H)$ = 8.8 Hz, 1H), 2.62 (dd, $J(^1H\text{-}^1H)$ = 16.2 Hz, $J'(^1H\text{-}^1H)$ = 2.5 Hz, 1H), 2.33 (s, 3H), 2.20 (s, 3H), 1.63 (s, 3H) ppm. **^{13}C NMR** (25 °C, $CDCl_3$, 100.61 MHz): δ = 209.8, 175.1, 161.1, 137.9 (2C), 137.4, 135.8, 133.9, 129.7 (2C), 129.4 (2C), 128.9 (2C), 127.8 (2C), 127.7 (2C), 125.7, 120.0 (2C), 61.9, 44.3, 42.3, 41.8, 40.9, 21.2, 21.1, 15.9 ppm. LRMS (ESI) m/z: $[M+H]^+$calcd for $C_{27}H_{25}N_2O_2$ 437.2; found 437.2.

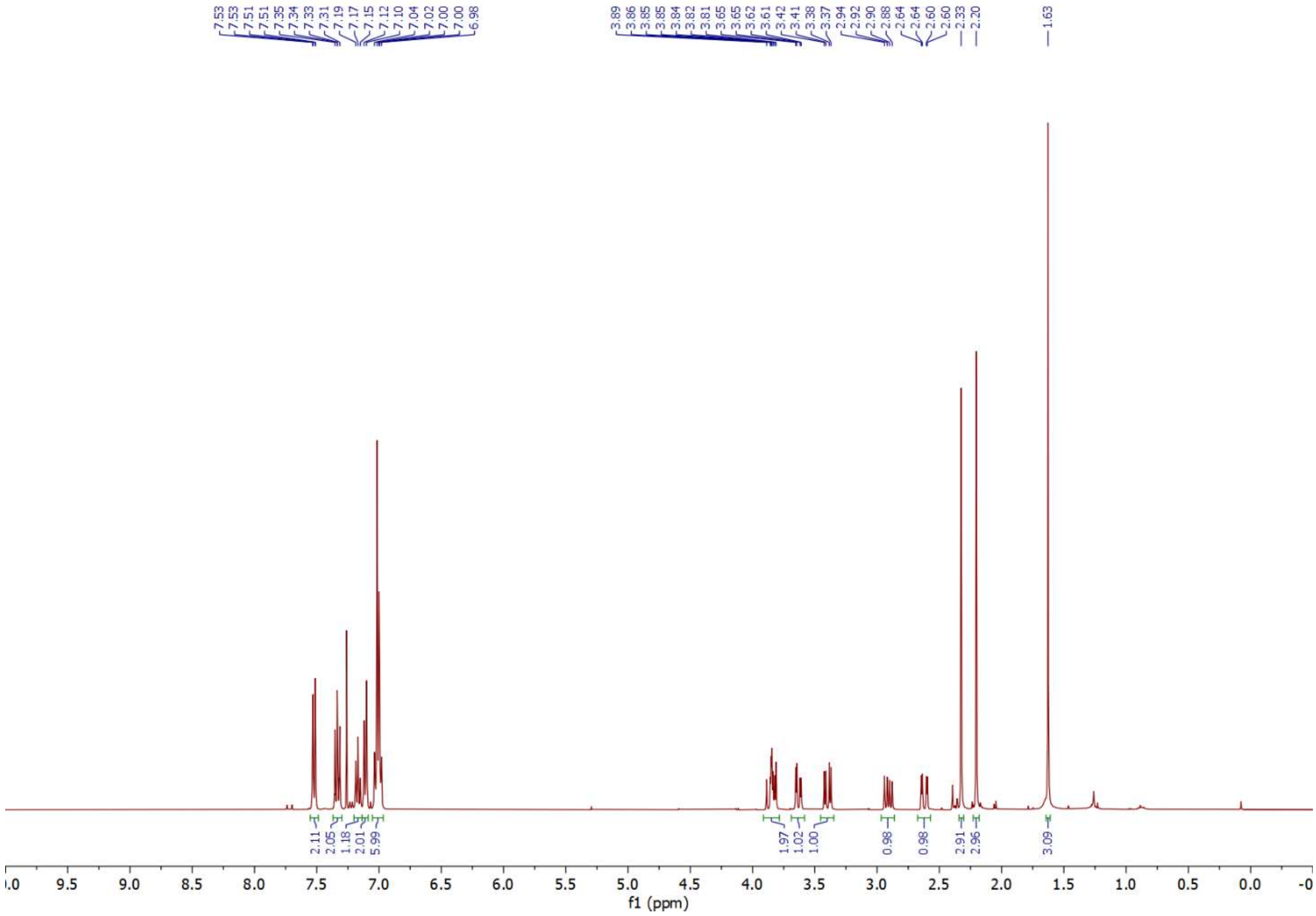

Supplementary Figure S101. The ^{1}H (400.1 MHz) NMR spectrum of 8b.

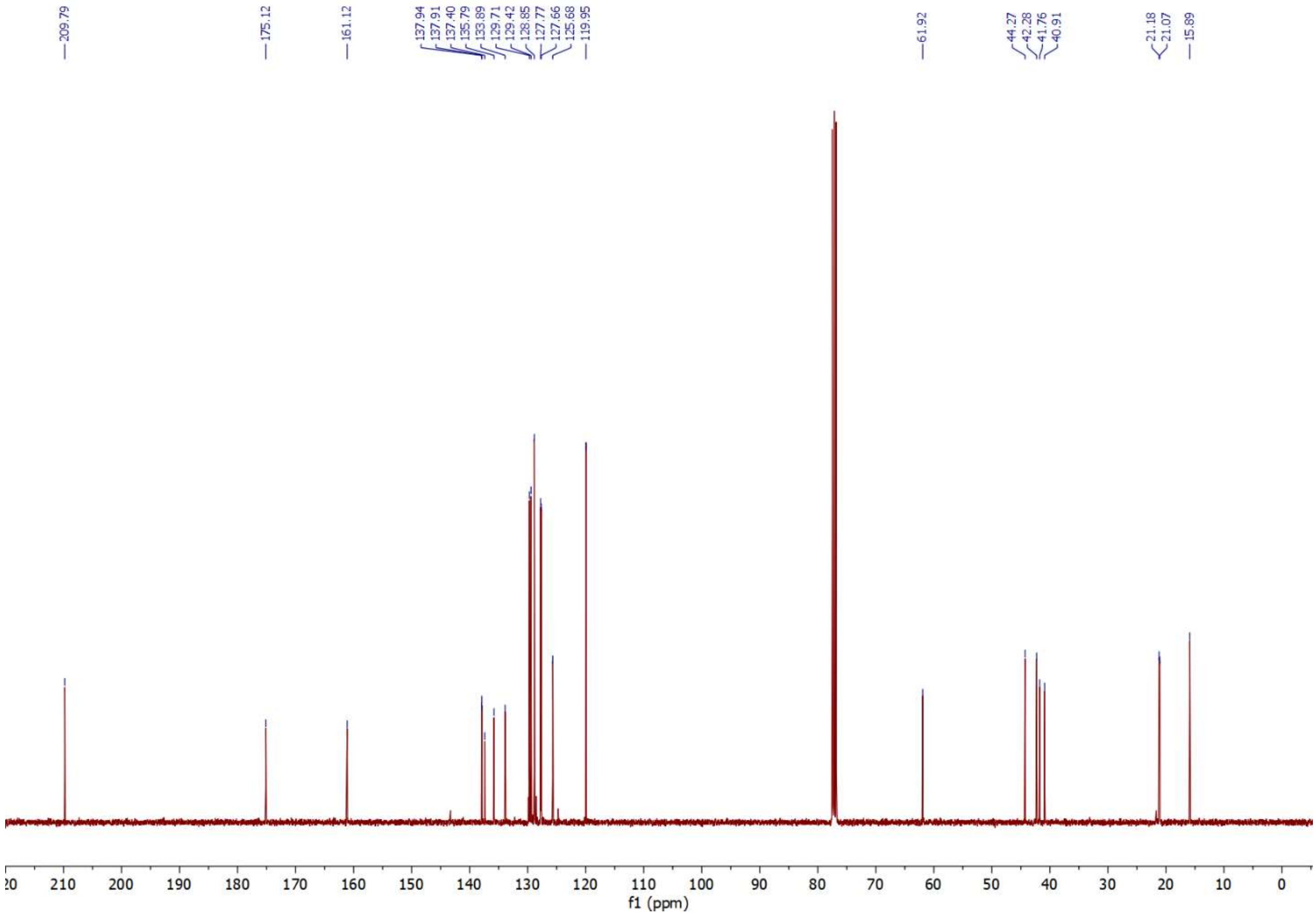

Supplementary Figure S102. The $^{13}C\{^1H\}$ (100.6 MHz) NMR spectrum of 8b.

3´-Methyl-2,6-bis(3-methylphenyl)-1´-phenyl-1*H*-spiro[cyclohexane-1,4´-pyrazole]-4,5´(4*H*)-dione (8c)

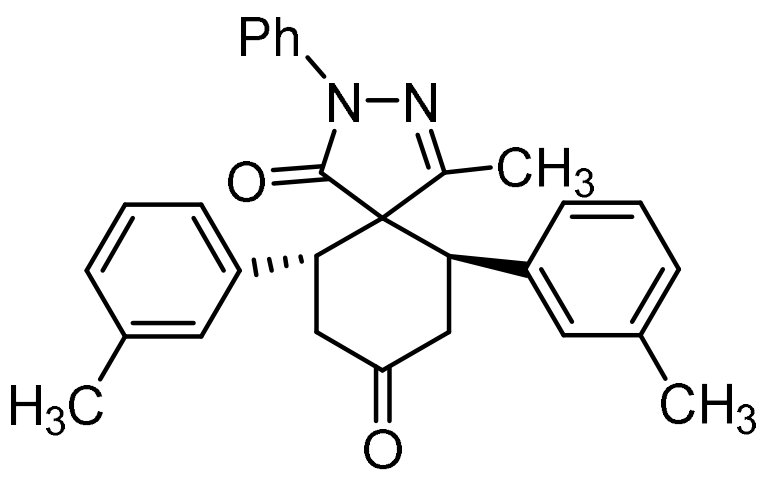

Compound was prepared and isolated by **Procedure I**. Transparent oil. Yield 51% (22 mg), *Dr*. > 20:1. **^{1}H NMR** (25 °C, $CDCl_3$, 400 MHz): δ = 7.54–7.48 (m, 2H), 7.39–7.30 (m, 2H), 7.24–7.14 (m, 2H), 7.13–7.05 (m, 2H), 7.00–6.88 (m, 5H), 3.90–3.80 (m, 2H), 3.63 (dd, $J(^1H\text{-}^1H)$ = 14.3 Hz, $J´(^1H\text{-}^1H)$ = 3.3 Hz, 1H), 3.42 (dd, $J(^1H\text{-}^1H)$ = 16.3 Hz, $J´(^1H\text{-}^1H)$ = 5.4 Hz, 1H), 2.92 (dd, $J(^1H\text{-}^1H)$ = 16.3 Hz, $J´(^1H\text{-}^1H)$ = 8.7 Hz, 1H), 2.63 (dd, $J(^1H\text{-}^1H)$ = 16.1 Hz, $J´(^1H\text{-}^1H)$ = 3.4 Hz, 1H), 2.29 (s, 3H), 2.20 (s, 3H), 1.60 (s, 3H) ppm. **^{13}C NMR** (25 °C, $CDCl_3$, 100.61 MHz): δ = δ 209.7, 175.2, 161.0, 138.8, 138.8, 138.4, 137.4, 136.8, 129.0 (2C), 128.9, 128.9 (2C), 128.8, 128.6, 128.4, 125.7, 125.0, 124.8, 120.0 (2C), 61.8, 44.6, 42.7, 41.7, 40.8, 21.6, 21.5, 15.9 ppm. LRMS (ESI) m/z: $[M+H]^+$calcd for $C_{27}H_{25}N_2O_2$ 437.2; found 437.2.

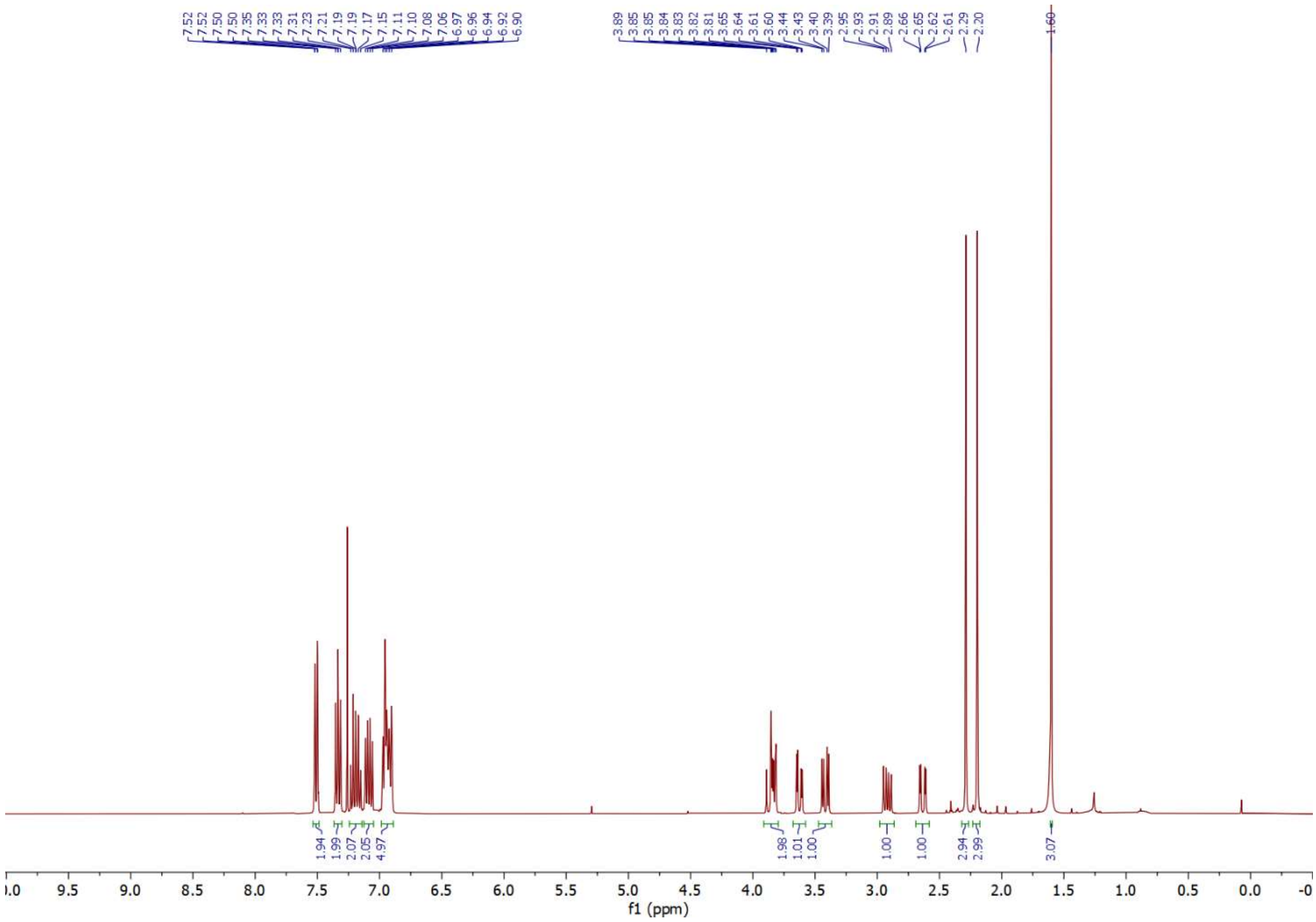

Supplementary Figure S103. The ¹H (400.1 MHz) NMR spectrum of 8c.

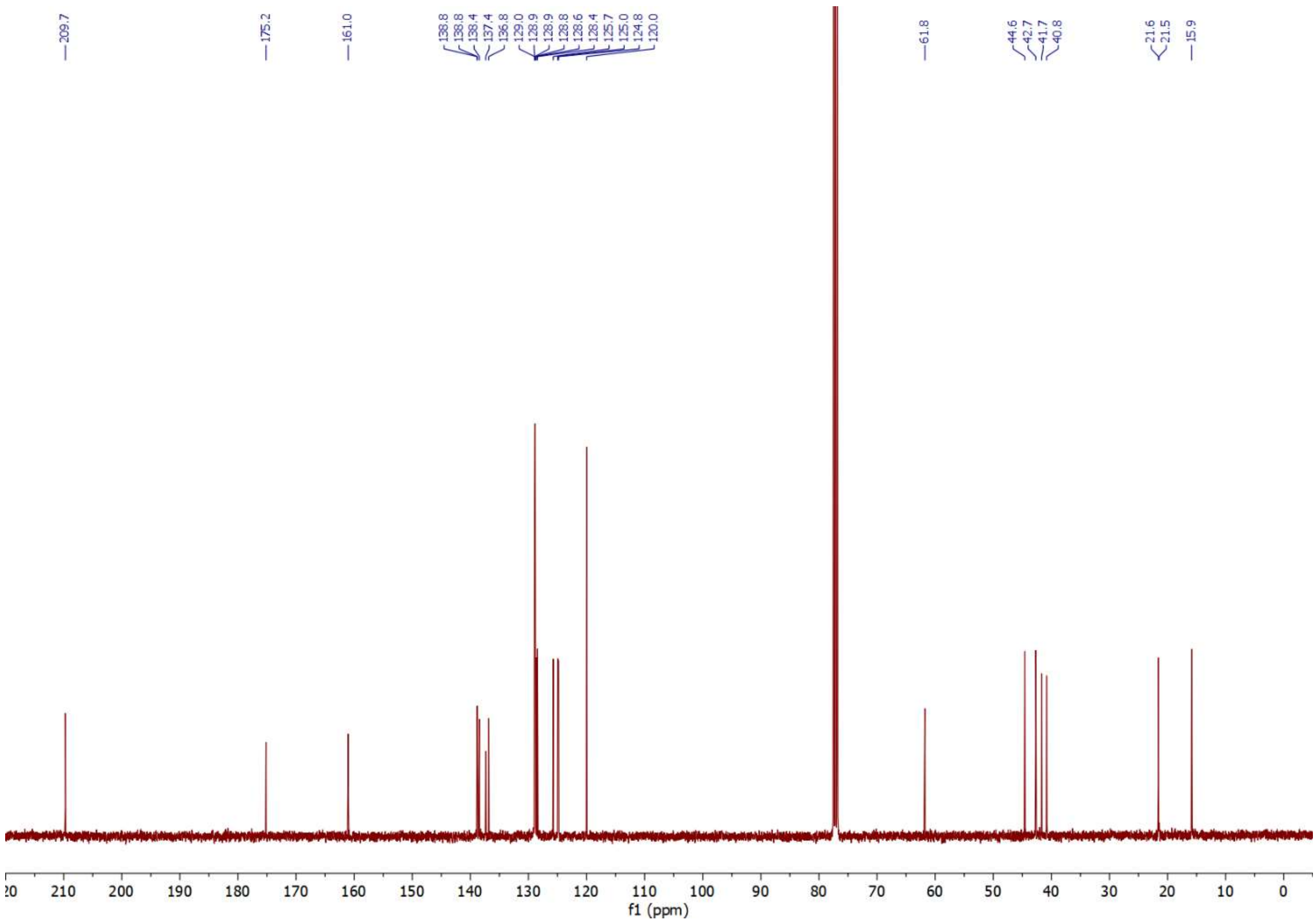

Supplementary Figure S104. The ^{13}C{^{1}H} (100.6 MHz) NMR spectrum of 8c.

2,6-Bis(4-chlorophenyl)-3´-methyl-1´-phenyl-1*H*-spiro[cyclohexane-1,4´-pyrazole]-4,5´(4*H*)-dione (8d)

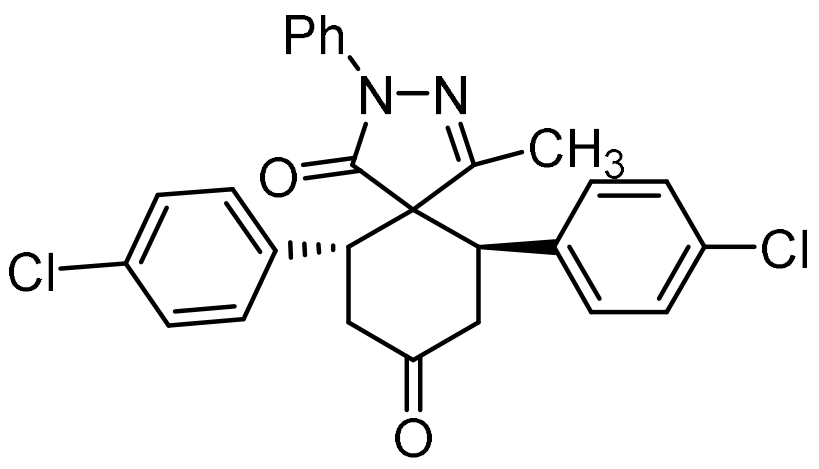

Compound was prepared and isolated by **Procedure I**. Amorphous solid. Yield 61% (29 mg), *Dr*. > 20:1. **^{1}H NMR** (25 °C, $CDCl_3$, 400 MHz): δ = 7.50–7.42 (m, 2H), 7.35–7.32 (m, 2H), 7.31–7.24 (m, 2H), 7.22–7.15 (m, 3H), 7.08–7.03 (m, 4H), 3.91 (dd, $J(^{1}H-^{1}H)$ = 10.2 Hz, $J'(^{1}H-^{1}H)$ = 4.8 Hz, 1H), 3.82 (dd, $J(^{1}H-^{1}H)$ = 16.3 Hz, $J'(^{1}H-^{1}H)$ = 14.4 Hz, 1H), 3.59 (dd, $J(^{1}H-^{1}H)$ = 14.4 Hz, $J'(^{1}H-^{1}H)$ = 3.2 Hz, 1H), 3.29 (dd, $J(^{1}H-^{1}H)$ = 16.5 Hz, $J'(^{1}H-^{1}H)$ = 4.8 Hz, 1H), 2.96 (dd, $J(^{1}H-^{1}H)$ = 16.5 Hz, $J'(^{1}H-^{1}H)$ = 10.2 Hz, 1H), 2.61 (dd, $J(^{1}H-^{1}H)$ = 16.3 Hz, $J'(^{1}H-^{1}H)$ = 3.2 Hz, 1H), 1.75 (s, 3H) ppm. **^{13}C NMR** (25 °C, $CDCl_3$, 100.61 MHz): δ = 208.4, 174.6, 160.0, 137.0, 136.8, 134.9, 134.3, 129.3, 129.2, 129.0, 129.0, 126.1, 119.8, 61.8, 43.5, 41.9, 41.1, 40.5, 15.9 ppm. LRMS (ESI) m/z: $[M+H]^+$calcd for $C_{27}H_{23}Cl_2N_2O_2$ 477.1; found 477.1.

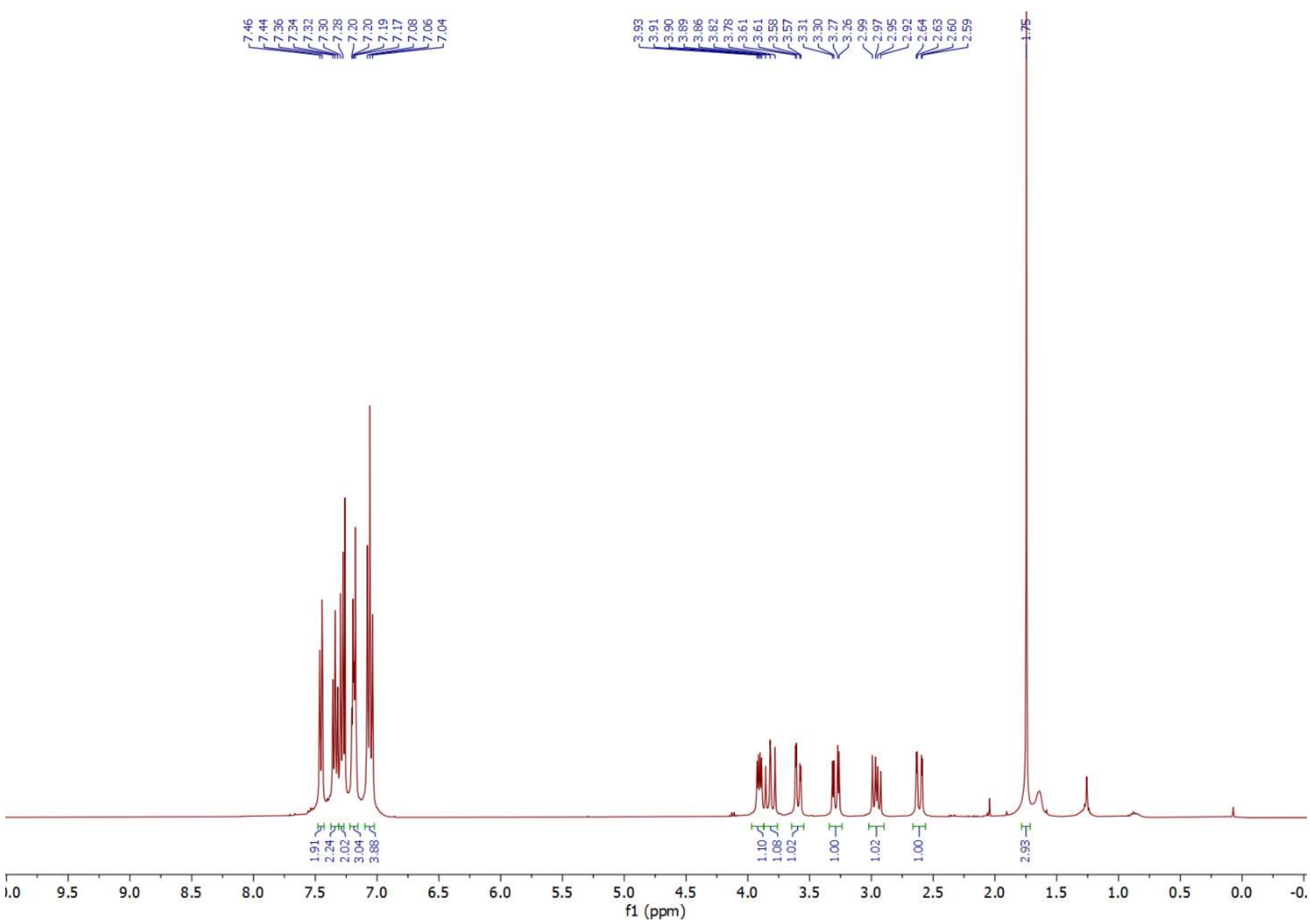

Supplementary Figure S105. The ^{1}H (400.1 MHz) NMR spectrum of 8d.

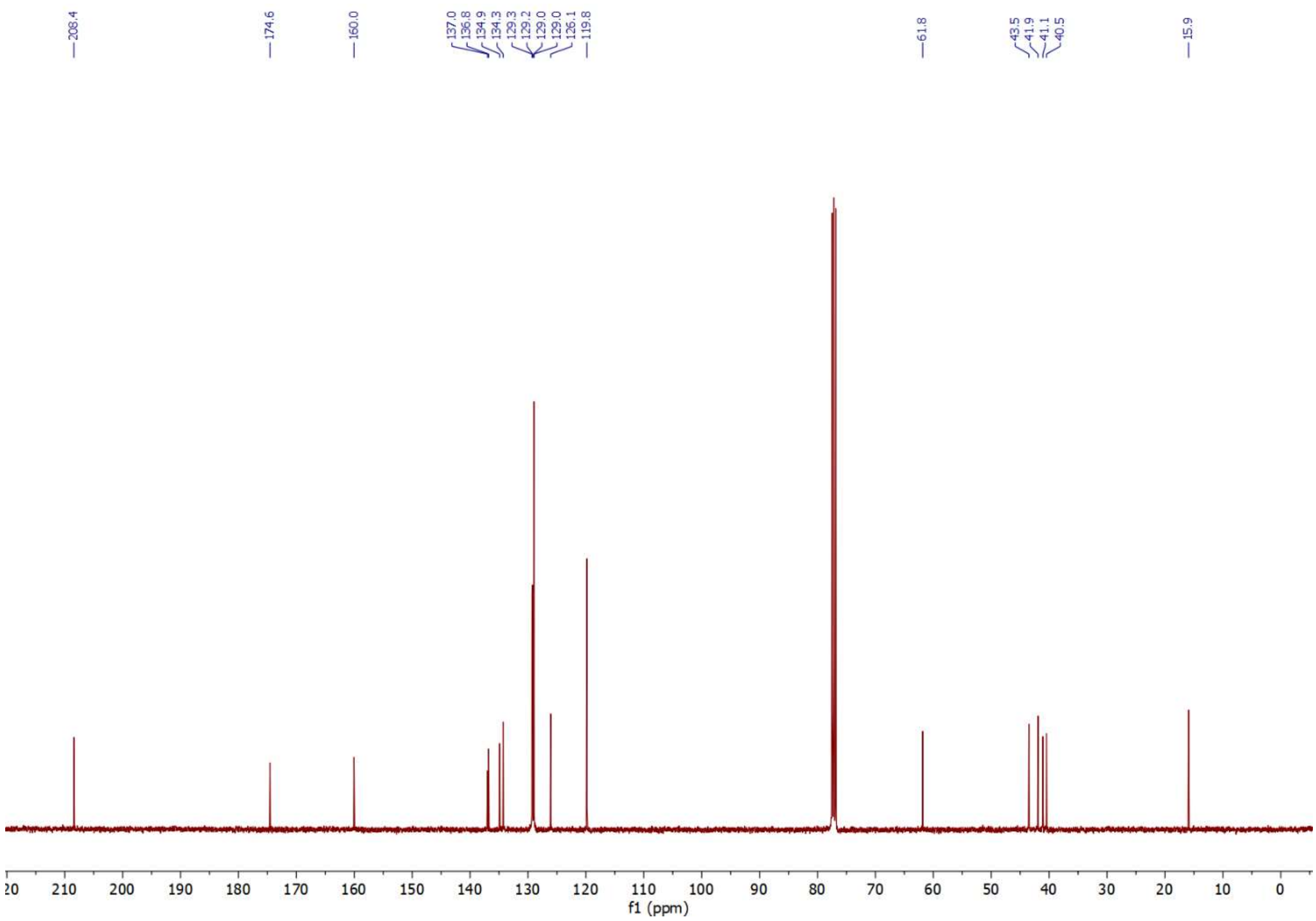

Supplementary Figure S106. The $^{13}C\{^{1}H\}$ (100.6 MHz) NMR spectrum of 8d.

2,6-Bis(2-thienyl)-3´-methyl-1´-phenyl-1*H*-spiro[cyclohexane-1,4´-pyrazole]-4,5´(4*H*)-dione (8e)

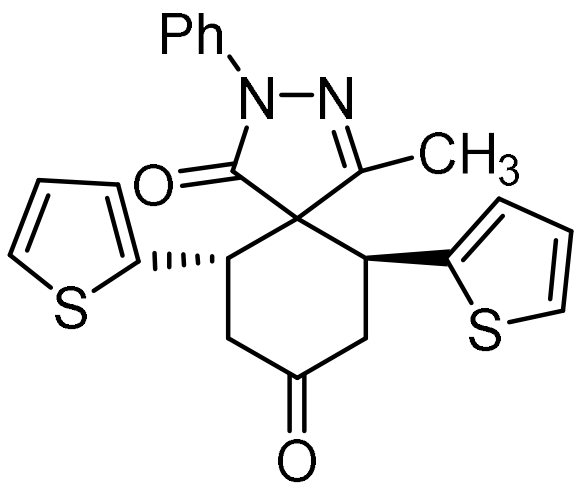

Compound was prepared and isolated by **Procedure I**. Amorphous solid. Yield 67% (28 mg), *Dr*. > 20:1. **^{1}H NMR** (25 °C, $CDCl_3$, 400 MHz): δ = 7.73–7.64 (m, 2H), 7.44–7.34 (m, 2H), 7.26–7.23 (m, 1H), 7.22–7.17 (m, 1H), 7.09 (dd, $J(^1H\text{-}^1H)$ = 5.0 Hz, $J´(^1H\text{-}^1H)$ = 1.2 Hz, 1H), 6.99 (dd, $J(^1H\text{-}^1H)$ = 5.1 Hz, $J´(^1H\text{-}^1H)$ = 3.5 Hz, 1H), 6.89–6.85 (m, 2H), 6.84 (dd, $J(^1H\text{-}^1H)$ = 5.1 Hz, $J´(^1H\text{-}^1H)$ = 3.5 Hz, 1H), 4.14 (dd, $J(^1H\text{-}^1H)$ = 13.9 Hz, $J´(^1H\text{-}^1H)$ = 3.9 Hz, 1H), 4.09 (t, $J(^1H\text{-}^1H)$ = 6.2 Hz, 1H), 3.82 (dd, $J(^1H\text{-}^1H)$ = 15.7 Hz, $J´(^1H\text{-}^1H)$ = 13.9 Hz, 1H), 3.67 (dd, $J(^1H\text{-}^1H)$ = 15.7 Hz, $J´(^1H\text{-}^1H)$ = 5.8 Hz, 1H), 2.86–2.69 (m, 2H), 1.67 (s, 3H) ppm. **^{13}C NMR** (25 °C, $CDCl_3$, 100.61 MHz): δ = 207.3, 174.0, 160.9, 141.6, 139.8, 137.5, 129.0 (2C), 127.4, 127.1, 126.2, 125.8, 125.7, 125.4, 125.0, 119.7 (2C), 61.3, 43.5, 42.1, 41.1, 38.6, 15.5 ppm. LRMS (ESI) m/z: calcd for $C_{23}H_{20}N_2O_2S_2Na$ $[M+Na]^+$ 443.1; found 443.1.

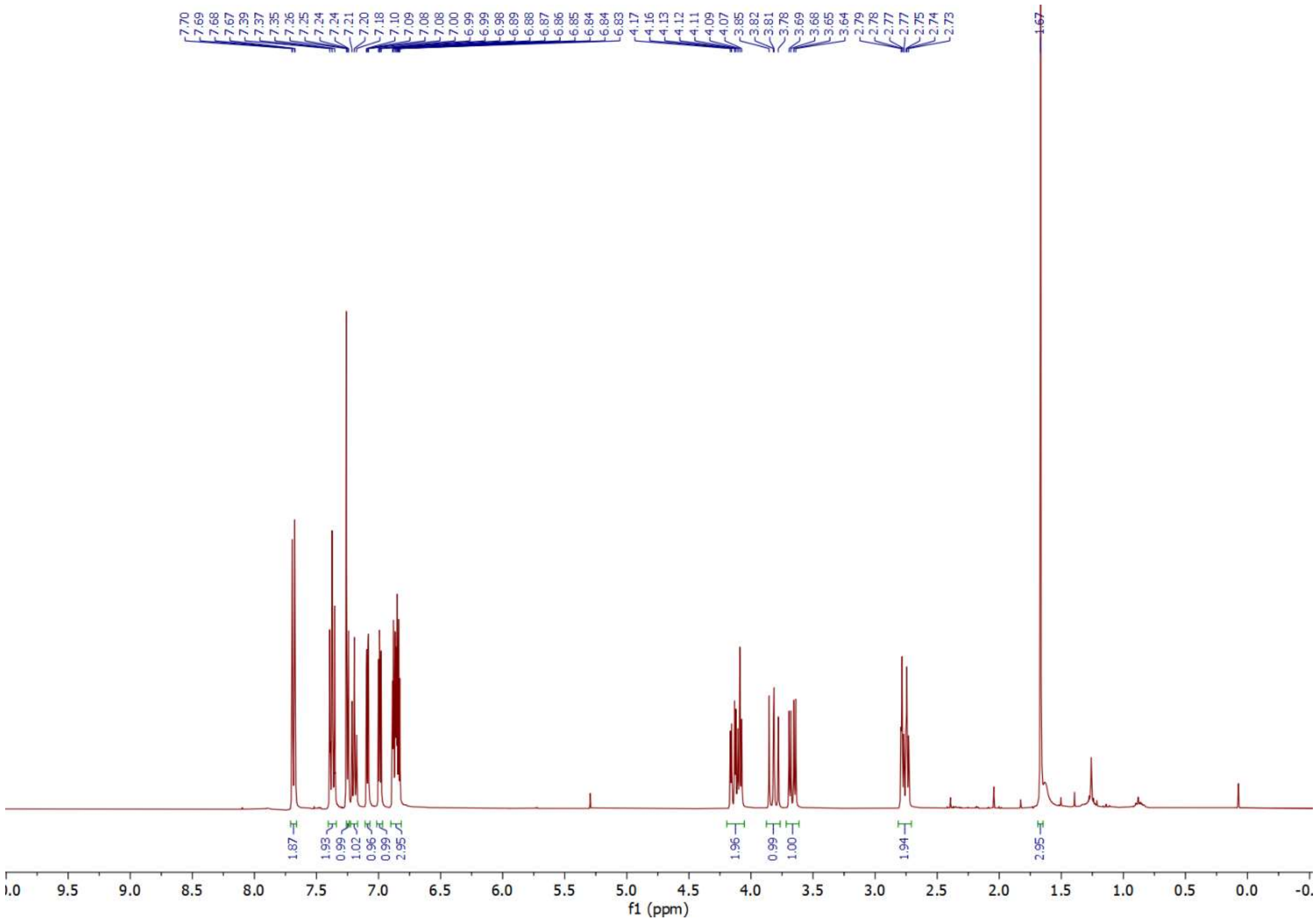

Supplementary Figure S107. The ^{1}H (400.1 MHz) NMR spectrum of 8e.

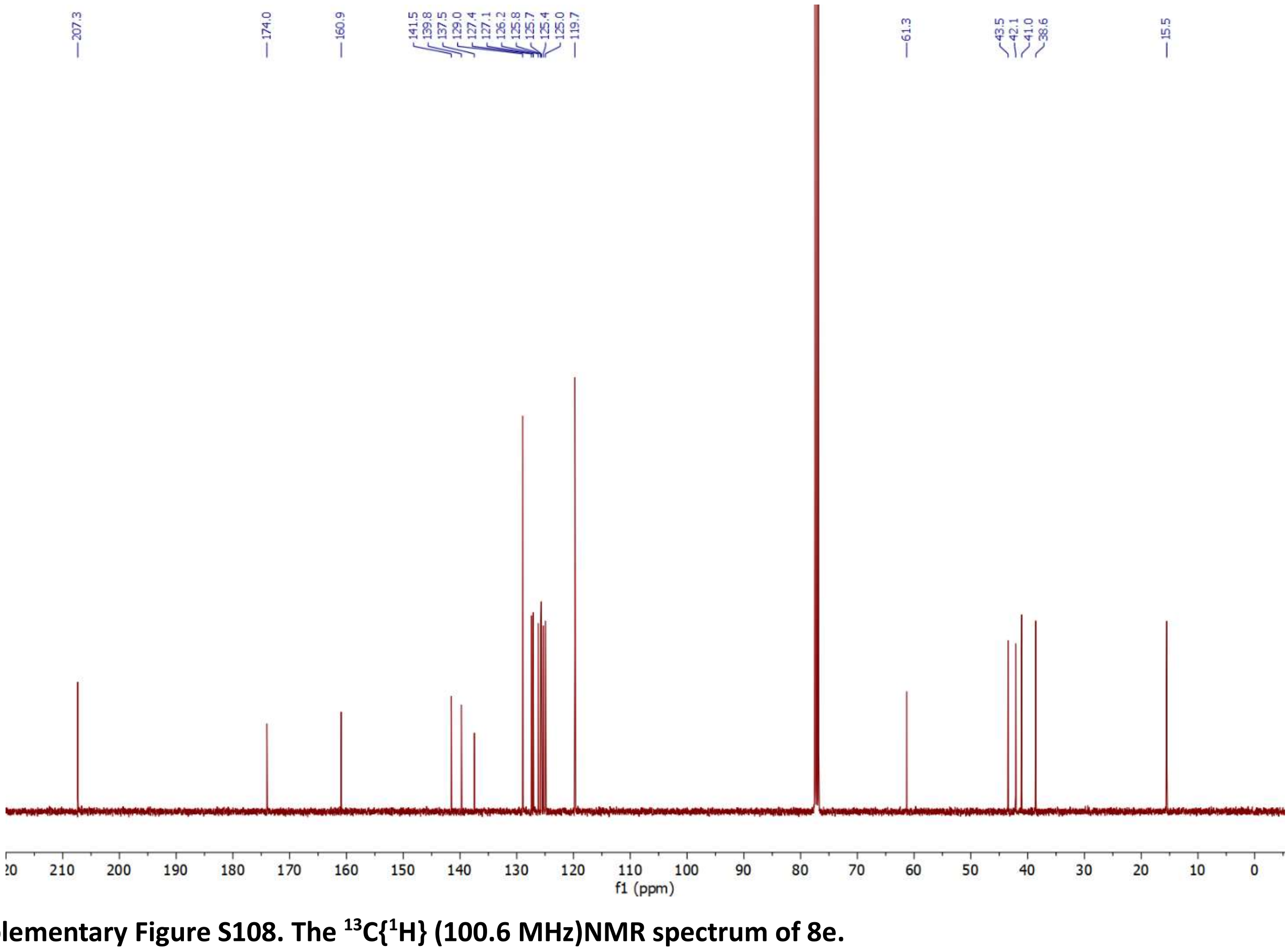

Supplementary Figure S108. The $^{13}C\{^{1}H\}$ (100.6 MHz)NMR spectrum of 8e.

2,6-Bis(phenyl)-3´-methyl-1´-(4-bromophenyl)-1*H*-spiro[cyclohexane-1,4´-pyrazole]-4,5´(4H)-dione (8f)

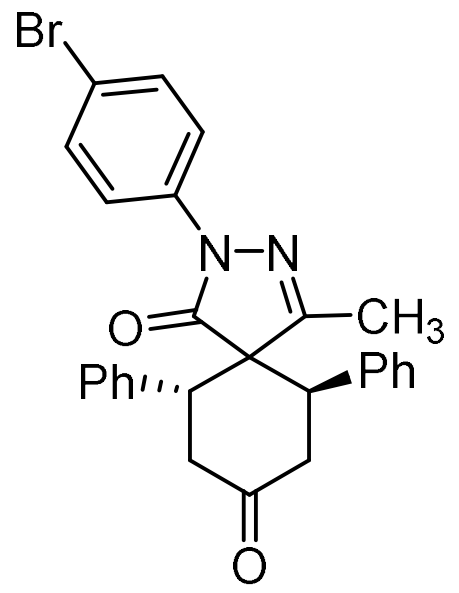

Compound was prepared and isolated by **Procedure I**. Amorphous solid. Yield 51% (25 mg), *Dr*. > 20:1. **^{1}H NMR** (25 °C, $CDCl_3$, 400 MHz): δ = 7.42 (s, 4H), 7.35–7.26 (m, 3H), 7.24–7.13 (m, 3H), 7.12–7.09 (m, 4H), 3.98–3.77 (m, 2H), 3.65 (dd, $J(^1H\text{-}^1H)$ = 14.3 Hz, $J(^1H\text{-}^1H)$ = 3.2 Hz, 1H), 3.35 (dd, $J(^1H\text{-}^1H)$ = 16.4 Hz, $J´(^1H\text{-}^1H)$ = 5.1 Hz, 1H), 2.98 (dd, $J(^1H\text{-}^1H)$ = 16.5 Hz, $J´(^1H\text{-}^1H)$ = 9.6 Hz, 1H), 2.66 (dd, $J(^1H\text{-}^1H)$ = 16.2 Hz, $J´(^1H\text{-}^1H)$ = 3.1 Hz, 1H), 1.68 (s, 3H) ppm. **^{13}C NMR** (25 °C, $CDCl_3$, 100.61 MHz): δ = 209.2, 174.9, 161.0, 138.5, 136.5, 136.3, 131.9, 129.1, 128.8, 128.3, 128.3, 127.8, 127.7, 121.0, 118.6, 62.2, 44.2, 42.6, 41.3, 40.6, 15.8 ppm. IR (ATR) ν_{max} = 3033, 2924, 2852, 1714, 1691, 1589, 1487, 1367, 1325, 1074 cm^{-1}. HRMS (ESI) m/z: calcd for $C_{27}H_{24}BrN_2O_2$ [M+H]$^+$ 487.1016; found 487.1011.

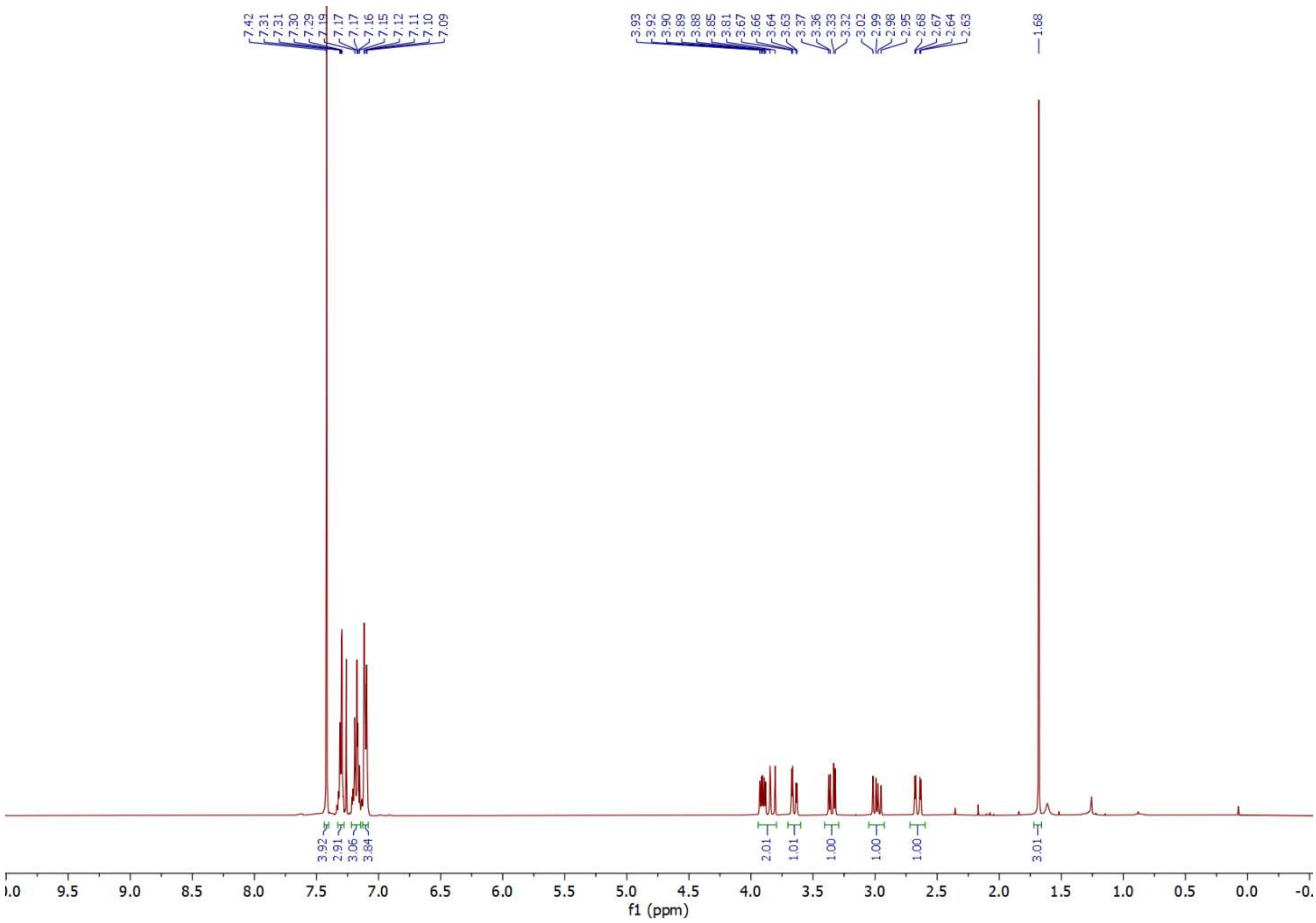

Supplementary Figure S109. The ^{1}H (400.1 MHz) NMR spectrum of 8f.

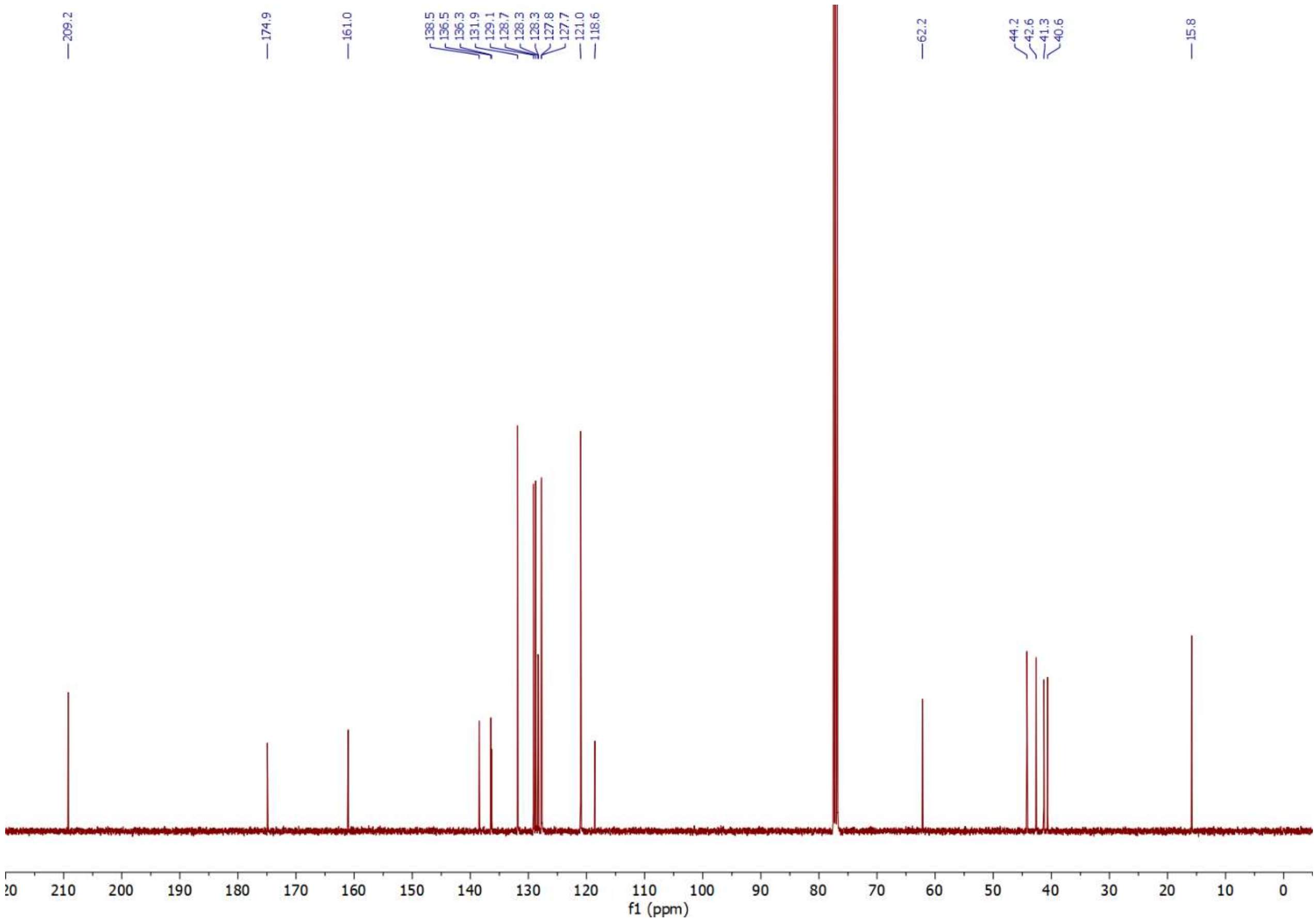

Supplementary Figure S110. The ^{13}C{^{1}H} (100.6 MHz) NMR spectrum of 8f.

2,6-bis(4-methylphenyl)-1´,3´-bisphenyl-1*H*-spiro[cyclohexane-1,4´-pyrazole]-4,5´(4*H*)-dione (8g)

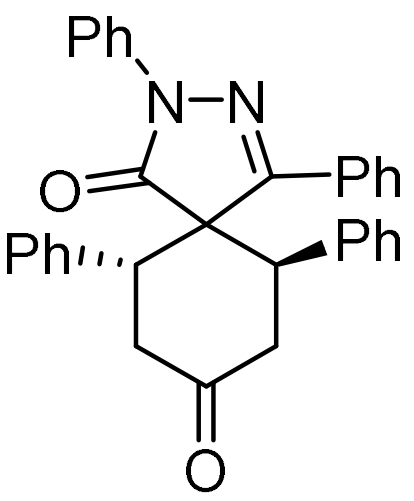

Compound was prepared and isolated by **Procedure I**. Amorphous solid. Yield 73% (34 mg), *Dr*. > 20:1. Mp 130 -132°C. ^{1}**H NMR** (25 °C, $CDCl_3$, 400 MHz): δ = 7.63–7.55 (m, 2H), 7.48–7.39 (m, 3H), 7.38–7.28 (m, 5H), 7.22–7.02 (m, 8H), 7.02–6.94 (m, 2H), 6.81–6.71 (m, 2H), 4.27 (ddd, *J*(^{1}H-^{1}H) = 14.1 Hz, *J*´(^{1}H-^{1}H) = 8.6 Hz, *J*´´(^{1}H-^{1}H) = 3.3 Hz, 1H), 4.05 (dd, *J*(^{1}H-^{1}H) = 16.9 Hz, *J*´(^{1}H-^{1}H) = 14.5 Hz, 1H), 3.44 (dd, *J*(^{1}H-^{1}H) = 17.5 Hz, ´*J*(^{1}H-^{1}H) = 14.1 Hz, 1H), 3.04 (dd, *J*(^{1}H-^{1}H) = 17.5 Hz, *J*´(^{1}H-^{1}H) = 4.0 Hz, 1H), 2.68 (dd, *J*(^{1}H-^{1}H) = 16.9 Hz, *J*´(^{1}H-^{1}H) = 2.6 Hz, 1H) ppm. ^{13}**C NMR** (25 °C, $CDCl_3$, 100.61 MHz): δ = 209.8, 175.6, 159.0, 137.3, 137.1, 135.8, 132.6, 130.2, 128.8 (2C), 128.7 (2C), 128.4 (4C), 128.2, 128.1 (2C), 127.6, 127.2 (4C), 126.1, 120.3 (2C), 63.6, 43.2, 43.2, 41.0, 40.0 ppm. IR (ATR) ν_{max} = 3059, 3032, 2954, 2920, 2897, 1718, 1695, 1597, 1498, 1406 cm^{-1}; HRMS (ESI) m/z: $[M+H]^+$calcd for $C_{32}H_{27}N_2O_2$ 471.2067; found 471.2063. Compound **8g** was also isolated in single-crystalline form. The material was dissolved in DCM, and crystallization was initiated by the addition of i-PrOH at room temperature.

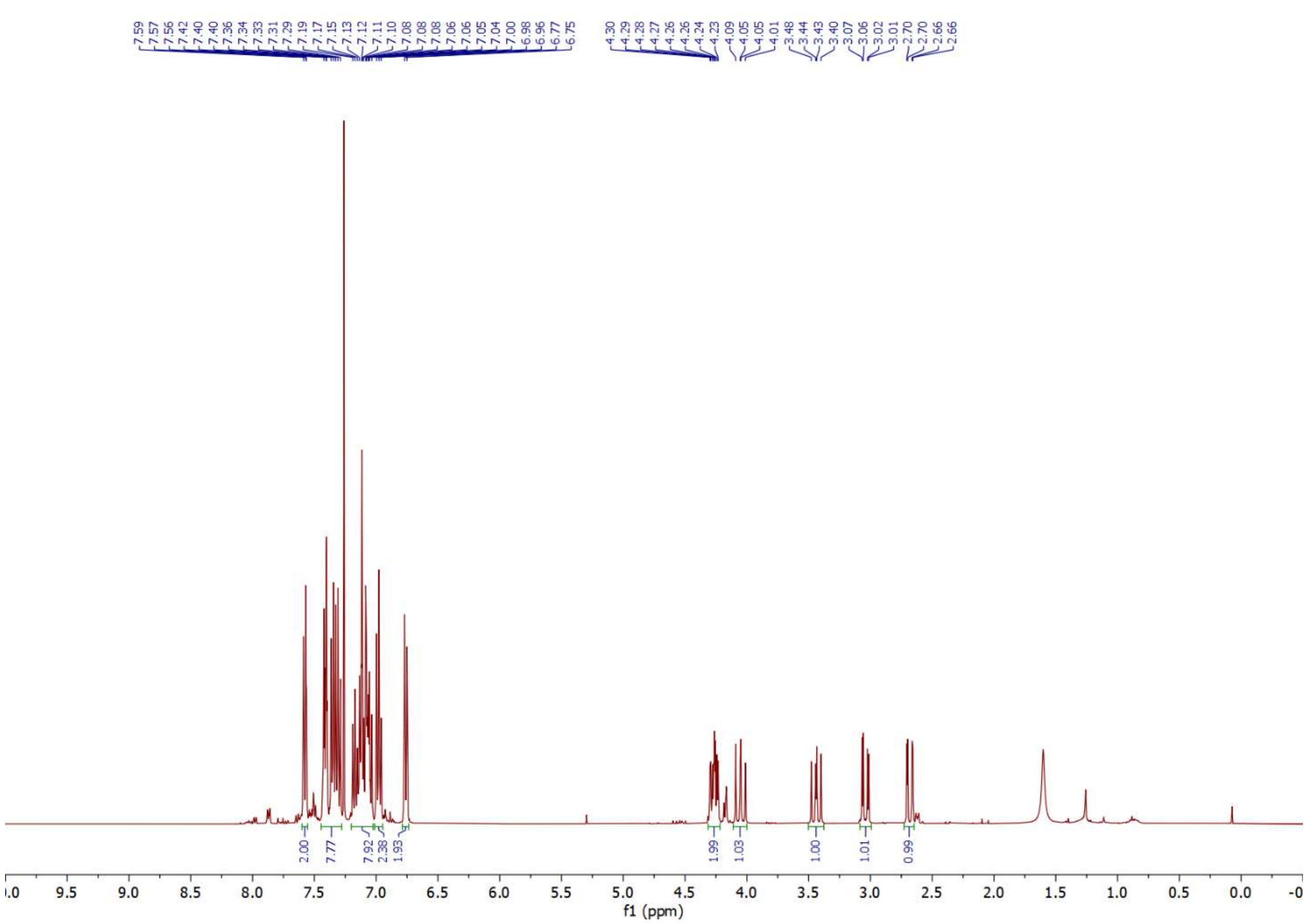

Supplementary Figure S111. The ^{1}H (400.1 MHz) NMR spectrum of 8g.

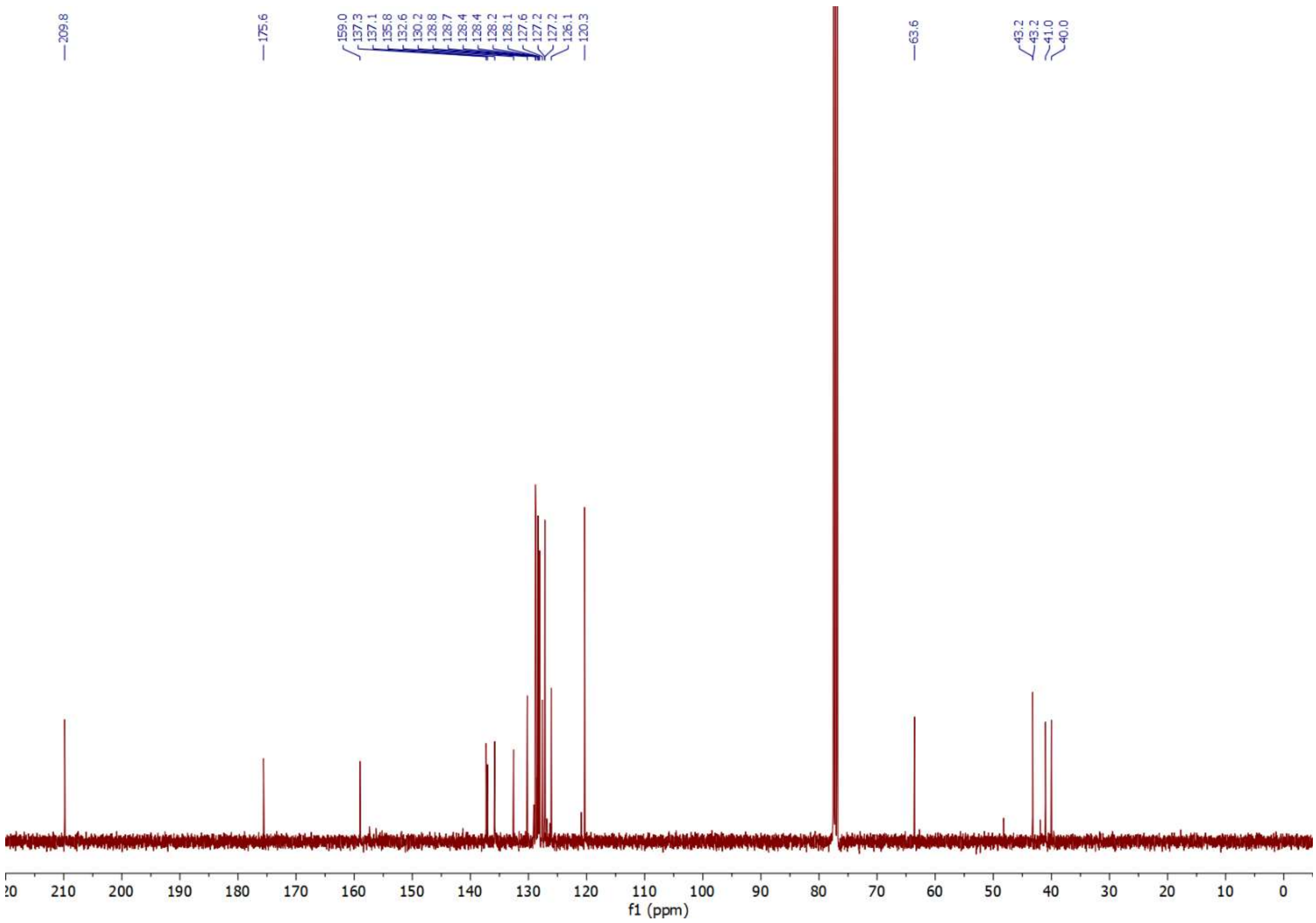

Supplementary Figure S112. The $^{13}C\{^{1}H\}$ (100.6 MHz) NMR spectrum of 8g.

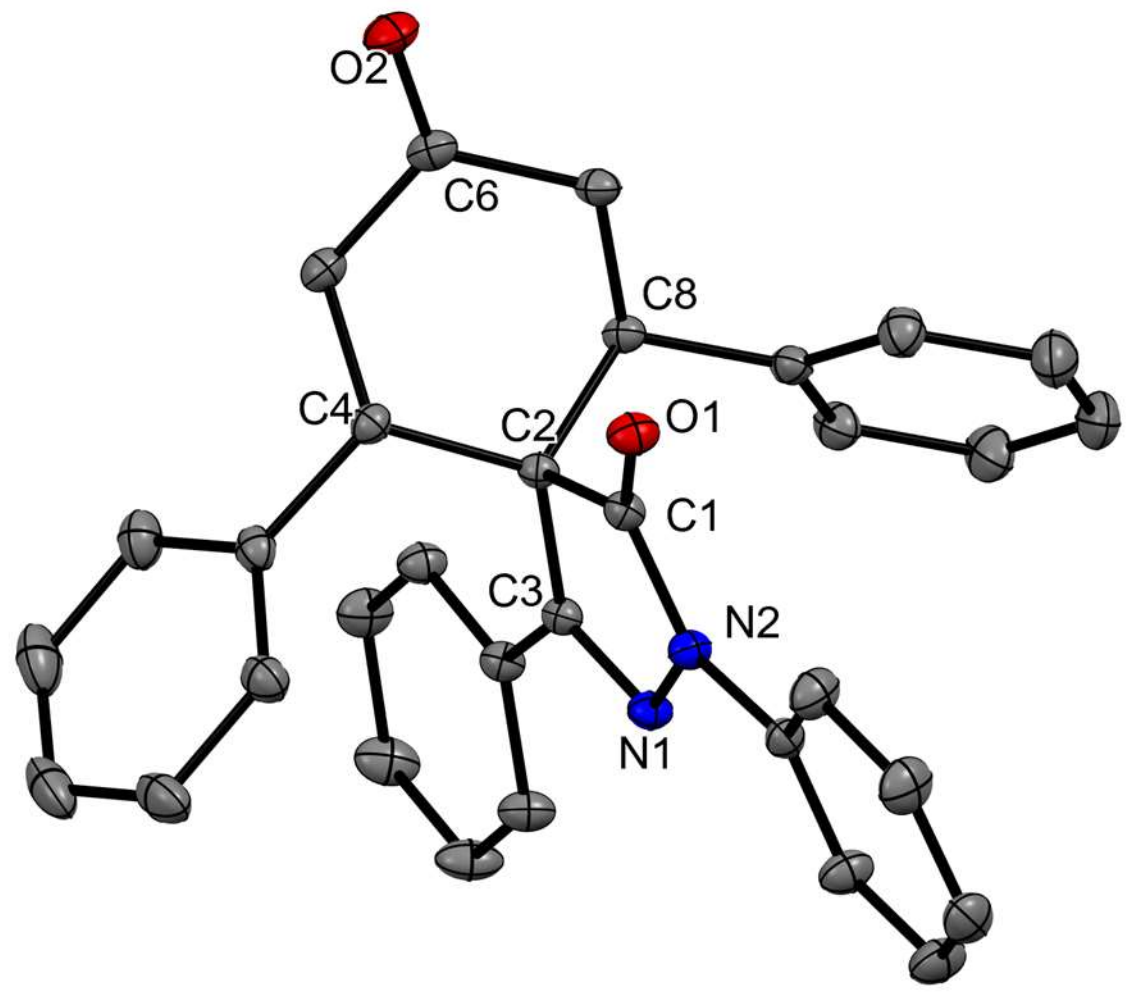

Supplementary Figure S113. The molecular structure of 8g. Only one of two molecules in the unit cell is displayed. ORTEP view, 30% probability level. Selected interatomic distances [Å]: C1-C2 1.5352(13), C2-C3 1.5187(12), C2-C4 1.5830(13), C2-C8 1.5764(13), C1-O1 1.2180(12), C6-O2 1.2155(13). Selected angles[°]: C3-C2-C1 99.73(7), C4-C2-C8 110.81(7).

Supplementary Table S17. Experimental details of **8g**.

Crystal data	
Chemical formula	$C_{32}H_{26}N_2O_2$
M_r	470.55
Crystal system, space group	Triclinic, *P*-1
Temperature (K)	150
a, *b*, *c* (Å)	8.8175(2), 14.2911(3), 19.6205(5)
α, β, γ (°)	79.650(1), 89.112(1), 89.999(1)
V (Å^3)	2431.88(10)
Z	4
Radiation type	Mo*K*α
μ (mm^{-1})	0.08
Crystal size (mm)	0.58 × 0.40 × 0.18
Data collection	
Diffractometer	Bruker D8 - Venture
Absorption correction	Multi-scan<br>*SADABS2016*/2 - Bruker AXS area detector scaling and absorption correction Reference: Krause, L., Herbst-Irmer, R., Sheldrick G.M. & Stalke D., *J. Appl. Cryst.* 2015, 48, 3–10.
T_{min}, T_{max}	0.728, 0.746
No. of measured, independent and observed [$I > 2\sigma(I)$] reflections	149247, 11972, 10653
R_{int}	0.042
$(\sin \theta/\lambda)_{max}$ (Å^{-1})	0.667
Refinement	
$R[F^2 > 2\sigma(F^2)]$, $wR(F^2)$, S	0.040, 0.105, 1.02
No. of reflections	11972
No. of parameters	649
No. of restraints	601
H-atom treatment	H-atom parameters constrained
$\Delta\rho_{max}$, $\Delta\rho_{min}$ (e Å^{-3})	0.31, −0.17

Dimethyl 4,4'-(1-methyl-4,8-dioxo-3-phenyl-2,3-diazaspiro[4.5]dec-1-ene-6,10-diyl)dibenzoate (8h)

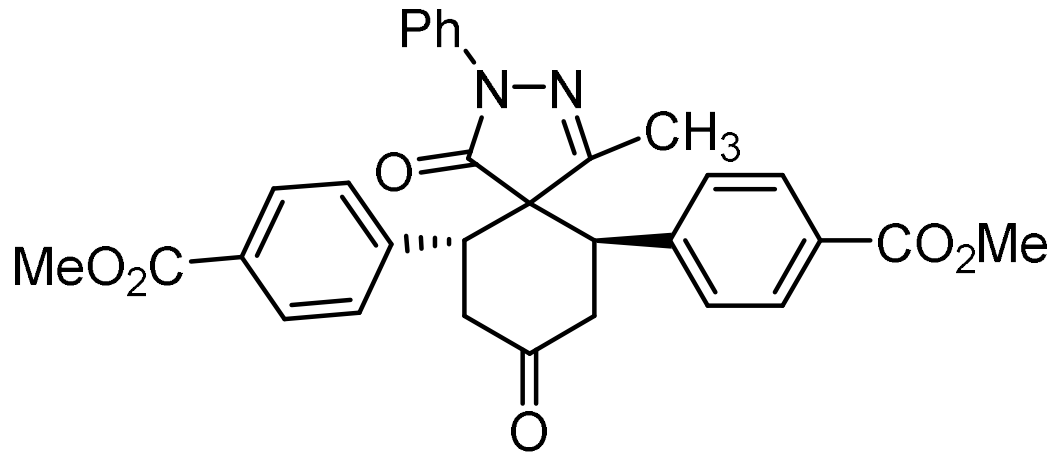

Compound was prepared according to the standard **Procedure I** after 60 h, as white semi-solid (26 mg, 50%). **^{1}H NMR** (25 °C, 400 MHz, $CDCl_3$) δ = 7.98 (d, J(^{1}H-^{1}H) = 8.4 Hz, 2H), 7.88 (d, J(^{1}H-^{1}H) = 8.4 Hz, 2H), 7.47 – 7.42 (m, 2H), 7.35 – 7.29 (m, 2H), 7.24 – 7.14 (m, 5H), 4.00 (dd, J(^{1}H-^{1}H) = 10.1 Hz, J'(^{1}H-^{1}H) = 4.8 Hz, 1H), 3.94 – 3.90 (m, 1H), 3.91 (s, 3H), 3.84 (s, 3H), 3.70 (dd, J(^{1}H-^{1}H) = 14.3 Hz, J'(^{1}H-^{1}H) = 3.1 Hz, 1H), 3.35 (dd, J(^{1}H-^{1}H) = 16.5 Hz, J'(^{1}H-^{1}H) = 4.8 Hz, 1H), 3.02 (dd, J(^{1}H-^{1}H) = 16.5 Hz, J'(^{1}H-^{1}H) = 10.1 Hz, 1H), 2.66 (dd, J(^{1}H-^{1}H) = 16.3 Hz, J'(^{1}H-^{1}H) = 3.1 Hz, 1H), 1.72 (s, 3H) ppm. **^{13}C NMR** (25 °C, 101 MHz, $CDCl_3$) δ = 208.1, 174.4, 166.5, 159.8, 143.3, 142.4, 141.3, 136.9, 130.4 (2C), 130.2 (2C), 130.1 (2C), 129.0 (2C), 128.0 (2C), 127.8 (2C), 126.0, 119.7 (2C), 61.7, 52.4, 52.3, 44.1, 42.5, 40.9, 40.3, 15.9 ppm; FT-IR (ATR): v= 2952, 2844, 1716, 1277, 1111 cm^{-1}; $C_{31}H_{29}N_2O_6$ $[M+H]^+$ 525.2020, found 525.2028.

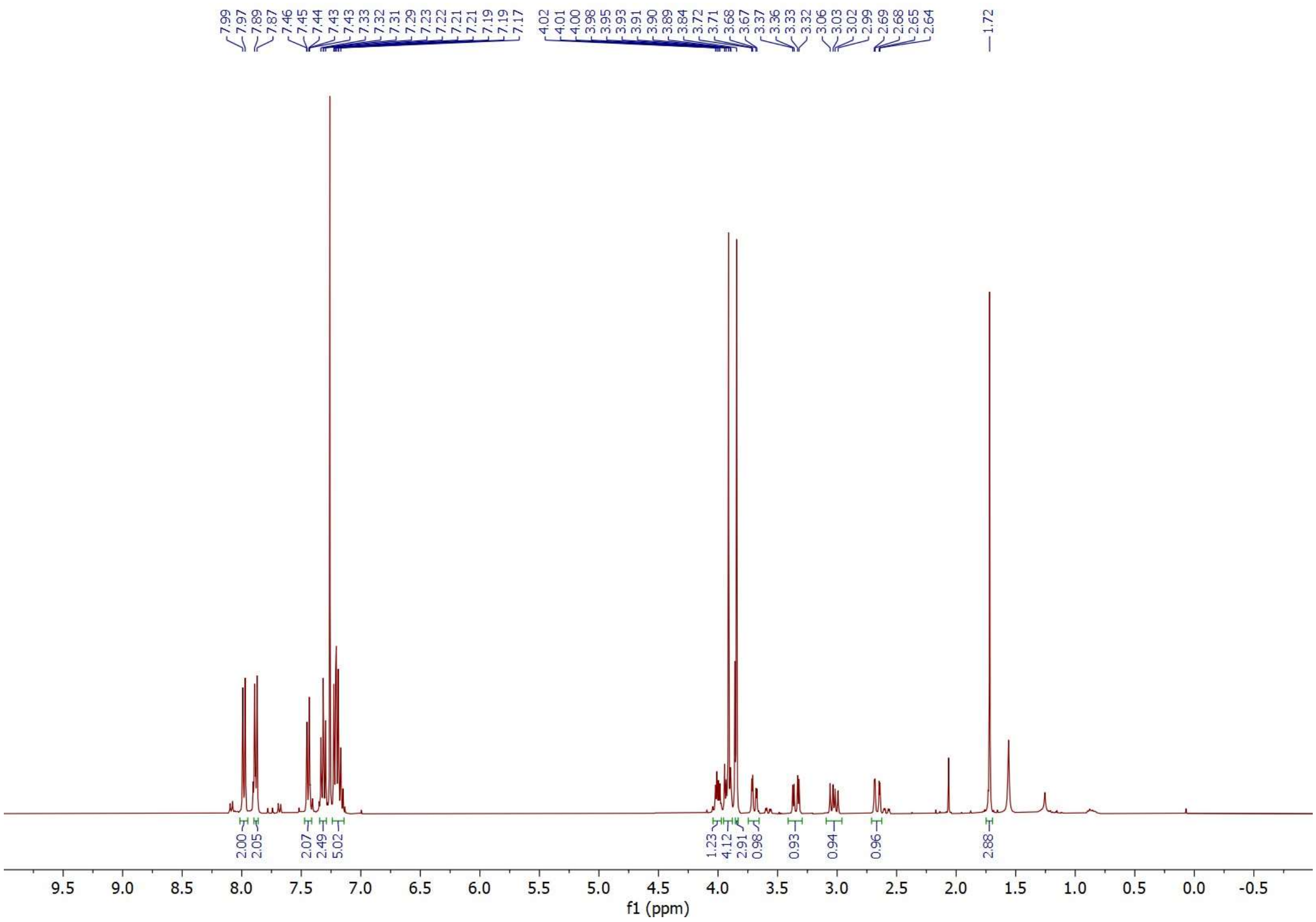

Supplementary Figure S114. The ^{1}H (400.1 MHz) NMR spectrum of 8h.

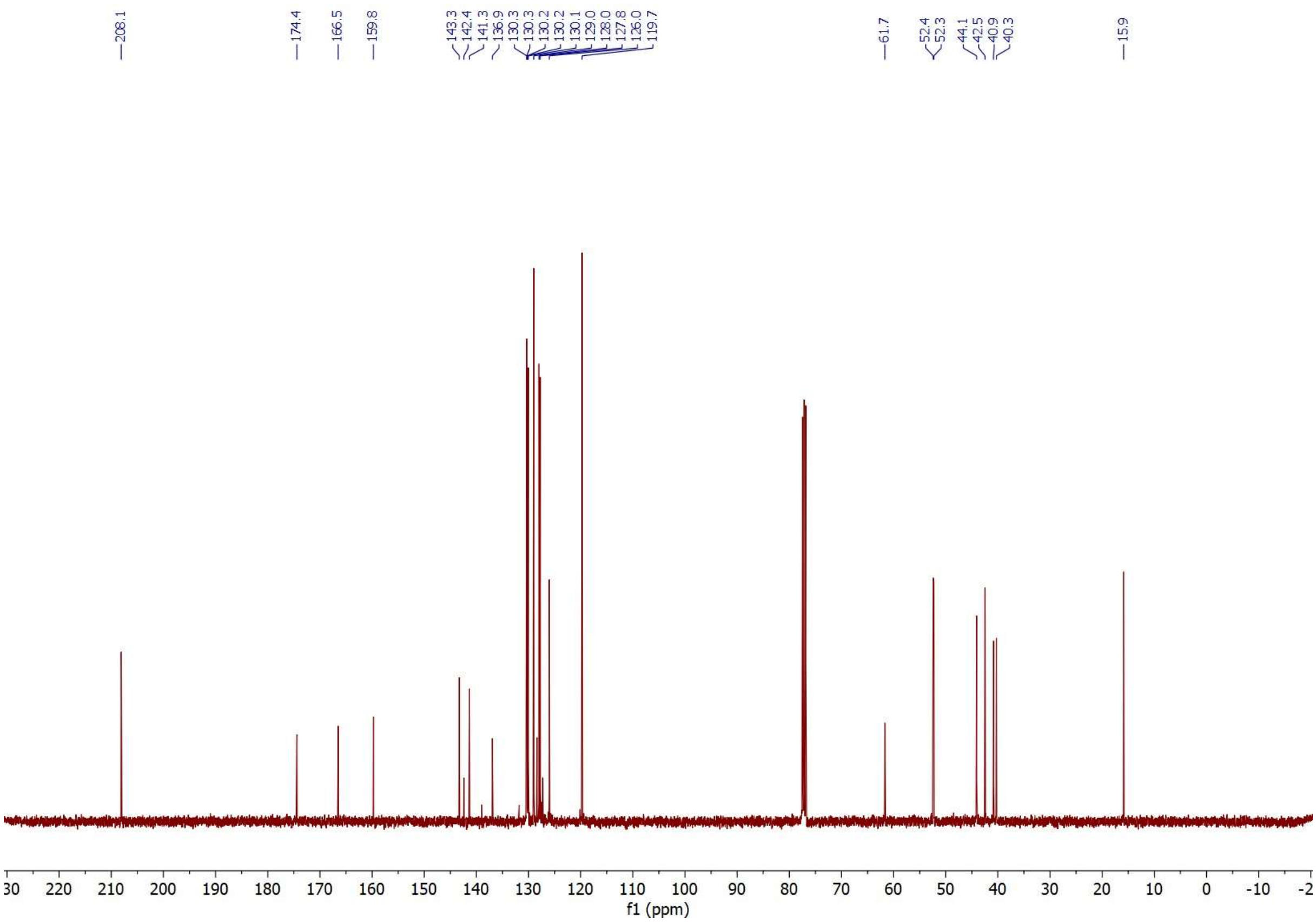

Supplementary Figure S115. The ^{13}C (100.6 MHz) NMR spectrum of 8h.

Supplementary References

S1. G.M. Sheldrick, *Acta Cryst.* **2015**, *A71*, 3–8.

S2. Dianati, V. et al. *J. Med. Chem*. **2018**, *61*, 8457-8467.

S3. Martínez-Aguirre, M. A.et al. *J. Org. Chem*. **2025**, *90*, 14232-14245.

S4. Rosh, L. E. et al. *Organometallics* **2022**, *41*, 2301–2316.

S5. Gao, F., Huang, Y. *Adv. Synth. Catal*. **2014**, *356*, 2422–2428.

S6. Masuda, T.; Jitoe, A.; Isobe, J.; Nakatani, N.; Yonemori, S. *Phytochemistry*, **1993**, *32*, 1557-1560.

S7. Kashyap, A., Bokosike, T. K., Bhuvanesh, N., Pattabiraman, M. *Org. Biomol. Chem*. **2018**, *16*, 6870–6875.

S8. Miao, C.-B.; Chen, M.-L.; Sun, X.-Q.; Yang, H.-T. *Synthesis* **2019**, *51*, 2945–2958.

S9. Bera, M.; Ghosh, T. K.; Akhuli, B.; Ghosh, P. *J. Mol. Catal. A Chem.* **2015**, *408*, 287–295.

S10. Seidalimu, I.; Fang, X.; Lv, W.; Yang, X.; He, X.; Zhang, J.; Wu, F. *Adv. Synth. Catal.* **2013**, *355*, 857–863.

S11. Zenz, I.; Mayr, H. *J. Org. Chem.* **2011**, *76*, 9370–9378.

S12. Xie, Z.-B. et al. *Beilstein J. Org. Chem.* **2012**, *8*, 534–538.

S13. Jao, T.-J.; Akula, P. S.; Hong, B.-C.; Lee, G.-H. *Org. Lett.* **2020**, *22*, 62–67.

S14. Tilly, D. P.; McColl, C.; Hu, M.; Vitórica-Yrezábal, I.; Webb, S. J. *Org. Biomol. Chem.* **2023**, *21*, 9562–9571.

S15. www.chemicalbook.com (Accessed 25.04.2025)

S16. www.pubchem.ncbi.nlm.nih.gov (Accessed 25.04.2025)

S17. Ballatore, C. et al. *J. Med. Chem.* **2011**, *54*, 6969–6983.

S18. www.organicchemistrydata.org (Accessed 25.04.2025)

S19. Wanner, D. M. et al. *Angew. Chem. Int. Ed*. **2023**, *62*, e202307317.

S20. Wu, B. et al. *Eur. J. Org. Chem*. **2012**, 1318–1327.
