# Supplementary material for: Uncover the strength of polyhedral dicarbaboranes as the next generation of superbases in organocatalysis
Source: Nat Commun. 2026 Aug 26;17:10198. doi: 10.1038/s41467-026-77070-6 (PMC13612482; doi:10.1038/s41467-026-77070-6)
Supplement: Supplementary file 2 [file 41467_2026_77070_MOESM2_ESM.pdf]

nature portfolio

Peer Review File

Uncover the Strength of Polyhedral Dicarbaboranes as The Next Generation of Superbases in Organocatalysis

Corresponding Author: Professor Ales Ruzicka

This file contains all reviewer reports in order by version, followed by all author rebuttals in order by version.

Version 0:

Reviewer comments:

Reviewer #1

(Remarks to the Author)
In this contribution, Ruzicka and Vesely report on the high basicity of the previously described bis-carbene–carborane adduct o-2, which, according to the authors, exceeds the intrinsic basicity of the parent carbene. As expected, various C–H acidic hydrocarbons with pK_a values (in DMSO) ranging from 10 to 14 were fully deprotonated, and the corresponding salts were isolated in good yields. These compounds were comprehensively characterized by NMR spectroscopy and single-crystal X-ray diffraction.

It would be beneficial if the authors provided the exact pK_a values of all acids employed in the study (see Scheme 2). These values are readily accessible from the Bordwell pK_a database, which is publicly available online.

In addition, the applicability of o-2 as an organo-superbase catalyst was demonstrated in several standard base-catalyzed transformations, including the aldol condensation of butyraldehyde and Michael additions of substituted β-nitrostyrenes and trans-chalcone with various C–H acidic hydrocarbons, as well as reactions involving pyrazolones as nucleophiles. These studies confirm the potential of o-2 as a base catalyst for C–C bond-forming reactions, although such transformations can be effectively catalyzed by numerous other known bases.

In summary, this is a carefully executed but largely routine study that lacks significant novelty. To strengthen the impact, the authors should consider applying o-2 to more challenging C–C bond-forming transformations.

Reviewer #2

(Remarks to the Author)
Authors effectively discovered that their carborane species stabilized by N-heterocyclic bulky carbenes can behave as a super base. I think this is a very interesting discovery as the landscape of superbases is not vast and there are a limited number of classes. Furthermore, I do not think there currently exists a polyhedral borane molecule that can be classified as a superbase (though it has been shown that stabilized boranes can behave as such: ACIE Volume62, Issue36 September 4, 2023e202308467) and so this makes it also interesting from a boron chemistry standpoint. I generally believe that this work has a potential of being very important and exciting to the general readership of chemists.

My general critique is that authors use quite a bit of over-generalizations in the text, so I encourage the authors to proof read their work again and correct these parts (a few most prolific examples are listed in the comments below but there are more and the onus of fixing these is on the authors). I also have also several technical critiques that need to be addressed before this work can be published.

I would remove the “changing the game” part in the title, it is too superficial. Every study is supposed to be “changing the game”.

It is unclear why cationic nature is a prerequisite for Lewis basicity for boron compounds as authors argue in their work (there are examples, see below, that certainly challenge this notion). Authors should clarify this point, as their introduction can mislead the readers.

I do not understand what “antagonistic hydrogen atoms ” is supposed to mean.

Likewise, this part makes little sense: “the bridging hydrogen could serve as a proton source—thus mimicking the behavior of a frustrated Lewis pair.” How is what authors describe here mimics FLP?

Can authors depict (explicitly draw out) clearly the H that is being engaged in the molecules in Scheme 2? It might not be obvious for some readers.

Authors need to provide a more quantitative measure for the basicity of their claimed carborane superbase and benchmark it against other superbase species. E.g. (Chem. Sci., 2024,15, 10018-10026, Figure 1).

On a subject of umpolung (and my comment above), authors should compare their system (boron-centered base) to other polynuclear boron species. These were never claimed as superbases, but there are bases nevertheless based on their chemical reactivity: ACIE Volume132, Issue20 May 11, 2020 Pages 7800-7805; Chem Volume 5, Issue 9 P2461-2469 September 12, 2019; Nature Communications
volume 8, Article number: 15022 (2017)

Also, do authors expect the basicity can be tuned by the nature of NHC?

Authors need to reorganize their catalytic reaction work in the paper better as there are a number of experiments that are shown in the supporting information but are not directly mentioned and referenced/explained in the body of the manuscript. This is confusing and chaotic.

Reported polymers need to be characterized and molecular weight determined (as well as end group) if the authors intend to claim successful anionic polymerization.

I do not understand this statement: “Surprisingly, the reaction of o-2 with water—typically a dead end for most carboranes—proceeds to an equilibrium similar to that seen with methanol, yielding protonated o-2 and most likely the OH^- counterion.” I would not generalize this statement as the majority of carboranes (e.g., 12 vertex ones) are actually stable in water. Perhaps, authors refer to the subset of carboranes they are working on, and if so, this needs to be explicitly stated.

This sentence is too loaded and makes no sense: “These results demonstrate that umpolung of polyhedral (car)boranes via electron enrichment—such as through carbene adduct formation—and their application in Lewis-base catalysis or anionic polymerization may offer a powerful and versatile strategy for future developments in synthetic chemistry. ” What is electron enrichment?

Reviewer #3

(Remarks to the Author)
The paper, "Changing the Game in Polyhedral Dicarbaboranes Driven Organocatalysis – Umpolung of Acids to Superbases" by Jan Vrána, Zuzana Kapustová, Martin Kamlar, Peter Šramel, Petr Vosáhlo, Zdeňka Růžičková, Josef Holub, Jan Veselý, and Aleš Růžička, reports the synthesis and catalytic application of a superbase derived from closo-1,2-dicarbadecaborane(10) and N-heterocyclic carbenes (NHCs). The neutral adduct o-2 is protonated with HCl to form the cationic species o-2a, which exhibits superbasic properties and catalyzes various C–C bond-forming reactions, including aldol condensation, Michael addition, and Robinson annulation, with seemingly high efficiency.
However, while the formation of cationic boron clusters is remarkable, the paper's interpretation of the data raises several concerns. The authors report that the bridging hydrogen atoms in B–H–B moieties appear at -4.18 to -4.25 ppm in the 1H-NMR, a value typical for hydrides. This suggests that the protonation step involves oxidation, yet the paper does not clarify what is oxidized to account for the positive charge. The extremely small intensity of the signal near -4.3 ppm is also puzzling, and the absence of B–H resonances in the 1H-NMR is notable. A 11B decoupled NMR experiment, which is standard for such systems, would have provided crucial information but is not included.
Regarding catalytic activity, the use of a superbase for reactions like the Knoevenagel condensation is questionable. Conventional bases such as pyridine, piperidine, pyrrolidine, or ethylenediamine are effective and much easier to handle. The authors do not convincingly argue why a superbase is necessary, especially given that strong bases may hinder proton transfer kinetics. The paper's explanations for the structural and catalytic results are not fully convincing, and the lack of detailed NMR data weakens the overall interpretation.
In summary, while the formation of cationic boron clusters is an interesting achievement, the paper's explanations and experimental data do not fully support the proposed mechanisms and catalytic applications. The absence of key NMR experiments and the lack of a clear rationale for using a superbase in place of conventional bases are significant shortcomings.
Based on all of this, I cannot accept this work, and it should be sent to a much more specialized journal.

Version 1:

Reviewer comments:

Reviewer #1

(Remarks to the Author)
In response to my concerns regarding the significance of the reported base-catalyzed reactions, the authors stated: “What would be a more challenging C–C bond-forming transformation than a spirocyclization delivering a diastereomerically pure product?”

However, the reported double Michael addition leading to these spirocyclic compounds is not unprecedented. A recent study describes the synthesis of compound 8a, apparently as the “meso” isomer (J. Serbian Chem. Soc. 2022, 87, 813–827). Using triethanolamine as the base, an isolated yield of 72% was obtained, which increased to 82% under microwave conditions.
Furthermore, numerous reports describe spirocyclizations via double Michael addition using 1,5-diphenyl-1,4-pentadien-3-one as the substrate. In most cases, simple bases were employed, including:

• Triethylamine (ARKIVOC, 2024, 8, 202412321)
• Sodium carbonate (Scientific Reports 2021, 11, 22834)
• Potassium acetate (Heterocycles 2021, 102, 93–104)
• Potassium hydroxide (Letters in Organic Chemistry 2019, 16, 983–995; ChemistrySelect 2017, 2, 4006–4009)
• Triethanolamine (Synthetic Communications 2018, 48, 1747–1754) etc.

In these studies, the reactions proceeded efficiently, typically affording high isolated yields. There is simply no need for superbases.

Again, I would encourage the authors to identify more challenging catalytic C-C bond formation reactions to justify publication in this Journal.

On a second note, under what conditions have these acid-base reactions of o-2-H+ with P1-tBu, P1-H and guanidine been performed? It is stated in the SI that after mixing the compounds in acetonitrile the NMR spectra were taken immediately. Have these reactions been heated to establish equilibrium? No information is provided.

In summary, this is a carefully executed but on the catalytic part largely routine study that lacks novelty. Therefore, I cannot recommend this work for publication. It should be sent to a more specialized journal.

Reviewer #2

(Remarks to the Author)
Reviewers have addressed my questions, I have no further comments and recommend this work for publication.

Reviewer #3

(Remarks to the Author)
The authors have not convinced me with their explanations. I find that the work is well done; I have no remarks about that, but I am not satisfied. I see it as a curiosity. The authors present it as a great novelty that it is a superbase. First, they should define the difference between a superbase and a strong base. For example, they say, "Complete deprotonation to regenerate... in the presence of strong bases such as LDA or tBuOK." In my understanding, LDA is a superbase, although I would call it a very strong base. I emphasize this because the authors differentiate between them, e.g., "direct experimental evidence of its superbasic character." However, I believe that the concept of superbase is misinterpreted, not only by these authors but by many others. For instance, a superbase like BuLi does not participate in catalytic processes. Nevertheless, the authors themselves say, "...we assume that the protonation/deprotonation of o-2 is driven kinetically and the pKa cannot fully evaluate the basicity of this carborane." I completely agree with this. Regarding the bridging hydrogen, it seems clear to me that it becomes a hydride. It is a similar case to diborane, where the bridging protons can be thought of as having hydride character due to their involvement in three-center bonding. This configuration allows for significant electron delocalization, which increases electron density around the bridging protons. We could say that it is the same as what happens in organometallic chemistry, where the formation of M-H bonds often involves the reaction of a metal complex with a hydride source, which can include protonation steps or acid treatment. For example, treating a metal complex with a strong acid can lead to the formation of metal hydrides by transferring a proton to the metal, thereby stabilizing the resulting M-H bond. For me, it is clear that this is a paper dedicated to organic chemistry, and the authors want o-2 to be a reagent. However, I believe it is not an easily manageable reagent. A superbase to be interesting as a catalyst must combine stability, availability, high reactivity, selectivity, compatibility, non-toxicity, and cost-effectiveness. The majority of these points are not met by o-2. It cannot compete with commercial superbases based on nitrogen or phosphorus. Who would undertake the synthesis of o-2 starting from dicarbadodecaborane? To begin with, at what price would it be found? And where? Would it be one of the first superbases someone would choose? Certainly not. Therefore, I believe this well-executed work should be submitted to a more specialized journal. The application of this work is directed towards organic chemistry, not towards the development of boron clusters that I understand the authors have developed. Even if it is aimed at organic chemistry, if it cannot be used, what is its purpose?

Open Access This Peer Review File is licensed under a Creative Commons Attribution 4.0 International License, which permits use, sharing, adaptation, distribution and reproduction in any medium or format, as long as you give appropriate credit to the original author(s) and the source, provide a link to the Creative Commons license, and indicate if changes were made.
In cases where reviewers are anonymous, credit should be given to 'Anonymous Referee' and the source.
The images or other third party material in this Peer Review File are included in the article's Creative Commons license, unless indicated otherwise in a credit line to the material. If material is not included in the article's Creative Commons license and your intended use is not permitted by statutory regulation or exceeds the permitted use, you will need to obtain permission directly from the copyright holder.
To view a copy of this license, visit https://creativecommons.org/licenses/by/4.0/

REVIEWER COMMENTS

Reviewer #1 (Remarks to the Author):

In this contribution, Ruzicka and Vesely report on the high basicity of the previously described bis-carbene–carborane adduct o-2, which, according to the authors, exceeds the intrinsic basicity of the parent carbene. As expected, various C–H acidic hydrocarbons with pK_a values (in DMSO) ranging from 10 to 14 were fully deprotonated, and the corresponding salts were isolated in good yields. These compounds were comprehensively characterized by NMR spectroscopy and single-crystal X-ray diffraction.

It would be beneficial if the authors provided the exact pK_a values of all acids employed in the study (see Scheme 2). These values are readily accessible from the Bordwell pK_a database, which is publicly available online.

These were added to appropriate Schemes.

In addition, the applicability of o-2 as an organo-superbase catalyst was demonstrated in several standard base-catalyzed transformations, including the aldol condensation of butyraldehyde and Michael additions of substituted β-nitrostyrenes and trans-chalcone with various C–H acidic hydrocarbons, as well as reactions involving pyrazolones as nucleophiles. These studies confirm the potential of o-2 as a base catalyst for C–C bond-forming reactions, although such transformations can be effectively catalyzed by numerous other known bases.

The reviewer is only partially correct here. There is no other base known, which would react with all the substrates mentioned, tolerating all functional groups present, perform so efficiently at ambient (or close to ambient) conditions, accepting wet solvents, and most importantly work under real catalytic conditions.

These claims are based on comparative experiments of our system with other known bases and superbases as tBuOK, nBuLi, various carbenes, phosphazenes or guanidines, provoked by this review and reports of other reporters.

In summary, this is a carefully executed but largely routine study that lacks significant novelty. To strengthen the impact, the authors should consider applying o-2 to more challenging C–C bond-forming transformations.

We strongly disagree here, given transformations look like to be of the routine interest, but these were performed under real catalytic conditions. Versatility, efficiency at mild conditions and broad scope of reactions are requested novelties. What would be more challenging C–C bond-forming transformation than spirocyclization giving diastereoselectively pure products. We started our investigations from basic transformations and logically ended up with the challenging ones.

There is no catalytic system based on electron deficient element species – in addition, superbasic, at the same time with low nucleophilicity, and tolerant to sensitive functional groups and water.

Reviewer #2 (Remarks to the Author):

Authors effectively discovered that their carborane species stabilized by N-heterocyclic bulky carbenes can behave as a super base. I think this is a very interesting discovery as the landscape of superbases is not vast and there are a limited number of classes.

The benchmark reactions with other substrates and superbases showing our system is robust, water-tolerant and efficient enough to overpass known phosphazenes, carbenes and guanidine superbases in deprotonation reactions. This is also supported by DFT calculations.

Furthermore, I do not think there currently exists a polyhedral borane molecule that can be classified as a superbase (though it has been shown that stabilized boranes can behave as such: ACIE Volume62, Issue36, September 4, 2023e202308467) and so this makes it also interesting from a boron chemistry standpoint.

A paragraph about this issue is added. These compounds are known, they are usually of anionic nature and are not able to perform catalytically.

I generally believe that this work has a potential of being very important and exciting to the general readership of chemists.

My general critique is that authors use quite a bit of over-generalizations in the text, so I encourage the authors to proof read their work again and correct these parts (a few most prolific examples are listed in the comments below but there are more and the onus of fixing these is on the authors). I also have also several technical critiques that need to be addressed before this work can be published.

Many thanks for these comments. We tried to fix it.

I would remove the “changing the game” part in the title, it is too superficial. Every study is supposed to be “changing the game”.

We changed it.

It is unclear why cationic nature is a prerequisite for Lewis basicity for boron compounds as authors argue in their work (there are examples, see below, that certainly challenge this notion). Authors should clarify this point, as their introduction can mislead the readers.

This sentence is misunderstood. We started with hypothesis that cationic compounds could work as hydrogenation or acid catalysts, but only negligible activity was achieved, so we switched to its precursor, which should have neutral and basic nature. We fixed it.

I do not understand what “antagonistic hydrogen atoms ” is supposed to mean.

This was an expression that protic and hydridic H atoms are really close in the structure. Is explained in the text.

Likewise, this part makes little sense: “the bridging hydrogen could serve as a proton source—thus mimicking the behavior of a frustrated Lewis pair.” How is what authors describe here mimics FLP?

This was an expression that protic and hydridic H atoms are really close in the structure. Is explained in the text.

Can authors depict (explicitly draw out) clearly the H that is being engaged in the molecules in Scheme 2? It might not be obvious for some readers.

We disagree with this suggestion, it is obvious to the readership, the only confusing could be the case of substrate H**g** – 2-coumaronone, which after deprotonation opens the heterocyclic ring and subsequently undergoes the Claisen condensation, which is explained in the text and SI.

Authors need to provide a more quantitative measure for the basicity of their claimed carborane superbase and benchmark it against other superbase species. E.g. (Chem. Sci., 2024,15, 10018-10026, Figure 1).

Is added and explained in conclusion.

On a subject of umpolung (and my comment above), authors should compare their system (boron-centered base) to other polynuclear boron species. These were never claimed as superbases, but there are bases nevertheless based on their chemical reactivity: ACIE Volume132, Issue20 May 11, 2020 Pages 7800-7805; Chem Volume 5, Issue 9 P2461-2469 September 12, 2019; Nature Communications
volume 8, Article number: 15022 (2017)

Is added and explained in the text.

Also, do authors expect the basicity can be tuned by the nature of NHC?

We think so, but in our case the use of “aliphatic” NHC provided extremely reactive, and so instable, species, which maybe could perform slightly better as a superbase, but for sure will not catalyze reported transformations.

Authors need to reorganize their catalytic reaction work in the paper better as there are a number of experiments that are shown in the supporting information but are not directly mentioned and referenced/explained in the body of the manuscript. This is confusing and chaotic.

We added and reorganized the figures.

Reported polymers need to be characterized and molecular weight determined (as well as end group) if the authors intend to claim successful anionic polymerization.

Unfortunately, as reported in the literature, it is not possible for this material, as the solubility even in fluorinated alcohols and chlorobenzenes is so low. The GPC exhibited only minor signal for species with Mw’ of ~10kDa. This is exactly the same as reported in the literature, we characterized it by m.p. and infrared where there no trace for C=C vibration-comment is added.

I do not understand this statement: “Surprisingly, the reaction of o-2 with water—typically a dead end for most carboranes—proceeds to an equilibrium similar to that seen with methanol, yielding protonated o-2 and most likely the OH^- counterion.” I would not generalize this statement as the majority of carboranes (e.g., 12 vertex ones) are actually stable in water. Perhaps, authors refer to the subset of carboranes they are working on, and if so, this needs to be explicitly stated.

It is corrected now.

This sentence is too loaded and makes no sense: “These results demonstrate that umpolung of

polyhedral (car)boranes via electron enrichment—such as through carbene adduct formation—and their application in Lewis-base catalysis or anionic polymerization may offer a powerful and versatile strategy for future developments in synthetic chemistry. ” What is electron enrichment?

The meaning of “enrichment” was the addition of the electron density to the skeletal electron count by attaching the NHCs. It is explained.

Reviewer #3 (Remarks to the Author):

The paper, "Changing the Game in Polyhedral Dicarbaboranes Driven Organocatalysis – Umpolung of Acids to Superbases" by Jan Vrána, Zuzana Kapustová, Martin Kamlar, Peter Šramel, Petr Vosáhlo, Zdeňka Růžičková, Josef Holub, Jan Veselý, and Aleš Růžička, reports the synthesis and catalytic application of a superbase derived from closo-1,2-dicarbadecaborane(10) and N-heterocyclic carbenes (NHCs). The neutral adduct o-2 is protonated with HCl to form the cationic species o-2a, which exhibits superbasic properties and catalyzes various C–C bond-forming reactions, including aldol condensation, Michael addition, and Robinson annulation, with seemingly high efficiency. However, while the formation of cationic boron clusters is remarkable, the paper's interpretation of the data raises several concerns. The authors report that the bridging hydrogen atoms in B–H–B moieties appear at -4.18 to -4.25 ppm in the 1H-NMR, a value typical for hydrides. This suggests that the protonation step involves oxidation, yet the paper does not clarify what is oxidized to account for the positive charge. The extremely small intensity of the signal near -4.3 ppm is also puzzling, and the absence of B–H resonances in the 1H-NMR is notable. A 11B decoupled NMR experiment, which is standard for such systems, would have provided crucial information but is not included.

The addition of proton forming the B-H-B bridge does not change the electronic properties of the cluster itself. Considering the standard Wade-Mingos rules, the cluster is formally *arachno*-shaped (as for example *arachno*-$C_2B_8H_{14}$) bearing 13 skeletal electron pairs. The addition of one hydrogen atom increases the count by one electron, however the positive charge decreases it again, so the SEP-count does not change and thus the addition involves no redox change within the cluster. Moreover, the bridging hydrogen atoms in polyhedral boranes have protic character and usually can be removed by reaction with a base. In many papers they are even referred as bridging protons. The downfield shift is typical for bridging protons (terminal B-H groups can be typically found in the range of −1–3 ppm). Many papers containing polyhedral borane compounds, which do not deal with the cluster transformation, e.g. compounds using the boranes as weakly coordinating anions, ligand scaffolds etc., do not include the $^{1}H\{^{11}B\}$ experiments (see for example *J. Am. Chem. Soc.* 2025, 147, 29, 25478; *Chem. Sci.*, 2025, 16, 3705 as recent examples)). As both *o*-**2** and *o*-**2**H^+ have been fully characterized earlier, we did not include these experiments as well. However, we added them to the revised version of the SI with the assignment of the signals. All compounds revealed the same pattern of 1:2:2:1:2 signals with negligible differences in their chemical shifts.

Regarding catalytic activity, the use of a superbase for reactions like the Knoevenagel condensation is questionable. Conventional bases such as pyridine, piperidine, pyrrolidine, or ethylenediamine are effective and much easier to handle. The authors do not convincingly argue why a superbase is necessary, especially given that strong bases may hinder proton transfer kinetics. The paper's explanations for the structural and catalytic results are not fully convincing, and the lack of detailed NMR data weakens the overall interpretation.

We agree with the referee, that the basic Knoevenagel and aldol condensations were not fully justified in the paper. The main goal of this paper was to introduce completely new type of applicable superbase catalyst (first boron-based), which has different chemical properties when compared with the standard superbases. It is even less nucleophilic, though stronger than commercially available bases and even water tolerant. To highlight this, we added several experiments comparing the basicity of *o*-**2** with BTMG, MTBD, P_1-H and P_1-*t*Bu (see Scheme).

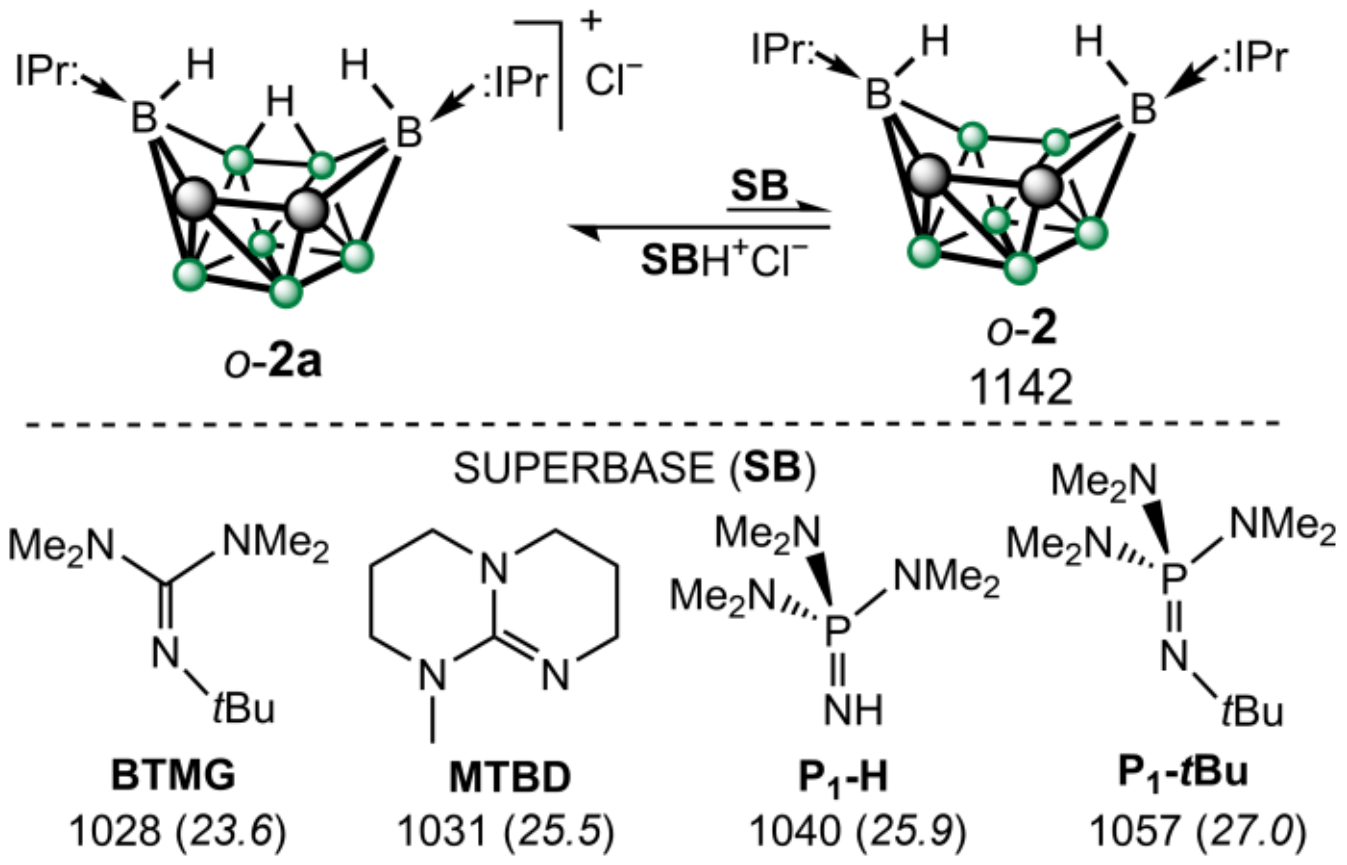

First, we tested the basicity experimentally by treating *o*-**2**H$^+$ with the superbases (Scheme). Both guanidines and the phosphazene P_1-H deprotonated *o*-**2**H$^+$ partially forming a similar dynamic equilibrium between the neutral and ionized form shifted towards the parent *o*-**2**H$^+$. Interestingly, the *o*-**2**/*o*-**2**H$^+$ ratio was nearly identical as in the case of the compound *o*-**2**h, which means, that acetylacetonate deprotonates *o*-**2**H$^+$ with the same efficiency as the superbases despite having dramatically lower pK_A. Thus, we assume that the protonation/deprotonation of *o*-**2** is driven kinetically and the pK_A cannot fully evaluate the basicity of this carborane. The phosphazene P_1-*t*Bu revealed no deprotonation of *o*-**2**H$^+$, most probably due to the steric reasons. To screen the reversibility of the reactions, we tested also the reversed reactions (**SB**H$^+$ + *o*-**2**), which ended with the same result, the **SB**H$^+$ has been only partially deprotonated, confirming there is no thermodynamical equilibrium. Therefore, we calculated the protonation affinities (in itallics in parentheses), which show, that protonation of *o*-**2** is more favourable. The most striking difference between *o*-**2** and the tested superbases is the behaviour towards water. Guanidines and phosphazenes are hygroscopic and the interaction with water implies higher loading in catalytic reactions or the water has to be removed, e.g. by molecular sieves. On the contrary, *o*-**2** fully tolerates water. As mentioned earlier, dissolving it in overstoichiometric amount of water (combined with acetonitrile for higher solubility) yields compound *o*-**2**w, which after evaporation even at room temperature yields again pure *o*-**2**, which, to the best of our knowledge, has not been described for any base.

Next, to compare the basicity, we conducted the aldol and Knoevenagel condensations and the Michael addition catalyzed by **MTBD** and **P_1-H**. While the addition of acetylacetone to *trans*-chalcone was completed within the same time with equal catalyst loading, the condensations required considerably higher amount of the superbase, highlighting the water-tolerance of the carborane *o*-**2**. Moreover, in the Knoevenagel condensation, *o*-**2** facilitates selectively the condensation, while **MTBD** and **P_1-H** revealed the byproduct of Thorpe reaction, prepared in the stoichiometric reaction of *o*-**2** with malononitrile (*o*-**2f'**). Similar trend has been found in the aldol condensation of butyraldehyde. **MTBD** and **P_1-H** required five times higher loading to match the performance of *o*-**2**.

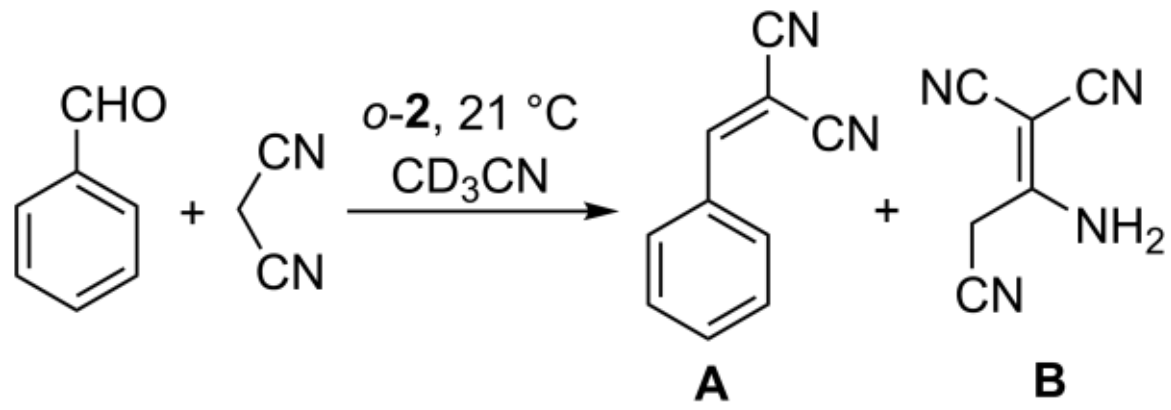

Entry	Catalyst	Cat. loading (mol%)	Conversion[a] (%)	Time (h)	A/B ratio[a] (%)
1	*o*-**2**	0.5	60	24	60/0
2	*o*-**2**	0.5	90	48	90/0
3	*o*-**2**	1	21	2	65/0
4	*o*-**2**	1	99	16	99/0
5	*o*-**2**	3	79	2	79/0
6	*o*-**2**	3	97	6	97/0
7	*o*-**2**	5	99	2	99/0
8	**MTBD**	5	56	2	53/3
9	**MTBD**	5	94	24	89/5
10	**MTBD**	10	61	1	56/5
11	**MTBD**	10	65	2	59/6
12	**MTBD**	20	97	1	87/10
13	**MTBD**	20	88	2	78/10
14	**P_1-H**	10	85	1	76/9
15	**P_1-H**	10	91	2	79/12
16	**P_1-H**	20	80	1	60/20
17	**P_1-H**	20	82	2	62/20

[a] determined by ^{1}H NMR

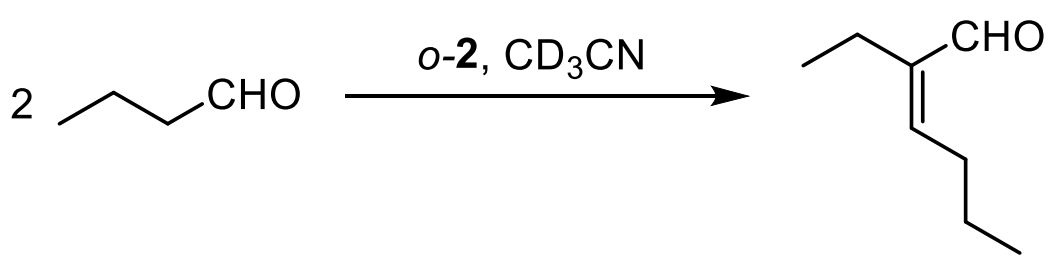

Entry	Catalyst	Cat. loading (mol%)	H_2O (mol%)	Temperature (°C)	Time (h)	Yield (%)[a]
**1**	*o*-**2**	10	0	21	1	<5
**2**	*o*-**2**	10	0	21	24	32
**3**	*o*-**2**	2	0	21	1	24
**4**	*o*-**2**	2	0	21	2	92
**5**	*o*-**2**	1	0	21	2	71
**6**	*o*-**2**	1	0	21	24	97
**7**	*o*-**2**	10	20[b]	21	2	24
**8**	*o*-**2**	10	20[b]	21	24	90
**9**	*o*-**2**	2	20[b]	21	2	32
**10**	*o*-**2**	2	20[b]	21	24	99
**11**	*o*-**2**	2	0	30	1	57
**12**	*o*-**2**	2	0	30	2	95
**13**	**MTBD**	1	0	21	2	<5
**14**	**MTBD**	1	0	21	24	76
**15**	**MTBD**	5	0	21	2	<5
**16**	**MTBD**	5	0	21	24	98
**17**	**P_1-H**	1	0	21	2	<5
**18**	**P_1-H**	1	0	21	24	<5
**19**	**P_1-H**	5	0	21	2	25
**20**	**P_1-H**	5	0	21	24	97

[a] determined by ^{1}H NMR; [b] water was added with the catalyst

In addition, the functional group tolerance was tested in the series of conjugate addition and spirocyclization reactions, where our system works with no byproducts, but other superbases promote formation of mixtures.

In summary, while the formation of cationic boron clusters is an interesting achievement, the paper's explanations and experimental data do not fully support the proposed mechanisms and catalytic applications. The absence of key NMR experiments and the lack of a clear rationale for using a superbase in place of conventional bases are significant shortcomings.

We are pretty sure, we fixed these issues by adding some explanatory Figures and text.

Based on all of this, I cannot accept this work, and it should be sent to a much more specialized journal.

Reviewer #1 (Remarks to the Author):

In response to my concerns regarding the significance of the reported base-catalyzed reactions, the authors stated: “What would be a more challenging C–C bond-forming transformation than a spirocyclization delivering a diastereomerically pure product?”

However, the reported double Michael addition leading to these spirocyclic compounds is not unprecedented. A recent study describes the synthesis of compound 8a, apparently as the “meso” isomer (J. Serbian Chem. Soc. 2022, 87, 813–827). Using triethanolamine as the base, an isolated yield of 72% was obtained, which increased to 82% under microwave conditions.
Furthermore, numerous reports describe spirocyclizations via double Michael addition using 1,5-diphenyl-1,4-pentadien-3-one as the substrate. In most cases, simple bases were employed, including:

• Triethylamine (ARKIVOC, 2024, 8, 202412321)
• Sodium carbonate (Scientific Reports 2021, 11, 22834)
• Potassium acetate (Heterocycles 2021, 102, 93–104)
• Potassium hydroxide (Letters in Organic Chemistry 2019, 16, 983–995; ChemistrySelect 2017, 2, 4006–4009)
• Triethanolamine (Synthetic Communications 2018, 48, 1747–1754) etc.

In these studies, the reactions proceeded efficiently, typically affording high isolated yields. There is simply no need for superbases.

Again, I would encourage the authors to identify more challenging catalytic C-C bond formation reactions to justify publication in this Journal.

We thank the reviewer for this detailed and constructive comment. We agree that double Michael additions leading to spirocyclic frameworks, including those derived from 1,5-diphenyl-1,4-pentadien-3-one, have been reported in the literature and can proceed efficiently under relatively simple basic conditions. In this context, we acknowledge the recent study reporting the synthesis of compound 8a, apparently obtained as the “meso” isomer (J. Serbian Chem. Soc. 2022, 87, 813–827, Scientific Reports 2021, 11, 22834), further demonstrating that such transformations are not unprecedented.
However, the key contribution of the present work does not lie in the discovery of the spirocyclization itself, but in the exceptional catalytic efficiency of *o*-**2**. Specifically, *o*-**2** promotes the transformation efficiently at only 1 mol% catalyst loading, whereas previously reported protocols typically require substantially higher catalyst loadings (e.g., Heterocycles 2021, 102, 93–104). Moreover, the reaction proceeds with complete diastereoselectivity, affording exclusively a single diastereoisomer.
Importantly, diastereoselectivity was not evaluated in all of the previously reported studies (e.g., Synthetic Communications 2018, 48, 1747–1754). Moreover, compared with methodologies relying on stoichiometric amounts of base (Arkivoc 2024, 8, 202412321; Scientific Reports 2021, 11, 22834; Letters in Organic Chemistry 2019, 16, 983–995; ChemistrySelect 2017, 2, 4006–4009), the present catalytic system represents a more efficient and atom-economical alternative.
Therefore, although related spirocyclizations can be achieved under conventional basic conditions, the previously reported systems generally do not combine low catalyst loading with exclusive

diastereocontrol. In contrast, the catalytic system described herein achieves both features simultaneously, which we believe represents the key advancement and distinctive value of this work.

On a second note, under what conditions have these acid-base reactions of o-2-H+ with P1-tBu, P1-H and guanidine been performed? It is stated in the SI that after mixing the compounds in acetonitrile the NMR spectra were taken immediately. Have these reactions been heated to establish equilibrium? No information is provided.
We selected acetonitrile as the most suitable solvent because it has previously been used to describe the *o*-**2**/weak acid equilibrium. The NMR spectra were recorded immediately over the temperature range of −20 to 60 °C, and no effect of temperature was observed. This information has been added to both the main text and the Supporting Information.

In summary, this is a carefully executed but on the catalytic part largely routine study that lacks novelty. Therefore, I cannot recommend this work for publication. It should be sent to a more specialized journal.

Reviewer #2 (Remarks to the Author):

Reviewers have addressed my questions, I have no further comments and recommend this work for publication.

Reviewer #3 (Remarks to the Author):

The authors have not convinced me with their explanations. I find that the work is well done; I have no remarks about that, but I am not satisfied. I see it as a curiosity.
We must declare here: sixty years ago metal-catalyzed reactions were considered speculative or as a curiosity; today they are indispensable in virtually every organic and biochemical laboratory.
The authors present it as a great novelty that it is a superbase. First, they should define the difference between a superbase and a strong base. For example, they say, "Complete deprotonation to regenerate... in the presence of strong bases such as LDA or tBuOK." In my understanding, LDA is a superbase, although I would call it a very strong base. I emphasize this because the authors differentiate between them, e.g., "direct experimental evidence of its superbasic character." However, I believe that the concept of superbase is misinterpreted, not only by these authors but by many others. For instance, a superbase like BuLi does not participate in catalytic processes. Nevertheless, the authors themselves say, "...we assume that the protonation/deprotonation of o-2 is driven kinetically and the pKa cannot fully evaluate the basicity of this carborane." I completely agree with this.
We agree that “superbase” is used inconsistently in the literature. Our assignment of *o*-**2** as a superbase is based on multiple observations reported in the manuscript. While LDA, tBuOK, BuLi, and phosphazene reagents are variously called “very strong” or “superbasic” depending on context, we use “superbase” to indicate behavior that exceeds conventional solvent pKa scales (e.g., OH^- or alkoxides). In practice this means exceptionally high proton affinity or gas-phase basicity and, importantly, reactivity that cannot be predicted from solution pKa alone. For example rapid deprotonation of very weak C–H acids in aprotic media. On both computational metrics and

experimental reactivity tests reported here, *o*-**2** matches or surpasses those criteria, which is why we consider the term "superbase" appropriate for this compound.

Regarding the bridging hydrogen, it seems clear to me that it becomes a hydride. It is a similar case to diborane, where the bridging protons can be thought of as having hydride character due to their involvement in three-center bonding. This configuration allows for significant electron delocalization, which increases electron density around the bridging protons. We could say that it is the same as what happens in organometallic chemistry, where the formation of M-H bonds often involves the reaction of a metal complex with a hydride source, which can include protonation steps or acid treatment. For example, treating a metal complex with a strong acid can lead to the formation of metal hydrides by transferring a proton to the metal, thereby stabilizing the resulting M-H bond.

We fully understand the reviewer's perspective regarding the bridging hydrogen atom, as it participates in a three-center two-electron bond. However, we would like to emphasize two important aspects of this interaction.

First, according to Skeletal Electron Pair Theory, the bridging hydrogen does not affect either the electron count or the geometry of the cluster. This contrasts with the formation of metal hydrides, where oxidation of the central atom occurs together with the formation of a terminal M–H bond, in which the hydrogen atom may exhibit either positive or negative polarization.

Second, the bridging hydrogen is consistently protic in character. To the best of our knowledge, there is no evidence that such bridges exhibit hydride-like reactivity. In general, even hydride abstraction from terminal B–H bonds, which possess the appropriate polarity, is difficult. For example, several anionic carboranes are known to form stable salts with tritylium counterions without undergoing any reaction.

For me, it is clear that this is a paper dedicated to organic chemistry, and the authors want o-2 to be a reagent. However, I believe it is not an easily manageable reagent. A superbase to be interesting as a catalyst must combine stability, availability, high reactivity, selectivity, compatibility, non-toxicity, and cost-effectiveness. The majority of these points are not met by o-2. It cannot compete with commercial superbases based on nitrogen or phosphorus. Who would undertake the synthesis of o-2 starting from dicarbadodecaborane? To begin with, at what price would it be found? And where? Would it be one of the first superbases someone would choose? Certainly not. Therefore, I believe this well-executed work should be submitted to a more specialized journal. The application of this work is directed towards organic chemistry, not towards the development of boron clusters that I understand the authors have developed. Even if it is aimed at organic chemistry, if it cannot be used, what is its purpose?

Our manuscript reports fundamental physicochemical and reactivity data that demonstrate *o*-**2**'s exceptional basicity and unique reactivity profile; the primary goal is to establish its properties and potential, not to claim immediate commercial readiness as a general-purpose reagent.

On the other hand, we demonstrated, *o*-**2** is not dangerous reagent surviving "wet" or water-born conditions, we are able to prepare it in a multi-gram scale from commercial chemicals.

Despite practical constraints, o-**2** offers distinctive features (non-nucleophilic behavior, unusual kinetic protonation/deprotonation patterns, and computable gas-phase/thermodynamic metrics) that advance understanding of superbasic behavior and could enable niche applications or guide design of more practical derivatives. We will emphasize scientific insight and future translational paths rather than immediate reagent replacement.
